# Single-Cell Hemoprotein Diet Changes Adipose Tissue Distributions and Re-Shapes Gut Microbiota in High-Fat Diet-Induced Obese Mice

**DOI:** 10.4014/jmb.2308.08046

**Published:** 2023-09-22

**Authors:** Seungki Lee, Ahyoung Choi, Kyung-Hoon Park, Youngjin Cho, Hyunjin Yoon, Pil Kim

**Affiliations:** 1 Department of Biotechnology, the Catholic University of Korea, Bucheon 14662, Republic of Korea; 2 HemoLab Ltd. Co., Bucheon, Republic of Korea; 3 Department of Molecular Science and Technology, Ajou University, Suwon 16499, Republic of Korea

**Keywords:** Heme-SCP, iron, obesity, lipid, microbiota

## Abstract

We have previously observed that feeding with single-cell hemoprotein (heme-SCP) in dogs (1 g/day for 6 days) and broiler chickens (1 ppm for 32 days) increased the proportion of lactic acid bacteria in the gut while reducing their body weights by approximately 1~2%. To define the roles of heme-SCP in modulating body weight and gut microbiota, obese C57BL/6N mice were administered varied heme-SCP concentrations (0, 0.05, and 0.5% heme-SCP in high fat diet) for 28 days. The heme-SCP diet seemed to restrain weight gain till day 14, but the mice gained weight again later, showing no significant differences in weight. However, the heme-SCP-fed mice had stiffer and oilier bodies compared with those of the control mice, which had flabby bodies and dull coats. When mice were dissected at day 10, the obese mice fed with heme-SCP exhibited a reduction in subcutaneous fat with an increase in muscle mass. The effect of heme-SCP on the obesity-associated dyslipidemia tended to be corroborated by the blood parameters (triglyceride, total cholesterol, and C-reactive protein) at day 10, though the correlation was not clear at day 28. Notably, the heme-SCP di*et al*tered gut microbiota, leading to the proliferation of known anti-obesity biomarkers such as *Akkermansia*, *Alistipes*, *Oscillibacter*, *Ruminococcus*, *Roseburia*, and *Faecalibacterium*. This study suggests the potential of heme-SCP as an anti-obesity supplement, which modulates serum biochemistry and gut microbiota in high-fat diet-induced obese mice.

## Introduction

The rising obesity rate is a concern not only for individuals but is also a global public health concern. Obesity is directly linked to cardiovascular diseases [1] and has long been recognized as a main cause of type II diabetes mellitus, hypertension, and dyslipidemia [2]. Apart from the adverse metabolic effects, accumulating evidence indicates overweight and obesity as causes of a wide variety of human cancers, including colon, female breast (after menopause), kidney, pancreatic, ovarian, cervical, and prostate cancers [3]. The national health care system is burdened by the increased frequency of obesity and geriatric diseases, particularly in developing countries with an aging population. Obesity and overweight are not self-inflicted diseases, according to life insurance data and epidemiological studies, but they do pose a threat to global well-being [4].

Obesity is associated with dysregulation of lipid metabolism, which can cause aberrant blood lipid levels and ectopic fat accumulation. Various medications have been tested in an attempt to modulate lipid metabolism [5]. Nutrients like carbohydrates and fatty acids play a major role in controlling lipid metabolism. However, a growing numbers of studies have demonstrated a correlation between lipid levels and the composition of the gut microbiota [6]. Mechanistic connections between lipid metabolism and microbial metabolites have been revealed in animal models [7-9]. Reshaping the gut microbiome can be a promising strategy to reshuffle lipid metabolic balance in obese individuals on high-fat diet. Human microbiota exerts beneficial and detrimental roles during the lifetime of a person, including birth, aging, sickness, and death [10]. A Western-style diet-an unhealthy diet with high consumption of fats-was found to aggravate cardiovascular disease by the imbalanced gut microbiota. Choline, high in dietary fats, stimulates facultatively anaerobic *Enterobacteriaceae* such as *Escherichia coli* to produce trimethylamine (TMA), which is oxidized in the liver to trimethylamine *N*-oxide (TMAO) [11]. A small organic compound TMAO alters cholesterol and bile acid metabolism, activates vascular inflammatory pathways, and enhances the hyperactivity of platelets, which consequently leads to the formation of atherosclerotic plaques [12]. Recent studies demonstrated that the gut microbiota of obese patients was distinguishable from those of healthy people [13-15], which suggests the probability that alterations in the gut microbiota can rewire the energy metabolism because the energy harvesting capacity varies between bacterial species [16-18].

Heme, an iron-centered porphyrin, functions as a prosthetic group in various hemoproteins (heme-bound proteins). Hemoproteins are extensively exploited in every life form and include globin proteins—hemoglobin, myoglobin, and leghemoglobin— cytochromes in electron transfer chain, serum albumin regulating the intravascular colloid osmotic pressure, many oxygenases (degradation pathways) and reductases (biosynthetic pathways), catalases and heme-peroxidases inactivating toxic reactive oxygen species, sensor proteins in signal transductions for CO, NOX, O_2_, and H_2_O_2_. Even commensal gut bacteria such as lactic acid bacteria utilize hemoproteins in many crucial biological activities. Although lactic acid bacteria have been historically regarded as non-respiratory facultative anaerobes, some species stimulate the aerobic growth utilizing their incomplete respiratory chains in response to the presence of bioavailable heme [19]. For example, the supplementation of hemin, a purified heme sodium salt derived from bovine blood, increased biomass and survivability of *Lactococcus lactis* [20, 21]. Thus, differential demand for heme among bacterial species can trigger reshaping of anaerobic gut microbiota upon hemoprotein-enriched food intake. Alteration of gut microbiota and metabolism by dietary iron supplementation has been extensively studied [22]. However, it should be noted that the majority of iron in human body, approximately 70%, is chelated within the porphyrin structure of heme or hemoprotein [23].

We previously demonstrated that hemoprotein-supplement (heme-SCP 1 g with a commercial feed 25 g per day for 6 days) containing single-cell biomass of a *Corynebacterium glutamicum* strain (*C. glutamicum* st. hemoP) triggered reshaping of gut microbiota in small pet dogs (*n* = 4) [24]. Bacterial diversity was increased from 172 to 202 species and the proportion of Firmicutes bacteria was increased from 91.9 to 99.1% by the heme-SCP administration. Notably, the body weights of dogs were reduced by approximately 1~2% after heme-SCP administration. We observed that free-style administration of a dog-treat containing 0.2% heme-SCP could also influence the fecal bacterial proportions in pet dogs (*n* = 10): an increase in Firmicutes (54.7% to 73.7%) and decreases in Proteobacteria, Bacteroidestes, and Fusobacteria (5.4 to 3.8%, 32.9 to 16.8%, and 6.3 to 3.6%, respectively) [25]. We also witnessed broiler chickens (*n* = 160) exhibited reduction in weight gain caused by the heme-SCP administration (supporting information Data 1). Heme-SCP at 1 ppm lowered weight gain 2.8%(1533.9 _± 31.0_ g in 1 ppm heme-SCP group; 1579.3 _± 21.4_ g in no heme-SCP group) and promoted the colonization of lactic acid bacteria in the caecum (6.9 × 10^8^ CFU/g in 1 ppm heme-SCP group; 3.7 × 10^8^ CFU/g in no heme-SCP group). These accumulated clues encouraged us to study the possible modulatory effect of heme-SCP nutritional supplement through reshaping gut microbiota on a high-fat diet (HFD) mouse model.

## Materials and Methods

### High-Fat Diet Formulation

For animal staple diets, 0.5 g or 5 g heme-SCP (hemoP, Hemolab l. c., Korea) was combined with 1 kg of a high-fat diet (HFD12492; Research Diet Inc., USA). The high-fat diet was consisted of 26% protein, 26% carbohydrate, and 35% fat (w/w) with total calorie of 5.24 kcal/g (20% from protein, 20% from carbohydrate, and 60% from fat). The heme-SCP used in this study was consisted of 90.5% solid and 9.5% moisture: 4.0% α-amino nitrogen and 53.6% protein. The estimated heme content was 16.9 mg/g.

### Ethics Sstatement

All mice experiments were conducted in South-east Medi-Chem Institute Inc. (animal experimental facility registration #412, Korea). Mice were purchased from Hana Biotech Inc. (Korea) and reared for 7 days in an animal breeding facility. Animal experiments were performed in accordance with the guidelines of the institutional animal care and use committee (protocol No. SEMI-22-001). All feasible measures were taken to reduce animal suffering.

### Mouse Study Design

**Fig. 1:**
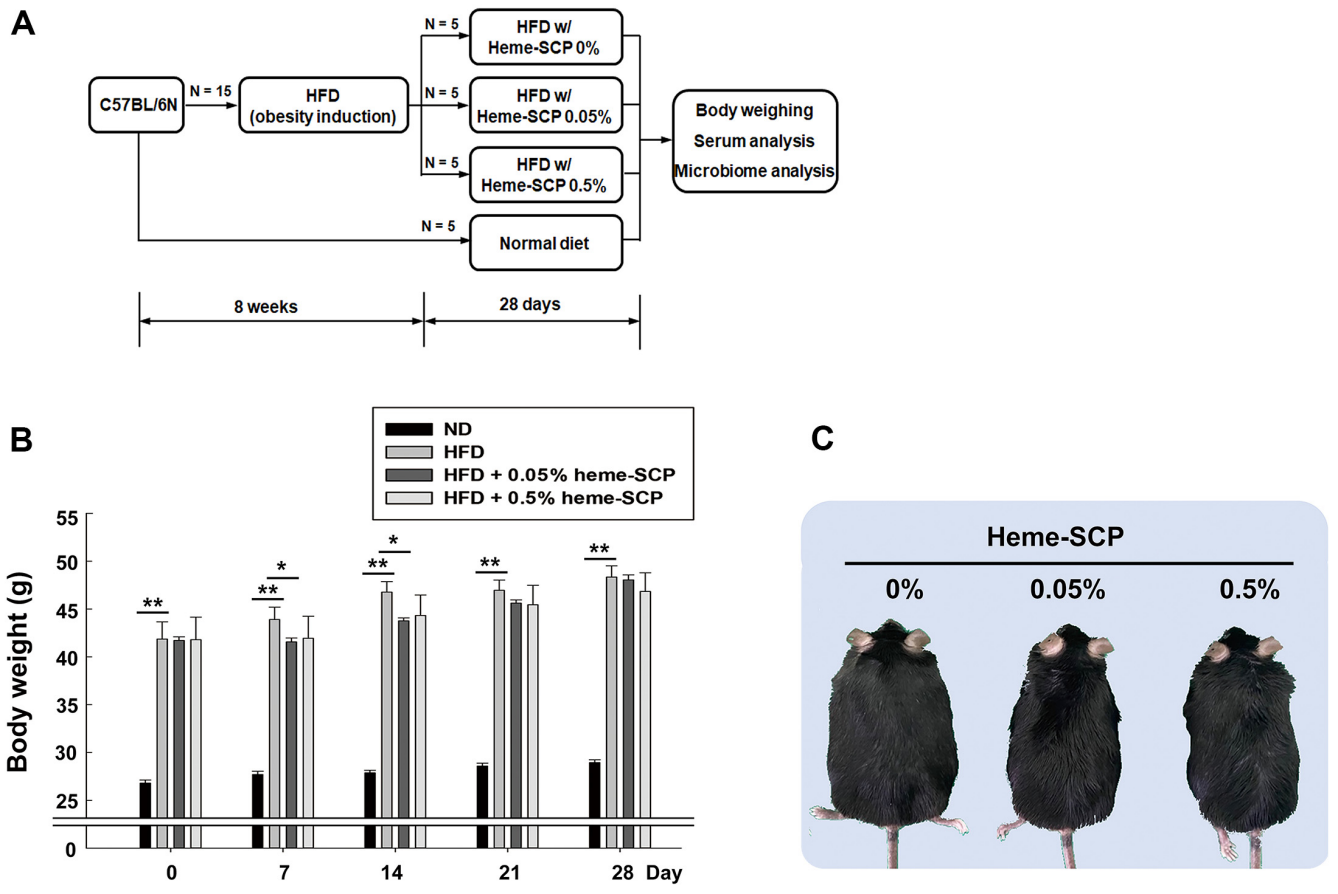
Body weight and shape of obese mouse administered heme-SCP for 28 days. Obesity was induced using high-fat diet (HFD) for 8 weeks and HFD mixed with 0, 0.05, and 0.5% heme-SCP was administered to mice for 28 days (**A**). Body weights (**B**) and body shapes (**C**) on day 28 were compared. Values are expressed as mean ± SEM (standard error of mean) for each group of five mice and the representative images are presented. Asterisks denote significant differences: *p value < 0.05 and **p value < 0.005.

**Fig. 2:**
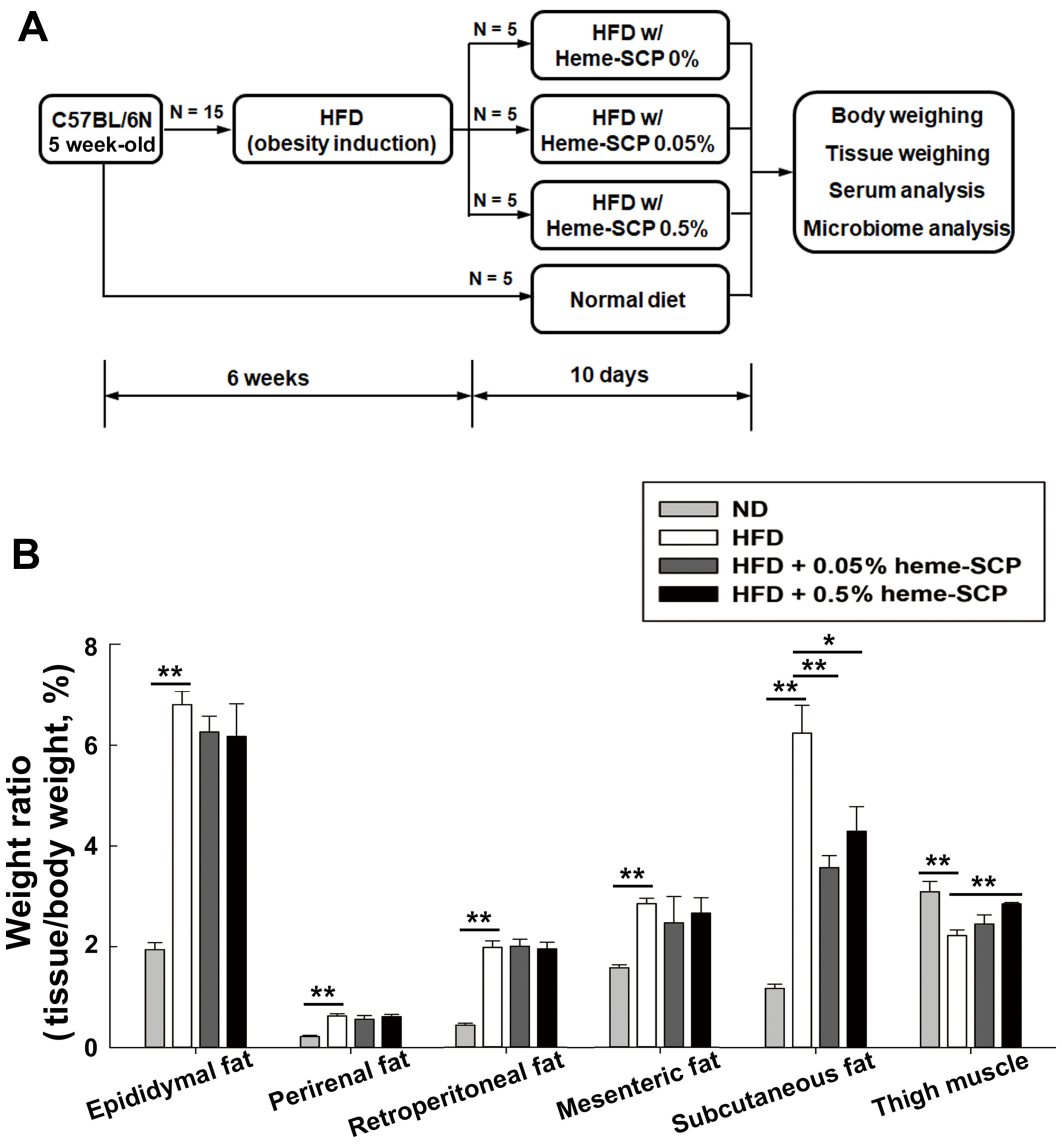
Ratio between tissue and body weight in obese mouse administered heme-SCP for 10 days. Obesity was induced using high-fat diet (HFD) for 6 weeks and HFD mixed with 0, 0.05, and 0.5% heme-SCP was administered to mice for 10 days (**A**). On day 10, the body and tissue weights were measured and the ratios (tissue/body weight) are plotted (**B**). Values are expressed as mean ± SEM for each group of five mice. Asterisks denote significant differences: *p-value < 0.05; **p-value < 0.005.

Five-week-old male mice (C57BL/6N) were housed in the facility, where access to food and drink was not restricted, and the lighting was adjusted to 12 h each day (07:00 to 19:00). Throughout the test, body weight and intake of food and water were measured every three days. Obese mice were induced by administering HFD for 8 weeks (1^st^ set; 28 days of heme-SCP administration) or for 6 weeks (2^nd^ set; 10 days of heme-SCP administration). Mice (*n* = 15) were separated into three groups of five to attain the same average body weight, following the obesity-induction period. Subsequently, the prepared diets (HFD supplemented with 0, 0.05, or 0.5 % heme-SCP) were administered to each mice group for 28 days (1^st^ set; Fig. 1A) or 10 days (2^nd^ set; Fig. 2A). The feces were collected at the end of the administration period and subjected to metagenomics.

### Autopsy

Mice were anesthetized by CO_2_ gas, and blood samples were taken from the abdominal aorta. The blood sample was placed at 18°C for 30 min and then centrifuged (1,200 ×*g*, 15 min, 4°C) to separate the serum. The serum samples were stored in a deep freezer until analyses. Tissues (epididymal, perirenal, retroperitoneal, mesenteric, and subcutaneous fat, and thigh muscle) were dissected to measure weights. Statistical validation was performed using a software (Starview statistical program). Significant differences were calculated using Student’s t-test and one-way analysis of variance (ANOVA) with Tukey’s post-hoc test.

### Biochemical Analysis of the Serum

The levels of triglyceride (TG) and total cholesterol (T-cholesterol) in the serum were measured using an autoanalyzer (model 7600II; Hitachi, Japan) in a biochemical institutional facility (Korea Non-clinic Test Support Center, Korea). The remaining serum samples were stored in a deep freezer for quantification using ELISA. The frozen serum samples were thawed in ice for 1 h before quantification. The C-reactive protein (CRP) in the serum samples were quantified using ELISA kits (ab222511, Mouse C Reactive Protein Simple Step ELISA Kit, Abcam Inc., USA) according to the protocol provided by the manufacturer.

### Microbiome Analysis

Metagenomic DNAs were extracted from the fecal samples using FastDNA Spin kit (116540600-CF, MP Biomedicals, USA). Using the barcoded universal primers, the V3-V4 region of bacterial 16S rRNA was amplified by PCR [26]. The amplicons were purified using CleanPCR (CPCR-0500, CleanNA, Netherlands) and analyzed using a Bioanalyzer 2100 (Agilent, USA) to assess the quality and product size. The pooled barcoded amplicons were sequenced using a MiSeq sequencer on the Ilumina platform (CJ Bioscience, Inc., Korea). The EzBioCloud database was used for taxonomic profiling of the microbiota [26]. Mann-Whitney U-test (SPSS IBM, USA) statistical analysis was used to examine the variation in taxonomic profiles among the samples. The in-house programs of CJ Bioscience, Inc. were utilized to acquire Shannon, Jackknife, and Simpson diversity. A heatmap of bacterial genera was generated using Gitools (http://www.gitools.org) and MultiExperiment Viewer (MeV; http://mev.tm4.org/).

## Results

### Heme-SCP Diet Slowed Weight Gain and Decreased Subcutaneous Fat in HFD-Fed Mice

After eight weeks on HFD, male C57BL/6N mice were switched to various doses (0, 0.05 and 0.5%) of heme-SCP supplemented HFD (Fig. 1A). During the administration period for 28 days (1^st^ set of 28 days heme-SCP), average body weights gradually increased, showing comparable levels among three groups on day 28: 48.344_±1.175_(0%), 48.046_±0.529_ (0.05%), and 46.845_±1.949_ g (0.5%). However, the rate of weight growth was slower in the 0.05 and 0.5% heme-SCP groups than that in the control group (0% heme-SCP) at the beginning of heme-SCP administration (Fig. 1B). Accordantly, the hemoprotein-fed groups (0.05% and 0.5% heme-SCP) maintained 1–5% lower average body weight compared with that of the control group (0% heme-SCP) on day 14, although no statistically significant differences were observed between the groups: 46.772_±1.099_ (0%), 43.771_±0.299_ (0.05%), and 44.324_±2.146_g (0.5%). However, the body types of the heme-SCP-fed mice could be distinguished from those of the control animals. The heme-SCP-fed mice had stiffer, more angulated, and oilier bodies compared with those of the control mice, which had flabby and roundish bodies and dull coats (Fig. 1C). During the administration tests, no signs of health issues, such as hair loss, diarrhea, polyuria, altered activity, and edema, were observed in all mouse groups.

The heme-SCP diet seemed to restrain weight gain till day 14, but the mice gained weight again later (Fig. 1A). To define the causes of the changes in body shape, the heme-SCP-fed mice were dissected at day 10 (2^nd^ set of 10 days heme-SCP), before a drastic weight gain (Fig. 2A). As observed in the 1^st^ set of data, the average body weights of the hemoprotein-fed groups were 1 to 3% lower than those of the control group on day 10: 37.840_±2.448_ (0%), 36.469_±2.943_ (0.05%), and 37.401_±2.862_ g (0.5%), with no statistical significance (Fig. S1A). The body shape was also altered by the heme-SCP diet but the differences in appearance were not as obvious as they were in the 1^st^ set (Fig. S1B). Further, the weights of the dissected tissues, including subcutaneous fat and thigh muscle, were measured and the tissue/body weight ratios were compared (Fig. 2B). The weights of visceral adipose tissues (VATs), including those of epididymal, perirenal, and mesenteric fats, tended to decline with heme-SCP administration, while no significant differences were observed with different hemoprotein doses. However, the weights of subcutaneous fat were significantly reduced by the heme-SCP diet: 2.373_±0.255_ (0%), 1.313_±0.137_ (0.05%), and 1.596_±0.174_ (0.5%) g. VAT, present in the abdominal cavity, is known to have a stronger potential to produce fatty acids and induce glucose uptake [27] compared with that of subcutaneous adipose tissue (SAT). Meanwhile, SAT readily absorbs free fatty acids and triglycerides in the bloodstream. On the other hand, heme-SCP increased the weights of thigh muscle in a dose-dependent manner: 0.838_±0.035_ (0%), 0.900_±0.092_ (0.05%), and 1.065_±0.033_ (0.5%) g.

### Serum Lipid Profiles Were Transiently Improved in Mice Fed Heme-SCP at Day 10

**Fig. 3:**
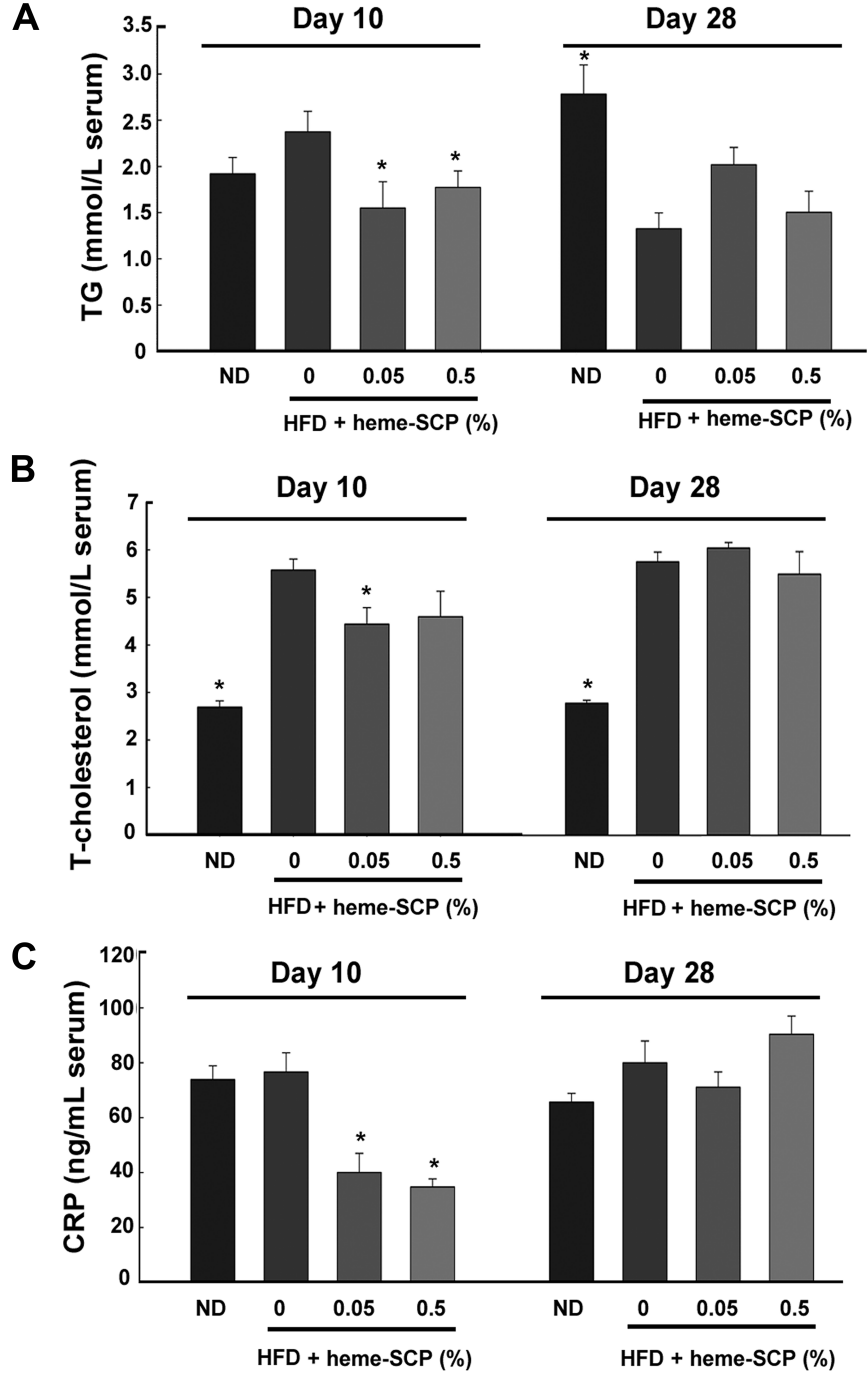
Serum profiles of mice fed heme-SCP. Serum metabolite profiles of high-fat diet (HFD)-fed mice were determined on days 10 and 28 after heme-SCP administration. The levels of triglyceride (A), total cholesterol (B), and Creactive protein (C) were averaged for each group of five mice. ND represents a control group fed a normal diet. Three HFD-fed groups were supplemented with 0, 0.05, or 0.5% heme-SCP. The *p*-value was calculated in comparison with mice fed HFD containing 0% heme-SCP, and *p*-values < 0.05 indicated significant differences that were represented with asterisks.

Despite the unnoticeable variations in body weight and shape, heme-SCP treatment decreased the weights of fat tissues, particularly subcutaneous fat. The altered fat disposition following heme-SCP administration encouraged us to evaluate the serum markers related with dyslipidemia. Compared with the group on the normal diet at day 10, the groups on the HFD without heme-SCP exhibited increased concentrations of serum triglyceride (TG) and total cholesterol (T-cholesterol). However, heme-SCP diet lowered the levels of TG and T-cholesterol on day 10 (Figs. 3A and 3B): 2.37_±0.49_ (0%), 1.55_±0.64_ (0.05%), and 1.77_±0.4_ (0.5%) mmol/L for TG; 5.58_±0.52_ (0%), 4.44_±0.77_ (0.05%), and 4.59_±1.2_ (0.5%) mmol/L for T-cholesterol. Notably, heme-SCP had no influence on the levels of TG or T-cholesterol by day 28. CRP, which is generated by liver cells in response to an increase in IL-6 production by macrophages and adipocytes, is a sensitive marker for systemic inflammation and is implicated in the etiology of numerous chronic disorders, such as obesity and coronary heart disease [28]. On day 10, mice fed heme-SCP exhibited a significant decrease in CRP levels in the blood serum; however, by day 28, no difference in CRP levels was observed between the mouse groups. (Fig. 3C).

### Heme-SCP Promoted the Proliferation of Gut Bacteria with Anti-Obesity Effects

**Fig. 4:**
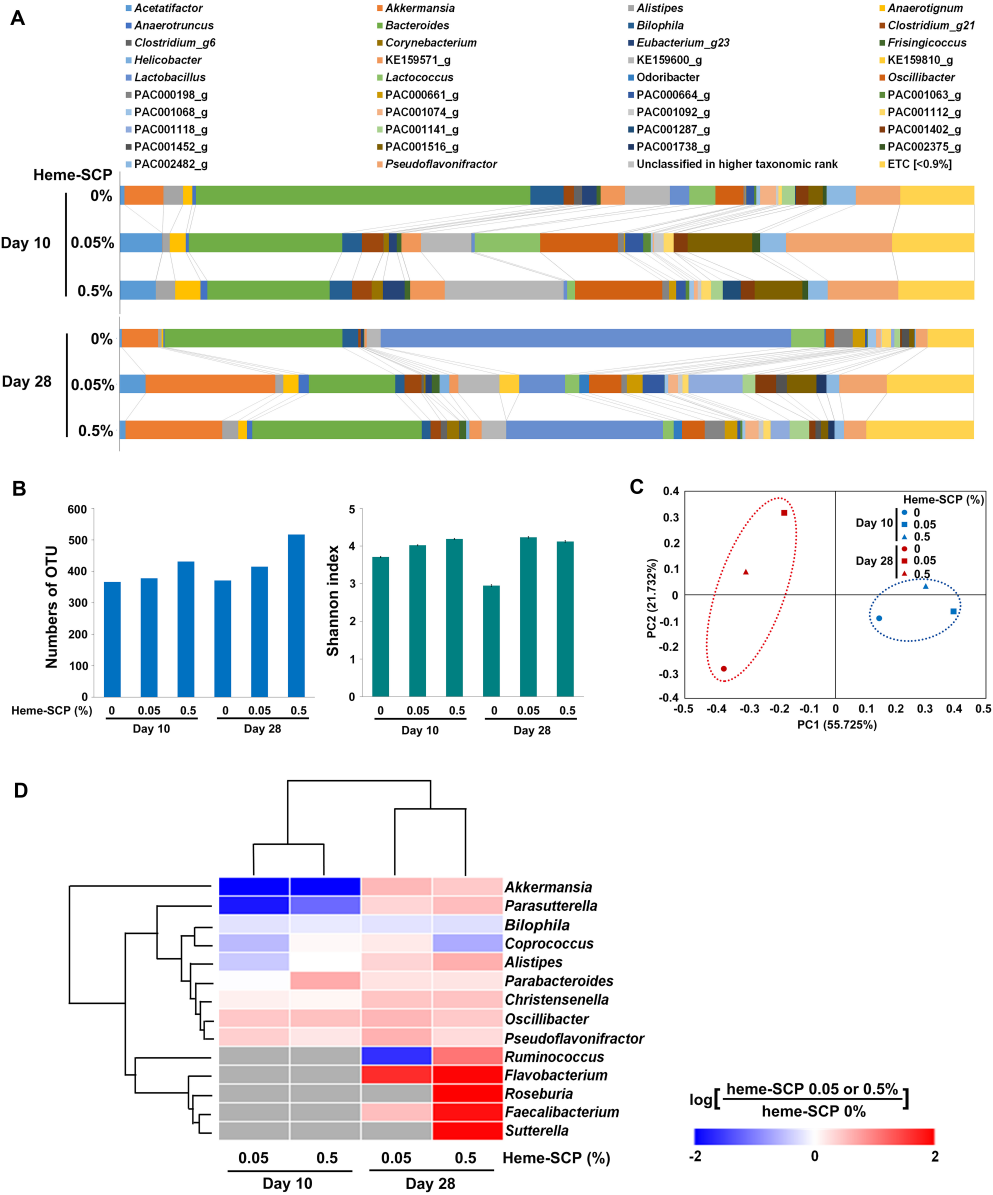
Alteration of the gut microbiota by heme-SCP. The gut microbiota of mice fed with heme-SCP (0, 0.05, and 0.5%) was analyzed. The feces from five mice of each group were combined for 16S rRNA sequencing. (**A**) Relative abundance plots of top 40 genera were displayed using EzBioClould database. (**B**) Alpha diversity was predicted using the numbers of observed operational taxonomic unit (OTU) (left) and Shannon indices (right). (**C**) Beta diversity comparison of fecal bacterial communities obtained from heme-SCP-fed mice between 10 and 28 days. The two-dimensional principal coordinate analysis (PCoA) based on Bray-Curtis dissimilarity metrics is displayed. (**D**) Heatmap of anti-obesity biomarkers was generated using the numbers of16S rRNA amplicons. Differences of relative abundance in bacterial genera between heme-SCP (0.05 or 0.5%) diet and no treatment is indicated in blue (decrease) or red (increase). Gray indicates ‘not determined’.

Metagenomic analysis of the fecal samples collected from each group of mice was performed (supporting information Table S1). Despite the different microbiota architectures of HFD-fed mice between days 10 and 28, the heme-SCP diet changed the composition of the gut microbiota in both mice groups (Fig. 4A). The total operational taxonomic unit (OTU) numbers and Shannon indices increased after heme-SCP diet, indicating a rise in the richness and diversity of the bacterial population (Fig. 4B). The beta diversity analysis revealed that the bacterial composition profiles varied between day 10 and day 28 as expected (Fig. 4C). Accumulating data suggest that changes in gut microbiota are responsible for numerous metabolic diseases, including obesity. The ratios of 14 bacterial genera known to prevent obesity were evaluated between mouse groups to obtain insight into the relationship between heme-SCP and obesity in the context of gut microbiota (Fig. 4D). On day 28 following the heme-SCP diet, the proportions of all genera with anti-obesity properties increased, except those of *Bilophila* and *Coprococcus*, and these increases were dependent on the heme-SCP concentrations. *Ruminococcus*, *Flavobacterium*, *Roseburia*, *Faecalibacterium*, and *Sutterella*, in particular, which were scarcely identified on day 10, showed considerable increases on day 28. Three genera *Akkermansia*, *Parasutterella*, and *Alistipes* responded to heme-SCP in a contradicting manner between day 10 (decreased) and day 28 (increased). On the other hand, four genera *Parabacteroides*, *Christensenella*, *Oscillibacter*, and *Pseudoflavonifractor* flourished even on day 10 of heme-SCP diet. By contrast, two genera *Bilophila* and *Coprococcus* exhibited a marginal reduction following heme-SCP administration. It is unclear whether the heme-SCP altered the gut microbiota, which in turn disturbed lipid metabolism, or the heme-SCP perturbed lipid metabolism, which subsequently caused the altered microbiota. However, this outcome demonstrated that the heme-SCP diet was capable of promoting the beneficial bacteria to outcompete in the gut of obese mice.

## Discussion

The gut microbiota serves as a key regulator between dietary nutrients and host metabolism. The gut microbiota converts dietary nutrients into metabolites. Further, food influences the composition of the gut microbiota, thereby affecting its metabolic potential and host health. Consequently, microbial imbalance is likely to result in metabolic disturbances. Obesity is associated with lipid metabolism dysregulation, which can lead to aberrant blood cholesterol levels and ectopic lipid accumulation. This could cause metabolic disorders such as dyslipidemia, non-alcoholic steatohepatitis, type II diabetes, and atherosclerosis [1, 2, 29]. Further, dietary lipids can have an impact on the physiology of the host through interactions with the gut microbiota. In both mice and humans, the gut microbiota is involved in the biosynthesis and degradation of lipids such as triglycerides, cholesterol, and fatty acids and affects lipid levels in blood and tissues [30, 31]. For example, genera such as *Alistipes*, *Lactobacillus*, and *Prevotella* produce saturated fatty acids in the gut, while genera such as *Akkermansia*, *Enterococcus*, and *Lactobacillus* utilize the luminal fatty acids [31, 32]. A variety of bacterial metabolites such as short-chain fatty acids (SCFA), secondary bile acids, and TMA and bacterial structural determinants such as lipopolysaccharides (LPS) inducing inflammatory response modulate lipid metabolism [30, 33]. SCFA produced by the gut microbiota can be utilized up to 75% for energy metabolism in the colonic epithelium [34, 35]. Hence, the rate of SCFA metabolism can determine whether the host’s energy balance shifts toward weight gain or weight loss. Besides, SCFA are involved in diverse metabolic pathways. Acetate, for instance, is a precursor for lipogenesis, including that of fatty acids and cholesterol, in the liver [34]. Conversely, propionate decreases food intake and cholesterol production while serving as a substrate for gluconeogenesis [36]. Additionally, butyrate alleviates insulin tolerance in mice and exerts anti-inflammatory and anticancer effects that are associated with obesity in humans [34, 35]. Obesity is characterized by a persistent, low-grade inflammation that is fueled by adipocyte-released proinflammatory mediators such TNF-α, IL-1, and IL-6. Excess bacterial LPS can induce endotoxemia and proinflammatory processes that are frequently associated with obesity and other metabolic disorders [35, 37].

This study explored the possibility that the hemoprotein-enriched diet (heme-SCP) could reshape the gut microbiota in HFD-fed mice. After heme-SCP administration, mice gained less body weight and had less fat tissues, particularly subcutaneous fat, while their thigh muscle content increased. Further, the TG and T-cholesterol levels in mice serum were reduced by the heme-SCP diet on day 10, which implies the pathophysiological changes in lipid metabolism. Interestingly, despite the distinct changes in body shape, the alleviated TG and T-cholesterol levels by heme-SCP at day 10 seemed to be restored at day 28. The discrepant blood parameters at day 28 might imply transient oscillation and new adaptation of microbiota and host body after the lowered lipids in the serum. Many studies using animal models have indicated that luminal iron deficiency drastically changed the gut microbiota composition [38, 39]. Our previous observations demonstrated that heme-SCP administration altered the structure of gut microbiota in pet dogs [24, 25] and broiler chickens (Supporting information Data 1). For all lifeforms, including bacteria, iron is a crucial nutrient. However, since excess free iron is harmful, practically all the iron is sequestered in iron-containing substances like heme molecules in the mammalian host. Therefore, enteric bacteria have developed various highly sophisticated systems for acquiring iron from host heme compounds. Gram-negative pathogens, in particular, harness one or more TonB-dependent outer membrane receptors to transport heme compounds across the outer membrane and into the periplasm [40]. According to the heme paradox, heme molecules are toxic at high concentrations yet necessary at low concentrations, thus varying the physiological needs for heme molecules in bacterial species.

We observed that the proportions of twelve bacterial genera, proposed as anti-obesity biomarkers, were increased in mice fed HFD containing heme-SCP. Of these twelve genera, *Alistipes*, *Faecalibacterium*, *Parabacteroides*, *Parasutterella*, *Roseburia*, and *Ruminococcus* are speculated as negative indicators of multiple metabolism dysregulation such as overweight, abnormal blood pressure, and increase in uric acid, serum lipid (TG, T-cholesterol, and LDL-c), and blood glucose levels [41]. Genera *Flavobacterium* and *Sutterella* were predominant in normal-weight populations but drastically decreased in overweight/obese patients [42]. *Akkermensia*, *Alistipes*, and *Pseudoflavonifractor* were enriched in obesity patients undergoing successful weight reduction [43-45]; *Christensenella* and *Oscillibacter* were inversely correlated with body mass index and obesity [15, 46]. The mechanism by which these bacterial taxa benefit from heme-SCP for their growth is unknown. However, some species of genera *Alistipes*, *Flavobacterium*, and *Parabacteroides* have elaborate mechanisms to scavenge and detoxify iron and heme compounds, such as siderophores, heme-binding proteins, membranous iron receptors, and ferritin-like iron-storage proteins [47-49]. In particular, *Roseburia*, which thrives in the presence of iron, increases the production of butyrate, a main anti-inflammatory metabolite, in environments with high iron levels [50].

Whether the heme-SCP affected lipid metabolism, which in turn resulted in the altered microbiota, or whether the heme-SCP affected the gut microbiota, which in turn led to the change in lipid metabolism, is still uncertain based on the results of this study. Increases in the anti-obesity-causing bacterial populations after heme-SCP diet could have accelerated the decomposition of fat in the gut. Although gut anaerobes cannot oxidize lipids to make energy, bacteria can still consume fatty acids in other ways and exert their metabolic potential which affects human health. For example, *Akkermansia* metabolizes saturated fatty acids such as palmitic acid and is negatively associated with the level of total free fatty acids (FFA) in the serum [32, 51]. FFA released by lipolysis play diverse roles associated with biological process. Besides energy supply, FFA modulate the production of TLR4, NF-κB, and cytokines, as a mediator linking nutritional signaling to immune responses [51]. *Akkermansia* also enhances lipid oxidation and bile acid metabolism by facilitating L-aspartate transportation to liver from the gut, which substantially promotes reshaping of the gut microbiota and energy expenditure, reducing lipid accumulation in the tissues [52]. Mice fed heme-SCP also exhibited an increase in the amount of thigh muscle tissue. However, the underlying mechanism is unknown. Modified microbiota composition possibly triggers metabolic signals that accelerate muscle building. Alternatively, myoglobin is directly synthesized from heme molecules obtained from a heme-SCP diet.

Many studies have warned that administering hemin (purified heme salt) can increase cellular toxicity, aggravate the glucose and insulin tolerance, raise the risk of type II diabetes, and even increase the likelihood of developing cancer [53-55]. In the context of heme conundrum, we also noticed that the beneficial outcomes of heme-SCP were unlikely to have a positive correlation with the dose concentrations. Sometimes, a lower dose of heme-SCP resulted in a more favorable alteration of the serum metabolite profiles. The difference between the administration forms of free heme and protein-bound heme might have determined the nutritional outcomes. Reactive oxygen species (ROS) are unavoidably produced when an electron-attaching or -detaching molecule, such as heme, transfers an electron to oxygen. Conversely, hemoproteins would also generate ROS, but the mistransferred electron can be absorbed by the accompanying protein, reducing the oxidative load on other functioning nucleotides and proteins. Besides, the utilization efficacy of free heme or protein-bound heme can be influenced by the administration routes, such as intraperitoneal injection and food supplement, and the target organ or tissues. Heme can selectively bind to regulatory proteins such as transcriptional factors and kinases, thereby modulating their associated biological functions [56]. Ju *et al*. observed that heme lowered TG levels and increased the glucose uptake in the skeletal muscle of HFD-fed mice, which consequently improved the glucose and insulin tolerance, but failed to observe the beneficial effects of heme in the liver [57]. We observed that heme-SCP diet lowered the serum levels of TG and T-cholesterol on day 10. However, the serum glucose concentrations were slightly elevated by heme-SCP treatments, while the serum insulin concentrations barely changed (data not shown). Heme-SCP is a single-cell biomass enriched with protein-bound heme. Thus, the metabolic effect can be different from that of the purified form of heme. Furthermore, the metabolic circuits utilizing iron can be different between organs or tissues.

This study revealed the potential modulatory effect of heme-SCP on lipid metabolism in HFD-fed mice. The obese mice fed heme-SCP exhibited a reduction in body fat and an increase in muscle mass, which was in accordance with the lowered serum parameters, including TG, T-cholesterol, and CRP levels. Gut microbiota analysis suggests the possibility that heme-SCP shows a modulatory effect on lipid metabolism through reshaping of gut microbial composition. Further detailed studies (such as host metabolism by hormones [resistin, leptin, and adiponectin]; host immune response by biomarkers [TNF-α, IL-1, and IL-6]; metabolite profiles from the intestinal lumen; gene expression patterns of the anti-obesity microbes and host tissues; histology of intestine and liver; comparisons of herbivorous and carnivorous animals with omnivorous animals in the heme-SCP administrations) are required to understand the precise mechanisms underlying the prebiotic effect of hemoproteins.

## Supplemental Materials

Supplementary data for this paper are available on-line only at http://jmb.or.kr.



## References

[1] Alpert MA, Hashimi MW (1993). Obesity and the heart. Am. J. Med. Sci..

[2] Rimm AA, Werner LH, Yserloo BV, Bernstein RA (1975). Relationship of ovesity and disease in 73,532 weight-conscious women. Public Health Rep..

[3] Calle EE, Thun MJ (2004). Obesity and cancer. Oncogene.

[4] Kopelman PG (2000). Obesity as a medical problem. Nature.

[5] Liu J, Ding H, Yan C, He Z, Zhu H, Ma KY (2023). Effect of tea catechins on gut microbiota in high fat diet-induced obese mice. J. Sci. Food Agric..

[6] Chen H, Zhao H, Qi X, Sun Y, Ma Y, Li Q (2023). *Lactobacillus plantarum* HF02 alleviates lipid accumulation and intestinal microbiota dysbiosis in high-fat diet-induced obese mice. J. Sci. Food Agric..

[7] Caesar R, Tremaroli V, Kovatcheva-Datchary P, Cani PD, Backhed F (2015). Crosstalk between gut microbiota and dietary lipids aggravates WAT inflammation through TLR signaling. Cell Metab..

[8] Lam YY, Ha CW, Hoffmann JM, Oscarsson J, Dinudom A, Mather TJ (2015). Effects of dietary fat profile on gut permeability and microbiota and their relationships with metabolic changes in mice. Obesity (Silver Spring).

[9] Just S, Mondot S, Ecker J, Wegner K, Rath E, Gau L (2018). The gut microbiota drives the impact of bile acids and fat source in diet on mouse metabolism. Microbiome.

[10] Shreiner AB, Kao JY, Young VB (2015). The gut microbiome in health and in disease. Curr. Opin. Gastroenterol..

[11] Yoo W, Zieba JK, Foegeding NJ, Torres TP, Shelton CD, Shealy NG (2021). High-fat diet-induced colonocyte dysfunction escalates microbiota-derived trimethylamine N-oxide. Science.

[12] Wang B, Qiu J, Lian J, Yang X, Zhou J (2021). Gut metabolite trimethylamine-N-oxide in atherosclerosis: From mechanism to therapy. Front. Cardiovasc. Med..

[13] Ley RE, Backhed F, Turnbaugh P, Lozupone CA, Knight RD, Gordon JI (2005). Obesity alters gut microbial ecology. Proc. Natl. Acad. Sci. USA.

[14] Turnbaugh PJ, Gordon JI (2009). The core gut microbiome, energy balance and obesity. J. Physiol..

[15] Hu HJ, Park SG, Jang HB, Choi MK, Park KH, Kang JH (2015). Obesity Alters the Microbial Community Profile in Korean Adolescents. PLoS One.

[16] Backhed F, Ding H, Wang T, Hooper LV, Koh GY, Nagy A (2004). The gut microbiota as an environmental factor that regulates fat storage. Proc. Natl. Acad. Sci. USA.

[17] Murphy EF, Cotter PD, Healy S, Marques TM, O'Sullivan O, Fouhy F (2010). Composition and energy harvesting capacity of the gut microbiota: relationship to diet, obesity and time in mouse models. Gut.

[18] Belzer C (2022). Nutritional strategies for mucosal health: the interplay between microbes and mucin glycans. Trends Microbiol..

[19] Brooijmans R, Smit B, Santos F, van Riel J, de Vos WM, Hugenholtz J (2009). Heme and menaquinone induced electron transport in lactic acid bacteria. Microb. Cell Fact..

[20] Lechardeur D, Cesselin B, Fernandez A, Lamberet G, Garrigues C, Pedersen M (2011). Using heme as an energy boost for lactic acid bacteria. Curr. Opin. Biotechnol..

[21] Baureder M, Hederstedt L (2013). Heme proteins in lactic acid bacteria. Adv. Microb. Physiol..

[22] Botta A, Barra NG, Lam NH, Chow S, Pantopoulos K, Schertzer JD (2021). Iron reshapes the gut microbiome and host metabolism. J. Lipid Atheroscler..

[23] Abbaspour N, Hurrell R, Kelishadi R (2014). Review on iron and its importance for human health. J. Res. Med. Sci..

[24] Lee S, Kim P (2021). Effect of heme-rich nutrient on anaerobic bacterial growth and survival: a model study on *Lactobacillus gasseri*. Microbiol. Biotechnol. Lett..

[25] Lee S, Choi A, Park K-H, Lee S, Yoon H, Kim P (2022). Single-cell hemoprotein (heme-SCP) exerts the prebiotic potential to establish a healthy gut microbiota in small pet dogs. Food Sci. Biotechnol..

[26] Yoon SH, Ha SM, Kwon S, Lim J, Kim Y, Seo H (2017). Introducing EzBioCloud: a taxonomically united database of 16S rRNA gene sequences and whole-genome assemblies. Int. J. Syst. Evol. Microbiol..

[27] Ibrahim MM (2010). Subcutaneous and visceral adipose tissue: structural and functional differences. Obes. Rev..

[28] Visser M, Bouter LM, McQuillan GM, Wener MH, Harris TB (1999). Elevated C-reactive protein levels in overweight and obese adults. JAMA.

[29] Grundy SM (2016). Metabolic syndrome update. Trends Cardiovasc. Med..

[30] Schoeler M, Caesar R (2019). Dietary lipids, gut microbiota and lipid metabolism. Rev. Endocr. Metab. Disord..

[31] Zhao L, Huang Y, Lu L, Yang W, Huang T, Lin Z (2018). Saturated long-chain fatty acid-producing bacteria contribute to enhanced colonic motility in rats. Microbiome.

[32] Chen P, Torralba M, Tan J, Embree M, Zengler K, Starkel P (2015). Supplementation of saturated long-chain fatty acids maintains intestinal eubiosis and reduces ethanol-induced liver injury in mice. Gastroenterology.

[33] Devkota S, Wang Y, Musch MW, Leone V, Fehlner-Peach H, Nadimpalli A (2012). Dietary-fat-induced taurocholic acid promotes pathobiont expansion and colitis in Il10-/- mice. Nature.

[34] Chakraborti CK (2015). New-found link between microbiota and obesity. World J. Gastrointest. Pathophysiol..

[35] Amabebe E, Robert FO, Agbalalah T, Orubu ESF (2020). Microbial dysbiosis-induced obesity: role of gut microbiota in homoeostasis of energy metabolism. Br. J. Nutr..

[36] Davis CD (2016). The gut microbiome and its role in obesity. Nutr. Today.

[37] Carvalho BM, Saad MJ (2013). Influence of gut microbiota on subclinical inflammation and insulin resistance. Mediators Inflamm..

[38] Dostal A, Chassard C, Hilty FM, Zimmermann MB, Jaeggi T, Rossi S (2012). Iron depletion and repletion with ferrous sulfate or electrolytic iron modifies the composition and metabolic activity of the gut microbiota in rats. J. Nutr..

[39] Pereira DI, Aslam MF, Frazer DM, Schmidt A, Walton GE, McCartney AL (2015). Dietary iron depletion at weaning imprints low microbiome diversity and this is not recovered with oral Nano Fe(III). Microbiologyopen.

[40] Anzaldi LL, Skaar EP (2010). Overcoming the heme paradox: heme toxicity and tolerance in bacterial pathogens. Infect. Immun..

[41] Zeng Q, Li D, He Y, Li Y, Yang Z, Zhao X (2019). Discrepant gut microbiota markers for the classification of obesity-related metabolic abnormalities. Sci. Rep..

[42] Palmas V, Pisanu S, Madau V, Casula E, Deledda A, Cusano R (2021). Gut microbiota markers associated with obesity and overweight in Italian adults. Sci. Rep..

[43] Everard A, Belzer C, Geurts L, Ouwerkerk JP, Druart C, Bindels LB (2013). Cross-talk between Akkermansia muciniphila and intestinal epithelium controls diet-induced obesity. Proc. Natl. Acad. Sci. USA.

[44] Louis S, Tappu RM, Damms-Machado A, Huson DH, Bischoff SC (2016). Characterization of the gut microbial community of obese patients following a weight-loss intervention using whole metagenome shotgun sequencing. PLoS One.

[45] Bischoff SC, Nguyen NK, Seethaler B, Beisner J, Kugler P, Stefan T (2022). Gut microbiota patterns predicting long-term weight loss success in individuals with obesity undergoing nonsurgical therapy. Nutrients..

[46] Waters JL, Ley RE (2019). The human gut bacteria Christensenellaceae are widespread, heritable, and associated with health. BMC Biol..

[47] Moschen AR, Gerner RR, Wang J, Klepsch V, Adolph TE, Reider SJ (2016). Lipocalin 2 protects from inflammation and tumorigenesis associated with gut microbiota alterations. Cell Host Microbe..

[48] Conrad RA, Evenhuis JP, Lipscomb RS, Perez-Pascual D, Stevick RJ, Birkett C (2022). Flavobacterium columnare ferric iron uptake systems are required for virulence. Front. Cell Infect. Microbiol..

[49] Rocha ER, Smith CJ (2013). Ferritin-like family proteins in the anaerobe *Bacteroides fragilis*: when an oxygen storm is coming, take your iron to the shelter. Biometals.

[50] Dostal A, Lacroix C, Bircher L, Pham VT, Follador R, Zimmermann MB (2015). Iron modulates butyrate production by a child gut microbiota in vitro. mBio.

[51] Rodriguez-Carrio J, Salazar N, Margolles A, Gonzalez S, Gueimonde M, de Los Reyes-Gavilan CG (2017). Free fatty acids profiles are related to gut microbiota signatures and short-chain fatty acids. Front. Immunol..

[52] Rao Y, Kuang Z, Li C, Guo S, Xu Y, Zhao D (2021). Gut *Akkermansia muciniphila* ameliorates metabolic dysfunction-associated fatty liver disease by regulating the metabolism of L-aspartate via gut-liver axis. Gut Microbes.

[53] Constante M, Fragoso G, Calve A, Samba-Mondonga M, Santos MM (2017). Dietary heme induces gut dysbiosis, aggravates colitis, and potentiates the development of adenomas in mice. Front. Microbiol..

[54] White DL, Collinson A (2013). Red meat, dietary heme iron, and risk of type 2 diabetes: the involvement of advanced lipoxidation endproducts. Adv. Nutr..

[55] N IJ, Derrien M, van Doorn GM, Rijnierse A, van den Bogert B, Muller M (2012). Dietary heme alters microbiota and mucosa of mouse colon without functional changes in host-microbe cross-talk. PLoS One.

[56] Schaer DJ, Buehler PW, Alayash AI, Belcher JD, Vercellotti GM (2013). Hemolysis and free hemoglobin revisited: exploring hemoglobin and hemin scavengers as a novel class of therapeutic proteins. Blood.

[57] Ju TJ, Kwon WY, Kim YW, Kim JY, Kim YD, Lee IK (2014). Hemin improves insulin sensitivity in skeletal muscle in high fat-fed mice. J. Pharmacol. Sci..

