# Supplementary material for: Single-Cell Hemoprotein Diet Changes Adipose Tissue Distributions and Re-Shapes Gut Microbiota in High-Fat Diet-Induced Obese Mice
Source: J Microbiol Biotechnol. 2023 Sep 22;33(12):1648–56. doi: 10.4014/jmb.2308.08046 (PMC10772551; doi:10.4014/jmb.2308.08046)
Supplement: Supplementary file 1 [file jmb-33-12-1648-supple.pdf]

J. Microbiol. Biotechnol.
https://doi.org/10.4014/jmb.2308.08046

# Supporting information Data 1. Heme-SCP study on poultry

## *Feeds*

Four test feeds were prepared. BD (basic diet, Central Livestock Feed Inc., Korea) were used as control. A ferrous chelate of glycine (B-TRAXIM Fe, Pancosma Inc., Voie-des-Traz, Switzerland) or single-cell hemoprotein (heme-SCP; Hemolab Inc., Korea) were mixed with BD to formulate feeds of BD + 50 ppm ferrous chelate of glycine; BD + 1 ppm heme-SCP; and BD + 10 ppm heme-SCP.

## *Fowl Study*

One hundred sixty broiler chickens (ROSS 308 strain, 1-day-old) were divided into 4 coops with 40 chickens each at a farm facility (Celltech Inc., Korea) and bred with a test feed for 32 days. The weight of the fowls and the amount of feed consumed were recorded every day. Caecum was separated, ground, diluted with distilled water, and spread on MRS agar plates to enumerate lactic acid bacteria (LAB). The plate was incubated for three days.

## *Characterization of chickens fed heme-SCP*

Broiler chickens fed a food supplemented with heme-SCP gained less weight than those on a normal diet (BD) or a negative control diet (BD + ferrous chelate glycine); however, the groups of the heme-SCP supplemented diet had higher levels of LAB in their caecums.

| [a]Feed Group | Weight gain (g) | Feed intake (g) | Feed conversion rate | Caecum LAB ($10^8$ CFU/g) |
|---|---|---|---|---|
| BD | 1579.3 ± 21.4 | 2247.0 ± 106.2 | 1.42 ± 0.08 | 3.7 |
| BD + 50 ppm Fe-glycine | 1582.6 ± 44.1 | 2090.3 ± 32.7 | 1.32 ± 0.05 | 2.1 |
| BD + 1 ppm heme-SCP | 1533.9 ± 31.0 | 2040.9 ± 57.7 | 1.33 ± 0.06 | 6.9 |
| BD + 10 ppm heme-SCP | 1559.3 ± 43.6 | 2088.1 ± 41.1 | 1.34 ± 0.04 | 8.8 |

[a]n = 40 for each group; Data are represented as mean ± SD.

※ This test's organizer, CellTech Inc., shared the findings with the product's creator, Hemolab l. c., to let them know that its heme-SCP was ineffective at promoting weight gain in broiler chickens.

**Supporting information Figure S1. Body weight and shape of obese mouse administered heme-SCP for 10 days.**

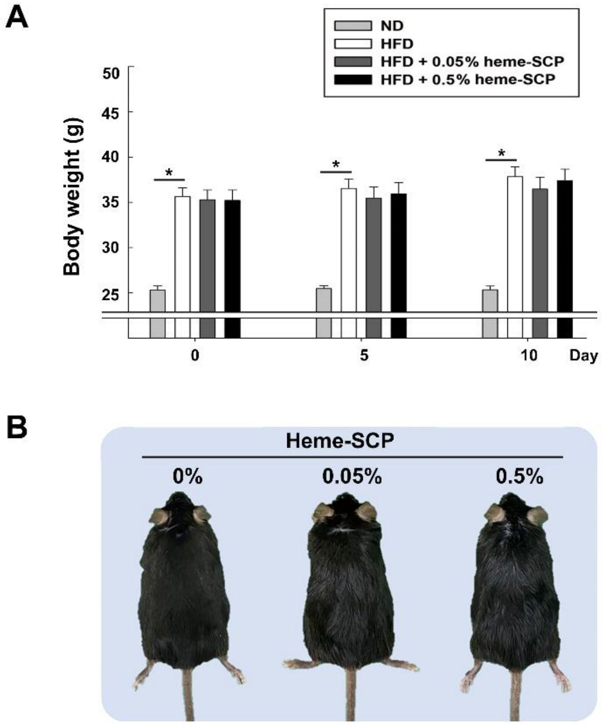


**Fig. S1. Body weight and shape of obese mouse administered heme-SCP for 10 days.** High fat diet mixed with 0, 0.05, and 0.5% heme-SCP was administered to mice for 10 days. ND indicates normal diet. Body weights **(A)** and body shapes on day 10 **(B)** were compared. Values are expressed as mean ± SEM for each group of five mice and the representative images are presented. Asterisks denote significant differences: $p$ value < 0.05.

## Gut microbiota analysis on heme-SCP fed mice

| Classification | | Description |
|---|---|---|
| Data set | Set 1 | **Day 28** |
| | Set 2 | **Day 10** |
| Heme-SCP concentration | 0% | **HFD + 0% heme-SCP** |
| | 0.05% | **HFD + 0.05% heme-SCP** |
| | 0.50% | **HFD + 0.5% heme-SCP** |
| Taxonomy | P | **Phylum** |
| | G | **Genus** |
| | S | **Species** |

**Set 2_0%_P: Day 10_heme-SCP 0%_Phylum**

| Taxon name | Taxonomy | Count | Proportion(%) |
|---|---|---|---|
| Bacteroidetes | Bacteria;Bacteroidetes | 17168 | 48.6718 |
| Firmicutes | Bacteria;Firmicutes | 14784 | 41.9131 |
| Verrucomicrobia | Bacteria;Verrucomicrobia | 1609 | 4.5616 |
| Proteobacteria | Bacteria;Proteobacteria | 1584 | 4.4907 |
| Actinobacteria | Bacteria;Actinobacteria | 75 | 0.2126 |
| Tenericutes | Bacteria;Tenericutes | 29 | 0.0822 |
| Deferribacteres | Bacteria;Deferribacteres | 24 | 0.068 |

**Set 2_0.05%_P: Day 10_heme-SCP 0.05%_Phylum**

| Taxon name | Taxonomy | Count | Proportion(%) |
|---|---|---|---|
| Firmicutes | Bacteria;Firmicutes | 31748 | 71.2845 |
| Bacteroidetes | Bacteria;Bacteroidetes | 11261 | 25.2846 |
| Proteobacteria | Bacteria;Proteobacteria | 1108 | 2.4878 |
| Actinobacteria | Bacteria;Actinobacteria | 315 | 0.7073 |
| Deferribacteres | Bacteria;Deferribacteres | 89 | 0.1998 |
| Tenericutes | Bacteria;Tenericutes | 10 | 0.0225 |
| Verrucomicrobia | Bacteria;Verrucomicrobia | 6 | 0.0135 |

**Set 2_0.5%_P: Day 10_heme-SCP 0.5%_Phylum**

| Taxon name | Taxonomy | Count | Proportion(%) |
|---|---|---|---|
| Firmicutes | Bacteria;Firmicutes | 28748 | 72.5117 |
| Bacteroidetes | Bacteria;Bacteroidetes | 9146 | 23.0692 |
| Proteobacteria | Bacteria;Proteobacteria | 1140 | 2.8754 |
| Actinobacteria | Bacteria;Actinobacteria | 528 | 1.3318 |
| Deferribacteres | Bacteria;Deferribacteres | 73 | 0.1841 |
| Tenericutes | Bacteria;Tenericutes | 11 | 0.0277 |

**Set 2_0%_G: Day 10_heme-SCP 0%_Genus**

| Taxon name | Taxonomy | Count | Proportion(%) |
|---|---|---|---|
| Bacteroides | Bacteria;Bacteroidetes;Bacteroidia;Bacteroidales;Bacteroidaceae;Bacteroides | 13808 | 39.1461 |
| KE159600_g | Bacteria;Firmicutes;Clostridia;Clostridiales;Lachnospiraceae;KE159600_g | 1853 | 5.2533 |
| Pseudoflavonifractor | Bacteria;Firmicutes;Clostridia;Clostridiales;Ruminococcaceae;Pseudoflavonifractor | 1836 | 5.2051 |
| Akkermansia | Bacteria;Verrucomicrobia;Verrucomicrobiae;Verrucomicrobiales;Akkermansiaceae;Akkermansia | 1609 | 4.5616 |
| Bilophila | Bacteria;Proteobacteria;Deltaproteobacteria;Desulfovibrionales;Desulfovibrionaceae;Bilophila | 1374 | 3.8953 |
| PAC002482_g | Bacteria;Bacteroidetes;Bacteroidia;Bacteroidales;AC160630_f;PAC002482_g | 1203 | 3.4105 |
| Oscillibacter | Bacteria;Firmicutes;Clostridia;Clostridiales;Ruminococcaceae;Oscillibacter | 1163 | 3.2971 |
| Lactococcus | Bacteria;Firmicutes;Bacilli;Lactobacillales;Streptococcaceae;Lactococcus | 1077 | 3.0533 |
| KE159571_g | Bacteria;Firmicutes;Clostridia;Clostridiales;Lachnospiraceae;KE159571_g | 1001 | 2.8379 |
| Lactobacillus | Bacteria;Firmicutes;Bacilli;Lactobacillales;Lactobacillaceae;Lactobacillus | 808 | 2.2907 |
| Alistipes | Bacteria;Bacteroidetes;Bacteroidia;Bacteroidales;Rikenellaceae;Alistipes | 805 | 2.2822 |
| PAC001074_g | Bacteria;Bacteroidetes;Bacteroidia;Bacteroidales;Muribaculaceae;PAC001074_g | 648 | 1.8371 |
| Eubacterium_g23 | Bacteria;Firmicutes;Clostridia;Clostridiales;Ruminococcaceae;Eubacterium_g23 | 603 | 1.7095 |
| PAC001516_g | Bacteria;Firmicutes;Clostridia;Clostridiales;Lachnospiraceae;PAC001516_g | 598 | 1.6953 |
| PAC001141_g | Bacteria;Firmicutes;Clostridia;Clostridiales;Christensenellaceae;PAC001141_g | 542 | 1.5366 |
| PAC001402_g | Bacteria;Firmicutes;Clostridia;Clostridiales;Ruminococcaceae;PAC001402_g | 511 | 1.4487 |
| Clostridium_g21 | Bacteria;Firmicutes;Clostridia;Clostridiales;Lachnospiraceae;Clostridium_g21 | 425 | 1.2049 |
| Anaerotignum | Bacteria;Firmicutes;Clostridia;Clostridiales;Lachnospiraceae;Anaerotignum | 383 | 1.0858 |
| Clostridium_g6 | Bacteria;Firmicutes;Erysipelotrichi;Erysipelotrichales;Erysipelotrichaceae;Clostridium_g6 | 337 | 0.9554 |
| PAC000664_g | Bacteria;Firmicutes;Clostridia;Clostridiales;Lachnospiraceae;PAC000664_g | 321 | 0.91 |
| KE159797_g | Bacteria;Firmicutes;Clostridia;Clostridiales;Lachnospiraceae;KE159797_g | 219 | 0.6209 |
| KE159605_g | Bacteria;Firmicutes;Clostridia;Clostridiales;Lachnospiraceae;KE159605_g | 206 | 0.584 |
| Acetatifactor | Bacteria;Firmicutes;Clostridia;Clostridiales;Lachnospiraceae;Acetatifactor | 190 | 0.5387 |
| PAC001165_g | Bacteria;Firmicutes;Clostridia;Clostridiales;Lachnospiraceae;PAC001165_g | 189 | 0.5358 |
| Frisingicoccus | Bacteria;Firmicutes;Clostridia;Clostridiales;Lachnospiraceae;Frisingicoccus | 163 | 0.4621 |
| PAC001068_g | Bacteria;Bacteroidetes;Bacteroidia;Bacteroidales;Muribaculaceae;PAC001068_g | 162 | 0.4593 |
| PAC002375_g | Bacteria;Firmicutes;Clostridia;Clostridiales;Lachnospiraceae;PAC002375_g | 157 | 0.4451 |
| PAC001201_g | Bacteria;Firmicutes;Clostridia;Clostridiales;Lachnospiraceae;PAC001201_g | 155 | 0.4394 |
| PAC001112_g | Bacteria;Bacteroidetes;Bacteroidia;Bacteroidales;Muribaculaceae;PAC001112_g | 151 | 0.4281 |
| Anaerotruncus | Bacteria;Firmicutes;Clostridia;Clostridiales;Ruminococcaceae;Anaerotruncus | 147 | 0.4167 |
| PAC001066_g | Bacteria;Bacteroidetes;Bacteroidia;Bacteroidales;Muribaculaceae;PAC001066_g | 135 | 0.3827 |
| KE159538_g | Bacteria;Firmicutes;Clostridia;Clostridiales;Lachnospiraceae;KE159538_g | 127 | 0.36 |
| Parasutterella | Bacteria;Proteobacteria;Betaproteobacteria;Burkholderiales;Sutterellaceae;Parasutterella | 107 | 0.3033 |
| PAC001221_g | Bacteria;Firmicutes;Clostridia;Clostridiales;Dehalobacterium_f;PAC001221_g | 104 | 0.2948 |
| PAC001092_g | Bacteria;Firmicutes;Clostridia;Clostridiales;Lachnospiraceae;PAC001092_g | 96 | 0.2722 |
| PAC001778_g | Bacteria;Firmicutes;Clostridia;Clostridiales;Ruminococcaceae;PAC001778_g | 95 | 0.2693 |
| PAC001063_g | Bacteria;Bacteroidetes;Bacteroidia;Bacteroidales;Muribaculaceae;PAC001063_g | 92 | 0.2608 |
| Sporobacter | Bacteria;Firmicutes;Clostridia;Clostridiales;Ruminococcaceae;Sporobacter | 91 | 0.258 |
| PAC000198_g | Bacteria;Bacteroidetes;Bacteroidia;Bacteroidales;Muribaculaceae;PAC000198_g | 81 | 0.2296 |
| PAC001400_g | Bacteria;Firmicutes;Clostridia;Clostridiales;Peptococcaceae;PAC001400_g | 73 | 0.207 |
| Enterorhabdus | Bacteria;Actinobacteria;Coriobacteriia;Coriobacteriales;Coriobacteriaceae;Enterorhabdus | 71 | 0.2013 |
| PAC001043_g | Bacteria;Firmicutes;Clostridia;Clostridiales;Lachnospiraceae;PAC001043_g | 68 | 0.1928 |
| PAC001544_g | Bacteria;Firmicutes;Clostridia;Clostridiales;Lachnospiraceae;PAC001544_g | 68 | 0.1928 |
| Paludicola | Bacteria;Firmicutes;Clostridia;Clostridiales;Ruminococcaceae;Paludicola | 66 | 0.1871 |
| Caproiciproducens | Bacteria;Firmicutes;Clostridia;Clostridiales;Ruminococcaceae;Caproiciproducens | 63 | 0.1786 |
| Lachnospiraceae_uc | Bacteria;Firmicutes;Clostridia;Clostridiales;Lachnospiraceae;Lachnospiraceae_uc | 58 | 0.1644 |
| Escherichia | Bacteria;Proteobacteria;Gammaproteobacteria;Enterobacterales;Enterobacteriaceae;Escherichia | 57 | 0.1616 |
| PAC000748_g | Bacteria;Firmicutes;Clostridia;Clostridiales;Ruminococcaceae;PAC000748_g | 56 | 0.1588 |
| PAC001103_g | Bacteria;Firmicutes;Clostridia;Clostridiales;Lachnospiraceae;PAC001103_g | 51 | 0.1446 |
| PAC001385_g | Bacteria;Firmicutes;Clostridia;Clostridiales;Lachnospiraceae;PAC001385_g | 50 | 0.1418 |
| Emergencia | Bacteria;Firmicutes;Clostridia;Clostridiales;Mogibacterium_f;Emergencia | 42 | 0.1191 |
| Desulfovibrio | Bacteria;Proteobacteria;Deltaproteobacteria;Desulfovibrionales;Desulfovibrionaceae;Desulfovibrio | 39 | 0.1106 |
| Eubacterium_g6 | Bacteria;Firmicutes;Clostridia;Clostridiales;Lachnospiraceae;Eubacterium_g6 | 39 | 0.1106 |
| PAC000186_g | Bacteria;Bacteroidetes;Bacteroidia;Bacteroidales;Muribaculaceae;PAC000186_g | 39 | 0.1106 |
| PAC001138_g | Bacteria;Firmicutes;Clostridia;Clostridiales;Lachnospiraceae;PAC001138_g | 39 | 0.1106 |
| Gemella | Bacteria;Firmicutes;Bacilli;Bacillales;Gemella_f;Gemella | 38 | 0.1077 |
| PAC001200_g | Bacteria;Firmicutes;Clostridia;Clostridiales;Lachnospiraceae;PAC001200_g | 35 | 0.0992 |
| Massilioclostridium | Bacteria;Firmicutes;Clostridia;Clostridiales;Ruminococcaceae;Massilioclostridium | 31 | 0.0879 |
| PAC001287_g | Bacteria;Firmicutes;Clostridia;Clostridiales;Lachnospiraceae;PAC001287_g | 31 | 0.0879 |
| PAC000661_g | Bacteria;Firmicutes;Clostridia;Clostridiales;Ruminococcaceae;PAC000661_g | 30 | 0.0851 |
| Acutalibacter | Bacteria;Firmicutes;Clostridia;Clostridiales;Ruminococcaceae;Acutalibacter | 28 | 0.0794 |
| PAC000671_g | Bacteria;Firmicutes;Clostridia;Clostridiales;Lachnospiraceae;PAC000671_g | 27 | 0.0765 |
| PAC000197_g | Bacteria;Tenericutes;Mollicutes;PAC001057_o;PAC000197_f;PAC000197_g | 26 | 0.0737 |
| PAC001457_g | Bacteria;Firmicutes;Clostridia;Clostridiales;Lachnospiraceae;PAC001457_g | 26 | 0.0737 |
| PAC000692_g | Bacteria;Firmicutes;Clostridia;Clostridiales;Lachnospiraceae;PAC000692_g | 25 | 0.0709 |
| PAC001528_g | Bacteria;Firmicutes;Erysipelotrichi;Erysipelotrichales;Erysipelotrichaceae;PAC001528_g | 25 | 0.0709 |
| Agathobaculum | Bacteria;Firmicutes;Clostridia;Clostridiales;Ruminococcaceae;Agathobaculum | 24 | 0.068 |
| Mucispirillum | Bacteria;Deferribacteres;Deferribacteres_c;Deferribacterales;Deferribacteraceae;Mucispirillum | 24 | 0.068 |
| PAC001144_g | Bacteria;Firmicutes;Clostridia;Clostridiales;Ruminococcaceae;PAC001144_g | 22 | 0.0624 |
| PAC001313_g | Bacteria;Firmicutes;Clostridia;Clostridiales;Ruminococcaceae;PAC001313_g | 22 | 0.0624 |
| PAC001149_g | Bacteria;Firmicutes;Clostridia;Clostridiales;Lachnospiraceae;PAC001149_g | 21 | 0.0595 |
| PAC002314_g | Bacteria;Firmicutes;Erysipelotrichi;Erysipelotrichales;Erysipelotrichaceae;PAC002314_g | 20 | 0.0567 |
| Harryflintia | Bacteria;Firmicutes;Clostridia;Clostridiales;Ruminococcaceae;Harryflintia | 19 | 0.0539 |
| PAC001308_g | Bacteria;Firmicutes;Clostridia;Clostridiales;Lachnospiraceae;PAC001308_g | 19 | 0.0539 |
| KE993550_g | Bacteria;Firmicutes;Clostridia;Clostridiales;Ruminococcaceae;KE993550_g | 16 | 0.0454 |
| PAC002390_g | Bacteria;Firmicutes;Clostridia;Clostridiales;Dehalobacterium_f;PAC002390_g | 16 | 0.0454 |
| Christensenella | Bacteria;Firmicutes;Clostridia;Clostridiales;Christensenellaceae;Christensenella | 15 | 0.0425 |
| Parabacteroides | Bacteria;Bacteroidetes;Bacteroidia;Bacteroidales;Porphyromonadaceae;Parabacteroides | 14 | 0.0397 |
| PAC001524_g | Bacteria;Firmicutes;Clostridia;Clostridiales;Lachnospiraceae;PAC001524_g | 14 | 0.0397 |
| PAC001573_g | Bacteria;Firmicutes;Clostridia;Clostridiales;Mogibacterium_f;PAC001573_g | 14 | 0.0397 |
| Clostridium_g24 | Bacteria;Firmicutes;Clostridia;Clostridiales;Lachnospiraceae;Clostridium_g24 | 13 | 0.0369 |
| Eubacterium_g17 | Bacteria;Firmicutes;Clostridia;Clostridiales;Lachnospiraceae;Eubacterium_g17 | 12 | 0.034 |
| PAC001270_g | Bacteria;Firmicutes;Clostridia;Clostridiales;Lachnospiraceae;PAC001270_g | 12 | 0.034 |
| Coprococcus_g2 | Bacteria;Firmicutes;Clostridia;Clostridiales;Lachnospiraceae;Coprococcus_g2 | 11 | 0.0312 |
| PAC001219_g | Bacteria;Firmicutes;Clostridia;Clostridiales;Christensenellaceae;PAC001219_g | 11 | 0.0312 |
| PAC001612_g | Bacteria;Firmicutes;Clostridia;Clostridiales;Mogibacterium_f;PAC001612_g | 11 | 0.0312 |
| PAC001062_g | Bacteria;Bacteroidetes;Bacteroidia;Bacteroidales;Muribaculaceae;PAC001062_g | 10 | 0.0284 |
| PAC001599_g | Bacteria;Firmicutes;Clostridia;Clostridiales;Ruminococcaceae;PAC001599_g | 10 | 0.0284 |
| Arthromitus | Bacteria;Firmicutes;Clostridia;Clostridiales;Clostridiaceae;Arthromitus | 9 | 0.0255 |
| Streptococcus | Bacteria;Firmicutes;Bacilli;Lactobacillales;Streptococcaceae;Streptococcus | 9 | 0.0255 |
| GQ451199_g | Bacteria;Firmicutes;Clostridia;Clostridiales;Ruminococcaceae;GQ451199_g | 9 | 0.0255 |
| Bacteroidaceae_uc | Bacteria;Bacteroidetes;Bacteroidia;Bacteroidales;Bacteroidaceae;Bacteroidaceae_uc | 9 | 0.0255 |
| PAC001225_g | Bacteria;Firmicutes;Clostridia;Clostridiales;Lachnospiraceae;PAC001225_g | 8 | 0.0227 |
| Marvinbryantia | Bacteria;Firmicutes;Clostridia;Clostridiales;Lachnospiraceae;Marvinbryantia | 7 | 0.0198 |
| Muribaculum | Bacteria;Bacteroidetes;Bacteroidia;Bacteroidales;Muribaculaceae;Muribaculum | 7 | 0.0198 |
| PAC002460_g | Bacteria;Firmicutes;Clostridia;Clostridiales;Lachnospiraceae;PAC002460_g | 7 | 0.0198 |
| Acinetobacter | Bacteria;Proteobacteria;Gammaproteobacteria;Pseudomonadales;Moraxellaceae;Acinetobacter | 6 | 0.017 |
| Eubacterium_g4 | Bacteria;Firmicutes;Clostridia;Clostridiales;Lachnospiraceae;Eubacterium_g4 | 6 | 0.017 |
| PAC001372_g | Bacteria;Firmicutes;Clostridia;Clostridiales;Lachnospiraceae;PAC001372_g | 6 | 0.017 |
| Coprobacillus | Bacteria;Firmicutes;Erysipelotrichi;Erysipelotrichales;Erysipelotrichaceae;Coprobacillus | 5 | 0.0142 |
| Staphylococcus | Bacteria;Firmicutes;Bacilli;Bacillales;Staphylococcaceae;Staphylococcus | 5 | 0.0142 |
| Clostridium_g35 | Bacteria;Firmicutes;Clostridia;Clostridiales;Lachnospiraceae;Clostridium_g35 | 5 | 0.0142 |
| Ruminococcaceae_uc | Bacteria;Firmicutes;Clostridia;Clostridiales;Ruminococcaceae;Ruminococcaceae_uc | 5 | 0.0142 |
| HM124077_g | Bacteria;Bacteroidetes;Bacteroidia;Bacteroidales;Muribaculaceae;HM124077_g | 4 | 0.0113 |
| HQ765927_g | Bacteria;Firmicutes;Clostridia;Clostridiales;Lachnospiraceae;HQ765927_g | 4 | 0.0113 |
| Blautia | Bacteria;Firmicutes;Clostridia;Clostridiales;Lachnospiraceae;Blautia | 3 | 0.0085 |
| Enterococcus | Bacteria;Firmicutes;Bacilli;Lactobacillales;Enterococcaceae;Enterococcus | 3 | 0.0085 |
| PAC000196_g | Bacteria;Firmicutes;Clostridia;Clostridiales;Lachnospiraceae;PAC000196_g | 3 | 0.0085 |
| PAC001118_g | Bacteria;Firmicutes;Clostridia;Clostridiales;Lachnospiraceae;PAC001118_g | 3 | 0.0085 |
| PAC001266_g | Bacteria;Actinobacteria;Coriobacteriia;Coriobacteriales;Coriobacteriaceae;PAC001266_g | 3 | 0.0085 |
| PAC001323_g | Bacteria;Tenericutes;Mollicutes;PAC001057_o;PAC000197_f;PAC001323_g | 3 | 0.0085 |
| PAC001588_g | Bacteria;Firmicutes;Clostridia;Clostridiales;Lachnospiraceae;PAC001588_g | 3 | 0.0085 |
| Erysipelotrichaceae_uc | Bacteria;Firmicutes;Erysipelotrichi;Erysipelotrichales;Erysipelotrichaceae;Erysipelotrichaceae_uc | 3 | 0.0085 |
| PAC001296_g | Bacteria;Firmicutes;Clostridia;Clostridiales;Lachnospiraceae;PAC001296_g | 2 | 0.0057 |
| PAC002039_g | Bacteria;Firmicutes;Clostridia;Clostridiales;Lachnospiraceae;PAC002039_g | 2 | 0.0057 |
| Agathobacter | Bacteria;Firmicutes;Clostridia;Clostridiales;Lachnospiraceae;Agathobacter | 1 | 0.0028 |
| Anaerostipes | Bacteria;Firmicutes;Clostridia;Clostridiales;Lachnospiraceae;Anaerostipes | 1 | 0.0028 |
| Phocea | Bacteria;Firmicutes;Clostridia;Clostridiales;Ruminococcaceae;Phocea | 1 | 0.0028 |
| Sphingomonas | Bacteria;Proteobacteria;Alphaproteobacteria;Sphingomonadales;Sphingomonadaceae;Sphingomonas | 1 | 0.0028 |
| Eubacterium_g5 | Bacteria;Firmicutes;Clostridia;Clostridiales;Lachnospiraceae;Eubacterium_g5 | 1 | 0.0028 |
| LLKB_g | Bacteria;Firmicutes;Clostridia;Clostridiales;Lachnospiraceae;LLKB_g | 1 | 0.0028 |
| PAC001389_g | Bacteria;Actinobacteria;Coriobacteriia;Coriobacteriales;Coriobacteriaceae;PAC001389_g | 1 | 0.0028 |
| PAC001609_g | Bacteria;Firmicutes;Clostridia;Clostridiales;Mogibacterium_f;PAC001609_g | 1 | 0.0028 |
| PAC001908_g | Bacteria;Firmicutes;Clostridia;Clostridiales;Ruminococcaceae;PAC001908_g | 1 | 0.0028 |
| PAC002042_g | Bacteria;Firmicutes;Clostridia;Clostridiales;Lachnospiraceae;PAC002042_g | 1 | 0.0028 |
| PAC002454_g | Bacteria;Firmicutes;Clostridia;Clostridiales;Lachnospiraceae;PAC002454_g | 1 | 0.0028 |

**Set 2_0.05%_G: Day 10_heme-SCP 0.05%_Genus**

| Taxon name | Taxonomy | Count | Proportion(%) |
|---|---|---|---|
| Bacteroides | Bacteria;Bacteroidetes;Bacteroidia;Bacteroidales;Bacteroidaceae;Bacteroides | 7996 | 17.9536 |
| Pseudoflavonifractor | Bacteria;Firmicutes;Clostridia;Clostridiales;Ruminococcaceae;Pseudoflavonifractor | 5538 | 12.4346 |
| Oscillibacter | Bacteria;Firmicutes;Clostridia;Clostridiales;Ruminococcaceae;Oscillibacter | 4050 | 9.0936 |
| Lactococcus | Bacteria;Firmicutes;Bacilli;Lactobacillales;Streptococcaceae;Lactococcus | 3409 | 7.6543 |
| PAC001516_g | Bacteria;Firmicutes;Clostridia;Clostridiales;Lachnospiraceae;PAC001516_g | 3377 | 7.5825 |
| KE159600_g | Bacteria;Firmicutes;Clostridia;Clostridiales;Lachnospiraceae;KE159600_g | 2595 | 5.8266 |
| Acetatifactor | Bacteria;Firmicutes;Clostridia;Clostridiales;Lachnospiraceae;Acetatifactor | 2216 | 4.9756 |
| PAC002482_g | Bacteria;Bacteroidetes;Bacteroidia;Bacteroidales;AC160630_f;PAC002482_g | 1345 | 3.02 |
| Clostridium_g21 | Bacteria;Firmicutes;Clostridia;Clostridiales;Lachnospiraceae;Clostridium_g21 | 1123 | 2.5215 |
| KE159571_g | Bacteria;Firmicutes;Clostridia;Clostridiales;Lachnospiraceae;KE159571_g | 1033 | 2.3194 |
| Bilophila | Bacteria;Proteobacteria;Deltaproteobacteria;Desulfovibrionales;Desulfovibrionaceae;Bilophila | 1028 | 2.3082 |
| PAC000664_g | Bacteria;Firmicutes;Clostridia;Clostridiales;Lachnospiraceae;PAC000664_g | 939 | 2.1084 |
| Anaerotignum | Bacteria;Firmicutes;Clostridia;Clostridiales;Lachnospiraceae;Anaerotignum | 836 | 1.8771 |
| PAC001402_g | Bacteria;Firmicutes;Clostridia;Clostridiales;Ruminococcaceae;PAC001402_g | 722 | 1.6211 |
| PAC001112_g | Bacteria;Bacteroidetes;Bacteroidia;Bacteroidales;Muribaculaceae;PAC001112_g | 520 | 1.1676 |
| PAC001092_g | Bacteria;Firmicutes;Clostridia;Clostridiales;Lachnospiraceae;PAC001092_g | 505 | 1.1339 |
| Eubacterium_g23 | Bacteria;Firmicutes;Clostridia;Clostridiales;Ruminococcaceae;Eubacterium_g23 | 420 | 0.943 |
| PAC002375_g | Bacteria;Firmicutes;Clostridia;Clostridiales;Lachnospiraceae;PAC002375_g | 404 | 0.9071 |
| PAC001063_g | Bacteria;Bacteroidetes;Bacteroidia;Bacteroidales;Muribaculaceae;PAC001063_g | 402 | 0.9026 |
| PAC001400_g | Bacteria;Firmicutes;Clostridia;Clostridiales;Peptococcaceae;PAC001400_g | 386 | 0.8667 |
| Alistipes | Bacteria;Bacteroidetes;Bacteroidia;Bacteroidales;Rikenellaceae;Alistipes | 378 | 0.8487 |
| KE159605_g | Bacteria;Firmicutes;Clostridia;Clostridiales;Lachnospiraceae;KE159605_g | 316 | 0.7095 |
| PAC000198_g | Bacteria;Bacteroidetes;Bacteroidia;Bacteroidales;Muribaculaceae;PAC000198_g | 316 | 0.7095 |
| Corynebacterium | Bacteria;Actinobacteria;Actinobacteria_c;Corynebacteriales;Corynebacteriaceae;Corynebacterium | 274 | 0.6152 |
| PAC001778_g | Bacteria;Firmicutes;Clostridia;Clostridiales;Ruminococcaceae;PAC001778_g | 249 | 0.5591 |
| Frisingicoccus | Bacteria;Firmicutes;Clostridia;Clostridiales;Lachnospiraceae;Frisingicoccus | 231 | 0.5187 |
| PAC001385_g | Bacteria;Firmicutes;Clostridia;Clostridiales;Lachnospiraceae;PAC001385_g | 227 | 0.5097 |
| PAC001228_g | Bacteria;Firmicutes;Clostridia;Clostridiales;Lachnospiraceae;PAC001228_g | 215 | 0.4827 |
| Caproiciproducens | Bacteria;Firmicutes;Clostridia;Clostridiales;Ruminococcaceae;Caproiciproducens | 192 | 0.4311 |
| Lactobacillus | Bacteria;Firmicutes;Bacilli;Lactobacillales;Lactobacillaceae;Lactobacillus | 187 | 0.4199 |
| PAC001103_g | Bacteria;Firmicutes;Clostridia;Clostridiales;Lachnospiraceae;PAC001103_g | 184 | 0.4131 |
| PAC000692_g | Bacteria;Firmicutes;Clostridia;Clostridiales;Lachnospiraceae;PAC000692_g | 172 | 0.3862 |
| Anaerotruncus | Bacteria;Firmicutes;Clostridia;Clostridiales;Ruminococcaceae;Anaerotruncus | 156 | 0.3503 |
| PAC001221_g | Bacteria;Firmicutes;Clostridia;Clostridiales;Dehalobacterium_f;PAC001221_g | 152 | 0.3413 |
| PAC001165_g | Bacteria;Firmicutes;Clostridia;Clostridiales;Lachnospiraceae;PAC001165_g | 140 | 0.3143 |
| PAC001308_g | Bacteria;Firmicutes;Clostridia;Clostridiales;Lachnospiraceae;PAC001308_g | 133 | 0.2986 |
| PAC001544_g | Bacteria;Firmicutes;Clostridia;Clostridiales;Lachnospiraceae;PAC001544_g | 124 | 0.2784 |
| PAC001043_g | Bacteria;Firmicutes;Clostridia;Clostridiales;Lachnospiraceae;PAC001043_g | 111 | 0.2492 |
| Eubacterium_g6 | Bacteria;Firmicutes;Clostridia;Clostridiales;Lachnospiraceae;Eubacterium_g6 | 109 | 0.2447 |
| PAC001066_g | Bacteria;Bacteroidetes;Bacteroidia;Bacteroidales;Muribaculaceae;PAC001066_g | 99 | 0.2223 |
| PAC001068_g | Bacteria;Bacteroidetes;Bacteroidia;Bacteroidales;Muribaculaceae;PAC001068_g | 92 | 0.2066 |
| Mucispirillum | Bacteria;Deferribacteres;Deferribacteres_c;Deferribacterales;Deferribacteraceae;Mucispirillum | 89 | 0.1998 |
| PAC001201_g | Bacteria;Firmicutes;Clostridia;Clostridiales;Lachnospiraceae;PAC001201_g | 87 | 0.1953 |
| PAC001457_g | Bacteria;Firmicutes;Clostridia;Clostridiales;Lachnospiraceae;PAC001457_g | 85 | 0.1909 |
| PAC001149_g | Bacteria;Firmicutes;Clostridia;Clostridiales;Lachnospiraceae;PAC001149_g | 82 | 0.1841 |
| PAC001074_g | Bacteria;Bacteroidetes;Bacteroidia;Bacteroidales;Muribaculaceae;PAC001074_g | 69 | 0.1549 |
| PAC000661_g | Bacteria;Firmicutes;Clostridia;Clostridiales;Ruminococcaceae;PAC000661_g | 68 | 0.1527 |
| Desulfovibrio | Bacteria;Proteobacteria;Deltaproteobacteria;Desulfovibrionales;Desulfovibrionaceae;Desulfovibrio | 67 | 0.1504 |
| Acutalibacter | Bacteria;Firmicutes;Clostridia;Clostridiales;Ruminococcaceae;Acutalibacter | 61 | 0.137 |
| Clostridium_g24 | Bacteria;Firmicutes;Clostridia;Clostridiales;Lachnospiraceae;Clostridium_g24 | 54 | 0.1212 |
| PAC001524_g | Bacteria;Firmicutes;Clostridia;Clostridiales;Lachnospiraceae;PAC001524_g | 54 | 0.1212 |
| PAC001270_g | Bacteria;Firmicutes;Clostridia;Clostridiales;Lachnospiraceae;PAC001270_g | 52 | 0.1168 |
| PAC001138_g | Bacteria;Firmicutes;Clostridia;Clostridiales;Lachnospiraceae;PAC001138_g | 49 | 0.11 |
| Paludicola | Bacteria;Firmicutes;Clostridia;Clostridiales;Ruminococcaceae;Paludicola | 43 | 0.0965 |
| PAC001612_g | Bacteria;Firmicutes;Clostridia;Clostridiales;Mogibacterium_f;PAC001612_g | 43 | 0.0965 |
| Massilioclostridium | Bacteria;Firmicutes;Clostridia;Clostridiales;Ruminococcaceae;Massilioclostridium | 40 | 0.0898 |
| KE159797_g | Bacteria;Firmicutes;Clostridia;Clostridiales;Lachnospiraceae;KE159797_g | 32 | 0.0719 |
| PAC001588_g | Bacteria;Firmicutes;Clostridia;Clostridiales;Lachnospiraceae;PAC001588_g | 31 | 0.0696 |
| Lachnospiraceae_uc | Bacteria;Firmicutes;Clostridia;Clostridiales;Lachnospiraceae;Lachnospiraceae_uc | 31 | 0.0696 |
| PAC001225_g | Bacteria;Firmicutes;Clostridia;Clostridiales;Lachnospiraceae;PAC001225_g | 29 | 0.0651 |
| Eubacterium_g17 | Bacteria;Firmicutes;Clostridia;Clostridiales;Lachnospiraceae;Eubacterium_g17 | 27 | 0.0606 |
| PAC000671_g | Bacteria;Firmicutes;Clostridia;Clostridiales;Lachnospiraceae;PAC000671_g | 27 | 0.0606 |
| PAC000748_g | Bacteria;Firmicutes;Clostridia;Clostridiales;Ruminococcaceae;PAC000748_g | 26 | 0.0584 |
| PAC001313_g | Bacteria;Firmicutes;Clostridia;Clostridiales;Ruminococcaceae;PAC001313_g | 26 | 0.0584 |
| Christensenella | Bacteria;Firmicutes;Clostridia;Clostridiales;Christensenellaceae;Christensenella | 25 | 0.0561 |
| Enterorhabdus | Bacteria;Actinobacteria;Coriobacteriia;Coriobacteriales;Coriobacteriaceae;Enterorhabdus | 25 | 0.0561 |
| Sporobacter | Bacteria;Firmicutes;Clostridia;Clostridiales;Ruminococcaceae;Sporobacter | 25 | 0.0561 |
| PAC002460_g | Bacteria;Firmicutes;Clostridia;Clostridiales;Lachnospiraceae;PAC002460_g | 25 | 0.0561 |
| Bacillus | Bacteria;Firmicutes;Bacilli;Bacillales;Bacillaceae;Bacillus | 23 | 0.0516 |
| Harryflintia | Bacteria;Firmicutes;Clostridia;Clostridiales;Ruminococcaceae;Harryflintia | 23 | 0.0516 |
| PAC001372_g | Bacteria;Firmicutes;Clostridia;Clostridiales;Lachnospiraceae;PAC001372_g | 22 | 0.0494 |
| PAC002390_g | Bacteria;Firmicutes;Clostridia;Clostridiales;Dehalobacterium_f;PAC002390_g | 21 | 0.0472 |
| Arthromitus | Bacteria;Firmicutes;Clostridia;Clostridiales;Clostridiaceae;Arthromitus | 17 | 0.0382 |
| Parabacteroides | Bacteria;Bacteroidetes;Bacteroidia;Bacteroidales;Porphyromonadaceae;Parabacteroides | 17 | 0.0382 |
| Gemella | Bacteria;Firmicutes;Bacilli;Bacillales;Gemella_f;Gemella | 16 | 0.0359 |
| Ruminococcaceae_uc | Bacteria;Firmicutes;Clostridia;Clostridiales;Ruminococcaceae;Ruminococcaceae_uc | 16 | 0.0359 |
| Streptococcus | Bacteria;Firmicutes;Bacilli;Lactobacillales;Streptococcaceae;Streptococcus | 14 | 0.0314 |
| PAC001062_g | Bacteria;Bacteroidetes;Bacteroidia;Bacteroidales;Muribaculaceae;PAC001062_g | 14 | 0.0314 |
| Agathobaculum | Bacteria;Firmicutes;Clostridia;Clostridiales;Ruminococcaceae;Agathobaculum | 11 | 0.0247 |
| Escherichia | Bacteria;Proteobacteria;Gammaproteobacteria;Enterobacterales;Enterobacteriaceae;Escherichia | 11 | 0.0247 |
| PAC001200_g | Bacteria;Firmicutes;Clostridia;Clostridiales;Lachnospiraceae;PAC001200_g | 11 | 0.0247 |
| Emergencia | Bacteria;Firmicutes;Clostridia;Clostridiales;Mogibacterium_f;Emergencia | 9 | 0.0202 |
| KE159810_g | Bacteria;Firmicutes;Clostridia;Clostridiales;Lachnospiraceae;KE159810_g | 9 | 0.0202 |
| PAC001144_g | Bacteria;Firmicutes;Clostridia;Clostridiales;Ruminococcaceae;PAC001144_g | 9 | 0.0202 |
| PAC001266_g | Bacteria;Actinobacteria;Coriobacteriia;Coriobacteriales;Coriobacteriaceae;PAC001266_g | 9 | 0.0202 |
| PAC001323_g | Bacteria;Tenericutes;Mollicutes;PAC001057_o;PAC000197_f;PAC001323_g | 8 | 0.018 |
| PAC002039_g | Bacteria;Firmicutes;Clostridia;Clostridiales;Lachnospiraceae;PAC002039_g | 7 | 0.0157 |
| Akkermansia | Bacteria;Verrucomicrobia;Verrucomicrobiae;Verrucomicrobiales;Akkermansiaceae;Akkermansia | 6 | 0.0135 |
| PAC000186_g | Bacteria;Bacteroidetes;Bacteroidia;Bacteroidales;Muribaculaceae;PAC000186_g | 6 | 0.0135 |
| PAC001528_g | Bacteria;Firmicutes;Erysipelotrichi;Erysipelotrichales;Erysipelotrichaceae;PAC001528_g | 5 | 0.0112 |
| PAC002454_g | Bacteria;Firmicutes;Clostridia;Clostridiales;Lachnospiraceae;PAC002454_g | 5 | 0.0112 |
| CCYH_g | Bacteria;Firmicutes;Clostridia;Clostridiales;Ruminococcaceae;CCYH_g | 4 | 0.009 |
| Coprococcus_g2 | Bacteria;Firmicutes;Clostridia;Clostridiales;Lachnospiraceae;Coprococcus_g2 | 4 | 0.009 |
| Eubacterium_g4 | Bacteria;Firmicutes;Clostridia;Clostridiales;Lachnospiraceae;Eubacterium_g4 | 4 | 0.009 |
| Bacteroidaceae_uc | Bacteria;Bacteroidetes;Bacteroidia;Bacteroidales;Bacteroidaceae;Bacteroidaceae_uc | 4 | 0.009 |
| Clostridium | Bacteria;Firmicutes;Clostridia;Clostridiales;Clostridiaceae;Clostridium | 3 | 0.0067 |
| Muribaculum | Bacteria;Bacteroidetes;Bacteroidia;Bacteroidales;Muribaculaceae;Muribaculum | 3 | 0.0067 |
| Parvibacter | Bacteria;Actinobacteria;Coriobacteriia;Coriobacteriales;Coriobacteriaceae;Parvibacter | 3 | 0.0067 |
| Romboutsia | Bacteria;Firmicutes;Clostridia;Clostridiales;Peptostreptococcaceae;Romboutsia | 3 | 0.0067 |
| Clostridium_g6 | Bacteria;Firmicutes;Erysipelotrichi;Erysipelotrichales;Erysipelotrichaceae;Clostridium_g6 | 3 | 0.0067 |
| GQ451199_g | Bacteria;Firmicutes;Clostridia;Clostridiales;Ruminococcaceae;GQ451199_g | 3 | 0.0067 |
| PAC001219_g | Bacteria;Firmicutes;Clostridia;Clostridiales;Christensenellaceae;PAC001219_g | 3 | 0.0067 |
| PAC001296_g | Bacteria;Firmicutes;Clostridia;Clostridiales;Lachnospiraceae;PAC001296_g | 3 | 0.0067 |
| PAC001573_g | Bacteria;Firmicutes;Clostridia;Clostridiales;Mogibacterium_f;PAC001573_g | 3 | 0.0067 |
| Herbiconiux | Bacteria;Actinobacteria;Actinobacteria_c;Micrococcales;Microbacteriaceae;Herbiconiux | 2 | 0.0045 |
| Parasutterella | Bacteria;Proteobacteria;Betaproteobacteria;Burkholderiales;Sutterellaceae;Parasutterella | 2 | 0.0045 |
| LLKB_g | Bacteria;Firmicutes;Clostridia;Clostridiales;Lachnospiraceae;LLKB_g | 2 | 0.0045 |
| PAC000196_g | Bacteria;Firmicutes;Clostridia;Clostridiales;Lachnospiraceae;PAC000196_g | 2 | 0.0045 |
| PAC000197_g | Bacteria;Tenericutes;Mollicutes;PAC001057_o;PAC000197_f;PAC000197_g | 2 | 0.0045 |
| PAC000673_g | Bacteria;Firmicutes;Clostridia;Clostridiales;Lachnospiraceae;PAC000673_g | 2 | 0.0045 |
| PAC001141_g | Bacteria;Firmicutes;Clostridia;Clostridiales;Christensenellaceae;PAC001141_g | 2 | 0.0045 |
| PAC002042_g | Bacteria;Firmicutes;Clostridia;Clostridiales;Lachnospiraceae;PAC002042_g | 2 | 0.0045 |
| PAC002471_g | Bacteria;Firmicutes;Clostridia;Clostridiales;Lachnospiraceae;PAC002471_g | 2 | 0.0045 |
| PAC002528_g | Bacteria;Actinobacteria;Coriobacteriia;Coriobacteriales;Coriobacteriaceae;PAC002528_g | 2 | 0.0045 |
| Clostridiales_uc | Bacteria;Firmicutes;Clostridia;Clostridiales;Clostridiales_uc | 1 | 0.0022 |
| Blautia | Bacteria;Firmicutes;Clostridia;Clostridiales;Lachnospiraceae;Blautia | 1 | 0.0022 |
| Kineothrix | Bacteria;Firmicutes;Clostridia;Clostridiales;Lachnospiraceae;Kineothrix | 1 | 0.0022 |
| Lysinibacillus | Bacteria;Firmicutes;Bacilli;Bacillales;Planococcaceae;Lysinibacillus | 1 | 0.0022 |
| Staphylococcus | Bacteria;Firmicutes;Bacilli;Bacillales;Staphylococcaceae;Staphylococcus | 1 | 0.0022 |
| PAC001097_g | Bacteria;Firmicutes;Clostridia;Clostridiales;Lachnospiraceae;PAC001097_g | 1 | 0.0022 |
| PAC001116_g | Bacteria;Firmicutes;Clostridia;Clostridiales;Lachnospiraceae;PAC001116_g | 1 | 0.0022 |
| PAC001360_g | Bacteria;Firmicutes;Clostridia;Clostridiales;Christensenellaceae;PAC001360_g | 1 | 0.0022 |
| PAC001609_g | Bacteria;Firmicutes;Clostridia;Clostridiales;Mogibacterium_f;PAC001609_g | 1 | 0.0022 |
| PAC001908_g | Bacteria;Firmicutes;Clostridia;Clostridiales;Ruminococcaceae;PAC001908_g | 1 | 0.0022 |
| PAC002178_g | Bacteria;Firmicutes;Clostridia;Clostridiales;Lachnospiraceae;PAC002178_g | 1 | 0.0022 |
| PAC002314_g | Bacteria;Firmicutes;Erysipelotrichi;Erysipelotrichales;Erysipelotrichaceae;PAC002314_g | 1 | 0.0022 |

**Set 2_0.5%_G: Day 10_heme-SCP 0.5%_Genus**

| Taxon name | Taxonomy | Count | Proportion(%) |
|---|---|---|---|
| Bacteroides | Bacteria;Bacteroidetes;Bacteroidia;Bacteroidales;Bacteroidaceae;Bacteroides | 5682 | 14.3318 |
| KE159600_g | Bacteria;Firmicutes;Clostridia;Clostridiales;Lachnospiraceae;KE159600_g | 5511 | 13.9005 |
| Oscillibacter | Bacteria;Firmicutes;Clostridia;Clostridiales;Ruminococcaceae;Oscillibacter | 4045 | 10.2028 |
| Pseudoflavonifractor | Bacteria;Firmicutes;Clostridia;Clostridiales;Ruminococcaceae;Pseudoflavonifractor | 3268 | 8.243 |
| PAC001516_g | Bacteria;Firmicutes;Clostridia;Clostridiales;Lachnospiraceae;PAC001516_g | 2221 | 5.6021 |
| Acetatifactor | Bacteria;Firmicutes;Clostridia;Clostridiales;Lachnospiraceae;Acetatifactor | 1663 | 4.1946 |
| KE159571_g | Bacteria;Firmicutes;Clostridia;Clostridiales;Lachnospiraceae;KE159571_g | 1611 | 4.0635 |
| Anaerotignum | Bacteria;Firmicutes;Clostridia;Clostridiales;Lachnospiraceae;Anaerotignum | 1177 | 2.9688 |
| Bilophila | Bacteria;Proteobacteria;Deltaproteobacteria;Desulfovibrionales;Desulfovibrionaceae;Bilophila | 1040 | 2.6232 |
| Eubacterium_g23 | Bacteria;Firmicutes;Clostridia;Clostridiales;Ruminococcaceae;Eubacterium_g23 | 1017 | 2.5652 |
| PAC002482_g | Bacteria;Bacteroidetes;Bacteroidia;Bacteroidales;AC160630_f;PAC002482_g | 919 | 2.318 |
| Clostridium_g21 | Bacteria;Firmicutes;Clostridia;Clostridiales;Lachnospiraceae;Clostridium_g21 | 913 | 2.3029 |
| Alistipes | Bacteria;Bacteroidetes;Bacteroidia;Bacteroidales;Rikenellaceae;Alistipes | 902 | 2.2751 |
| PAC001287_g | Bacteria;Firmicutes;Clostridia;Clostridiales;Lachnospiraceae;PAC001287_g | 837 | 2.1112 |
| PAC001402_g | Bacteria;Firmicutes;Clostridia;Clostridiales;Ruminococcaceae;PAC001402_g | 646 | 1.6294 |
| PAC001141_g | Bacteria;Firmicutes;Clostridia;Clostridiales;Christensenellaceae;PAC001141_g | 539 | 1.3595 |
| Corynebacterium | Bacteria;Actinobacteria;Actinobacteria_c;Corynebacteriales;Corynebacteriaceae;Corynebacterium | 516 | 1.3015 |
| PAC001112_g | Bacteria;Bacteroidetes;Bacteroidia;Bacteroidales;Muribaculaceae;PAC001112_g | 452 | 1.1401 |
| PAC000664_g | Bacteria;Firmicutes;Clostridia;Clostridiales;Lachnospiraceae;PAC000664_g | 443 | 1.1174 |
| Lactococcus | Bacteria;Firmicutes;Bacilli;Lactobacillales;Streptococcaceae;Lactococcus | 370 | 0.9333 |
| PAC001544_g | Bacteria;Firmicutes;Clostridia;Clostridiales;Lachnospiraceae;PAC001544_g | 354 | 0.8929 |
| PAC000661_g | Bacteria;Firmicutes;Clostridia;Clostridiales;Ruminococcaceae;PAC000661_g | 333 | 0.8399 |
| PAC000198_g | Bacteria;Bacteroidetes;Bacteroidia;Bacteroidales;Muribaculaceae;PAC000198_g | 311 | 0.7844 |
| Anaerotruncus | Bacteria;Firmicutes;Clostridia;Clostridiales;Ruminococcaceae;Anaerotruncus | 308 | 0.7769 |
| PAC002375_g | Bacteria;Firmicutes;Clostridia;Clostridiales;Lachnospiraceae;PAC002375_g | 258 | 0.6508 |
| Frisingicoccus | Bacteria;Firmicutes;Clostridia;Clostridiales;Lachnospiraceae;Frisingicoccus | 241 | 0.6079 |
| PAC001068_g | Bacteria;Bacteroidetes;Bacteroidia;Bacteroidales;Muribaculaceae;PAC001068_g | 218 | 0.5499 |
| PAC001066_g | Bacteria;Bacteroidetes;Bacteroidia;Bacteroidales;Muribaculaceae;PAC001066_g | 200 | 0.5045 |
| PAC001103_g | Bacteria;Firmicutes;Clostridia;Clostridiales;Lachnospiraceae;PAC001103_g | 197 | 0.4969 |
| PAC001074_g | Bacteria;Bacteroidetes;Bacteroidia;Bacteroidales;Muribaculaceae;PAC001074_g | 170 | 0.4288 |
| PAC001092_g | Bacteria;Firmicutes;Clostridia;Clostridiales;Lachnospiraceae;PAC001092_g | 170 | 0.4288 |
| Lactobacillus | Bacteria;Firmicutes;Bacilli;Lactobacillales;Lactobacillaceae;Lactobacillus | 166 | 0.4187 |
| PAC001063_g | Bacteria;Bacteroidetes;Bacteroidia;Bacteroidales;Muribaculaceae;PAC001063_g | 166 | 0.4187 |
| Caproiciproducens | Bacteria;Firmicutes;Clostridia;Clostridiales;Ruminococcaceae;Caproiciproducens | 151 | 0.3809 |
| PAC001165_g | Bacteria;Firmicutes;Clostridia;Clostridiales;Lachnospiraceae;PAC001165_g | 139 | 0.3506 |
| KE159797_g | Bacteria;Firmicutes;Clostridia;Clostridiales;Lachnospiraceae;KE159797_g | 136 | 0.343 |
| KE159605_g | Bacteria;Firmicutes;Clostridia;Clostridiales;Lachnospiraceae;KE159605_g | 124 | 0.3128 |
| PAC001228_g | Bacteria;Firmicutes;Clostridia;Clostridiales;Lachnospiraceae;PAC001228_g | 115 | 0.2901 |
| Eubacterium_g6 | Bacteria;Firmicutes;Clostridia;Clostridiales;Lachnospiraceae;Eubacterium_g6 | 114 | 0.2875 |
| PAC001400_g | Bacteria;Firmicutes;Clostridia;Clostridiales;Peptococcaceae;PAC001400_g | 109 | 0.2749 |
| PAC001221_g | Bacteria;Firmicutes;Clostridia;Clostridiales;Dehalobacterium_f;PAC001221_g | 99 | 0.2497 |
| PAC001138_g | Bacteria;Firmicutes;Clostridia;Clostridiales;Lachnospiraceae;PAC001138_g | 90 | 0.227 |
| PAC001360_g | Bacteria;Firmicutes;Clostridia;Clostridiales;Christensenellaceae;PAC001360_g | 81 | 0.2043 |
| PAC001043_g | Bacteria;Firmicutes;Clostridia;Clostridiales;Lachnospiraceae;PAC001043_g | 80 | 0.2018 |
| PAC001385_g | Bacteria;Firmicutes;Clostridia;Clostridiales;Lachnospiraceae;PAC001385_g | 77 | 0.1942 |
| Mucispirillum | Bacteria;Deferribacteres;Deferribacteres_c;Deferribacterales;Deferribacteraceae;Mucispirillum | 73 | 0.1841 |
| PAC000748_g | Bacteria;Firmicutes;Clostridia;Clostridiales;Ruminococcaceae;PAC000748_g | 73 | 0.1841 |
| Parabacteroides | Bacteria;Bacteroidetes;Bacteroidia;Bacteroidales;Porphyromonadaceae;Parabacteroides | 72 | 0.1816 |
| Sporobacter | Bacteria;Firmicutes;Clostridia;Clostridiales;Ruminococcaceae;Sporobacter | 69 | 0.174 |
| PAC001778_g | Bacteria;Firmicutes;Clostridia;Clostridiales;Ruminococcaceae;PAC001778_g | 67 | 0.169 |
| Paludicola | Bacteria;Firmicutes;Clostridia;Clostridiales;Ruminococcaceae;Paludicola | 61 | 0.1539 |
| Acutalibacter | Bacteria;Firmicutes;Clostridia;Clostridiales;Ruminococcaceae;Acutalibacter | 57 | 0.1438 |
| PAC001524_g | Bacteria;Firmicutes;Clostridia;Clostridiales;Lachnospiraceae;PAC001524_g | 50 | 0.1261 |
| Massilioclostridium | Bacteria;Firmicutes;Clostridia;Clostridiales;Ruminococcaceae;Massilioclostridium | 47 | 0.1185 |
| PAC001308_g | Bacteria;Firmicutes;Clostridia;Clostridiales;Lachnospiraceae;PAC001308_g | 45 | 0.1135 |
| PAC001457_g | Bacteria;Firmicutes;Clostridia;Clostridiales;Lachnospiraceae;PAC001457_g | 43 | 0.1085 |
| Lachnospiraceae_uc | Bacteria;Firmicutes;Clostridia;Clostridiales;Lachnospiraceae;Lachnospiraceae_uc | 42 | 0.1059 |
| Bacillus | Bacteria;Firmicutes;Bacilli;Bacillales;Bacillaceae;Bacillus | 40 | 0.1009 |
| LARJ_g | Bacteria;Proteobacteria;Alphaproteobacteria;Rhodospirillales;Rhodospirillaceae;LARJ_g | 39 | 0.0984 |
| Harryflintia | Bacteria;Firmicutes;Clostridia;Clostridiales;Ruminococcaceae;Harryflintia | 37 | 0.0933 |
| PAC001062_g | Bacteria;Bacteroidetes;Bacteroidia;Bacteroidales;Muribaculaceae;PAC001062_g | 35 | 0.0883 |
| Arthromitus | Bacteria;Firmicutes;Clostridia;Clostridiales;Clostridiaceae;Arthromitus | 32 | 0.0807 |
| Escherichia | Bacteria;Proteobacteria;Gammaproteobacteria;Enterobacterales;Enterobacteriaceae;Escherichia | 28 | 0.0706 |
| PAC001270_g | Bacteria;Firmicutes;Clostridia;Clostridiales;Lachnospiraceae;PAC001270_g | 28 | 0.0706 |
| Eubacterium_g17 | Bacteria;Firmicutes;Clostridia;Clostridiales;Lachnospiraceae;Eubacterium_g17 | 26 | 0.0656 |
| PAC001313_g | Bacteria;Firmicutes;Clostridia;Clostridiales;Ruminococcaceae;PAC001313_g | 26 | 0.0656 |
| PAC002169_g | Bacteria;Firmicutes;Clostridia;Clostridiales;Christensenellaceae;PAC002169_g | 26 | 0.0656 |
| PAC000692_g | Bacteria;Firmicutes;Clostridia;Clostridiales;Lachnospiraceae;PAC000692_g | 24 | 0.0605 |
| KE159538_g | Bacteria;Firmicutes;Clostridia;Clostridiales;Lachnospiraceae;KE159538_g | 22 | 0.0555 |
| PAC001225_g | Bacteria;Firmicutes;Clostridia;Clostridiales;Lachnospiraceae;PAC001225_g | 22 | 0.0555 |
| Christensenella | Bacteria;Firmicutes;Clostridia;Clostridiales;Christensenellaceae;Christensenella | 20 | 0.0504 |
| Desulfovibrio | Bacteria;Proteobacteria;Deltaproteobacteria;Desulfovibrionales;Desulfovibrionaceae;Desulfovibrio | 20 | 0.0504 |
| Gemella | Bacteria;Firmicutes;Bacilli;Bacillales;Gemella_f;Gemella | 20 | 0.0504 |
| PAC001144_g | Bacteria;Firmicutes;Clostridia;Clostridiales;Ruminococcaceae;PAC001144_g | 20 | 0.0504 |
| PAC001573_g | Bacteria;Firmicutes;Clostridia;Clostridiales;Mogibacterium_f;PAC001573_g | 20 | 0.0504 |
| PAC002390_g | Bacteria;Firmicutes;Clostridia;Clostridiales;Dehalobacterium_f;PAC002390_g | 20 | 0.0504 |
| Coprococcus_g2 | Bacteria;Firmicutes;Clostridia;Clostridiales;Lachnospiraceae;Coprococcus_g2 | 14 | 0.0353 |
| PAC000671_g | Bacteria;Firmicutes;Clostridia;Clostridiales;Lachnospiraceae;PAC000671_g | 14 | 0.0353 |
| Ruminococcaceae_uc | Bacteria;Firmicutes;Clostridia;Clostridiales;Ruminococcaceae;Ruminococcaceae_uc | 14 | 0.0353 |
| Clostridium_g24 | Bacteria;Firmicutes;Clostridia;Clostridiales;Lachnospiraceae;Clostridium_g24 | 13 | 0.0328 |
| PAC001612_g | Bacteria;Firmicutes;Clostridia;Clostridiales;Mogibacterium_f;PAC001612_g | 12 | 0.0303 |
| Enterorhabdus | Bacteria;Actinobacteria;Coriobacteriia;Coriobacteriales;Coriobacteriaceae;Enterorhabdus | 11 | 0.0277 |
| PAC000186_g | Bacteria;Bacteroidetes;Bacteroidia;Bacteroidales;Muribaculaceae;PAC000186_g | 11 | 0.0277 |
| PAC001372_g | Bacteria;Firmicutes;Clostridia;Clostridiales;Lachnospiraceae;PAC001372_g | 10 | 0.0252 |
| Streptococcus | Bacteria;Firmicutes;Bacilli;Lactobacillales;Streptococcaceae;Streptococcus | 9 | 0.0227 |
| PAC001149_g | Bacteria;Firmicutes;Clostridia;Clostridiales;Lachnospiraceae;PAC001149_g | 9 | 0.0227 |
| PAC001201_g | Bacteria;Firmicutes;Clostridia;Clostridiales;Lachnospiraceae;PAC001201_g | 9 | 0.0227 |
| PAC001362_g | Bacteria;Firmicutes;Erysipelotrichi;Erysipelotrichales;Erysipelotrichaceae;PAC001362_g | 9 | 0.0227 |
| PAC001651_g | Bacteria;Firmicutes;Clostridia;Clostridiales;Ruminococcaceae;PAC001651_g | 9 | 0.0227 |
| Parasutterella | Bacteria;Proteobacteria;Betaproteobacteria;Burkholderiales;Sutterellaceae;Parasutterella | 8 | 0.0202 |
| PAC001219_g | Bacteria;Firmicutes;Clostridia;Clostridiales;Christensenellaceae;PAC001219_g | 8 | 0.0202 |
| PAC002460_g | Bacteria;Firmicutes;Clostridia;Clostridiales;Lachnospiraceae;PAC002460_g | 8 | 0.0202 |
| PAC002471_g | Bacteria;Firmicutes;Clostridia;Clostridiales;Lachnospiraceae;PAC002471_g | 8 | 0.0202 |
| PAC001200_g | Bacteria;Firmicutes;Clostridia;Clostridiales;Lachnospiraceae;PAC001200_g | 7 | 0.0177 |
| PAC001528_g | Bacteria;Firmicutes;Erysipelotrichi;Erysipelotrichales;Erysipelotrichaceae;PAC001528_g | 7 | 0.0177 |
| GQ451199_g | Bacteria;Firmicutes;Clostridia;Clostridiales;Ruminococcaceae;GQ451199_g | 6 | 0.0151 |
| PAC000197_g | Bacteria;Tenericutes;Mollicutes;PAC001057_o;PAC000197_f;PAC000197_g | 6 | 0.0151 |
| Bacteroidaceae_uc | Bacteria;Bacteroidetes;Bacteroidia;Bacteroidales;Bacteroidaceae;Bacteroidaceae_uc | 6 | 0.0151 |
| PAC001301_g | Bacteria;Firmicutes;Clostridia;Clostridiales;Christensenellaceae;PAC001301_g | 5 | 0.0126 |
| Clostridium | Bacteria;Firmicutes;Clostridia;Clostridiales;Clostridiaceae;Clostridium | 4 | 0.0101 |
| PAC001236_g | Bacteria;Firmicutes;Clostridia;Clostridiales;Mogibacterium_f;PAC001236_g | 4 | 0.0101 |
| PAC001588_g | Bacteria;Firmicutes;Clostridia;Clostridiales;Lachnospiraceae;PAC001588_g | 4 | 0.0101 |
| PAC001908_g | Bacteria;Firmicutes;Clostridia;Clostridiales;Ruminococcaceae;PAC001908_g | 4 | 0.0101 |
| PAC002454_g | Bacteria;Firmicutes;Clostridia;Clostridiales;Lachnospiraceae;PAC002454_g | 4 | 0.0101 |
| Emergencia | Bacteria;Firmicutes;Clostridia;Clostridiales;Mogibacterium_f;Emergencia | 3 | 0.0076 |
| Neisseria | Bacteria;Proteobacteria;Betaproteobacteria;Neisseriales;Neisseriaceae;Neisseria | 3 | 0.0076 |
| PAC001323_g | Bacteria;Tenericutes;Mollicutes;PAC001057_o;PAC000197_f;PAC001323_g | 3 | 0.0076 |
| Agathobaculum | Bacteria;Firmicutes;Clostridia;Clostridiales;Ruminococcaceae;Agathobaculum | 2 | 0.005 |
| Paenibacillus | Bacteria;Firmicutes;Bacilli;Bacillales;Paenibacillaceae;Paenibacillus | 2 | 0.005 |
| Brucellaceae_g | Bacteria;Proteobacteria;Alphaproteobacteria;Rhizobiales;Brucellaceae;Brucellaceae_g | 2 | 0.005 |
| Eubacterium_g4 | Bacteria;Firmicutes;Clostridia;Clostridiales;Lachnospiraceae;Eubacterium_g4 | 2 | 0.005 |
| PAC000196_g | Bacteria;Firmicutes;Clostridia;Clostridiales;Lachnospiraceae;PAC000196_g | 2 | 0.005 |
| PAC001108_g | Bacteria;Tenericutes;Mollicutes;PAC001057_o;PAC001057_f;PAC001108_g | 2 | 0.005 |
| Faecalimonas | Bacteria;Firmicutes;Clostridia;Clostridiales;Lachnospiraceae;Faecalimonas | 1 | 0.0025 |
| Muribaculum | Bacteria;Bacteroidetes;Bacteroidia;Bacteroidales;Muribaculaceae;Muribaculum | 1 | 0.0025 |
| Prevotella | Bacteria;Bacteroidetes;Bacteroidia;Bacteroidales;Prevotellaceae;Prevotella | 1 | 0.0025 |
| Clostridium_g35 | Bacteria;Firmicutes;Clostridia;Clostridiales;Lachnospiraceae;Clostridium_g35 | 1 | 0.0025 |
| KE159810_g | Bacteria;Firmicutes;Clostridia;Clostridiales;Lachnospiraceae;KE159810_g | 1 | 0.0025 |
| PAC001389_g | Bacteria;Actinobacteria;Coriobacteriia;Coriobacteriales;Coriobacteriaceae;PAC001389_g | 1 | 0.0025 |
| PAC001609_g | Bacteria;Firmicutes;Clostridia;Clostridiales;Mogibacterium_f;PAC001609_g | 1 | 0.0025 |
| PAC002042_g | Bacteria;Firmicutes;Clostridia;Clostridiales;Lachnospiraceae;PAC002042_g | 1 | 0.0025 |
| PAC002178_g | Bacteria;Firmicutes;Clostridia;Clostridiales;Lachnospiraceae;PAC002178_g | 1 | 0.0025 |

**Set 2_0%_S: Day 10_heme-SCP 0%_Species**

| Taxon name | Taxonomy | Count | Proportion(%) |
|---|---|---|---|
| Bacteroides vulgatus | Bacteria;Bacteroidetes;Bacteroidia;Bacteroidales;Bacteroidaceae;Bacteroides;Bacteroides vulgatus | 6372 | 18.0648 |
| Bacteroides HM124113_s | Bacteria;Bacteroidetes;Bacteroidia;Bacteroidales;Bacteroidaceae;Bacteroides;HM124113_s | 4628 | 13.1205 |
| KE159600_g KE159600_s | Bacteria;Firmicutes;Clostridia;Clostridiales;Lachnospiraceae;KE159600_g;KE159600_s | 1831 | 5.1909 |
| Akkermansia muciniphila | Bacteria;Verrucomicrobia;Verrucomicrobiae;Verrucomicrobiales;Akkermansiaceae;Akkermansia;Akkermansia muciniphila | 1607 | 4.5559 |
| Bilophila wadsworthia | Bacteria;Proteobacteria;Deltaproteobacteria;Desulfovibrionales;Desulfovibrionaceae;Bilophila;Bilophila wadsworthia | 1346 | 3.8159 |
| Bacteroides acidifaciens | Bacteria;Bacteroidetes;Bacteroidia;Bacteroidales;Bacteroidaceae;Bacteroides;Bacteroides acidifaciens | 1344 | 3.8103 |
| PAC002482_g EF603735_s | Bacteria;Bacteroidetes;Bacteroidia;Bacteroidales;AC160630_f;PAC002482_g;EF603735_s | 1184 | 3.3567 |
| Lactococcus lactis group | Bacteria;Firmicutes;Bacilli;Lactobacillales;Streptococcaceae;Lactococcus;Lactococcus lactis group | 1076 | 3.0505 |
| Bacteroides_uc | Bacteria;Bacteroidetes;Bacteroidia;Bacteroidales;Bacteroidaceae;Bacteroides;Bacteroides_uc | 1054 | 2.9881 |
| KE159571_g PAC002009_s group | Bacteria;Firmicutes;Clostridia;Clostridiales;Lachnospiraceae;KE159571_g;PAC002009_s group | 937 | 2.6564 |
| Alistipes PAC001061_s | Bacteria;Bacteroidetes;Bacteroidia;Bacteroidales;Rikenellaceae;Alistipes;PAC001061_s | 772 | 2.1886 |
| PAC001074_g PAC001074_s | Bacteria;Bacteroidetes;Bacteroidia;Bacteroidales;Muribaculaceae;PAC001074_g;PAC001074_s | 648 | 1.8371 |
| Eubacterium_g23 PAC001121_s group | Bacteria;Firmicutes;Clostridia;Clostridiales;Ruminococcaceae;Eubacterium_g23;PAC001121_s group | 597 | 1.6925 |
| Flintibacter butyricus group | Bacteria;Firmicutes;Clostridia;Clostridiales;Ruminococcaceae;Pseudoflavonifractor;Flintibacter butyricus group | 593 | 1.6812 |
| Oscillibacter PAC001501_s | Bacteria;Firmicutes;Clostridia;Clostridiales;Ruminococcaceae;Oscillibacter;PAC001501_s | 553 | 1.5678 |
| PAC001141_g PAC001141_s | Bacteria;Firmicutes;Clostridia;Clostridiales;Christensenellaceae;PAC001141_g;PAC001141_s | 542 | 1.5366 |
| Lactobacillus gasseri group | Bacteria;Firmicutes;Bacilli;Lactobacillales;Lactobacillaceae;Lactobacillus;Lactobacillus gasseri group | 455 | 1.2899 |
| PAC001402_g PAC001764_s | Bacteria;Firmicutes;Clostridia;Clostridiales;Ruminococcaceae;PAC001402_g;PAC001764_s | 423 | 1.1992 |
| Anaerotignum PAC001740_s | Bacteria;Firmicutes;Clostridia;Clostridiales;Lachnospiraceae;Anaerotignum;PAC001740_s | 382 | 1.083 |
| PAC001516_g PAC001741_s | Bacteria;Firmicutes;Clostridia;Clostridiales;Lachnospiraceae;PAC001516_g;PAC001741_s | 339 | 0.9611 |
| Clostridium cocleatum | Bacteria;Firmicutes;Erysipelotrichi;Erysipelotrichales;Erysipelotrichaceae;Clostridium_g6;Clostridium cocleatum | 332 | 0.9412 |
| Pseudoflavonifractor PAC001379_s | Bacteria;Firmicutes;Clostridia;Clostridiales;Ruminococcaceae;Pseudoflavonifractor;PAC001379_s | 312 | 0.8845 |
| PAC000664_g PAC001104_s | Bacteria;Firmicutes;Clostridia;Clostridiales;Lachnospiraceae;PAC000664_g;PAC001104_s | 271 | 0.7683 |
| PAC001516_g PAC002384_s | Bacteria;Firmicutes;Clostridia;Clostridiales;Lachnospiraceae;PAC001516_g;PAC002384_s | 251 | 0.7116 |
| Bacteroides stercorirosoris | Bacteria;Bacteroidetes;Bacteroidia;Bacteroidales;Bacteroidaceae;Bacteroides;Bacteroides stercorirosoris | 219 | 0.6209 |
| KE159605_g KE159605_s | Bacteria;Firmicutes;Clostridia;Clostridiales;Lachnospiraceae;KE159605_g;KE159605_s | 194 | 0.55 |
| PAC001165_g PAC001384_s | Bacteria;Firmicutes;Clostridia;Clostridiales;Lachnospiraceae;PAC001165_g;PAC001384_s | 184 | 0.5216 |
| Lactobacillus murinus group | Bacteria;Firmicutes;Bacilli;Lactobacillales;Lactobacillaceae;Lactobacillus;Lactobacillus murinus group | 181 | 0.5131 |
| Oscillibacter PAC001186_s group | Bacteria;Firmicutes;Clostridia;Clostridiales;Ruminococcaceae;Oscillibacter;PAC001186_s group | 178 | 0.5046 |
| Lactobacillus reuteri group | Bacteria;Firmicutes;Bacilli;Lactobacillales;Lactobacillaceae;Lactobacillus;Lactobacillus reuteri group | 172 | 0.4876 |
| KE159797_g_uc | Bacteria;Firmicutes;Clostridia;Clostridiales;Lachnospiraceae;KE159797_g;KE159797_g_uc | 168 | 0.4763 |
| PAC002375_g PAC002375_s | Bacteria;Firmicutes;Clostridia;Clostridiales;Lachnospiraceae;PAC002375_g;PAC002375_s | 157 | 0.4451 |
| PAC001068_g PAC001068_s | Bacteria;Bacteroidetes;Bacteroidia;Bacteroidales;Muribaculaceae;PAC001068_g;PAC001068_s | 155 | 0.4394 |
| PAC001201_g PAC001201_s group | Bacteria;Firmicutes;Clostridia;Clostridiales;Lachnospiraceae;PAC001201_g;PAC001201_s group | 155 | 0.4394 |
| Pseudoflavonifractor PAC001543_s | Bacteria;Firmicutes;Clostridia;Clostridiales;Ruminococcaceae;Pseudoflavonifractor;PAC001543_s | 154 | 0.4366 |
| Pseudoflavonifractor PAC001087_s | Bacteria;Firmicutes;Clostridia;Clostridiales;Ruminococcaceae;Pseudoflavonifractor;PAC001087_s | 143 | 0.4054 |
| PAC001112_g PAC001267_s | Bacteria;Bacteroidetes;Bacteroidia;Bacteroidales;Muribaculaceae;PAC001112_g;PAC001267_s | 140 | 0.3969 |
| Pseudoflavonifractor PAC001550_s | Bacteria;Firmicutes;Clostridia;Clostridiales;Ruminococcaceae;Pseudoflavonifractor;PAC001550_s | 131 | 0.3714 |
| Anaerotruncus KE159677_s | Bacteria;Firmicutes;Clostridia;Clostridiales;Ruminococcaceae;Anaerotruncus;KE159677_s | 129 | 0.3657 |
| PAC001066_g PAC001066_s | Bacteria;Bacteroidetes;Bacteroidia;Bacteroidales;Muribaculaceae;PAC001066_g;PAC001066_s | 125 | 0.3544 |
| Pseudoflavonifractor PAC001574_s group | Bacteria;Firmicutes;Clostridia;Clostridiales;Ruminococcaceae;Pseudoflavonifractor;PAC001574_s group | 124 | 0.3515 |
| Acetatifactor PAC001801_s | Bacteria;Firmicutes;Clostridia;Clostridiales;Lachnospiraceae;Acetatifactor;PAC001801_s | 109 | 0.309 |
| Parasutterella PAC002476_s | Bacteria;Proteobacteria;Betaproteobacteria;Burkholderiales;Sutterellaceae;Parasutterella;PAC002476_s | 107 | 0.3033 |
| PAC001221_g PAC001221_s | Bacteria;Firmicutes;Clostridia;Clostridiales;Dehalobacterium_f;PAC001221_g;PAC001221_s | 104 | 0.2948 |
| Clostridium_g21 EU506235_s | Bacteria;Firmicutes;Clostridia;Clostridiales;Lachnospiraceae;Clostridium_g21;EU506235_s | 102 | 0.2892 |
| Frisingicoccus PAC001744_s group | Bacteria;Firmicutes;Clostridia;Clostridiales;Lachnospiraceae;Frisingicoccus;PAC001744_s group | 96 | 0.2722 |
| PAC001063_g PAC001063_s | Bacteria;Bacteroidetes;Bacteroidia;Bacteroidales;Muribaculaceae;PAC001063_g;PAC001063_s | 91 | 0.258 |
| PAC001778_g PAC001778_s | Bacteria;Firmicutes;Clostridia;Clostridiales;Ruminococcaceae;PAC001778_g;PAC001778_s | 91 | 0.258 |
| Bacteroides uniformis | Bacteria;Bacteroidetes;Bacteroidia;Bacteroidales;Bacteroidaceae;Bacteroides;Bacteroides uniformis | 89 | 0.2523 |
| Oscillibacter PAC001188_s | Bacteria;Firmicutes;Clostridia;Clostridiales;Ruminococcaceae;Oscillibacter;PAC001188_s | 86 | 0.2438 |
| Pseudoflavonifractor PAC001549_s | Bacteria;Firmicutes;Clostridia;Clostridiales;Ruminococcaceae;Pseudoflavonifractor;PAC001549_s | 84 | 0.2381 |
| PAC000198_g PAC001075_s | Bacteria;Bacteroidetes;Bacteroidia;Bacteroidales;Muribaculaceae;PAC000198_g;PAC001075_s | 81 | 0.2296 |
| Clostridium_g21 PAC001085_s | Bacteria;Firmicutes;Clostridia;Clostridiales;Lachnospiraceae;Clostridium_g21;PAC001085_s | 78 | 0.2211 |
| Pseudoflavonifractor FJ879979_s | Bacteria;Firmicutes;Clostridia;Clostridiales;Ruminococcaceae;Pseudoflavonifractor;FJ879979_s | 74 | 0.2098 |
| Oscillibacter PAC002372_s | Bacteria;Firmicutes;Clostridia;Clostridiales;Ruminococcaceae;Oscillibacter;PAC002372_s | 74 | 0.2098 |
| PAC001400_g PAC001400_s | Bacteria;Firmicutes;Clostridia;Clostridiales;Peptococcaceae;PAC001400_g;PAC001400_s | 73 | 0.207 |
| Clostridium_g21 KE159781_s | Bacteria;Firmicutes;Clostridia;Clostridiales;Lachnospiraceae;Clostridium_g21;KE159781_s | 72 | 0.2041 |
| Oscillibacter PAC001584_s group | Bacteria;Firmicutes;Clostridia;Clostridiales;Ruminococcaceae;Oscillibacter;PAC001584_s group | 72 | 0.2041 |
| Clostridium_g21_uc | Bacteria;Firmicutes;Clostridia;Clostridiales;Lachnospiraceae;Clostridium_g21;Clostridium_g21_uc | 70 | 0.1985 |
| Pseudoflavonifractor KI535319_s | Bacteria;Firmicutes;Clostridia;Clostridiales;Ruminococcaceae;Pseudoflavonifractor;KI535319_s | 68 | 0.1928 |
| Oscillibacter PAC001502_s | Bacteria;Firmicutes;Clostridia;Clostridiales;Ruminococcaceae;Oscillibacter;PAC001502_s | 68 | 0.1928 |
| PAC001544_g PAC001544_s | Bacteria;Firmicutes;Clostridia;Clostridiales;Lachnospiraceae;PAC001544_g;PAC001544_s | 68 | 0.1928 |
| KE159538_g PAC001708_s | Bacteria;Firmicutes;Clostridia;Clostridiales;Lachnospiraceae;KE159538_g;PAC001708_s | 65 | 0.1843 |
| PAC001402_g PAC001748_s | Bacteria;Firmicutes;Clostridia;Clostridiales;Ruminococcaceae;PAC001402_g;PAC001748_s | 65 | 0.1843 |
| Lachnospiraceae_uc | Bacteria;Firmicutes;Clostridia;Clostridiales;Lachnospiraceae;Lachnospiraceae_uc | 58 | 0.1644 |
| Enterorhabdus caecimuris | Bacteria;Actinobacteria;Coriobacteriia;Coriobacteriales;Coriobacteriaceae;Enterorhabdus;Enterorhabdus caecimuris | 57 | 0.1616 |
| Escherichia coli group | Bacteria;Proteobacteria;Gammaproteobacteria;Enterobacterales;Enterobacteriaceae;Escherichia;Escherichia coli group | 57 | 0.1616 |
| Pseudoflavonifractor_uc | Bacteria;Firmicutes;Clostridia;Clostridiales;Ruminococcaceae;Pseudoflavonifractor;Pseudoflavonifractor_uc | 56 | 0.1588 |
| PAC001092_g PAC001774_s | Bacteria;Firmicutes;Clostridia;Clostridiales;Lachnospiraceae;PAC001092_g;PAC001774_s | 54 | 0.1531 |
| Sporobacter PAC001659_s | Bacteria;Firmicutes;Clostridia;Clostridiales;Ruminococcaceae;Sporobacter;PAC001659_s | 52 | 0.1474 |
| PAC001385_g PAC001727_s group | Bacteria;Firmicutes;Clostridia;Clostridiales;Lachnospiraceae;PAC001385_g;PAC001727_s group | 50 | 0.1418 |
| Acetatifactor PAC001773_s | Bacteria;Firmicutes;Clostridia;Clostridiales;Lachnospiraceae;Acetatifactor;PAC001773_s | 50 | 0.1418 |
| PAC001043_g PAC002511_s group | Bacteria;Firmicutes;Clostridia;Clostridiales;Lachnospiraceae;PAC001043_g;PAC002511_s group | 49 | 0.1389 |
| Clostridium_g21 PAC002512_s group | Bacteria;Firmicutes;Clostridia;Clostridiales;Lachnospiraceae;Clostridium_g21;PAC002512_s group | 49 | 0.1389 |
| PAC000748_g PAC001099_s | Bacteria;Firmicutes;Clostridia;Clostridiales;Ruminococcaceae;PAC000748_g;PAC001099_s | 47 | 0.1332 |
| Paludicola PAC001784_s | Bacteria;Firmicutes;Clostridia;Clostridiales;Ruminococcaceae;Paludicola;PAC001784_s | 46 | 0.1304 |
| Oscillibacter PAC001156_s | Bacteria;Firmicutes;Clostridia;Clostridiales;Ruminococcaceae;Oscillibacter;PAC001156_s | 43 | 0.1219 |
| PAC001103_g_uc | Bacteria;Firmicutes;Clostridia;Clostridiales;Lachnospiraceae;PAC001103_g;PAC001103_g_uc | 40 | 0.1134 |
| Desulfovibrio PAC001079_s | Bacteria;Proteobacteria;Deltaproteobacteria;Desulfovibrionales;Desulfovibrionaceae;Desulfovibrio;PAC001079_s | 39 | 0.1106 |
| PAC001138_g PAC001518_s | Bacteria;Firmicutes;Clostridia;Clostridiales;Lachnospiraceae;PAC001138_g;PAC001518_s | 39 | 0.1106 |
| Gemella palaticanis | Bacteria;Firmicutes;Bacilli;Bacillales;Gemella_f;Gemella;Gemella palaticanis | 37 | 0.1049 |
| Pseudoflavonifractor EF602939_s | Bacteria;Firmicutes;Clostridia;Clostridiales;Ruminococcaceae;Pseudoflavonifractor;EF602939_s | 37 | 0.1049 |
| KE159538_g_uc | Bacteria;Firmicutes;Clostridia;Clostridiales;Lachnospiraceae;KE159538_g;KE159538_g_uc | 37 | 0.1049 |
| Caproiciproducens EU622658_s | Bacteria;Firmicutes;Clostridia;Clostridiales;Ruminococcaceae;Caproiciproducens;EU622658_s | 36 | 0.1021 |
| PAC000186_g PAC001064_s | Bacteria;Bacteroidetes;Bacteroidia;Bacteroidales;Muribaculaceae;PAC000186_g;PAC001064_s | 36 | 0.1021 |
| Sporobacter PAC001096_s | Bacteria;Firmicutes;Clostridia;Clostridiales;Ruminococcaceae;Sporobacter;PAC001096_s | 36 | 0.1021 |
| KE159571_g PAC002035_s | Bacteria;Firmicutes;Clostridia;Clostridiales;Lachnospiraceae;KE159571_g;PAC002035_s | 36 | 0.1021 |
| KE159797_g KE159797_s | Bacteria;Firmicutes;Clostridia;Clostridiales;Lachnospiraceae;KE159797_g;KE159797_s | 35 | 0.0992 |
| PAC001200_g PAC001554_s | Bacteria;Firmicutes;Clostridia;Clostridiales;Lachnospiraceae;PAC001200_g;PAC001554_s | 35 | 0.0992 |
| Oscillibacter PAC001369_s | Bacteria;Firmicutes;Clostridia;Clostridiales;Ruminococcaceae;Oscillibacter;PAC001369_s | 34 | 0.0964 |
| Bacteroides DQ805799_s | Bacteria;Bacteroidetes;Bacteroidia;Bacteroidales;Bacteroidaceae;Bacteroides;DQ805799_s | 33 | 0.0936 |
| PAC001287_g PAC001287_s | Bacteria;Firmicutes;Clostridia;Clostridiales;Lachnospiraceae;PAC001287_g;PAC001287_s | 31 | 0.0879 |
| Alistipes finegoldii | Bacteria;Bacteroidetes;Bacteroidia;Bacteroidales;Rikenellaceae;Alistipes;Alistipes finegoldii | 30 | 0.0851 |
| Pseudoflavonifractor PAC001786_s | Bacteria;Firmicutes;Clostridia;Clostridiales;Ruminococcaceae;Pseudoflavonifractor;PAC001786_s | 28 | 0.0794 |
| Bilophila_uc | Bacteria;Proteobacteria;Deltaproteobacteria;Desulfovibrionales;Desulfovibrionaceae;Bilophila;Bilophila_uc | 28 | 0.0794 |
| PAC000671_g PAC001473_s | Bacteria;Firmicutes;Clostridia;Clostridiales;Lachnospiraceae;PAC000671_g;PAC001473_s | 27 | 0.0765 |
| Caproiciproducens PAC001505_s | Bacteria;Firmicutes;Clostridia;Clostridiales;Ruminococcaceae;Caproiciproducens;PAC001505_s | 27 | 0.0765 |
| PAC001457_g PAC001495_s group | Bacteria;Firmicutes;Clostridia;Clostridiales;Lachnospiraceae;PAC001457_g;PAC001495_s group | 26 | 0.0737 |
| KE159538_g EF097676_s | Bacteria;Firmicutes;Clostridia;Clostridiales;Lachnospiraceae;KE159538_g;EF097676_s | 25 | 0.0709 |
| PAC001528_g PAC001528_s | Bacteria;Firmicutes;Erysipelotrichi;Erysipelotrichales;Erysipelotrichaceae;PAC001528_g;PAC001528_s | 25 | 0.0709 |
| Bacteroides intestinalis | Bacteria;Bacteroidetes;Bacteroidia;Bacteroidales;Bacteroidaceae;Bacteroides;Bacteroides intestinalis | 24 | 0.068 |
| Mucispirillum schaedleri | Bacteria;Deferribacteres;Deferribacteres_c;Deferribacterales;Deferribacteraceae;Mucispirillum;Mucispirillum schaedleri | 24 | 0.068 |
| Emergencia PAC001235_s | Bacteria;Firmicutes;Clostridia;Clostridiales;Mogibacterium_f;Emergencia;PAC001235_s | 24 | 0.068 |
| PAC001313_g PAC001313_s | Bacteria;Firmicutes;Clostridia;Clostridiales;Ruminococcaceae;PAC001313_g;PAC001313_s | 22 | 0.0624 |
| PAC000197_g_uc | Bacteria;Tenericutes;Mollicutes;PAC001057_o;PAC000197_f;PAC000197_g;PAC000197_g_uc | 22 | 0.0624 |
| PAC001149_g PAC001511_s | Bacteria;Firmicutes;Clostridia;Clostridiales;Lachnospiraceae;PAC001149_g;PAC001511_s | 21 | 0.0595 |
| Eubacterium_g6 PAC001586_s | Bacteria;Firmicutes;Clostridia;Clostridiales;Lachnospiraceae;Eubacterium_g6;PAC001586_s | 21 | 0.0595 |
| Clostridium_g21 PAC001294_s | Bacteria;Firmicutes;Clostridia;Clostridiales;Lachnospiraceae;Clostridium_g21;PAC001294_s | 20 | 0.0567 |
| PAC001402_g PAC001561_s | Bacteria;Firmicutes;Clostridia;Clostridiales;Ruminococcaceae;PAC001402_g;PAC001561_s | 20 | 0.0567 |
| PAC002314_g PAC002314_s | Bacteria;Firmicutes;Erysipelotrichi;Erysipelotrichales;Erysipelotrichaceae;PAC002314_g;PAC002314_s | 20 | 0.0567 |
| KE159600_g EU511112_s | Bacteria;Firmicutes;Clostridia;Clostridiales;Lachnospiraceae;KE159600_g;EU511112_s | 19 | 0.0539 |
| KE159571_g PAC001555_s | Bacteria;Firmicutes;Clostridia;Clostridiales;Lachnospiraceae;KE159571_g;PAC001555_s | 19 | 0.0539 |
| PAC002482_g_uc | Bacteria;Bacteroidetes;Bacteroidia;Bacteroidales;AC160630_f;PAC002482_g;PAC002482_g_uc | 19 | 0.0539 |
| PAC000664_g AB702805_s | Bacteria;Firmicutes;Clostridia;Clostridiales;Lachnospiraceae;PAC000664_g;AB702805_s | 18 | 0.051 |
| Frisingicoccus EF409953_s | Bacteria;Firmicutes;Clostridia;Clostridiales;Lachnospiraceae;Frisingicoccus;EF409953_s | 18 | 0.051 |
| Massilioclostridium PAC001521_s | Bacteria;Firmicutes;Clostridia;Clostridiales;Ruminococcaceae;Massilioclostridium;PAC001521_s | 18 | 0.051 |
| Agathobaculum PAC001688_s | Bacteria;Firmicutes;Clostridia;Clostridiales;Ruminococcaceae;Agathobaculum;PAC001688_s | 18 | 0.051 |
| Emergencia PAC001931_s | Bacteria;Firmicutes;Clostridia;Clostridiales;Mogibacterium_f;Emergencia;PAC001931_s | 18 | 0.051 |
| Clostridium_g21 PAC001757_s group | Bacteria;Firmicutes;Clostridia;Clostridiales;Lachnospiraceae;Clostridium_g21;PAC001757_s group | 17 | 0.0482 |
| Oscillibacter PAC002110_s | Bacteria;Firmicutes;Clostridia;Clostridiales;Ruminococcaceae;Oscillibacter;PAC002110_s | 17 | 0.0482 |
| Harryflintia PAC002149_s | Bacteria;Firmicutes;Clostridia;Clostridiales;Ruminococcaceae;Harryflintia;PAC002149_s | 17 | 0.0482 |
| Anaerotruncus colihominis | Bacteria;Firmicutes;Clostridia;Clostridiales;Ruminococcaceae;Anaerotruncus;Anaerotruncus colihominis | 16 | 0.0454 |
| KE159797_g PAC001222_s | Bacteria;Firmicutes;Clostridia;Clostridiales;Lachnospiraceae;KE159797_g;PAC001222_s | 16 | 0.0454 |
| Frisingicoccus PAC001399_s | Bacteria;Firmicutes;Clostridia;Clostridiales;Lachnospiraceae;Frisingicoccus;PAC001399_s | 16 | 0.0454 |
| Oscillibacter PAC001522_s | Bacteria;Firmicutes;Clostridia;Clostridiales;Ruminococcaceae;Oscillibacter;PAC001522_s | 16 | 0.0454 |
| KE993550_g PAC002377_s | Bacteria;Firmicutes;Clostridia;Clostridiales;Ruminococcaceae;KE993550_g;PAC002377_s | 16 | 0.0454 |
| PAC002390_g PAC002390_s | Bacteria;Firmicutes;Clostridia;Clostridiales;Dehalobacterium_f;PAC002390_g;PAC002390_s | 16 | 0.0454 |
| Bacteroides oleiciplenus | Bacteria;Bacteroidetes;Bacteroidia;Bacteroidales;Bacteroidaceae;Bacteroides;Bacteroides oleiciplenus | 15 | 0.0425 |
| PAC001144_g PAC001593_s | Bacteria;Firmicutes;Clostridia;Clostridiales;Ruminococcaceae;PAC001144_g;PAC001593_s | 15 | 0.0425 |
| Pseudoflavonifractor PAC002302_s | Bacteria;Firmicutes;Clostridia;Clostridiales;Ruminococcaceae;Pseudoflavonifractor;PAC002302_s | 15 | 0.0425 |
| Acetatifactor muris | Bacteria;Firmicutes;Clostridia;Clostridiales;Lachnospiraceae;Acetatifactor;Acetatifactor muris | 14 | 0.0397 |
| Parabacteroides goldsteinii | Bacteria;Bacteroidetes;Bacteroidia;Bacteroidales;Porphyromonadaceae;Parabacteroides;Parabacteroides goldsteinii | 14 | 0.0397 |
| PAC001524_g PAC001524_s | Bacteria;Firmicutes;Clostridia;Clostridiales;Lachnospiraceae;PAC001524_g;PAC001524_s | 14 | 0.0397 |
| PAC001573_g PAC001808_s | Bacteria;Firmicutes;Clostridia;Clostridiales;Mogibacterium_f;PAC001573_g;PAC001808_s | 14 | 0.0397 |
| Clostridium methylpentosum | Bacteria;Firmicutes;Clostridia;Clostridiales;Ruminococcaceae;Massilioclostridium;Clostridium methylpentosum | 13 | 0.0369 |
| Frisingicoccus PAC001728_s group | Bacteria;Firmicutes;Clostridia;Clostridiales;Lachnospiraceae;Frisingicoccus;PAC001728_s group | 13 | 0.0369 |
| PAC000664_g PAC001769_s | Bacteria;Firmicutes;Clostridia;Clostridiales;Lachnospiraceae;PAC000664_g;PAC001769_s | 13 | 0.0369 |
| Acutalibacter PAC002354_s | Bacteria;Firmicutes;Clostridia;Clostridiales;Ruminococcaceae;Acutalibacter;PAC002354_s | 13 | 0.0369 |
| Bacteroides caecimuris | Bacteria;Bacteroidetes;Bacteroidia;Bacteroidales;Bacteroidaceae;Bacteroides;Bacteroides caecimuris | 12 | 0.034 |
| Eubacterium_g17 PAC001119_s | Bacteria;Firmicutes;Clostridia;Clostridiales;Lachnospiraceae;Eubacterium_g17;PAC001119_s | 12 | 0.034 |
| Paludicola PAC001507_s | Bacteria;Firmicutes;Clostridia;Clostridiales;Ruminococcaceae;Paludicola;PAC001507_s | 12 | 0.034 |
| Acetatifactor_uc | Bacteria;Firmicutes;Clostridia;Clostridiales;Lachnospiraceae;Acetatifactor;Acetatifactor_uc | 12 | 0.034 |
| Enterorhabdus mucosicola | Bacteria;Actinobacteria;Coriobacteriia;Coriobacteriales;Coriobacteriaceae;Enterorhabdus;Enterorhabdus mucosicola | 11 | 0.0312 |
| Bacteroides DQ799557_s | Bacteria;Bacteroidetes;Bacteroidia;Bacteroidales;Bacteroidaceae;Bacteroides;DQ799557_s | 11 | 0.0312 |
| PAC001308_g DQ815557_s | Bacteria;Firmicutes;Clostridia;Clostridiales;Lachnospiraceae;PAC001308_g;DQ815557_s | 11 | 0.0312 |
| KE159605_g KB822463_s | Bacteria;Firmicutes;Clostridia;Clostridiales;Lachnospiraceae;KE159605_g;KB822463_s | 11 | 0.0312 |
| Oscillibacter KE159714_s group | Bacteria;Firmicutes;Clostridia;Clostridiales;Ruminococcaceae;Oscillibacter;KE159714_s group | 11 | 0.0312 |
| Clostridium_g24 PAC001374_s | Bacteria;Firmicutes;Clostridia;Clostridiales;Lachnospiraceae;Clostridium_g24;PAC001374_s | 11 | 0.0312 |
| PAC000692_g PAC001387_s | Bacteria;Firmicutes;Clostridia;Clostridiales;Lachnospiraceae;PAC000692_g;PAC001387_s | 11 | 0.0312 |
| PAC000661_g PAC001536_s | Bacteria;Firmicutes;Clostridia;Clostridiales;Ruminococcaceae;PAC000661_g;PAC001536_s | 11 | 0.0312 |
| PAC001612_g PAC001612_s | Bacteria;Firmicutes;Clostridia;Clostridiales;Mogibacterium_f;PAC001612_g;PAC001612_s | 11 | 0.0312 |
| Coprococcus_g2 PAC001722_s | Bacteria;Firmicutes;Clostridia;Clostridiales;Lachnospiraceae;Coprococcus_g2;PAC001722_s | 11 | 0.0312 |
| PAC001112_g PAC002441_s | Bacteria;Bacteroidetes;Bacteroidia;Bacteroidales;Muribaculaceae;PAC001112_g;PAC002441_s | 11 | 0.0312 |
| PAC001062_g PAC001062_s | Bacteria;Bacteroidetes;Bacteroidia;Bacteroidales;Muribaculaceae;PAC001062_g;PAC001062_s | 10 | 0.0284 |
| PAC000661_g PAC001095_s | Bacteria;Firmicutes;Clostridia;Clostridiales;Ruminococcaceae;PAC000661_g;PAC001095_s | 10 | 0.0284 |
| Acutalibacter PAC001519_s | Bacteria;Firmicutes;Clostridia;Clostridiales;Ruminococcaceae;Acutalibacter;PAC001519_s | 10 | 0.0284 |
| PAC001599_g PAC001599_s | Bacteria;Firmicutes;Clostridia;Clostridiales;Ruminococcaceae;PAC001599_g;PAC001599_s | 10 | 0.0284 |
| PAC001066_g_uc | Bacteria;Bacteroidetes;Bacteroidia;Bacteroidales;Muribaculaceae;PAC001066_g;PAC001066_g_uc | 10 | 0.0284 |
| Bacteroidaceae_uc | Bacteria;Bacteroidetes;Bacteroidia;Bacteroidales;Bacteroidaceae;Bacteroidaceae_uc | 9 | 0.0255 |
| Streptococcus acidominimus group | Bacteria;Firmicutes;Bacilli;Lactobacillales;Streptococcaceae;Streptococcus;Streptococcus acidominimus group | 9 | 0.0255 |
| Arthromitus AP012202_s group | Bacteria;Firmicutes;Clostridia;Clostridiales;Clostridiaceae;Arthromitus;AP012202_s group | 9 | 0.0255 |
| GQ451199_g EU472543_s | Bacteria;Firmicutes;Clostridia;Clostridiales;Ruminococcaceae;GQ451199_g;EU472543_s | 9 | 0.0255 |
| PAC001092_g PAC001499_s | Bacteria;Firmicutes;Clostridia;Clostridiales;Lachnospiraceae;PAC001092_g;PAC001499_s | 9 | 0.0255 |
| PAC001043_g PAC001552_s | Bacteria;Firmicutes;Clostridia;Clostridiales;Lachnospiraceae;PAC001043_g;PAC001552_s | 9 | 0.0255 |
| PAC001043_g PAC001752_s | Bacteria;Firmicutes;Clostridia;Clostridiales;Lachnospiraceae;PAC001043_g;PAC001752_s | 9 | 0.0255 |
| Clostridium_g21 PAC001772_s | Bacteria;Firmicutes;Clostridia;Clostridiales;Lachnospiraceae;Clostridium_g21;PAC001772_s | 9 | 0.0255 |
| PAC001270_g PAC001899_s group | Bacteria;Firmicutes;Clostridia;Clostridiales;Lachnospiraceae;PAC001270_g;PAC001899_s group | 9 | 0.0255 |
| PAC000664_g_uc | Bacteria;Firmicutes;Clostridia;Clostridiales;Lachnospiraceae;PAC000664_g;PAC000664_g_uc | 9 | 0.0255 |
| Frisingicoccus PAC000688_s | Bacteria;Firmicutes;Clostridia;Clostridiales;Lachnospiraceae;Frisingicoccus;PAC000688_s | 8 | 0.0227 |
| Frisingicoccus PAC000700_s | Bacteria;Firmicutes;Clostridia;Clostridiales;Lachnospiraceae;Frisingicoccus;PAC000700_s | 8 | 0.0227 |
| PAC001225_g PAC001225_s | Bacteria;Firmicutes;Clostridia;Clostridiales;Lachnospiraceae;PAC001225_g;PAC001225_s | 8 | 0.0227 |
| PAC001308_g PAC001308_s | Bacteria;Firmicutes;Clostridia;Clostridiales;Lachnospiraceae;PAC001308_g;PAC001308_s | 8 | 0.0227 |
| PAC001092_g PAC001710_s | Bacteria;Firmicutes;Clostridia;Clostridiales;Lachnospiraceae;PAC001092_g;PAC001710_s | 8 | 0.0227 |
| Paludicola PAC002097_s | Bacteria;Firmicutes;Clostridia;Clostridiales;Ruminococcaceae;Paludicola;PAC002097_s | 8 | 0.0227 |
| Muribaculum DQ815542_s | Bacteria;Bacteroidetes;Bacteroidia;Bacteroidales;Muribaculaceae;Muribaculum;DQ815542_s | 7 | 0.0198 |
| PAC000664_g PAC001083_s | Bacteria;Firmicutes;Clostridia;Clostridiales;Lachnospiraceae;PAC000664_g;PAC001083_s | 7 | 0.0198 |
| Christensenella PAC001140_s | Bacteria;Firmicutes;Clostridia;Clostridiales;Christensenellaceae;Christensenella;PAC001140_s | 7 | 0.0198 |
| PAC001103_g PAC001361_s | Bacteria;Firmicutes;Clostridia;Clostridiales;Lachnospiraceae;PAC001103_g;PAC001361_s | 7 | 0.0198 |
| Marvinbryantia PAC001753_s | Bacteria;Firmicutes;Clostridia;Clostridiales;Lachnospiraceae;Marvinbryantia;PAC001753_s | 7 | 0.0198 |
| PAC002460_g PAC002460_s | Bacteria;Firmicutes;Clostridia;Clostridiales;Lachnospiraceae;PAC002460_g;PAC002460_s | 7 | 0.0198 |
| PAC000661_g_uc | Bacteria;Firmicutes;Clostridia;Clostridiales;Ruminococcaceae;PAC000661_g;PAC000661_g_uc | 7 | 0.0198 |
| PAC001068_g_uc | Bacteria;Bacteroidetes;Bacteroidia;Bacteroidales;Muribaculaceae;PAC001068_g;PAC001068_g_uc | 7 | 0.0198 |
| PAC001219_g_uc | Bacteria;Firmicutes;Clostridia;Clostridiales;Christensenellaceae;PAC001219_g;PAC001219_g_uc | 7 | 0.0198 |
| Acinetobacter pittii group | Bacteria;Proteobacteria;Gammaproteobacteria;Pseudomonadales;Moraxellaceae;Acinetobacter;Acinetobacter pittii group | 6 | 0.017 |
| Clostridium_g21 EU509115_s | Bacteria;Firmicutes;Clostridia;Clostridiales;Lachnospiraceae;Clostridium_g21;EU509115_s | 6 | 0.017 |
| PAC001144_g PAC001144_s | Bacteria;Firmicutes;Clostridia;Clostridiales;Ruminococcaceae;PAC001144_g;PAC001144_s | 6 | 0.017 |
| Eubacterium_g4 PAC001174_s | Bacteria;Firmicutes;Clostridia;Clostridiales;Lachnospiraceae;Eubacterium_g4;PAC001174_s | 6 | 0.017 |
| PAC001372_g PAC001372_s | Bacteria;Firmicutes;Clostridia;Clostridiales;Lachnospiraceae;PAC001372_g;PAC001372_s | 6 | 0.017 |
| Eubacterium_g6 PAC001759_s | Bacteria;Firmicutes;Clostridia;Clostridiales;Lachnospiraceae;Eubacterium_g6;PAC001759_s | 6 | 0.017 |
| Eubacterium_g6 PAC002402_s | Bacteria;Firmicutes;Clostridia;Clostridiales;Lachnospiraceae;Eubacterium_g6;PAC002402_s | 6 | 0.017 |
| PAC001092_g_uc | Bacteria;Firmicutes;Clostridia;Clostridiales;Lachnospiraceae;PAC001092_g;PAC001092_g_uc | 6 | 0.017 |
| Ruminococcaceae_uc | Bacteria;Firmicutes;Clostridia;Clostridiales;Ruminococcaceae;Ruminococcaceae_uc | 5 | 0.0142 |
| Staphylococcus sciuri group | Bacteria;Firmicutes;Bacilli;Bacillales;Staphylococcaceae;Staphylococcus;Staphylococcus sciuri group | 5 | 0.0142 |
| Eubacterium_g23 EU509480_s | Bacteria;Firmicutes;Clostridia;Clostridiales;Ruminococcaceae;Eubacterium_g23;EU509480_s | 5 | 0.0142 |
| PAC000748_g PAC001412_s | Bacteria;Firmicutes;Clostridia;Clostridiales;Ruminococcaceae;PAC000748_g;PAC001412_s | 5 | 0.0142 |
| Agathobaculum PAC001476_s | Bacteria;Firmicutes;Clostridia;Clostridiales;Ruminococcaceae;Agathobaculum;PAC001476_s | 5 | 0.0142 |
| PAC001092_g PAC001587_s | Bacteria;Firmicutes;Clostridia;Clostridiales;Lachnospiraceae;PAC001092_g;PAC001587_s | 5 | 0.0142 |
| PAC001092_g PAC001742_s | Bacteria;Firmicutes;Clostridia;Clostridiales;Lachnospiraceae;PAC001092_g;PAC001742_s | 5 | 0.0142 |
| Eubacterium plexicaudatum | Bacteria;Firmicutes;Clostridia;Clostridiales;Lachnospiraceae;Eubacterium_g6;Eubacterium plexicaudatum | 4 | 0.0113 |
| Pseudoflavonifractor ADDX_s | Bacteria;Firmicutes;Clostridia;Clostridiales;Ruminococcaceae;Pseudoflavonifractor;ADDX_s | 4 | 0.0113 |
| Christensenella EU508399_s | Bacteria;Firmicutes;Clostridia;Clostridiales;Christensenellaceae;Christensenella;EU508399_s | 4 | 0.0113 |
| HM124077_g HM124077_s | Bacteria;Bacteroidetes;Bacteroidia;Bacteroidales;Muribaculaceae;HM124077_g;HM124077_s | 4 | 0.0113 |
| Coprobacillus HQ716342_s | Bacteria;Firmicutes;Erysipelotrichi;Erysipelotrichales;Erysipelotrichaceae;Coprobacillus;HQ716342_s | 4 | 0.0113 |
| HQ765927_g HQ765927_s | Bacteria;Firmicutes;Clostridia;Clostridiales;Lachnospiraceae;HQ765927_g;HQ765927_s | 4 | 0.0113 |
| Bacteroides JN559675_s | Bacteria;Bacteroidetes;Bacteroidia;Bacteroidales;Bacteroidaceae;Bacteroides;JN559675_s | 4 | 0.0113 |
| PAC000692_g PAC000692_s | Bacteria;Firmicutes;Clostridia;Clostridiales;Lachnospiraceae;PAC000692_g;PAC000692_s | 4 | 0.0113 |
| PAC000197_g PAC001123_s | Bacteria;Tenericutes;Mollicutes;PAC001057_o;PAC000197_f;PAC000197_g;PAC001123_s | 4 | 0.0113 |
| PAC000748_g PAC001489_s | Bacteria;Firmicutes;Clostridia;Clostridiales;Ruminococcaceae;PAC000748_g;PAC001489_s | 4 | 0.0113 |
| PAC001516_g PAC001516_s | Bacteria;Firmicutes;Clostridia;Clostridiales;Lachnospiraceae;PAC001516_g;PAC001516_s | 4 | 0.0113 |
| PAC001092_g PAC001668_s | Bacteria;Firmicutes;Clostridia;Clostridiales;Lachnospiraceae;PAC001092_g;PAC001668_s | 4 | 0.0113 |
| KE159571_g PAC001705_s | Bacteria;Firmicutes;Clostridia;Clostridiales;Lachnospiraceae;KE159571_g;PAC001705_s | 4 | 0.0113 |
| PAC001103_g PAC001724_s group | Bacteria;Firmicutes;Clostridia;Clostridiales;Lachnospiraceae;PAC001103_g;PAC001724_s group | 4 | 0.0113 |
| PAC000692_g PAC001745_s | Bacteria;Firmicutes;Clostridia;Clostridiales;Lachnospiraceae;PAC000692_g;PAC001745_s | 4 | 0.0113 |
| Christensenella PAC002165_s | Bacteria;Firmicutes;Clostridia;Clostridiales;Christensenellaceae;Christensenella;PAC002165_s | 4 | 0.0113 |
| Oscillibacter_uc | Bacteria;Firmicutes;Clostridia;Clostridiales;Ruminococcaceae;Oscillibacter;Oscillibacter_uc | 4 | 0.0113 |
| Clostridium_g35_uc | Bacteria;Firmicutes;Clostridia;Clostridiales;Lachnospiraceae;Clostridium_g35;Clostridium_g35_uc | 4 | 0.0113 |
| Erysipelotrichaceae_uc | Bacteria;Firmicutes;Erysipelotrichi;Erysipelotrichales;Erysipelotrichaceae;Erysipelotrichaceae_uc | 3 | 0.0085 |
| Blautia wexlerae | Bacteria;Firmicutes;Clostridia;Clostridiales;Lachnospiraceae;Blautia;Blautia wexlerae | 3 | 0.0085 |
| Clostridium saccharogumia group | Bacteria;Firmicutes;Erysipelotrichi;Erysipelotrichales;Erysipelotrichaceae;Clostridium_g6;Clostridium saccharogumia group | 3 | 0.0085 |
| Enterococcus faecalis | Bacteria;Firmicutes;Bacilli;Lactobacillales;Enterococcaceae;Enterococcus;Enterococcus faecalis | 3 | 0.0085 |
| Enterorhabdus muris | Bacteria;Actinobacteria;Coriobacteriia;Coriobacteriales;Coriobacteriaceae;Enterorhabdus;Enterorhabdus muris | 3 | 0.0085 |
| Acutalibacter AB622826_s | Bacteria;Firmicutes;Clostridia;Clostridiales;Ruminococcaceae;Acutalibacter;AB622826_s | 3 | 0.0085 |
| PAC000664_g AB626958_s | Bacteria;Firmicutes;Clostridia;Clostridiales;Lachnospiraceae;PAC000664_g;AB626958_s | 3 | 0.0085 |
| KE159600_g EF098562_s | Bacteria;Firmicutes;Clostridia;Clostridiales;Lachnospiraceae;KE159600_g;EF098562_s | 3 | 0.0085 |
| PAC000196_g EU466069_s | Bacteria;Firmicutes;Clostridia;Clostridiales;Lachnospiraceae;PAC000196_g;EU466069_s | 3 | 0.0085 |
| PAC001219_g HM124151_s | Bacteria;Firmicutes;Clostridia;Clostridiales;Christensenellaceae;PAC001219_g;HM124151_s | 3 | 0.0085 |
| PAC000186_g PAC001065_s | Bacteria;Bacteroidetes;Bacteroidia;Bacteroidales;Muribaculaceae;PAC000186_g;PAC001065_s | 3 | 0.0085 |
| PAC001266_g PAC001266_s | Bacteria;Actinobacteria;Coriobacteriia;Coriobacteriales;Coriobacteriaceae;PAC001266_g;PAC001266_s | 3 | 0.0085 |
| PAC001323_g PAC001323_s | Bacteria;Tenericutes;Mollicutes;PAC001057_o;PAC000197_f;PAC001323_g;PAC001323_s | 3 | 0.0085 |
| Frisingicoccus PAC001481_s | Bacteria;Firmicutes;Clostridia;Clostridiales;Lachnospiraceae;Frisingicoccus;PAC001481_s | 3 | 0.0085 |
| Pseudoflavonifractor PAC001548_s | Bacteria;Firmicutes;Clostridia;Clostridiales;Ruminococcaceae;Pseudoflavonifractor;PAC001548_s | 3 | 0.0085 |
| PAC000692_g PAC001577_s | Bacteria;Firmicutes;Clostridia;Clostridiales;Lachnospiraceae;PAC000692_g;PAC001577_s | 3 | 0.0085 |
| PAC001588_g PAC001588_s | Bacteria;Firmicutes;Clostridia;Clostridiales;Lachnospiraceae;PAC001588_g;PAC001588_s | 3 | 0.0085 |
| PAC001118_g PAC002344_s | Bacteria;Firmicutes;Clostridia;Clostridiales;Lachnospiraceae;PAC001118_g;PAC002344_s | 3 | 0.0085 |
| Sporobacter PAC002398_s | Bacteria;Firmicutes;Clostridia;Clostridiales;Ruminococcaceae;Sporobacter;PAC002398_s | 3 | 0.0085 |
| PAC001270_g PAC002420_s | Bacteria;Firmicutes;Clostridia;Clostridiales;Lachnospiraceae;PAC001270_g;PAC002420_s | 3 | 0.0085 |
| KE159571_g_uc | Bacteria;Firmicutes;Clostridia;Clostridiales;Lachnospiraceae;KE159571_g;KE159571_g_uc | 3 | 0.0085 |
| PAC001778_g_uc | Bacteria;Firmicutes;Clostridia;Clostridiales;Ruminococcaceae;PAC001778_g;PAC001778_g_uc | 3 | 0.0085 |
| Bacteroides sartorii | Bacteria;Bacteroidetes;Bacteroidia;Bacteroidales;Bacteroidaceae;Bacteroides;Bacteroides sartorii | 2 | 0.0057 |
| Clostridium_g24 EU504811_s | Bacteria;Firmicutes;Clostridia;Clostridiales;Lachnospiraceae;Clostridium_g24;EU504811_s | 2 | 0.0057 |
| PAC001516_g FJ880744_s | Bacteria;Firmicutes;Clostridia;Clostridiales;Lachnospiraceae;PAC001516_g;FJ880744_s | 2 | 0.0057 |
| Oscillibacter FJ881219_s | Bacteria;Firmicutes;Clostridia;Clostridiales;Ruminococcaceae;Oscillibacter;FJ881219_s | 2 | 0.0057 |
| Eubacterium_g6 PAC001227_s | Bacteria;Firmicutes;Clostridia;Clostridiales;Lachnospiraceae;Eubacterium_g6;PAC001227_s | 2 | 0.0057 |
| Acetatifactor PAC001373_s | Bacteria;Firmicutes;Clostridia;Clostridiales;Lachnospiraceae;Acetatifactor;PAC001373_s | 2 | 0.0057 |
| PAC001092_g PAC001381_s | Bacteria;Firmicutes;Clostridia;Clostridiales;Lachnospiraceae;PAC001092_g;PAC001381_s | 2 | 0.0057 |
| Pseudoflavonifractor PAC001542_s | Bacteria;Firmicutes;Clostridia;Clostridiales;Ruminococcaceae;Pseudoflavonifractor;PAC001542_s | 2 | 0.0057 |
| PAC001402_g PAC001546_s | Bacteria;Firmicutes;Clostridia;Clostridiales;Ruminococcaceae;PAC001402_g;PAC001546_s | 2 | 0.0057 |
| Oscillibacter PAC001551_s | Bacteria;Firmicutes;Clostridia;Clostridiales;Ruminococcaceae;Oscillibacter;PAC001551_s | 2 | 0.0057 |
| Anaerotruncus PAC001590_s | Bacteria;Firmicutes;Clostridia;Clostridiales;Ruminococcaceae;Anaerotruncus;PAC001590_s | 2 | 0.0057 |
| Harryflintia PAC001713_s | Bacteria;Firmicutes;Clostridia;Clostridiales;Ruminococcaceae;Harryflintia;PAC001713_s | 2 | 0.0057 |
| Acetatifactor PAC001749_s | Bacteria;Firmicutes;Clostridia;Clostridiales;Lachnospiraceae;Acetatifactor;PAC001749_s | 2 | 0.0057 |
| KE159571_g PAC001776_s group | Bacteria;Firmicutes;Clostridia;Clostridiales;Lachnospiraceae;KE159571_g;PAC001776_s group | 2 | 0.0057 |
| PAC001165_g PAC001796_s | Bacteria;Firmicutes;Clostridia;Clostridiales;Lachnospiraceae;PAC001165_g;PAC001796_s | 2 | 0.0057 |
| PAC002039_g PAC002039_s | Bacteria;Firmicutes;Clostridia;Clostridiales;Lachnospiraceae;PAC002039_g;PAC002039_s | 2 | 0.0057 |
| PAC000661_g PAC002422_s | Bacteria;Firmicutes;Clostridia;Clostridiales;Ruminococcaceae;PAC000661_g;PAC002422_s | 2 | 0.0057 |
| Clostridium_g21 PAC002492_s | Bacteria;Firmicutes;Clostridia;Clostridiales;Lachnospiraceae;Clostridium_g21;PAC002492_s | 2 | 0.0057 |
| Akkermansia_uc | Bacteria;Verrucomicrobia;Verrucomicrobiae;Verrucomicrobiales;Akkermansiaceae;Akkermansia;Akkermansia_uc | 2 | 0.0057 |
| Alistipes_uc | Bacteria;Bacteroidetes;Bacteroidia;Bacteroidales;Rikenellaceae;Alistipes;Alistipes_uc | 2 | 0.0057 |
| Clostridium_g6_uc | Bacteria;Firmicutes;Erysipelotrichi;Erysipelotrichales;Erysipelotrichaceae;Clostridium_g6;Clostridium_g6_uc | 2 | 0.0057 |
| PAC001296_g_uc | Bacteria;Firmicutes;Clostridia;Clostridiales;Lachnospiraceae;PAC001296_g;PAC001296_g_uc | 2 | 0.0057 |
| PAC001516_g_uc | Bacteria;Firmicutes;Clostridia;Clostridiales;Lachnospiraceae;PAC001516_g;PAC001516_g_uc | 2 | 0.0057 |
| Acutalibacter muris | Bacteria;Firmicutes;Clostridia;Clostridiales;Ruminococcaceae;Acutalibacter;Acutalibacter muris | 1 | 0.0028 |
| Alistipes obesi | Bacteria;Bacteroidetes;Bacteroidia;Bacteroidales;Rikenellaceae;Alistipes;Alistipes obesi | 1 | 0.0028 |
| Anaerostipes hadrus group | Bacteria;Firmicutes;Clostridia;Clostridiales;Lachnospiraceae;Anaerostipes;Anaerostipes hadrus group | 1 | 0.0028 |
| Clostridium symbiosum | Bacteria;Firmicutes;Clostridia;Clostridiales;Lachnospiraceae;Clostridium_g35;Clostridium symbiosum | 1 | 0.0028 |
| Eubacterium hallii | Bacteria;Firmicutes;Clostridia;Clostridiales;Lachnospiraceae;Eubacterium_g5;Eubacterium hallii | 1 | 0.0028 |
| Sphingomonas pruni group | Bacteria;Proteobacteria;Alphaproteobacteria;Sphingomonadales;Sphingomonadaceae;Sphingomonas;Sphingomonas pruni group | 1 | 0.0028 |
| LLKB_g AF371672_s | Bacteria;Firmicutes;Clostridia;Clostridiales;Lachnospiraceae;LLKB_g;AF371672_s | 1 | 0.0028 |
| Pseudoflavonifractor EU454100_s | Bacteria;Firmicutes;Clostridia;Clostridiales;Ruminococcaceae;Pseudoflavonifractor;EU454100_s | 1 | 0.0028 |
| Oscillibacter EU454374_s | Bacteria;Firmicutes;Clostridia;Clostridiales;Ruminococcaceae;Oscillibacter;EU454374_s | 1 | 0.0028 |
| Phocea EU457191_s | Bacteria;Firmicutes;Clostridia;Clostridiales;Ruminococcaceae;Phocea;EU457191_s | 1 | 0.0028 |
| Pseudoflavonifractor EU460161_s | Bacteria;Firmicutes;Clostridia;Clostridiales;Ruminococcaceae;Pseudoflavonifractor;EU460161_s | 1 | 0.0028 |
| PAC001165_g EU622677_s | Bacteria;Firmicutes;Clostridia;Clostridiales;Lachnospiraceae;PAC001165_g;EU622677_s | 1 | 0.0028 |
| Agathobaculum FJ510897_s | Bacteria;Firmicutes;Clostridia;Clostridiales;Ruminococcaceae;Agathobaculum;FJ510897_s | 1 | 0.0028 |
| Pseudoflavonifractor FJ879530_s | Bacteria;Firmicutes;Clostridia;Clostridiales;Ruminococcaceae;Pseudoflavonifractor;FJ879530_s | 1 | 0.0028 |
| Pseudoflavonifractor LN866274_s | Bacteria;Firmicutes;Clostridia;Clostridiales;Ruminococcaceae;Pseudoflavonifractor;LN866274_s | 1 | 0.0028 |
| PAC001165_g PAC001165_s | Bacteria;Firmicutes;Clostridia;Clostridiales;Lachnospiraceae;PAC001165_g;PAC001165_s | 1 | 0.0028 |
| PAC001219_g PAC001280_s | Bacteria;Firmicutes;Clostridia;Clostridiales;Christensenellaceae;PAC001219_g;PAC001280_s | 1 | 0.0028 |
| Frisingicoccus PAC001364_s | Bacteria;Firmicutes;Clostridia;Clostridiales;Lachnospiraceae;Frisingicoccus;PAC001364_s | 1 | 0.0028 |
| PAC001389_g PAC001389_s | Bacteria;Actinobacteria;Coriobacteriia;Coriobacteriales;Coriobacteriaceae;PAC001389_g;PAC001389_s | 1 | 0.0028 |
| PAC001402_g PAC001402_s | Bacteria;Firmicutes;Clostridia;Clostridiales;Ruminococcaceae;PAC001402_g;PAC001402_s | 1 | 0.0028 |
| Acetatifactor PAC001463_s | Bacteria;Firmicutes;Clostridia;Clostridiales;Lachnospiraceae;Acetatifactor;PAC001463_s | 1 | 0.0028 |
| Anaerotignum PAC001464_s | Bacteria;Firmicutes;Clostridia;Clostridiales;Lachnospiraceae;Anaerotignum;PAC001464_s | 1 | 0.0028 |
| Eubacterium_g23 PAC001490_s | Bacteria;Firmicutes;Clostridia;Clostridiales;Ruminococcaceae;Eubacterium_g23;PAC001490_s | 1 | 0.0028 |
| PAC001092_g PAC001517_s | Bacteria;Firmicutes;Clostridia;Clostridiales;Lachnospiraceae;PAC001092_g;PAC001517_s | 1 | 0.0028 |
| Oscillibacter PAC001526_s | Bacteria;Firmicutes;Clostridia;Clostridiales;Ruminococcaceae;Oscillibacter;PAC001526_s | 1 | 0.0028 |
| PAC001043_g PAC001557_s | Bacteria;Firmicutes;Clostridia;Clostridiales;Lachnospiraceae;PAC001043_g;PAC001557_s | 1 | 0.0028 |
| PAC000692_g PAC001567_s | Bacteria;Firmicutes;Clostridia;Clostridiales;Lachnospiraceae;PAC000692_g;PAC001567_s | 1 | 0.0028 |
| Acutalibacter PAC001594_s | Bacteria;Firmicutes;Clostridia;Clostridiales;Ruminococcaceae;Acutalibacter;PAC001594_s | 1 | 0.0028 |
| PAC001609_g PAC001650_s | Bacteria;Firmicutes;Clostridia;Clostridiales;Mogibacterium_f;PAC001609_g;PAC001650_s | 1 | 0.0028 |
| PAC000692_g PAC001666_s | Bacteria;Firmicutes;Clostridia;Clostridiales;Lachnospiraceae;PAC000692_g;PAC001666_s | 1 | 0.0028 |
| Pseudoflavonifractor PAC001667_s | Bacteria;Firmicutes;Clostridia;Clostridiales;Ruminococcaceae;Pseudoflavonifractor;PAC001667_s | 1 | 0.0028 |
| Oscillibacter PAC001706_s | Bacteria;Firmicutes;Clostridia;Clostridiales;Ruminococcaceae;Oscillibacter;PAC001706_s | 1 | 0.0028 |
| PAC001092_g PAC001726_s | Bacteria;Firmicutes;Clostridia;Clostridiales;Lachnospiraceae;PAC001092_g;PAC001726_s | 1 | 0.0028 |
| PAC001092_g PAC001811_s | Bacteria;Firmicutes;Clostridia;Clostridiales;Lachnospiraceae;PAC001092_g;PAC001811_s | 1 | 0.0028 |
| PAC001908_g PAC001908_s | Bacteria;Firmicutes;Clostridia;Clostridiales;Ruminococcaceae;PAC001908_g;PAC001908_s | 1 | 0.0028 |
| PAC001778_g PAC001909_s | Bacteria;Firmicutes;Clostridia;Clostridiales;Ruminococcaceae;PAC001778_g;PAC001909_s | 1 | 0.0028 |
| PAC002042_g PAC002042_s | Bacteria;Firmicutes;Clostridia;Clostridiales;Lachnospiraceae;PAC002042_g;PAC002042_s | 1 | 0.0028 |
| Pseudoflavonifractor PAC002119_s group | Bacteria;Firmicutes;Clostridia;Clostridiales;Ruminococcaceae;Pseudoflavonifractor;PAC002119_s group | 1 | 0.0028 |
| Bacteroides PAC002300_s | Bacteria;Bacteroidetes;Bacteroidia;Bacteroidales;Bacteroidaceae;Bacteroides;PAC002300_s | 1 | 0.0028 |
| PAC001144_g PAC002359_s | Bacteria;Firmicutes;Clostridia;Clostridiales;Ruminococcaceae;PAC001144_g;PAC002359_s | 1 | 0.0028 |
| Pseudoflavonifractor PAC002397_s | Bacteria;Firmicutes;Clostridia;Clostridiales;Ruminococcaceae;Pseudoflavonifractor;PAC002397_s | 1 | 0.0028 |
| PAC002454_g PAC002454_s | Bacteria;Firmicutes;Clostridia;Clostridiales;Lachnospiraceae;PAC002454_g;PAC002454_s | 1 | 0.0028 |
| Pseudoflavonifractor PAC002475_s | Bacteria;Firmicutes;Clostridia;Clostridiales;Ruminococcaceae;Pseudoflavonifractor;PAC002475_s | 1 | 0.0028 |
| Agathobacter_uc | Bacteria;Firmicutes;Clostridia;Clostridiales;Lachnospiraceae;Agathobacter;Agathobacter_uc | 1 | 0.0028 |
| Coprobacillus_uc | Bacteria;Firmicutes;Erysipelotrichi;Erysipelotrichales;Erysipelotrichaceae;Coprobacillus;Coprobacillus_uc | 1 | 0.0028 |
| Gemella_uc | Bacteria;Firmicutes;Bacilli;Bacillales;Gemella_f;Gemella;Gemella_uc | 1 | 0.0028 |
| Lactococcus_uc | Bacteria;Firmicutes;Bacilli;Lactobacillales;Streptococcaceae;Lactococcus;Lactococcus_uc | 1 | 0.0028 |
| KE159605_g_uc | Bacteria;Firmicutes;Clostridia;Clostridiales;Lachnospiraceae;KE159605_g;KE159605_g_uc | 1 | 0.0028 |
| PAC000692_g_uc | Bacteria;Firmicutes;Clostridia;Clostridiales;Lachnospiraceae;PAC000692_g;PAC000692_g_uc | 1 | 0.0028 |
| PAC001063_g_uc | Bacteria;Bacteroidetes;Bacteroidia;Bacteroidales;Muribaculaceae;PAC001063_g;PAC001063_g_uc | 1 | 0.0028 |
| PAC001165_g_uc | Bacteria;Firmicutes;Clostridia;Clostridiales;Lachnospiraceae;PAC001165_g;PAC001165_g_uc | 1 | 0.0028 |

**Set 2_0.05%_S: Day 10_heme-SCP 0.05%_Species**

| Taxon name | Taxonomy | Count | Proportion(%) |
|---|---|---|---|
| Bacteroides vulgatus | Bacteria;Bacteroidetes;Bacteroidia;Bacteroidales;Bacteroidaceae;Bacteroides;Bacteroides vulgatus | 5909 | 13.2676 |
| Lactococcus lactis group | Bacteria;Firmicutes;Bacilli;Lactobacillales;Streptococcaceae;Lactococcus;Lactococcus lactis group | 3403 | 7.6408 |
| PAC001516_g PAC001516_s | Bacteria;Firmicutes;Clostridia;Clostridiales;Lachnospiraceae;PAC001516_g;PAC001516_s | 2544 | 5.7121 |
| KE159600_g KE159600_s | Bacteria;Firmicutes;Clostridia;Clostridiales;Lachnospiraceae;KE159600_g;KE159600_s | 2515 | 5.647 |
| Flintibacter butyricus group | Bacteria;Firmicutes;Clostridia;Clostridiales;Ruminococcaceae;Pseudoflavonifractor;Flintibacter butyricus group | 1877 | 4.2145 |
| Oscillibacter PAC001186_s group | Bacteria;Firmicutes;Clostridia;Clostridiales;Ruminococcaceae;Oscillibacter;PAC001186_s group | 1524 | 3.4219 |
| PAC002482_g EF603735_s | Bacteria;Bacteroidetes;Bacteroidia;Bacteroidales;AC160630_f;PAC002482_g;EF603735_s | 1307 | 2.9346 |
| Bacteroides HM124113_s | Bacteria;Bacteroidetes;Bacteroidia;Bacteroidales;Bacteroidaceae;Bacteroides;HM124113_s | 1139 | 2.5574 |
| Oscillibacter PAC001501_s | Bacteria;Firmicutes;Clostridia;Clostridiales;Ruminococcaceae;Oscillibacter;PAC001501_s | 1062 | 2.3845 |
| Bilophila wadsworthia | Bacteria;Proteobacteria;Deltaproteobacteria;Desulfovibrionales;Desulfovibrionaceae;Bilophila;Bilophila wadsworthia | 1014 | 2.2768 |
| Pseudoflavonifractor PAC001574_s group | Bacteria;Firmicutes;Clostridia;Clostridiales;Ruminococcaceae;Pseudoflavonifractor;PAC001574_s group | 895 | 2.0096 |
| PAC001516_g PAC002384_s | Bacteria;Firmicutes;Clostridia;Clostridiales;Lachnospiraceae;PAC001516_g;PAC002384_s | 829 | 1.8614 |
| Anaerotignum PAC001740_s | Bacteria;Firmicutes;Clostridia;Clostridiales;Lachnospiraceae;Anaerotignum;PAC001740_s | 827 | 1.8569 |
| Acetatifactor PAC001463_s | Bacteria;Firmicutes;Clostridia;Clostridiales;Lachnospiraceae;Acetatifactor;PAC001463_s | 801 | 1.7985 |
| Pseudoflavonifractor PAC001379_s | Bacteria;Firmicutes;Clostridia;Clostridiales;Ruminococcaceae;Pseudoflavonifractor;PAC001379_s | 763 | 1.7132 |
| KE159571_g PAC002009_s group | Bacteria;Firmicutes;Clostridia;Clostridiales;Lachnospiraceae;KE159571_g;PAC002009_s group | 717 | 1.6099 |
| Acetatifactor PAC002332_s | Bacteria;Firmicutes;Clostridia;Clostridiales;Lachnospiraceae;Acetatifactor;PAC002332_s | 695 | 1.5605 |
| PAC000664_g PAC001104_s | Bacteria;Firmicutes;Clostridia;Clostridiales;Lachnospiraceae;PAC000664_g;PAC001104_s | 635 | 1.4258 |
| PAC001402_g PAC001764_s | Bacteria;Firmicutes;Clostridia;Clostridiales;Ruminococcaceae;PAC001402_g;PAC001764_s | 608 | 1.3652 |
| Clostridium_g21 PAC001294_s | Bacteria;Firmicutes;Clostridia;Clostridiales;Lachnospiraceae;Clostridium_g21;PAC001294_s | 551 | 1.2372 |
| Oscillibacter PAC002372_s | Bacteria;Firmicutes;Clostridia;Clostridiales;Ruminococcaceae;Oscillibacter;PAC002372_s | 467 | 1.0486 |
| Pseudoflavonifractor_uc | Bacteria;Firmicutes;Clostridia;Clostridiales;Ruminococcaceae;Pseudoflavonifractor;Pseudoflavonifractor_uc | 457 | 1.0261 |
| PAC001112_g PAC001267_s | Bacteria;Bacteroidetes;Bacteroidia;Bacteroidales;Muribaculaceae;PAC001112_g;PAC001267_s | 453 | 1.0171 |
| PAC002375_g PAC002375_s | Bacteria;Firmicutes;Clostridia;Clostridiales;Lachnospiraceae;PAC002375_g;PAC002375_s | 404 | 0.9071 |
| PAC001063_g PAC001063_s | Bacteria;Bacteroidetes;Bacteroidia;Bacteroidales;Muribaculaceae;PAC001063_g;PAC001063_s | 401 | 0.9004 |
| Pseudoflavonifractor PAC001543_s | Bacteria;Firmicutes;Clostridia;Clostridiales;Ruminococcaceae;Pseudoflavonifractor;PAC001543_s | 399 | 0.8959 |
| Bacteroides_uc | Bacteria;Bacteroidetes;Bacteroidia;Bacteroidales;Bacteroidaceae;Bacteroides;Bacteroides_uc | 398 | 0.8936 |
| Bacteroides acidifaciens | Bacteria;Bacteroidetes;Bacteroidia;Bacteroidales;Bacteroidaceae;Bacteroides;Bacteroides acidifaciens | 386 | 0.8667 |
| PAC001400_g PAC001400_s | Bacteria;Firmicutes;Clostridia;Clostridiales;Peptococcaceae;PAC001400_g;PAC001400_s | 386 | 0.8667 |
| Alistipes PAC001061_s | Bacteria;Bacteroidetes;Bacteroidia;Bacteroidales;Rikenellaceae;Alistipes;PAC001061_s | 378 | 0.8487 |
| Eubacterium_g23 PAC001121_s group | Bacteria;Firmicutes;Clostridia;Clostridiales;Ruminococcaceae;Eubacterium_g23;PAC001121_s group | 368 | 0.8263 |
| Pseudoflavonifractor PAC001550_s | Bacteria;Firmicutes;Clostridia;Clostridiales;Ruminococcaceae;Pseudoflavonifractor;PAC001550_s | 324 | 0.7275 |
| PAC000198_g PAC001075_s | Bacteria;Bacteroidetes;Bacteroidia;Bacteroidales;Muribaculaceae;PAC000198_g;PAC001075_s | 316 | 0.7095 |
| KE159605_g KE159605_s | Bacteria;Firmicutes;Clostridia;Clostridiales;Lachnospiraceae;KE159605_g;KE159605_s | 307 | 0.6893 |
| Acetatifactor PAC001801_s | Bacteria;Firmicutes;Clostridia;Clostridiales;Lachnospiraceae;Acetatifactor;PAC001801_s | 285 | 0.6399 |
| Corynebacterium glutamicum | Bacteria;Actinobacteria;Actinobacteria_c;Corynebacteriales;Corynebacteriaceae;Corynebacterium;Corynebacterium glutamicum | 274 | 0.6152 |
| Acetatifactor PAC001773_s | Bacteria;Firmicutes;Clostridia;Clostridiales;Lachnospiraceae;Acetatifactor;PAC001773_s | 266 | 0.5973 |
| PAC001092_g PAC001668_s | Bacteria;Firmicutes;Clostridia;Clostridiales;Lachnospiraceae;PAC001092_g;PAC001668_s | 246 | 0.5523 |
| PAC001778_g PAC001778_s | Bacteria;Firmicutes;Clostridia;Clostridiales;Ruminococcaceae;PAC001778_g;PAC001778_s | 239 | 0.5366 |
| Clostridium_g21 EU506235_s | Bacteria;Firmicutes;Clostridia;Clostridiales;Lachnospiraceae;Clostridium_g21;EU506235_s | 230 | 0.5164 |
| Oscillibacter PAC001584_s group | Bacteria;Firmicutes;Clostridia;Clostridiales;Ruminococcaceae;Oscillibacter;PAC001584_s group | 228 | 0.5119 |
| PAC001385_g PAC001727_s group | Bacteria;Firmicutes;Clostridia;Clostridiales;Lachnospiraceae;PAC001385_g;PAC001727_s group | 227 | 0.5097 |
| Oscillibacter PAC001188_s | Bacteria;Firmicutes;Clostridia;Clostridiales;Ruminococcaceae;Oscillibacter;PAC001188_s | 219 | 0.4917 |
| PAC001228_g PAC002027_s | Bacteria;Firmicutes;Clostridia;Clostridiales;Lachnospiraceae;PAC001228_g;PAC002027_s | 215 | 0.4827 |
| Pseudoflavonifractor PAC001549_s | Bacteria;Firmicutes;Clostridia;Clostridiales;Ruminococcaceae;Pseudoflavonifractor;PAC001549_s | 203 | 0.4558 |
| Lactobacillus murinus group | Bacteria;Firmicutes;Bacilli;Lactobacillales;Lactobacillaceae;Lactobacillus;Lactobacillus murinus group | 186 | 0.4176 |
| Pseudoflavonifractor PAC001548_s | Bacteria;Firmicutes;Clostridia;Clostridiales;Ruminococcaceae;Pseudoflavonifractor;PAC001548_s | 179 | 0.4019 |
| PAC001221_g PAC001221_s | Bacteria;Firmicutes;Clostridia;Clostridiales;Dehalobacterium_f;PAC001221_g;PAC001221_s | 152 | 0.3413 |
| Clostridium_g21_uc | Bacteria;Firmicutes;Clostridia;Clostridiales;Lachnospiraceae;Clostridium_g21;Clostridium_g21_uc | 142 | 0.3188 |
| Caproiciproducens EU622658_s | Bacteria;Firmicutes;Clostridia;Clostridiales;Ruminococcaceae;Caproiciproducens;EU622658_s | 141 | 0.3166 |
| KE159571_g PAC001776_s group | Bacteria;Firmicutes;Clostridia;Clostridiales;Lachnospiraceae;KE159571_g;PAC001776_s group | 141 | 0.3166 |
| PAC001308_g DQ815557_s | Bacteria;Firmicutes;Clostridia;Clostridiales;Lachnospiraceae;PAC001308_g;DQ815557_s | 132 | 0.2964 |
| PAC001165_g PAC001384_s | Bacteria;Firmicutes;Clostridia;Clostridiales;Lachnospiraceae;PAC001165_g;PAC001384_s | 128 | 0.2874 |
| Frisingicoccus PAC001744_s group | Bacteria;Firmicutes;Clostridia;Clostridiales;Lachnospiraceae;Frisingicoccus;PAC001744_s group | 126 | 0.2829 |
| PAC001544_g PAC001544_s | Bacteria;Firmicutes;Clostridia;Clostridiales;Lachnospiraceae;PAC001544_g;PAC001544_s | 124 | 0.2784 |
| PAC000664_g PAC001769_s | Bacteria;Firmicutes;Clostridia;Clostridiales;Lachnospiraceae;PAC000664_g;PAC001769_s | 122 | 0.2739 |
| Anaerotruncus KE159677_s | Bacteria;Firmicutes;Clostridia;Clostridiales;Ruminococcaceae;Anaerotruncus;KE159677_s | 115 | 0.2582 |
| Pseudoflavonifractor PAC001087_s | Bacteria;Firmicutes;Clostridia;Clostridiales;Ruminococcaceae;Pseudoflavonifractor;PAC001087_s | 113 | 0.2537 |
| Oscillibacter PAC001369_s | Bacteria;Firmicutes;Clostridia;Clostridiales;Ruminococcaceae;Oscillibacter;PAC001369_s | 107 | 0.2402 |
| PAC000692_g PAC001387_s | Bacteria;Firmicutes;Clostridia;Clostridiales;Lachnospiraceae;PAC000692_g;PAC001387_s | 106 | 0.238 |
| PAC001103_g_uc | Bacteria;Firmicutes;Clostridia;Clostridiales;Lachnospiraceae;PAC001103_g;PAC001103_g_uc | 102 | 0.229 |
| PAC000664_g AB702805_s | Bacteria;Firmicutes;Clostridia;Clostridiales;Lachnospiraceae;PAC000664_g;AB702805_s | 96 | 0.2156 |
| Oscillibacter PAC001522_s | Bacteria;Firmicutes;Clostridia;Clostridiales;Ruminococcaceae;Oscillibacter;PAC001522_s | 95 | 0.2133 |
| Eubacterium_g6 PAC001586_s | Bacteria;Firmicutes;Clostridia;Clostridiales;Lachnospiraceae;Eubacterium_g6;PAC001586_s | 93 | 0.2088 |
| PAC001068_g PAC001068_s | Bacteria;Bacteroidetes;Bacteroidia;Bacteroidales;Muribaculaceae;PAC001068_g;PAC001068_s | 92 | 0.2066 |
| Clostridium_g21 KE159781_s | Bacteria;Firmicutes;Clostridia;Clostridiales;Lachnospiraceae;Clostridium_g21;KE159781_s | 91 | 0.2043 |
| PAC001066_g PAC001066_s | Bacteria;Bacteroidetes;Bacteroidia;Bacteroidales;Muribaculaceae;PAC001066_g;PAC001066_s | 90 | 0.2021 |
| Mucispirillum schaedleri | Bacteria;Deferribacteres;Deferribacteres_c;Deferribacterales;Deferribacteraceae;Mucispirillum;Mucispirillum schaedleri | 89 | 0.1998 |
| PAC001201_g PAC001201_s group | Bacteria;Firmicutes;Clostridia;Clostridiales;Lachnospiraceae;PAC001201_g;PAC001201_s group | 87 | 0.1953 |
| Oscillibacter PAC002110_s | Bacteria;Firmicutes;Clostridia;Clostridiales;Ruminococcaceae;Oscillibacter;PAC002110_s | 87 | 0.1953 |
| Bacteroides stercorirosoris | Bacteria;Bacteroidetes;Bacteroidia;Bacteroidales;Bacteroidaceae;Bacteroides;Bacteroides stercorirosoris | 86 | 0.1931 |
| PAC001457_g PAC001495_s group | Bacteria;Firmicutes;Clostridia;Clostridiales;Lachnospiraceae;PAC001457_g;PAC001495_s group | 85 | 0.1909 |
| PAC001043_g PAC002511_s group | Bacteria;Firmicutes;Clostridia;Clostridiales;Lachnospiraceae;PAC001043_g;PAC002511_s group | 85 | 0.1909 |
| PAC001092_g PAC001774_s | Bacteria;Firmicutes;Clostridia;Clostridiales;Lachnospiraceae;PAC001092_g;PAC001774_s | 84 | 0.1886 |
| PAC001149_g PAC001511_s | Bacteria;Firmicutes;Clostridia;Clostridiales;Lachnospiraceae;PAC001149_g;PAC001511_s | 82 | 0.1841 |
| Oscillibacter PAC001156_s | Bacteria;Firmicutes;Clostridia;Clostridiales;Ruminococcaceae;Oscillibacter;PAC001156_s | 74 | 0.1662 |
| Acetatifactor_uc | Bacteria;Firmicutes;Clostridia;Clostridiales;Lachnospiraceae;Acetatifactor;Acetatifactor_uc | 74 | 0.1662 |
| PAC001103_g PAC001361_s | Bacteria;Firmicutes;Clostridia;Clostridiales;Lachnospiraceae;PAC001103_g;PAC001361_s | 70 | 0.1572 |
| PAC001074_g PAC001074_s | Bacteria;Bacteroidetes;Bacteroidia;Bacteroidales;Muribaculaceae;PAC001074_g;PAC001074_s | 69 | 0.1549 |
| PAC001402_g PAC001748_s | Bacteria;Firmicutes;Clostridia;Clostridiales;Ruminococcaceae;PAC001402_g;PAC001748_s | 68 | 0.1527 |
| Desulfovibrio PAC001079_s | Bacteria;Proteobacteria;Deltaproteobacteria;Desulfovibrionales;Desulfovibrionaceae;Desulfovibrio;PAC001079_s | 67 | 0.1504 |
| PAC001112_g PAC002441_s | Bacteria;Bacteroidetes;Bacteroidia;Bacteroidales;Muribaculaceae;PAC001112_g;PAC002441_s | 67 | 0.1504 |
| Acetatifactor muris | Bacteria;Firmicutes;Clostridia;Clostridiales;Lachnospiraceae;Acetatifactor;Acetatifactor muris | 66 | 0.1482 |
| PAC000664_g PAC001083_s | Bacteria;Firmicutes;Clostridia;Clostridiales;Lachnospiraceae;PAC000664_g;PAC001083_s | 60 | 0.1347 |
| KE159600_g EU511112_s | Bacteria;Firmicutes;Clostridia;Clostridiales;Lachnospiraceae;KE159600_g;EU511112_s | 58 | 0.1302 |
| Pseudoflavonifractor KI535319_s | Bacteria;Firmicutes;Clostridia;Clostridiales;Ruminococcaceae;Pseudoflavonifractor;KI535319_s | 57 | 0.128 |
| PAC001524_g PAC001524_s | Bacteria;Firmicutes;Clostridia;Clostridiales;Lachnospiraceae;PAC001524_g;PAC001524_s | 54 | 0.1212 |
| KE159571_g PAC002442_s | Bacteria;Firmicutes;Clostridia;Clostridiales;Lachnospiraceae;KE159571_g;PAC002442_s | 53 | 0.119 |
| PAC001092_g PAC001742_s | Bacteria;Firmicutes;Clostridia;Clostridiales;Lachnospiraceae;PAC001092_g;PAC001742_s | 51 | 0.1145 |
| Clostridium_g21 PAC001757_s group | Bacteria;Firmicutes;Clostridia;Clostridiales;Lachnospiraceae;Clostridium_g21;PAC001757_s group | 51 | 0.1145 |
| Eubacterium_g23 PAC001490_s | Bacteria;Firmicutes;Clostridia;Clostridiales;Ruminococcaceae;Eubacterium_g23;PAC001490_s | 50 | 0.1123 |
| PAC001138_g PAC001518_s | Bacteria;Firmicutes;Clostridia;Clostridiales;Lachnospiraceae;PAC001138_g;PAC001518_s | 49 | 0.11 |
| KE159571_g KE159571_s | Bacteria;Firmicutes;Clostridia;Clostridiales;Lachnospiraceae;KE159571_g;KE159571_s | 47 | 0.1055 |
| Caproiciproducens PAC001505_s | Bacteria;Firmicutes;Clostridia;Clostridiales;Ruminococcaceae;Caproiciproducens;PAC001505_s | 47 | 0.1055 |
| PAC000692_g PAC001745_s | Bacteria;Firmicutes;Clostridia;Clostridiales;Lachnospiraceae;PAC000692_g;PAC001745_s | 47 | 0.1055 |
| Pseudoflavonifractor PAC001542_s | Bacteria;Firmicutes;Clostridia;Clostridiales;Ruminococcaceae;Pseudoflavonifractor;PAC001542_s | 46 | 0.1033 |
| Acutalibacter PAC002354_s | Bacteria;Firmicutes;Clostridia;Clostridiales;Ruminococcaceae;Acutalibacter;PAC002354_s | 46 | 0.1033 |
| Oscillibacter KE159714_s group | Bacteria;Firmicutes;Clostridia;Clostridiales;Ruminococcaceae;Oscillibacter;KE159714_s group | 45 | 0.101 |
| PAC001270_g PAC002420_s | Bacteria;Firmicutes;Clostridia;Clostridiales;Lachnospiraceae;PAC001270_g;PAC002420_s | 45 | 0.101 |
| PAC001612_g PAC001612_s | Bacteria;Firmicutes;Clostridia;Clostridiales;Mogibacterium_f;PAC001612_g;PAC001612_s | 43 | 0.0965 |
| Paludicola PAC001507_s | Bacteria;Firmicutes;Clostridia;Clostridiales;Ruminococcaceae;Paludicola;PAC001507_s | 42 | 0.0943 |
| PAC001092_g PAC001587_s | Bacteria;Firmicutes;Clostridia;Clostridiales;Lachnospiraceae;PAC001092_g;PAC001587_s | 42 | 0.0943 |
| Oscillibacter FJ881219_s | Bacteria;Firmicutes;Clostridia;Clostridiales;Ruminococcaceae;Oscillibacter;FJ881219_s | 38 | 0.0853 |
| PAC002482_g_uc | Bacteria;Bacteroidetes;Bacteroidia;Bacteroidales;AC160630_f;PAC002482_g;PAC002482_g_uc | 38 | 0.0853 |
| Massilioclostridium PAC001521_s | Bacteria;Firmicutes;Clostridia;Clostridiales;Ruminococcaceae;Massilioclostridium;PAC001521_s | 35 | 0.0786 |
| KE159571_g KE159641_s | Bacteria;Firmicutes;Clostridia;Clostridiales;Lachnospiraceae;KE159571_g;KE159641_s | 34 | 0.0763 |
| Clostridium_g21 PAC002512_s group | Bacteria;Firmicutes;Clostridia;Clostridiales;Lachnospiraceae;Clostridium_g21;PAC002512_s group | 34 | 0.0763 |
| Frisingicoccus EF409953_s | Bacteria;Firmicutes;Clostridia;Clostridiales;Lachnospiraceae;Frisingicoccus;EF409953_s | 33 | 0.0741 |
| Clostridium_g24 PAC001374_s | Bacteria;Firmicutes;Clostridia;Clostridiales;Lachnospiraceae;Clostridium_g24;PAC001374_s | 33 | 0.0741 |
| Bacteroides caecimuris | Bacteria;Bacteroidetes;Bacteroidia;Bacteroidales;Bacteroidaceae;Bacteroides;Bacteroides caecimuris | 32 | 0.0719 |
| Lachnospiraceae_uc | Bacteria;Firmicutes;Clostridia;Clostridiales;Lachnospiraceae;Lachnospiraceae_uc | 31 | 0.0696 |
| PAC001588_g PAC001588_s | Bacteria;Firmicutes;Clostridia;Clostridiales;Lachnospiraceae;PAC001588_g;PAC001588_s | 31 | 0.0696 |
| KE159797_g PAC001222_s | Bacteria;Firmicutes;Clostridia;Clostridiales;Lachnospiraceae;KE159797_g;PAC001222_s | 30 | 0.0674 |
| Frisingicoccus PAC001399_s | Bacteria;Firmicutes;Clostridia;Clostridiales;Lachnospiraceae;Frisingicoccus;PAC001399_s | 30 | 0.0674 |
| PAC001402_g PAC001561_s | Bacteria;Firmicutes;Clostridia;Clostridiales;Ruminococcaceae;PAC001402_g;PAC001561_s | 30 | 0.0674 |
| Anaerotruncus PAC001590_s | Bacteria;Firmicutes;Clostridia;Clostridiales;Ruminococcaceae;Anaerotruncus;PAC001590_s | 29 | 0.0651 |
| PAC001092_g_uc | Bacteria;Firmicutes;Clostridia;Clostridiales;Lachnospiraceae;PAC001092_g;PAC001092_g_uc | 29 | 0.0651 |
| Pseudoflavonifractor EU454100_s | Bacteria;Firmicutes;Clostridia;Clostridiales;Ruminococcaceae;Pseudoflavonifractor;EU454100_s | 28 | 0.0629 |
| Pseudoflavonifractor PAC001667_s | Bacteria;Firmicutes;Clostridia;Clostridiales;Ruminococcaceae;Pseudoflavonifractor;PAC001667_s | 28 | 0.0629 |
| Eubacterium_g17 PAC001119_s | Bacteria;Firmicutes;Clostridia;Clostridiales;Lachnospiraceae;Eubacterium_g17;PAC001119_s | 27 | 0.0606 |
| PAC000671_g PAC001473_s | Bacteria;Firmicutes;Clostridia;Clostridiales;Lachnospiraceae;PAC000671_g;PAC001473_s | 27 | 0.0606 |
| PAC001225_g PAC001225_s | Bacteria;Firmicutes;Clostridia;Clostridiales;Lachnospiraceae;PAC001225_g;PAC001225_s | 26 | 0.0584 |
| PAC001313_g PAC001313_s | Bacteria;Firmicutes;Clostridia;Clostridiales;Ruminococcaceae;PAC001313_g;PAC001313_s | 26 | 0.0584 |
| PAC001043_g PAC001752_s | Bacteria;Firmicutes;Clostridia;Clostridiales;Lachnospiraceae;PAC001043_g;PAC001752_s | 26 | 0.0584 |
| PAC002460_g PAC002460_s | Bacteria;Firmicutes;Clostridia;Clostridiales;Lachnospiraceae;PAC002460_g;PAC002460_s | 25 | 0.0561 |
| Enterorhabdus caecimuris | Bacteria;Actinobacteria;Coriobacteriia;Coriobacteriales;Coriobacteriaceae;Enterorhabdus;Enterorhabdus caecimuris | 24 | 0.0539 |
| Oscillibacter PAC001502_s | Bacteria;Firmicutes;Clostridia;Clostridiales;Ruminococcaceae;Oscillibacter;PAC001502_s | 24 | 0.0539 |
| Oscillibacter EU454374_s | Bacteria;Firmicutes;Clostridia;Clostridiales;Ruminococcaceae;Oscillibacter;EU454374_s | 23 | 0.0516 |
| PAC000664_g_uc | Bacteria;Firmicutes;Clostridia;Clostridiales;Lachnospiraceae;PAC000664_g;PAC000664_g_uc | 23 | 0.0516 |
| Bacillus cereus group | Bacteria;Firmicutes;Bacilli;Bacillales;Bacillaceae;Bacillus;Bacillus cereus group | 22 | 0.0494 |
| PAC001372_g PAC001372_s | Bacteria;Firmicutes;Clostridia;Clostridiales;Lachnospiraceae;PAC001372_g;PAC001372_s | 22 | 0.0494 |
| Pseudoflavonifractor FJ879979_s | Bacteria;Firmicutes;Clostridia;Clostridiales;Ruminococcaceae;Pseudoflavonifractor;FJ879979_s | 21 | 0.0472 |
| PAC000661_g PAC001154_s | Bacteria;Firmicutes;Clostridia;Clostridiales;Ruminococcaceae;PAC000661_g;PAC001154_s | 21 | 0.0472 |
| PAC002390_g PAC002390_s | Bacteria;Firmicutes;Clostridia;Clostridiales;Dehalobacterium_f;PAC002390_g;PAC002390_s | 21 | 0.0472 |
| Oscillibacter PAC001638_s | Bacteria;Firmicutes;Clostridia;Clostridiales;Ruminococcaceae;Oscillibacter;PAC001638_s | 20 | 0.0449 |
| Acetatifactor FJ879577_s | Bacteria;Firmicutes;Clostridia;Clostridiales;Lachnospiraceae;Acetatifactor;FJ879577_s | 19 | 0.0427 |
| Harryflintia PAC002149_s | Bacteria;Firmicutes;Clostridia;Clostridiales;Ruminococcaceae;Harryflintia;PAC002149_s | 19 | 0.0427 |
| Clostridium_g21 PAC001085_s | Bacteria;Firmicutes;Clostridia;Clostridiales;Lachnospiraceae;Clostridium_g21;PAC001085_s | 18 | 0.0404 |
| PAC001092_g PAC001499_s | Bacteria;Firmicutes;Clostridia;Clostridiales;Lachnospiraceae;PAC001092_g;PAC001499_s | 18 | 0.0404 |
| Parabacteroides goldsteinii | Bacteria;Bacteroidetes;Bacteroidia;Bacteroidales;Porphyromonadaceae;Parabacteroides;Parabacteroides goldsteinii | 17 | 0.0382 |
| Arthromitus AP012202_s group | Bacteria;Firmicutes;Clostridia;Clostridiales;Clostridiaceae;Arthromitus;AP012202_s group | 17 | 0.0382 |
| Pseudoflavonifractor LN866274_s | Bacteria;Firmicutes;Clostridia;Clostridiales;Ruminococcaceae;Pseudoflavonifractor;LN866274_s | 17 | 0.0382 |
| Pseudoflavonifractor PAC001088_s | Bacteria;Firmicutes;Clostridia;Clostridiales;Ruminococcaceae;Pseudoflavonifractor;PAC001088_s | 17 | 0.0382 |
| Pseudoflavonifractor PAC001786_s | Bacteria;Firmicutes;Clostridia;Clostridiales;Ruminococcaceae;Pseudoflavonifractor;PAC001786_s | 17 | 0.0382 |
| Ruminococcaceae_uc | Bacteria;Firmicutes;Clostridia;Clostridiales;Ruminococcaceae;Ruminococcaceae_uc | 16 | 0.0359 |
| Gemella palaticanis | Bacteria;Firmicutes;Bacilli;Bacillales;Gemella_f;Gemella;Gemella palaticanis | 16 | 0.0359 |
| Clostridium_g24 EU504811_s | Bacteria;Firmicutes;Clostridia;Clostridiales;Lachnospiraceae;Clostridium_g24;EU504811_s | 16 | 0.0359 |
| Frisingicoccus PAC000700_s | Bacteria;Firmicutes;Clostridia;Clostridiales;Lachnospiraceae;Frisingicoccus;PAC000700_s | 16 | 0.0359 |
| Bacteroides uniformis | Bacteria;Bacteroidetes;Bacteroidia;Bacteroidales;Bacteroidaceae;Bacteroides;Bacteroides uniformis | 15 | 0.0337 |
| PAC001402_g PAC001546_s | Bacteria;Firmicutes;Clostridia;Clostridiales;Ruminococcaceae;PAC001402_g;PAC001546_s | 15 | 0.0337 |
| Streptococcus acidominimus group | Bacteria;Firmicutes;Bacilli;Lactobacillales;Streptococcaceae;Streptococcus;Streptococcus acidominimus group | 14 | 0.0314 |
| PAC000661_g EU842956_s | Bacteria;Firmicutes;Clostridia;Clostridiales;Ruminococcaceae;PAC000661_g;EU842956_s | 14 | 0.0314 |
| PAC001062_g PAC001062_s | Bacteria;Bacteroidetes;Bacteroidia;Bacteroidales;Muribaculaceae;PAC001062_g;PAC001062_s | 14 | 0.0314 |
| Frisingicoccus PAC001728_s group | Bacteria;Firmicutes;Clostridia;Clostridiales;Lachnospiraceae;Frisingicoccus;PAC001728_s group | 14 | 0.0314 |
| KE159571_g PAC002035_s | Bacteria;Firmicutes;Clostridia;Clostridiales;Lachnospiraceae;KE159571_g;PAC002035_s | 14 | 0.0314 |
| Eubacterium_g6 PAC002402_s | Bacteria;Firmicutes;Clostridia;Clostridiales;Lachnospiraceae;Eubacterium_g6;PAC002402_s | 14 | 0.0314 |
| Bilophila_uc | Bacteria;Proteobacteria;Deltaproteobacteria;Desulfovibrionales;Desulfovibrionaceae;Bilophila;Bilophila_uc | 14 | 0.0314 |
| Bacteroides DQ805799_s | Bacteria;Bacteroidetes;Bacteroidia;Bacteroidales;Bacteroidaceae;Bacteroides;DQ805799_s | 13 | 0.0292 |
| Pseudoflavonifractor EU460161_s | Bacteria;Firmicutes;Clostridia;Clostridiales;Ruminococcaceae;Pseudoflavonifractor;EU460161_s | 13 | 0.0292 |
| Sporobacter PAC001096_s | Bacteria;Firmicutes;Clostridia;Clostridiales;Ruminococcaceae;Sporobacter;PAC001096_s | 13 | 0.0292 |
| Anaerotruncus colihominis | Bacteria;Firmicutes;Clostridia;Clostridiales;Ruminococcaceae;Anaerotruncus;Anaerotruncus colihominis | 12 | 0.0269 |
| Bacteroides DQ799557_s | Bacteria;Bacteroidetes;Bacteroidia;Bacteroidales;Bacteroidaceae;Bacteroides;DQ799557_s | 12 | 0.0269 |
| PAC000748_g PAC001099_s | Bacteria;Firmicutes;Clostridia;Clostridiales;Ruminococcaceae;PAC000748_g;PAC001099_s | 12 | 0.0269 |
| Sporobacter PAC001659_s | Bacteria;Firmicutes;Clostridia;Clostridiales;Ruminococcaceae;Sporobacter;PAC001659_s | 12 | 0.0269 |
| Pseudoflavonifractor PAC002490_s | Bacteria;Firmicutes;Clostridia;Clostridiales;Ruminococcaceae;Pseudoflavonifractor;PAC002490_s | 12 | 0.0269 |
| Oscillibacter_uc | Bacteria;Firmicutes;Clostridia;Clostridiales;Ruminococcaceae;Oscillibacter;Oscillibacter_uc | 12 | 0.0269 |
| Escherichia coli group | Bacteria;Proteobacteria;Gammaproteobacteria;Enterobacterales;Enterobacteriaceae;Escherichia;Escherichia coli group | 11 | 0.0247 |
| Christensenella EU508399_s | Bacteria;Firmicutes;Clostridia;Clostridiales;Christensenellaceae;Christensenella;EU508399_s | 11 | 0.0247 |
| Agathobaculum PAC001476_s | Bacteria;Firmicutes;Clostridia;Clostridiales;Ruminococcaceae;Agathobaculum;PAC001476_s | 11 | 0.0247 |
| Pseudoflavonifractor PAC002119_s group | Bacteria;Firmicutes;Clostridia;Clostridiales;Ruminococcaceae;Pseudoflavonifractor;PAC002119_s group | 11 | 0.0247 |
| KE159600_g EF098562_s | Bacteria;Firmicutes;Clostridia;Clostridiales;Lachnospiraceae;KE159600_g;EF098562_s | 10 | 0.0225 |
| PAC001092_g PAC001388_s | Bacteria;Firmicutes;Clostridia;Clostridiales;Lachnospiraceae;PAC001092_g;PAC001388_s | 10 | 0.0225 |
| PAC001200_g PAC001554_s | Bacteria;Firmicutes;Clostridia;Clostridiales;Lachnospiraceae;PAC001200_g;PAC001554_s | 10 | 0.0225 |
| Pseudoflavonifractor PAC001608_s | Bacteria;Firmicutes;Clostridia;Clostridiales;Ruminococcaceae;Pseudoflavonifractor;PAC001608_s | 10 | 0.0225 |
| PAC001092_g PAC001710_s | Bacteria;Firmicutes;Clostridia;Clostridiales;Lachnospiraceae;PAC001092_g;PAC001710_s | 10 | 0.0225 |
| Pseudoflavonifractor FJ879507_s | Bacteria;Firmicutes;Clostridia;Clostridiales;Ruminococcaceae;Pseudoflavonifractor;FJ879507_s | 9 | 0.0202 |
| KE159810_g KE159810_s | Bacteria;Firmicutes;Clostridia;Clostridiales;Lachnospiraceae;KE159810_g;KE159810_s | 9 | 0.0202 |
| PAC001266_g PAC001266_s | Bacteria;Actinobacteria;Coriobacteriia;Coriobacteriales;Coriobacteriaceae;PAC001266_g;PAC001266_s | 9 | 0.0202 |
| PAC000748_g PAC001489_s | Bacteria;Firmicutes;Clostridia;Clostridiales;Ruminococcaceae;PAC000748_g;PAC001489_s | 9 | 0.0202 |
| KE159571_g PAC001705_s | Bacteria;Firmicutes;Clostridia;Clostridiales;Lachnospiraceae;KE159571_g;PAC001705_s | 9 | 0.0202 |
| Oscillibacter PAC001706_s | Bacteria;Firmicutes;Clostridia;Clostridiales;Ruminococcaceae;Oscillibacter;PAC001706_s | 9 | 0.0202 |
| Christensenella PAC002165_s | Bacteria;Firmicutes;Clostridia;Clostridiales;Christensenellaceae;Christensenella;PAC002165_s | 9 | 0.0202 |
| PAC001066_g_uc | Bacteria;Bacteroidetes;Bacteroidia;Bacteroidales;Muribaculaceae;PAC001066_g;PAC001066_g_uc | 9 | 0.0202 |
| PAC001323_g PAC001323_s | Bacteria;Tenericutes;Mollicutes;PAC001057_o;PAC000197_f;PAC001323_g;PAC001323_s | 8 | 0.018 |
| KE159571_g PAC001555_s | Bacteria;Firmicutes;Clostridia;Clostridiales;Lachnospiraceae;KE159571_g;PAC001555_s | 8 | 0.018 |
| PAC001144_g PAC001593_s | Bacteria;Firmicutes;Clostridia;Clostridiales;Ruminococcaceae;PAC001144_g;PAC001593_s | 8 | 0.018 |
| Pseudoflavonifractor PAC002302_s | Bacteria;Firmicutes;Clostridia;Clostridiales;Ruminococcaceae;Pseudoflavonifractor;PAC002302_s | 8 | 0.018 |
| KE159605_g KB822463_s | Bacteria;Firmicutes;Clostridia;Clostridiales;Lachnospiraceae;KE159605_g;KB822463_s | 7 | 0.0157 |
| KE159600_g PAC001232_s | Bacteria;Firmicutes;Clostridia;Clostridiales;Lachnospiraceae;KE159600_g;PAC001232_s | 7 | 0.0157 |
| Acetatifactor PAC001373_s | Bacteria;Firmicutes;Clostridia;Clostridiales;Lachnospiraceae;Acetatifactor;PAC001373_s | 7 | 0.0157 |
| PAC001270_g PAC001899_s group | Bacteria;Firmicutes;Clostridia;Clostridiales;Lachnospiraceae;PAC001270_g;PAC001899_s group | 7 | 0.0157 |
| PAC002039_g PAC002039_s | Bacteria;Firmicutes;Clostridia;Clostridiales;Lachnospiraceae;PAC002039_g;PAC002039_s | 7 | 0.0157 |
| PAC000661_g_uc | Bacteria;Firmicutes;Clostridia;Clostridiales;Ruminococcaceae;PAC000661_g;PAC000661_g_uc | 7 | 0.0157 |
| Acutalibacter muris | Bacteria;Firmicutes;Clostridia;Clostridiales;Ruminococcaceae;Acutalibacter;Acutalibacter muris | 6 | 0.0135 |
| Akkermansia muciniphila | Bacteria;Verrucomicrobia;Verrucomicrobiae;Verrucomicrobiales;Akkermansiaceae;Akkermansia;Akkermansia muciniphila | 6 | 0.0135 |
| Oscillibacter EU771312_s | Bacteria;Firmicutes;Clostridia;Clostridiales;Ruminococcaceae;Oscillibacter;EU771312_s | 6 | 0.0135 |
| Anaerotignum PAC000685_s | Bacteria;Firmicutes;Clostridia;Clostridiales;Lachnospiraceae;Anaerotignum;PAC000685_s | 6 | 0.0135 |
| PAC000692_g PAC000692_s | Bacteria;Firmicutes;Clostridia;Clostridiales;Lachnospiraceae;PAC000692_g;PAC000692_s | 6 | 0.0135 |
| PAC001165_g PAC001366_s | Bacteria;Firmicutes;Clostridia;Clostridiales;Lachnospiraceae;PAC001165_g;PAC001366_s | 6 | 0.0135 |
| Oscillibacter PAC001551_s | Bacteria;Firmicutes;Clostridia;Clostridiales;Ruminococcaceae;Oscillibacter;PAC001551_s | 6 | 0.0135 |
| PAC000692_g PAC001666_s | Bacteria;Firmicutes;Clostridia;Clostridiales;Lachnospiraceae;PAC000692_g;PAC001666_s | 6 | 0.0135 |
| PAC001092_g PAC001726_s | Bacteria;Firmicutes;Clostridia;Clostridiales;Lachnospiraceae;PAC001092_g;PAC001726_s | 6 | 0.0135 |
| PAC000661_g PAC001768_s | Bacteria;Firmicutes;Clostridia;Clostridiales;Ruminococcaceae;PAC000661_g;PAC001768_s | 6 | 0.0135 |
| Lactococcus_uc | Bacteria;Firmicutes;Bacilli;Lactobacillales;Streptococcaceae;Lactococcus;Lactococcus_uc | 6 | 0.0135 |
| Clostridium methylpentosum | Bacteria;Firmicutes;Clostridia;Clostridiales;Ruminococcaceae;Massilioclostridium;Clostridium methylpentosum | 5 | 0.0112 |
| Frisingicoccus PAC000688_s | Bacteria;Firmicutes;Clostridia;Clostridiales;Lachnospiraceae;Frisingicoccus;PAC000688_s | 5 | 0.0112 |
| PAC001103_g PAC001103_s | Bacteria;Firmicutes;Clostridia;Clostridiales;Lachnospiraceae;PAC001103_g;PAC001103_s | 5 | 0.0112 |
| Christensenella PAC001140_s | Bacteria;Firmicutes;Clostridia;Clostridiales;Christensenellaceae;Christensenella;PAC001140_s | 5 | 0.0112 |
| Emergencia PAC001235_s | Bacteria;Firmicutes;Clostridia;Clostridiales;Mogibacterium_f;Emergencia;PAC001235_s | 5 | 0.0112 |
| PAC000748_g PAC001412_s | Bacteria;Firmicutes;Clostridia;Clostridiales;Ruminococcaceae;PAC000748_g;PAC001412_s | 5 | 0.0112 |
| Acutalibacter PAC001519_s | Bacteria;Firmicutes;Clostridia;Clostridiales;Ruminococcaceae;Acutalibacter;PAC001519_s | 5 | 0.0112 |
| PAC001528_g PAC001528_s | Bacteria;Firmicutes;Erysipelotrichi;Erysipelotrichales;Erysipelotrichaceae;PAC001528_g;PAC001528_s | 5 | 0.0112 |
| PAC000661_g PAC001716_s | Bacteria;Firmicutes;Clostridia;Clostridiales;Ruminococcaceae;PAC000661_g;PAC001716_s | 5 | 0.0112 |
| PAC000661_g PAC001777_s group | Bacteria;Firmicutes;Clostridia;Clostridiales;Ruminococcaceae;PAC000661_g;PAC001777_s group | 5 | 0.0112 |
| PAC001778_g PAC001909_s | Bacteria;Firmicutes;Clostridia;Clostridiales;Ruminococcaceae;PAC001778_g;PAC001909_s | 5 | 0.0112 |
| PAC002454_g PAC002454_s | Bacteria;Firmicutes;Clostridia;Clostridiales;Lachnospiraceae;PAC002454_g;PAC002454_s | 5 | 0.0112 |
| Pseudoflavonifractor PAC002475_s | Bacteria;Firmicutes;Clostridia;Clostridiales;Ruminococcaceae;Pseudoflavonifractor;PAC002475_s | 5 | 0.0112 |
| KE159571_g_uc | Bacteria;Firmicutes;Clostridia;Clostridiales;Lachnospiraceae;KE159571_g;KE159571_g_uc | 5 | 0.0112 |
| KE159600_g_uc | Bacteria;Firmicutes;Clostridia;Clostridiales;Lachnospiraceae;KE159600_g;KE159600_g_uc | 5 | 0.0112 |
| PAC001778_g_uc | Bacteria;Firmicutes;Clostridia;Clostridiales;Ruminococcaceae;PAC001778_g;PAC001778_g_uc | 5 | 0.0112 |
| Bacteroidaceae_uc | Bacteria;Bacteroidetes;Bacteroidia;Bacteroidales;Bacteroidaceae;Bacteroidaceae_uc | 4 | 0.009 |
| Bacteroides intestinalis | Bacteria;Bacteroidetes;Bacteroidia;Bacteroidales;Bacteroidaceae;Bacteroides;Bacteroides intestinalis | 4 | 0.009 |
| PAC000661_g AB185622_s | Bacteria;Firmicutes;Clostridia;Clostridiales;Ruminococcaceae;PAC000661_g;AB185622_s | 4 | 0.009 |
| Pseudoflavonifractor EF602939_s | Bacteria;Firmicutes;Clostridia;Clostridiales;Ruminococcaceae;Pseudoflavonifractor;EF602939_s | 4 | 0.009 |
| Pseudoflavonifractor FJ879818_s | Bacteria;Firmicutes;Clostridia;Clostridiales;Ruminococcaceae;Pseudoflavonifractor;FJ879818_s | 4 | 0.009 |
| Pseudoflavonifractor HQ716472_s | Bacteria;Firmicutes;Clostridia;Clostridiales;Ruminococcaceae;Pseudoflavonifractor;HQ716472_s | 4 | 0.009 |
| PAC000186_g PAC001065_s | Bacteria;Bacteroidetes;Bacteroidia;Bacteroidales;Muribaculaceae;PAC000186_g;PAC001065_s | 4 | 0.009 |
| Eubacterium_g4 PAC001174_s | Bacteria;Firmicutes;Clostridia;Clostridiales;Lachnospiraceae;Eubacterium_g4;PAC001174_s | 4 | 0.009 |
| PAC001103_g PAC001487_s | Bacteria;Firmicutes;Clostridia;Clostridiales;Lachnospiraceae;PAC001103_g;PAC001487_s | 4 | 0.009 |
| PAC001092_g PAC001517_s | Bacteria;Firmicutes;Clostridia;Clostridiales;Lachnospiraceae;PAC001092_g;PAC001517_s | 4 | 0.009 |
| Pseudoflavonifractor PAC001547_s | Bacteria;Firmicutes;Clostridia;Clostridiales;Ruminococcaceae;Pseudoflavonifractor;PAC001547_s | 4 | 0.009 |
| Harryflintia PAC001713_s | Bacteria;Firmicutes;Clostridia;Clostridiales;Ruminococcaceae;Harryflintia;PAC001713_s | 4 | 0.009 |
| Coprococcus_g2 PAC001722_s | Bacteria;Firmicutes;Clostridia;Clostridiales;Lachnospiraceae;Coprococcus_g2;PAC001722_s | 4 | 0.009 |
| Emergencia PAC001931_s | Bacteria;Firmicutes;Clostridia;Clostridiales;Mogibacterium_f;Emergencia;PAC001931_s | 4 | 0.009 |
| CCYH_g PAC002071_s group | Bacteria;Firmicutes;Clostridia;Clostridiales;Ruminococcaceae;CCYH_g;PAC002071_s group | 4 | 0.009 |
| Clostridium cocleatum | Bacteria;Firmicutes;Erysipelotrichi;Erysipelotrichales;Erysipelotrichaceae;Clostridium_g6;Clostridium cocleatum | 3 | 0.0067 |
| Clostridium tertium group | Bacteria;Firmicutes;Clostridia;Clostridiales;Clostridiaceae;Clostridium;Clostridium tertium group | 3 | 0.0067 |
| Parvibacter caecicola | Bacteria;Actinobacteria;Coriobacteriia;Coriobacteriales;Coriobacteriaceae;Parvibacter;Parvibacter caecicola | 3 | 0.0067 |
| Pseudoflavonifractor ADDX_s | Bacteria;Firmicutes;Clostridia;Clostridiales;Ruminococcaceae;Pseudoflavonifractor;ADDX_s | 3 | 0.0067 |
| PAC000661_g DQ456215_s | Bacteria;Firmicutes;Clostridia;Clostridiales;Ruminococcaceae;PAC000661_g;DQ456215_s | 3 | 0.0067 |
| Muribaculum DQ815542_s | Bacteria;Bacteroidetes;Bacteroidia;Bacteroidales;Muribaculaceae;Muribaculum;DQ815542_s | 3 | 0.0067 |
| GQ451199_g EU472543_s | Bacteria;Firmicutes;Clostridia;Clostridiales;Ruminococcaceae;GQ451199_g;EU472543_s | 3 | 0.0067 |
| PAC001225_g EU504346_s | Bacteria;Firmicutes;Clostridia;Clostridiales;Lachnospiraceae;PAC001225_g;EU504346_s | 3 | 0.0067 |
| Pseudoflavonifractor FJ879530_s | Bacteria;Firmicutes;Clostridia;Clostridiales;Ruminococcaceae;Pseudoflavonifractor;FJ879530_s | 3 | 0.0067 |
| Romboutsia FJTZ_s | Bacteria;Firmicutes;Clostridia;Clostridiales;Peptostreptococcaceae;Romboutsia;FJTZ_s | 3 | 0.0067 |
| Clostridium_g24 PAC000183_s | Bacteria;Firmicutes;Clostridia;Clostridiales;Lachnospiraceae;Clostridium_g24;PAC000183_s | 3 | 0.0067 |
| Pseudoflavonifractor PAC001131_s | Bacteria;Firmicutes;Clostridia;Clostridiales;Ruminococcaceae;Pseudoflavonifractor;PAC001131_s | 3 | 0.0067 |
| PAC001092_g PAC001381_s | Bacteria;Firmicutes;Clostridia;Clostridiales;Lachnospiraceae;PAC001092_g;PAC001381_s | 3 | 0.0067 |
| PAC000692_g PAC001577_s | Bacteria;Firmicutes;Clostridia;Clostridiales;Lachnospiraceae;PAC000692_g;PAC001577_s | 3 | 0.0067 |
| Anaerotignum PAC001709_s | Bacteria;Firmicutes;Clostridia;Clostridiales;Lachnospiraceae;Anaerotignum;PAC001709_s | 3 | 0.0067 |
| PAC001103_g PAC001724_s group | Bacteria;Firmicutes;Clostridia;Clostridiales;Lachnospiraceae;PAC001103_g;PAC001724_s group | 3 | 0.0067 |
| PAC001516_g PAC001741_s | Bacteria;Firmicutes;Clostridia;Clostridiales;Lachnospiraceae;PAC001516_g;PAC001741_s | 3 | 0.0067 |
| PAC000692_g PAC001760_s | Bacteria;Firmicutes;Clostridia;Clostridiales;Lachnospiraceae;PAC000692_g;PAC001760_s | 3 | 0.0067 |
| KE159571_g PAC002468_s | Bacteria;Firmicutes;Clostridia;Clostridiales;Lachnospiraceae;KE159571_g;PAC002468_s | 3 | 0.0067 |
| Frisingicoccus PAC002470_s | Bacteria;Firmicutes;Clostridia;Clostridiales;Lachnospiraceae;Frisingicoccus;PAC002470_s | 3 | 0.0067 |
| PAC001296_g_uc | Bacteria;Firmicutes;Clostridia;Clostridiales;Lachnospiraceae;PAC001296_g;PAC001296_g_uc | 3 | 0.0067 |
| Bacteroides oleiciplenus | Bacteria;Bacteroidetes;Bacteroidia;Bacteroidales;Bacteroidaceae;Bacteroides;Bacteroides oleiciplenus | 2 | 0.0045 |
| Eubacterium plexicaudatum | Bacteria;Firmicutes;Clostridia;Clostridiales;Lachnospiraceae;Eubacterium_g6;Eubacterium plexicaudatum | 2 | 0.0045 |
| Herbiconiux ginsengi group | Bacteria;Actinobacteria;Actinobacteria_c;Micrococcales;Microbacteriaceae;Herbiconiux;Herbiconiux ginsengi group | 2 | 0.0045 |
| Acutalibacter AB622826_s | Bacteria;Firmicutes;Clostridia;Clostridiales;Ruminococcaceae;Acutalibacter;AB622826_s | 2 | 0.0045 |
| PAC000664_g AB626958_s | Bacteria;Firmicutes;Clostridia;Clostridiales;Lachnospiraceae;PAC000664_g;AB626958_s | 2 | 0.0045 |
| LLKB_g AF371672_s | Bacteria;Firmicutes;Clostridia;Clostridiales;Lachnospiraceae;LLKB_g;AF371672_s | 2 | 0.0045 |
| Caproiciproducens DQ777923_s | Bacteria;Firmicutes;Clostridia;Clostridiales;Ruminococcaceae;Caproiciproducens;DQ777923_s | 2 | 0.0045 |
| Oscillibacter DQ806484_s | Bacteria;Firmicutes;Clostridia;Clostridiales;Ruminococcaceae;Oscillibacter;DQ806484_s | 2 | 0.0045 |
| KE159571_g DQ815838_s | Bacteria;Firmicutes;Clostridia;Clostridiales;Lachnospiraceae;KE159571_g;DQ815838_s | 2 | 0.0045 |
| Clostridium_g21 EU455375_s | Bacteria;Firmicutes;Clostridia;Clostridiales;Lachnospiraceae;Clostridium_g21;EU455375_s | 2 | 0.0045 |
| PAC000196_g EU466069_s | Bacteria;Firmicutes;Clostridia;Clostridiales;Lachnospiraceae;PAC000196_g;EU466069_s | 2 | 0.0045 |
| Acetatifactor EU508111_s | Bacteria;Firmicutes;Clostridia;Clostridiales;Lachnospiraceae;Acetatifactor;EU508111_s | 2 | 0.0045 |
| Clostridium_g21 EU509115_s | Bacteria;Firmicutes;Clostridia;Clostridiales;Lachnospiraceae;Clostridium_g21;EU509115_s | 2 | 0.0045 |
| PAC001573_g EU509323_s | Bacteria;Firmicutes;Clostridia;Clostridiales;Mogibacterium_f;PAC001573_g;EU509323_s | 2 | 0.0045 |
| Eubacterium_g23 EU509480_s | Bacteria;Firmicutes;Clostridia;Clostridiales;Ruminococcaceae;Eubacterium_g23;EU509480_s | 2 | 0.0045 |
| Pseudoflavonifractor EU542517_s | Bacteria;Firmicutes;Clostridia;Clostridiales;Ruminococcaceae;Pseudoflavonifractor;EU542517_s | 2 | 0.0045 |
| PAC001165_g EU622677_s | Bacteria;Firmicutes;Clostridia;Clostridiales;Lachnospiraceae;PAC001165_g;EU622677_s | 2 | 0.0045 |
| PAC001219_g HM124151_s | Bacteria;Firmicutes;Clostridia;Clostridiales;Christensenellaceae;PAC001219_g;HM124151_s | 2 | 0.0045 |
| KE159797_g KE159797_s | Bacteria;Firmicutes;Clostridia;Clostridiales;Lachnospiraceae;KE159797_g;KE159797_s | 2 | 0.0045 |
| PAC001141_g PAC001141_s | Bacteria;Firmicutes;Clostridia;Clostridiales;Christensenellaceae;PAC001141_g;PAC001141_s | 2 | 0.0045 |
| PAC001165_g PAC001165_s | Bacteria;Firmicutes;Clostridia;Clostridiales;Lachnospiraceae;PAC001165_g;PAC001165_s | 2 | 0.0045 |
| Frisingicoccus PAC001364_s | Bacteria;Firmicutes;Clostridia;Clostridiales;Lachnospiraceae;Frisingicoccus;PAC001364_s | 2 | 0.0045 |
| Frisingicoccus PAC001481_s | Bacteria;Firmicutes;Clostridia;Clostridiales;Lachnospiraceae;Frisingicoccus;PAC001481_s | 2 | 0.0045 |
| Acutalibacter PAC001594_s | Bacteria;Firmicutes;Clostridia;Clostridiales;Ruminococcaceae;Acutalibacter;PAC001594_s | 2 | 0.0045 |
| Oscillibacter PAC001684_s | Bacteria;Firmicutes;Clostridia;Clostridiales;Ruminococcaceae;Oscillibacter;PAC001684_s | 2 | 0.0045 |
| PAC000661_g PAC002012_s | Bacteria;Firmicutes;Clostridia;Clostridiales;Ruminococcaceae;PAC000661_g;PAC002012_s | 2 | 0.0045 |
| PAC002042_g PAC002042_s | Bacteria;Firmicutes;Clostridia;Clostridiales;Lachnospiraceae;PAC002042_g;PAC002042_s | 2 | 0.0045 |
| KE159605_g PAC002401_s group | Bacteria;Firmicutes;Clostridia;Clostridiales;Lachnospiraceae;KE159605_g;PAC002401_s group | 2 | 0.0045 |
| PAC002471_g PAC002471_s | Bacteria;Firmicutes;Clostridia;Clostridiales;Lachnospiraceae;PAC002471_g;PAC002471_s | 2 | 0.0045 |
| Parasutterella PAC002476_s | Bacteria;Proteobacteria;Betaproteobacteria;Burkholderiales;Sutterellaceae;Parasutterella;PAC002476_s | 2 | 0.0045 |
| Clostridium_g21 PAC002492_s | Bacteria;Firmicutes;Clostridia;Clostridiales;Lachnospiraceae;Clostridium_g21;PAC002492_s | 2 | 0.0045 |
| PAC002528_g PAC002528_s | Bacteria;Actinobacteria;Coriobacteriia;Coriobacteriales;Coriobacteriaceae;PAC002528_g;PAC002528_s | 2 | 0.0045 |
| Caproiciproducens_uc | Bacteria;Firmicutes;Clostridia;Clostridiales;Ruminococcaceae;Caproiciproducens;Caproiciproducens_uc | 2 | 0.0045 |
| PAC000197_g_uc | Bacteria;Tenericutes;Mollicutes;PAC001057_o;PAC000197_f;PAC000197_g;PAC000197_g_uc | 2 | 0.0045 |
| PAC000673_g_uc | Bacteria;Firmicutes;Clostridia;Clostridiales;Lachnospiraceae;PAC000673_g;PAC000673_g_uc | 2 | 0.0045 |
| PAC001165_g_uc | Bacteria;Firmicutes;Clostridia;Clostridiales;Lachnospiraceae;PAC001165_g;PAC001165_g_uc | 2 | 0.0045 |
| Clostridiales_uc | Bacteria;Firmicutes;Clostridia;Clostridiales;Clostridiales_uc | 1 | 0.0022 |
| Bacillus subtilis group | Bacteria;Firmicutes;Bacilli;Bacillales;Bacillaceae;Bacillus;Bacillus subtilis group | 1 | 0.0022 |
| Enterorhabdus mucosicola | Bacteria;Actinobacteria;Coriobacteriia;Coriobacteriales;Coriobacteriaceae;Enterorhabdus;Enterorhabdus mucosicola | 1 | 0.0022 |
| Intestinimonas butyriciproducens | Bacteria;Firmicutes;Clostridia;Clostridiales;Ruminococcaceae;Pseudoflavonifractor;Intestinimonas butyriciproducens | 1 | 0.0022 |
| Lactobacillus gasseri group | Bacteria;Firmicutes;Bacilli;Lactobacillales;Lactobacillaceae;Lactobacillus;Lactobacillus gasseri group | 1 | 0.0022 |
| Lysinibacillus xylanilyticus group | Bacteria;Firmicutes;Bacilli;Bacillales;Planococcaceae;Lysinibacillus;Lysinibacillus xylanilyticus group | 1 | 0.0022 |
| Staphylococcus sciuri group | Bacteria;Firmicutes;Bacilli;Bacillales;Staphylococcaceae;Staphylococcus;Staphylococcus sciuri group | 1 | 0.0022 |
| Kineothrix AB606257_s | Bacteria;Firmicutes;Clostridia;Clostridiales;Lachnospiraceae;Kineothrix;AB606257_s | 1 | 0.0022 |
| PAC000186_g EF406572_s | Bacteria;Bacteroidetes;Bacteroidia;Bacteroidales;Muribaculaceae;PAC000186_g;EF406572_s | 1 | 0.0022 |
| PAC000664_g EU622646_s | Bacteria;Firmicutes;Clostridia;Clostridiales;Lachnospiraceae;PAC000664_g;EU622646_s | 1 | 0.0022 |
| Pseudoflavonifractor EU794285_s | Bacteria;Firmicutes;Clostridia;Clostridiales;Ruminococcaceae;Pseudoflavonifractor;EU794285_s | 1 | 0.0022 |
| Acetatifactor HM123975_s | Bacteria;Firmicutes;Clostridia;Clostridiales;Lachnospiraceae;Acetatifactor;HM123975_s | 1 | 0.0022 |
| PAC000692_g HM124219_s | Bacteria;Firmicutes;Clostridia;Clostridiales;Lachnospiraceae;PAC000692_g;HM124219_s | 1 | 0.0022 |
| Blautia LN913006_s group | Bacteria;Firmicutes;Clostridia;Clostridiales;Lachnospiraceae;Blautia;LN913006_s group | 1 | 0.0022 |
| Clostridium_g24 PAC000184_s | Bacteria;Firmicutes;Clostridia;Clostridiales;Lachnospiraceae;Clostridium_g24;PAC000184_s | 1 | 0.0022 |
| PAC000186_g PAC001064_s | Bacteria;Bacteroidetes;Bacteroidia;Bacteroidales;Muribaculaceae;PAC000186_g;PAC001064_s | 1 | 0.0022 |
| PAC000661_g PAC001095_s | Bacteria;Firmicutes;Clostridia;Clostridiales;Ruminococcaceae;PAC000661_g;PAC001095_s | 1 | 0.0022 |
| PAC001097_g PAC001097_s | Bacteria;Firmicutes;Clostridia;Clostridiales;Lachnospiraceae;PAC001097_g;PAC001097_s | 1 | 0.0022 |
| PAC001200_g PAC001200_s | Bacteria;Firmicutes;Clostridia;Clostridiales;Lachnospiraceae;PAC001200_g;PAC001200_s | 1 | 0.0022 |
| PAC001219_g PAC001280_s | Bacteria;Firmicutes;Clostridia;Clostridiales;Christensenellaceae;PAC001219_g;PAC001280_s | 1 | 0.0022 |
| PAC001360_g PAC001371_s | Bacteria;Firmicutes;Clostridia;Clostridiales;Christensenellaceae;PAC001360_g;PAC001371_s | 1 | 0.0022 |
| Clostridium_g24 PAC001376_s | Bacteria;Firmicutes;Clostridia;Clostridiales;Lachnospiraceae;Clostridium_g24;PAC001376_s | 1 | 0.0022 |
| PAC001116_g PAC001589_s | Bacteria;Firmicutes;Clostridia;Clostridiales;Lachnospiraceae;PAC001116_g;PAC001589_s | 1 | 0.0022 |
| PAC001609_g PAC001650_s | Bacteria;Firmicutes;Clostridia;Clostridiales;Mogibacterium_f;PAC001609_g;PAC001650_s | 1 | 0.0022 |
| PAC001063_g PAC001664_s | Bacteria;Bacteroidetes;Bacteroidia;Bacteroidales;Muribaculaceae;PAC001063_g;PAC001664_s | 1 | 0.0022 |
| PAC001573_g PAC001808_s | Bacteria;Firmicutes;Clostridia;Clostridiales;Mogibacterium_f;PAC001573_g;PAC001808_s | 1 | 0.0022 |
| PAC001092_g PAC001811_s | Bacteria;Firmicutes;Clostridia;Clostridiales;Lachnospiraceae;PAC001092_g;PAC001811_s | 1 | 0.0022 |
| PAC001908_g PAC001908_s | Bacteria;Firmicutes;Clostridia;Clostridiales;Ruminococcaceae;PAC001908_g;PAC001908_s | 1 | 0.0022 |
| PAC001092_g PAC002030_s | Bacteria;Firmicutes;Clostridia;Clostridiales;Lachnospiraceae;PAC001092_g;PAC002030_s | 1 | 0.0022 |
| Paludicola PAC002097_s | Bacteria;Firmicutes;Clostridia;Clostridiales;Ruminococcaceae;Paludicola;PAC002097_s | 1 | 0.0022 |
| PAC002178_g PAC002178_s | Bacteria;Firmicutes;Clostridia;Clostridiales;Lachnospiraceae;PAC002178_g;PAC002178_s | 1 | 0.0022 |
| PAC002314_g PAC002314_s | Bacteria;Firmicutes;Erysipelotrichi;Erysipelotrichales;Erysipelotrichaceae;PAC002314_g;PAC002314_s | 1 | 0.0022 |
| PAC001144_g PAC002359_s | Bacteria;Firmicutes;Clostridia;Clostridiales;Ruminococcaceae;PAC001144_g;PAC002359_s | 1 | 0.0022 |
| PAC001308_g_uc | Bacteria;Firmicutes;Clostridia;Clostridiales;Lachnospiraceae;PAC001308_g;PAC001308_g_uc | 1 | 0.0022 |
| PAC001402_g_uc | Bacteria;Firmicutes;Clostridia;Clostridiales;Ruminococcaceae;PAC001402_g;PAC001402_g_uc | 1 | 0.0022 |
| PAC001516_g_uc | Bacteria;Firmicutes;Clostridia;Clostridiales;Lachnospiraceae;PAC001516_g;PAC001516_g_uc | 1 | 0.0022 |

**Set 2_0.5%_S: Day 10_heme-SCP 0.5%_Species**

| Taxon name | Taxonomy | Count | Proportion(%) |
|---|---|---|---|
| KE159600_g KE159600_s | Bacteria;Firmicutes;Clostridia;Clostridiales;Lachnospiraceae;KE159600_g;KE159600_s | 5431 | 13.6987 |
| Bacteroides HM124113_s | Bacteria;Bacteroidetes;Bacteroidia;Bacteroidales;Bacteroidaceae;Bacteroides;HM124113_s | 2086 | 5.2616 |
| Bacteroides_uc | Bacteria;Bacteroidetes;Bacteroidia;Bacteroidales;Bacteroidaceae;Bacteroides;Bacteroides_uc | 1743 | 4.3964 |
| Oscillibacter PAC001186_s group | Bacteria;Firmicutes;Clostridia;Clostridiales;Ruminococcaceae;Oscillibacter;PAC001186_s group | 1357 | 3.4228 |
| Flintibacter butyricus group | Bacteria;Firmicutes;Clostridia;Clostridiales;Ruminococcaceae;Pseudoflavonifractor;Flintibacter butyricus group | 1287 | 3.2462 |
| Anaerotignum PAC001740_s | Bacteria;Firmicutes;Clostridia;Clostridiales;Lachnospiraceae;Anaerotignum;PAC001740_s | 1167 | 2.9436 |
| PAC001516_g PAC002384_s | Bacteria;Firmicutes;Clostridia;Clostridiales;Lachnospiraceae;PAC001516_g;PAC002384_s | 1146 | 2.8906 |
| KE159571_g PAC002009_s group | Bacteria;Firmicutes;Clostridia;Clostridiales;Lachnospiraceae;KE159571_g;PAC002009_s group | 1140 | 2.8754 |
| Oscillibacter PAC002372_s | Bacteria;Firmicutes;Clostridia;Clostridiales;Ruminococcaceae;Oscillibacter;PAC002372_s | 1070 | 2.6989 |
| Bilophila wadsworthia | Bacteria;Proteobacteria;Deltaproteobacteria;Desulfovibrionales;Desulfovibrionaceae;Bilophila;Bilophila wadsworthia | 1030 | 2.598 |
| Eubacterium_g23 PAC001121_s group | Bacteria;Firmicutes;Clostridia;Clostridiales;Ruminococcaceae;Eubacterium_g23;PAC001121_s group | 1010 | 2.5475 |
| PAC001516_g PAC001516_s | Bacteria;Firmicutes;Clostridia;Clostridiales;Lachnospiraceae;PAC001516_g;PAC001516_s | 940 | 2.371 |
| PAC002482_g EF603735_s | Bacteria;Bacteroidetes;Bacteroidia;Bacteroidales;AC160630_f;PAC002482_g;EF603735_s | 904 | 2.2802 |
| Alistipes PAC001061_s | Bacteria;Bacteroidetes;Bacteroidia;Bacteroidales;Rikenellaceae;Alistipes;PAC001061_s | 899 | 2.2676 |
| PAC001287_g PAC001287_s | Bacteria;Firmicutes;Clostridia;Clostridiales;Lachnospiraceae;PAC001287_g;PAC001287_s | 837 | 2.1112 |
| Oscillibacter PAC001501_s | Bacteria;Firmicutes;Clostridia;Clostridiales;Ruminococcaceae;Oscillibacter;PAC001501_s | 682 | 1.7202 |
| Acetatifactor PAC001463_s | Bacteria;Firmicutes;Clostridia;Clostridiales;Lachnospiraceae;Acetatifactor;PAC001463_s | 615 | 1.5512 |
| Bacteroides acidifaciens | Bacteria;Bacteroidetes;Bacteroidia;Bacteroidales;Bacteroidaceae;Bacteroides;Bacteroides acidifaciens | 602 | 1.5184 |
| Bacteroides stercorirosoris | Bacteria;Bacteroidetes;Bacteroidia;Bacteroidales;Bacteroidaceae;Bacteroides;Bacteroides stercorirosoris | 579 | 1.4604 |
| PAC001141_g PAC001141_s | Bacteria;Firmicutes;Clostridia;Clostridiales;Christensenellaceae;PAC001141_g;PAC001141_s | 539 | 1.3595 |
| Corynebacterium glutamicum | Bacteria;Actinobacteria;Actinobacteria_c;Corynebacteriales;Corynebacteriaceae;Corynebacterium;Corynebacterium glutamicum | 515 | 1.299 |
| Bacteroides vulgatus | Bacteria;Bacteroidetes;Bacteroidia;Bacteroidales;Bacteroidaceae;Bacteroides;Bacteroides vulgatus | 504 | 1.2713 |
| Acetatifactor PAC001801_s | Bacteria;Firmicutes;Clostridia;Clostridiales;Lachnospiraceae;Acetatifactor;PAC001801_s | 473 | 1.1931 |
| Clostridium_g21 KE159781_s | Bacteria;Firmicutes;Clostridia;Clostridiales;Lachnospiraceae;Clostridium_g21;KE159781_s | 460 | 1.1603 |
| PAC001112_g PAC001267_s | Bacteria;Bacteroidetes;Bacteroidia;Bacteroidales;Muribaculaceae;PAC001112_g;PAC001267_s | 449 | 1.1325 |
| Acetatifactor PAC001773_s | Bacteria;Firmicutes;Clostridia;Clostridiales;Lachnospiraceae;Acetatifactor;PAC001773_s | 407 | 1.0266 |
| PAC001402_g PAC001764_s | Bacteria;Firmicutes;Clostridia;Clostridiales;Ruminococcaceae;PAC001402_g;PAC001764_s | 375 | 0.9459 |
| Lactococcus lactis group | Bacteria;Firmicutes;Bacilli;Lactobacillales;Streptococcaceae;Lactococcus;Lactococcus lactis group | 370 | 0.9333 |
| PAC000664_g PAC001104_s | Bacteria;Firmicutes;Clostridia;Clostridiales;Lachnospiraceae;PAC000664_g;PAC001104_s | 359 | 0.9055 |
| PAC001544_g PAC001544_s | Bacteria;Firmicutes;Clostridia;Clostridiales;Lachnospiraceae;PAC001544_g;PAC001544_s | 354 | 0.8929 |
| Pseudoflavonifractor PAC001549_s | Bacteria;Firmicutes;Clostridia;Clostridiales;Ruminococcaceae;Pseudoflavonifractor;PAC001549_s | 343 | 0.8652 |
| Pseudoflavonifractor PAC001379_s | Bacteria;Firmicutes;Clostridia;Clostridiales;Ruminococcaceae;Pseudoflavonifractor;PAC001379_s | 330 | 0.8324 |
| Pseudoflavonifractor_uc | Bacteria;Firmicutes;Clostridia;Clostridiales;Ruminococcaceae;Pseudoflavonifractor;Pseudoflavonifractor_uc | 322 | 0.8122 |
| Pseudoflavonifractor PAC001574_s group | Bacteria;Firmicutes;Clostridia;Clostridiales;Ruminococcaceae;Pseudoflavonifractor;PAC001574_s group | 317 | 0.7996 |
| PAC000198_g PAC001075_s | Bacteria;Bacteroidetes;Bacteroidia;Bacteroidales;Muribaculaceae;PAC000198_g;PAC001075_s | 311 | 0.7844 |
| Anaerotruncus KE159677_s | Bacteria;Firmicutes;Clostridia;Clostridiales;Ruminococcaceae;Anaerotruncus;KE159677_s | 286 | 0.7214 |
| KE159571_g PAC001776_s group | Bacteria;Firmicutes;Clostridia;Clostridiales;Lachnospiraceae;KE159571_g;PAC001776_s group | 282 | 0.7113 |
| PAC002375_g PAC002375_s | Bacteria;Firmicutes;Clostridia;Clostridiales;Lachnospiraceae;PAC002375_g;PAC002375_s | 258 | 0.6508 |
| Oscillibacter PAC001584_s group | Bacteria;Firmicutes;Clostridia;Clostridiales;Ruminococcaceae;Oscillibacter;PAC001584_s group | 253 | 0.6381 |
| PAC001068_g PAC001068_s | Bacteria;Bacteroidetes;Bacteroidia;Bacteroidales;Muribaculaceae;PAC001068_g;PAC001068_s | 209 | 0.5272 |
| Clostridium_g21 PAC001294_s | Bacteria;Firmicutes;Clostridia;Clostridiales;Lachnospiraceae;Clostridium_g21;PAC001294_s | 201 | 0.507 |
| Pseudoflavonifractor PAC001543_s | Bacteria;Firmicutes;Clostridia;Clostridiales;Ruminococcaceae;Pseudoflavonifractor;PAC001543_s | 180 | 0.454 |
| PAC001066_g PAC001066_s | Bacteria;Bacteroidetes;Bacteroidia;Bacteroidales;Muribaculaceae;PAC001066_g;PAC001066_s | 172 | 0.4338 |
| PAC001074_g PAC001074_s | Bacteria;Bacteroidetes;Bacteroidia;Bacteroidales;Muribaculaceae;PAC001074_g;PAC001074_s | 170 | 0.4288 |
| PAC001063_g PAC001063_s | Bacteria;Bacteroidetes;Bacteroidia;Bacteroidales;Muribaculaceae;PAC001063_g;PAC001063_s | 166 | 0.4187 |
| Pseudoflavonifractor KI535319_s | Bacteria;Firmicutes;Clostridia;Clostridiales;Ruminococcaceae;Pseudoflavonifractor;KI535319_s | 161 | 0.4061 |
| PAC000661_g_uc | Bacteria;Firmicutes;Clostridia;Clostridiales;Ruminococcaceae;PAC000661_g;PAC000661_g_uc | 154 | 0.3884 |
| PAC001402_g PAC001402_s | Bacteria;Firmicutes;Clostridia;Clostridiales;Ruminococcaceae;PAC001402_g;PAC001402_s | 152 | 0.3834 |
| Oscillibacter PAC002110_s | Bacteria;Firmicutes;Clostridia;Clostridiales;Ruminococcaceae;Oscillibacter;PAC002110_s | 146 | 0.3683 |
| Lactobacillus murinus group | Bacteria;Firmicutes;Bacilli;Lactobacillales;Lactobacillaceae;Lactobacillus;Lactobacillus murinus group | 141 | 0.3556 |
| PAC001103_g_uc | Bacteria;Firmicutes;Clostridia;Clostridiales;Lachnospiraceae;PAC001103_g;PAC001103_g_uc | 141 | 0.3556 |
| Pseudoflavonifractor PAC001550_s | Bacteria;Firmicutes;Clostridia;Clostridiales;Ruminococcaceae;Pseudoflavonifractor;PAC001550_s | 131 | 0.3304 |
| Frisingicoccus PAC001744_s group | Bacteria;Firmicutes;Clostridia;Clostridiales;Lachnospiraceae;Frisingicoccus;PAC001744_s group | 131 | 0.3304 |
| PAC001516_g PAC001741_s | Bacteria;Firmicutes;Clostridia;Clostridiales;Lachnospiraceae;PAC001516_g;PAC001741_s | 125 | 0.3153 |
| Caproiciproducens EU622658_s | Bacteria;Firmicutes;Clostridia;Clostridiales;Ruminococcaceae;Caproiciproducens;EU622658_s | 123 | 0.3102 |
| PAC001165_g PAC001384_s | Bacteria;Firmicutes;Clostridia;Clostridiales;Lachnospiraceae;PAC001165_g;PAC001384_s | 120 | 0.3027 |
| KE159605_g KE159605_s | Bacteria;Firmicutes;Clostridia;Clostridiales;Lachnospiraceae;KE159605_g;KE159605_s | 115 | 0.2901 |
| Clostridium_g21 PAC001085_s | Bacteria;Firmicutes;Clostridia;Clostridiales;Lachnospiraceae;Clostridium_g21;PAC001085_s | 112 | 0.2825 |
| Acetatifactor_uc | Bacteria;Firmicutes;Clostridia;Clostridiales;Lachnospiraceae;Acetatifactor;Acetatifactor_uc | 112 | 0.2825 |
| PAC001400_g PAC001400_s | Bacteria;Firmicutes;Clostridia;Clostridiales;Peptococcaceae;PAC001400_g;PAC001400_s | 109 | 0.2749 |
| PAC001228_g PAC002027_s | Bacteria;Firmicutes;Clostridia;Clostridiales;Lachnospiraceae;PAC001228_g;PAC002027_s | 107 | 0.2699 |
| Oscillibacter PAC001188_s | Bacteria;Firmicutes;Clostridia;Clostridiales;Ruminococcaceae;Oscillibacter;PAC001188_s | 99 | 0.2497 |
| PAC001221_g PAC001221_s | Bacteria;Firmicutes;Clostridia;Clostridiales;Dehalobacterium_f;PAC001221_g;PAC001221_s | 99 | 0.2497 |
| KE159797_g_uc | Bacteria;Firmicutes;Clostridia;Clostridiales;Lachnospiraceae;KE159797_g;KE159797_g_uc | 98 | 0.2472 |
| KE159571_g PAC002442_s | Bacteria;Firmicutes;Clostridia;Clostridiales;Lachnospiraceae;KE159571_g;PAC002442_s | 94 | 0.2371 |
| Oscillibacter EU454374_s | Bacteria;Firmicutes;Clostridia;Clostridiales;Ruminococcaceae;Oscillibacter;EU454374_s | 91 | 0.2295 |
| PAC001138_g PAC001518_s | Bacteria;Firmicutes;Clostridia;Clostridiales;Lachnospiraceae;PAC001138_g;PAC001518_s | 90 | 0.227 |
| Clostridium_g21 PAC002512_s group | Bacteria;Firmicutes;Clostridia;Clostridiales;Lachnospiraceae;Clostridium_g21;PAC002512_s group | 78 | 0.1967 |
| PAC001385_g PAC001727_s group | Bacteria;Firmicutes;Clostridia;Clostridiales;Lachnospiraceae;PAC001385_g;PAC001727_s group | 77 | 0.1942 |
| Oscillibacter PAC001522_s | Bacteria;Firmicutes;Clostridia;Clostridiales;Ruminococcaceae;Oscillibacter;PAC001522_s | 75 | 0.1892 |
| Mucispirillum schaedleri | Bacteria;Deferribacteres;Deferribacteres_c;Deferribacterales;Deferribacteraceae;Mucispirillum;Mucispirillum schaedleri | 73 | 0.1841 |
| Parabacteroides goldsteinii | Bacteria;Bacteroidetes;Bacteroidia;Bacteroidales;Porphyromonadaceae;Parabacteroides;Parabacteroides goldsteinii | 70 | 0.1766 |
| Oscillibacter PAC001369_s | Bacteria;Firmicutes;Clostridia;Clostridiales;Ruminococcaceae;Oscillibacter;PAC001369_s | 69 | 0.174 |
| PAC001402_g PAC001748_s | Bacteria;Firmicutes;Clostridia;Clostridiales;Ruminococcaceae;PAC001402_g;PAC001748_s | 68 | 0.1715 |
| PAC000664_g PAC001083_s | Bacteria;Firmicutes;Clostridia;Clostridiales;Lachnospiraceae;PAC000664_g;PAC001083_s | 65 | 0.164 |
| Eubacterium_g6 PAC001586_s | Bacteria;Firmicutes;Clostridia;Clostridiales;Lachnospiraceae;Eubacterium_g6;PAC001586_s | 65 | 0.164 |
| PAC001360_g PAC001371_s | Bacteria;Firmicutes;Clostridia;Clostridiales;Christensenellaceae;PAC001360_g;PAC001371_s | 64 | 0.1614 |
| PAC001043_g PAC002511_s group | Bacteria;Firmicutes;Clostridia;Clostridiales;Lachnospiraceae;PAC001043_g;PAC002511_s group | 64 | 0.1614 |
| PAC001778_g PAC001778_s | Bacteria;Firmicutes;Clostridia;Clostridiales;Ruminococcaceae;PAC001778_g;PAC001778_s | 63 | 0.1589 |
| KE159600_g EU511112_s | Bacteria;Firmicutes;Clostridia;Clostridiales;Lachnospiraceae;KE159600_g;EU511112_s | 62 | 0.1564 |
| PAC001092_g PAC001668_s | Bacteria;Firmicutes;Clostridia;Clostridiales;Lachnospiraceae;PAC001092_g;PAC001668_s | 61 | 0.1539 |
| Frisingicoccus EF409953_s | Bacteria;Firmicutes;Clostridia;Clostridiales;Lachnospiraceae;Frisingicoccus;EF409953_s | 60 | 0.1513 |
| Oscillibacter PAC001156_s | Bacteria;Firmicutes;Clostridia;Clostridiales;Ruminococcaceae;Oscillibacter;PAC001156_s | 60 | 0.1513 |
| Bacteroides uniformis | Bacteria;Bacteroidetes;Bacteroidia;Bacteroidales;Bacteroidaceae;Bacteroides;Bacteroides uniformis | 57 | 0.1438 |
| Sporobacter PAC001096_s | Bacteria;Firmicutes;Clostridia;Clostridiales;Ruminococcaceae;Sporobacter;PAC001096_s | 57 | 0.1438 |
| Oscillibacter PAC001526_s | Bacteria;Firmicutes;Clostridia;Clostridiales;Ruminococcaceae;Oscillibacter;PAC001526_s | 57 | 0.1438 |
| PAC000748_g PAC001099_s | Bacteria;Firmicutes;Clostridia;Clostridiales;Ruminococcaceae;PAC000748_g;PAC001099_s | 51 | 0.1286 |
| PAC001524_g PAC001524_s | Bacteria;Firmicutes;Clostridia;Clostridiales;Lachnospiraceae;PAC001524_g;PAC001524_s | 50 | 0.1261 |
| Bacteroides intestinalis | Bacteria;Bacteroidetes;Bacteroidia;Bacteroidales;Bacteroidaceae;Bacteroides;Bacteroides intestinalis | 46 | 0.116 |
| PAC001308_g DQ815557_s | Bacteria;Firmicutes;Clostridia;Clostridiales;Lachnospiraceae;PAC001308_g;DQ815557_s | 45 | 0.1135 |
| PAC000661_g PAC002012_s | Bacteria;Firmicutes;Clostridia;Clostridiales;Ruminococcaceae;PAC000661_g;PAC002012_s | 45 | 0.1135 |
| Massilioclostridium PAC001521_s | Bacteria;Firmicutes;Clostridia;Clostridiales;Ruminococcaceae;Massilioclostridium;PAC001521_s | 44 | 0.111 |
| PAC001457_g PAC001495_s group | Bacteria;Firmicutes;Clostridia;Clostridiales;Lachnospiraceae;PAC001457_g;PAC001495_s group | 43 | 0.1085 |
| Lachnospiraceae_uc | Bacteria;Firmicutes;Clostridia;Clostridiales;Lachnospiraceae;Lachnospiraceae_uc | 42 | 0.1059 |
| Pseudoflavonifractor PAC001087_s | Bacteria;Firmicutes;Clostridia;Clostridiales;Ruminococcaceae;Pseudoflavonifractor;PAC001087_s | 42 | 0.1059 |
| Acetatifactor muris | Bacteria;Firmicutes;Clostridia;Clostridiales;Lachnospiraceae;Acetatifactor;Acetatifactor muris | 40 | 0.1009 |
| PAC001402_g PAC001561_s | Bacteria;Firmicutes;Clostridia;Clostridiales;Ruminococcaceae;PAC001402_g;PAC001561_s | 40 | 0.1009 |
| Paludicola PAC001507_s | Bacteria;Firmicutes;Clostridia;Clostridiales;Ruminococcaceae;Paludicola;PAC001507_s | 37 | 0.0933 |
| Bacillus cereus group | Bacteria;Firmicutes;Bacilli;Bacillales;Bacillaceae;Bacillus;Bacillus cereus group | 36 | 0.0908 |
| Bacteroides caecimuris | Bacteria;Bacteroidetes;Bacteroidia;Bacteroidales;Bacteroidaceae;Bacteroides;Bacteroides caecimuris | 36 | 0.0908 |
| PAC001103_g PAC001361_s | Bacteria;Firmicutes;Clostridia;Clostridiales;Lachnospiraceae;PAC001103_g;PAC001361_s | 36 | 0.0908 |
| PAC001092_g PAC001587_s | Bacteria;Firmicutes;Clostridia;Clostridiales;Lachnospiraceae;PAC001092_g;PAC001587_s | 36 | 0.0908 |
| PAC001062_g PAC001062_s | Bacteria;Bacteroidetes;Bacteroidia;Bacteroidales;Muribaculaceae;PAC001062_g;PAC001062_s | 35 | 0.0883 |
| Acutalibacter PAC002354_s | Bacteria;Firmicutes;Clostridia;Clostridiales;Ruminococcaceae;Acutalibacter;PAC002354_s | 35 | 0.0883 |
| Eubacterium_g6 PAC001759_s | Bacteria;Firmicutes;Clostridia;Clostridiales;Lachnospiraceae;Eubacterium_g6;PAC001759_s | 34 | 0.0858 |
| LARJ_g PAC001927_s | Bacteria;Proteobacteria;Alphaproteobacteria;Rhodospirillales;Rhodospirillaceae;LARJ_g;PAC001927_s | 33 | 0.0832 |
| Arthromitus AP012202_s group | Bacteria;Firmicutes;Clostridia;Clostridiales;Clostridiaceae;Arthromitus;AP012202_s group | 32 | 0.0807 |
| Oscillibacter PAC001502_s | Bacteria;Firmicutes;Clostridia;Clostridiales;Ruminococcaceae;Oscillibacter;PAC001502_s | 31 | 0.0782 |
| KE159571_g PAC001705_s | Bacteria;Firmicutes;Clostridia;Clostridiales;Lachnospiraceae;KE159571_g;PAC001705_s | 31 | 0.0782 |
| Harryflintia PAC002149_s | Bacteria;Firmicutes;Clostridia;Clostridiales;Ruminococcaceae;Harryflintia;PAC002149_s | 31 | 0.0782 |
| Clostridium_g21 EU506235_s | Bacteria;Firmicutes;Clostridia;Clostridiales;Lachnospiraceae;Clostridium_g21;EU506235_s | 29 | 0.0731 |
| PAC000661_g PAC001536_s | Bacteria;Firmicutes;Clostridia;Clostridiales;Ruminococcaceae;PAC000661_g;PAC001536_s | 29 | 0.0731 |
| Escherichia coli group | Bacteria;Proteobacteria;Gammaproteobacteria;Enterobacterales;Enterobacteriaceae;Escherichia;Escherichia coli group | 28 | 0.0706 |
| Caproiciproducens PAC001505_s | Bacteria;Firmicutes;Clostridia;Clostridiales;Ruminococcaceae;Caproiciproducens;PAC001505_s | 28 | 0.0706 |
| PAC001066_g_uc | Bacteria;Bacteroidetes;Bacteroidia;Bacteroidales;Muribaculaceae;PAC001066_g;PAC001066_g_uc | 28 | 0.0706 |
| PAC000661_g PAC001716_s | Bacteria;Firmicutes;Clostridia;Clostridiales;Ruminococcaceae;PAC000661_g;PAC001716_s | 27 | 0.0681 |
| Eubacterium_g17 PAC001119_s | Bacteria;Firmicutes;Clostridia;Clostridiales;Lachnospiraceae;Eubacterium_g17;PAC001119_s | 26 | 0.0656 |
| PAC001313_g PAC001313_s | Bacteria;Firmicutes;Clostridia;Clostridiales;Ruminococcaceae;PAC001313_g;PAC001313_s | 26 | 0.0656 |
| PAC002169_g PAC002169_s | Bacteria;Firmicutes;Clostridia;Clostridiales;Christensenellaceae;PAC002169_g;PAC002169_s | 26 | 0.0656 |
| PAC000661_g PAC001154_s | Bacteria;Firmicutes;Clostridia;Clostridiales;Ruminococcaceae;PAC000661_g;PAC001154_s | 24 | 0.0605 |
| Frisingicoccus PAC001399_s | Bacteria;Firmicutes;Clostridia;Clostridiales;Lachnospiraceae;Frisingicoccus;PAC001399_s | 24 | 0.0605 |
| PAC001092_g PAC001774_s | Bacteria;Firmicutes;Clostridia;Clostridiales;Lachnospiraceae;PAC001092_g;PAC001774_s | 23 | 0.058 |
| Anaerotruncus PAC001590_s | Bacteria;Firmicutes;Clostridia;Clostridiales;Ruminococcaceae;Anaerotruncus;PAC001590_s | 22 | 0.0555 |
| KE159571_g PAC002495_s | Bacteria;Firmicutes;Clostridia;Clostridiales;Lachnospiraceae;KE159571_g;PAC002495_s | 22 | 0.0555 |
| KE159797_g PAC001222_s | Bacteria;Firmicutes;Clostridia;Clostridiales;Lachnospiraceae;KE159797_g;PAC001222_s | 21 | 0.053 |
| PAC000748_g PAC001412_s | Bacteria;Firmicutes;Clostridia;Clostridiales;Ruminococcaceae;PAC000748_g;PAC001412_s | 21 | 0.053 |
| Gemella palaticanis | Bacteria;Firmicutes;Bacilli;Bacillales;Gemella_f;Gemella;Gemella palaticanis | 20 | 0.0504 |
| Desulfovibrio PAC001079_s | Bacteria;Proteobacteria;Deltaproteobacteria;Desulfovibrionales;Desulfovibrionaceae;Desulfovibrio;PAC001079_s | 20 | 0.0504 |
| PAC002390_g PAC002390_s | Bacteria;Firmicutes;Clostridia;Clostridiales;Dehalobacterium_f;PAC002390_g;PAC002390_s | 20 | 0.0504 |
| PAC001225_g PAC001225_s | Bacteria;Firmicutes;Clostridia;Clostridiales;Lachnospiraceae;PAC001225_g;PAC001225_s | 19 | 0.0479 |
| PAC001144_g PAC001593_s | Bacteria;Firmicutes;Clostridia;Clostridiales;Ruminococcaceae;PAC001144_g;PAC001593_s | 19 | 0.0479 |
| Pseudoflavonifractor FJ879979_s | Bacteria;Firmicutes;Clostridia;Clostridiales;Ruminococcaceae;Pseudoflavonifractor;FJ879979_s | 18 | 0.0454 |
| KE159538_g PAC001708_s | Bacteria;Firmicutes;Clostridia;Clostridiales;Lachnospiraceae;KE159538_g;PAC001708_s | 18 | 0.0454 |
| Pseudoflavonifractor PAC001786_s | Bacteria;Firmicutes;Clostridia;Clostridiales;Ruminococcaceae;Pseudoflavonifractor;PAC001786_s | 18 | 0.0454 |
| Pseudoflavonifractor EF602939_s | Bacteria;Firmicutes;Clostridia;Clostridiales;Ruminococcaceae;Pseudoflavonifractor;EF602939_s | 17 | 0.0429 |
| PAC000692_g PAC001745_s | Bacteria;Firmicutes;Clostridia;Clostridiales;Lachnospiraceae;PAC000692_g;PAC001745_s | 17 | 0.0429 |
| PAC001270_g PAC001899_s group | Bacteria;Firmicutes;Clostridia;Clostridiales;Lachnospiraceae;PAC001270_g;PAC001899_s group | 17 | 0.0429 |
| Clostridium_g21_uc | Bacteria;Firmicutes;Clostridia;Clostridiales;Lachnospiraceae;Clostridium_g21;Clostridium_g21_uc | 17 | 0.0429 |
| Bacteroides oleiciplenus | Bacteria;Bacteroidetes;Bacteroidia;Bacteroidales;Bacteroidaceae;Bacteroides;Bacteroides oleiciplenus | 16 | 0.0404 |
| KE159797_g KE159797_s | Bacteria;Firmicutes;Clostridia;Clostridiales;Lachnospiraceae;KE159797_g;KE159797_s | 16 | 0.0404 |
| PAC001043_g PAC001752_s | Bacteria;Firmicutes;Clostridia;Clostridiales;Lachnospiraceae;PAC001043_g;PAC001752_s | 16 | 0.0404 |
| Paludicola PAC001784_s | Bacteria;Firmicutes;Clostridia;Clostridiales;Ruminococcaceae;Paludicola;PAC001784_s | 16 | 0.0404 |
| Acutalibacter muris | Bacteria;Firmicutes;Clostridia;Clostridiales;Ruminococcaceae;Acutalibacter;Acutalibacter muris | 15 | 0.0378 |
| PAC001092_g PAC001499_s | Bacteria;Firmicutes;Clostridia;Clostridiales;Lachnospiraceae;PAC001092_g;PAC001499_s | 15 | 0.0378 |
| PAC002482_g_uc | Bacteria;Bacteroidetes;Bacteroidia;Bacteroidales;AC160630_f;PAC002482_g;PAC002482_g_uc | 15 | 0.0378 |
| Ruminococcaceae_uc | Bacteria;Firmicutes;Clostridia;Clostridiales;Ruminococcaceae;Ruminococcaceae_uc | 14 | 0.0353 |
| Oscillibacter FJ881219_s | Bacteria;Firmicutes;Clostridia;Clostridiales;Ruminococcaceae;Oscillibacter;FJ881219_s | 14 | 0.0353 |
| Oscillibacter KE159714_s group | Bacteria;Firmicutes;Clostridia;Clostridiales;Ruminococcaceae;Oscillibacter;KE159714_s group | 14 | 0.0353 |
| Frisingicoccus PAC000700_s | Bacteria;Firmicutes;Clostridia;Clostridiales;Lachnospiraceae;Frisingicoccus;PAC000700_s | 14 | 0.0353 |
| PAC000671_g PAC001473_s | Bacteria;Firmicutes;Clostridia;Clostridiales;Lachnospiraceae;PAC000671_g;PAC001473_s | 14 | 0.0353 |
| Coprococcus_g2 PAC001722_s | Bacteria;Firmicutes;Clostridia;Clostridiales;Lachnospiraceae;Coprococcus_g2;PAC001722_s | 14 | 0.0353 |
| PAC001360_g_uc | Bacteria;Firmicutes;Clostridia;Clostridiales;Christensenellaceae;PAC001360_g;PAC001360_g_uc | 14 | 0.0353 |
| Lactobacillus gasseri group | Bacteria;Firmicutes;Bacilli;Lactobacillales;Lactobacillaceae;Lactobacillus;Lactobacillus gasseri group | 13 | 0.0328 |
| Lactobacillus reuteri group | Bacteria;Firmicutes;Bacilli;Lactobacillales;Lactobacillaceae;Lactobacillus;Lactobacillus reuteri group | 12 | 0.0303 |
| PAC001573_g EU509323_s | Bacteria;Firmicutes;Clostridia;Clostridiales;Mogibacterium_f;PAC001573_g;EU509323_s | 12 | 0.0303 |
| Pseudoflavonifractor PAC001131_s | Bacteria;Firmicutes;Clostridia;Clostridiales;Ruminococcaceae;Pseudoflavonifractor;PAC001131_s | 12 | 0.0303 |
| PAC001612_g PAC001612_s | Bacteria;Firmicutes;Clostridia;Clostridiales;Mogibacterium_f;PAC001612_g;PAC001612_s | 12 | 0.0303 |
| Sporobacter PAC001659_s | Bacteria;Firmicutes;Clostridia;Clostridiales;Ruminococcaceae;Sporobacter;PAC001659_s | 12 | 0.0303 |
| Pseudoflavonifractor PAC001667_s | Bacteria;Firmicutes;Clostridia;Clostridiales;Ruminococcaceae;Pseudoflavonifractor;PAC001667_s | 12 | 0.0303 |
| PAC001092_g_uc | Bacteria;Firmicutes;Clostridia;Clostridiales;Lachnospiraceae;PAC001092_g;PAC001092_g_uc | 12 | 0.0303 |
| Christensenella EU508399_s | Bacteria;Firmicutes;Clostridia;Clostridiales;Christensenellaceae;Christensenella;EU508399_s | 11 | 0.0277 |
| PAC001103_g PAC001103_s | Bacteria;Firmicutes;Clostridia;Clostridiales;Lachnospiraceae;PAC001103_g;PAC001103_s | 11 | 0.0277 |
| PAC000661_g PAC001251_s | Bacteria;Firmicutes;Clostridia;Clostridiales;Ruminococcaceae;PAC000661_g;PAC001251_s | 11 | 0.0277 |
| PAC001402_g PAC001546_s | Bacteria;Firmicutes;Clostridia;Clostridiales;Ruminococcaceae;PAC001402_g;PAC001546_s | 11 | 0.0277 |
| KE159571_g PAC001555_s | Bacteria;Firmicutes;Clostridia;Clostridiales;Lachnospiraceae;KE159571_g;PAC001555_s | 11 | 0.0277 |
| Clostridium_g21 PAC001772_s | Bacteria;Firmicutes;Clostridia;Clostridiales;Lachnospiraceae;Clostridium_g21;PAC001772_s | 11 | 0.0277 |
| Eubacterium_g6 PAC002402_s | Bacteria;Firmicutes;Clostridia;Clostridiales;Lachnospiraceae;Eubacterium_g6;PAC002402_s | 11 | 0.0277 |
| PAC001270_g PAC002420_s | Bacteria;Firmicutes;Clostridia;Clostridiales;Lachnospiraceae;PAC001270_g;PAC002420_s | 11 | 0.0277 |
| Enterorhabdus caecimuris | Bacteria;Actinobacteria;Coriobacteriia;Coriobacteriales;Coriobacteriaceae;Enterorhabdus;Enterorhabdus caecimuris | 10 | 0.0252 |
| PAC001372_g PAC001372_s | Bacteria;Firmicutes;Clostridia;Clostridiales;Lachnospiraceae;PAC001372_g;PAC001372_s | 10 | 0.0252 |
| Pseudoflavonifractor PAC001548_s | Bacteria;Firmicutes;Clostridia;Clostridiales;Ruminococcaceae;Pseudoflavonifractor;PAC001548_s | 10 | 0.0252 |
| KE159571_g PAC002035_s | Bacteria;Firmicutes;Clostridia;Clostridiales;Lachnospiraceae;KE159571_g;PAC002035_s | 10 | 0.0252 |
| Bilophila_uc | Bacteria;Proteobacteria;Deltaproteobacteria;Desulfovibrionales;Desulfovibrionaceae;Bilophila;Bilophila_uc | 10 | 0.0252 |
| Oscillibacter_uc | Bacteria;Firmicutes;Clostridia;Clostridiales;Ruminococcaceae;Oscillibacter;Oscillibacter_uc | 10 | 0.0252 |
| PAC001516_g_uc | Bacteria;Firmicutes;Clostridia;Clostridiales;Lachnospiraceae;PAC001516_g;PAC001516_g_uc | 10 | 0.0252 |
| Streptococcus acidominimus group | Bacteria;Firmicutes;Bacilli;Lactobacillales;Streptococcaceae;Streptococcus;Streptococcus acidominimus group | 9 | 0.0227 |
| KE159571_g KE159571_s | Bacteria;Firmicutes;Clostridia;Clostridiales;Lachnospiraceae;KE159571_g;KE159571_s | 9 | 0.0227 |
| Pseudoflavonifractor PAC001088_s | Bacteria;Firmicutes;Clostridia;Clostridiales;Ruminococcaceae;Pseudoflavonifractor;PAC001088_s | 9 | 0.0227 |
| PAC001165_g PAC001165_s | Bacteria;Firmicutes;Clostridia;Clostridiales;Lachnospiraceae;PAC001165_g;PAC001165_s | 9 | 0.0227 |
| PAC001201_g PAC001201_s group | Bacteria;Firmicutes;Clostridia;Clostridiales;Lachnospiraceae;PAC001201_g;PAC001201_s group | 9 | 0.0227 |
| KE159600_g PAC001232_s | Bacteria;Firmicutes;Clostridia;Clostridiales;Lachnospiraceae;KE159600_g;PAC001232_s | 9 | 0.0227 |
| Clostridium_g24 PAC001374_s | Bacteria;Firmicutes;Clostridia;Clostridiales;Lachnospiraceae;Clostridium_g24;PAC001374_s | 9 | 0.0227 |
| PAC001149_g PAC001511_s | Bacteria;Firmicutes;Clostridia;Clostridiales;Lachnospiraceae;PAC001149_g;PAC001511_s | 9 | 0.0227 |
| PAC001651_g PAC001651_s | Bacteria;Firmicutes;Clostridia;Clostridiales;Ruminococcaceae;PAC001651_g;PAC001651_s | 9 | 0.0227 |
| Frisingicoccus PAC001728_s group | Bacteria;Firmicutes;Clostridia;Clostridiales;Lachnospiraceae;Frisingicoccus;PAC001728_s group | 9 | 0.0227 |
| PAC000661_g PAC001777_s group | Bacteria;Firmicutes;Clostridia;Clostridiales;Ruminococcaceae;PAC000661_g;PAC001777_s group | 9 | 0.0227 |
| KE159571_g_uc | Bacteria;Firmicutes;Clostridia;Clostridiales;Lachnospiraceae;KE159571_g;KE159571_g_uc | 9 | 0.0227 |
| PAC000664_g_uc | Bacteria;Firmicutes;Clostridia;Clostridiales;Lachnospiraceae;PAC000664_g;PAC000664_g_uc | 9 | 0.0227 |
| PAC001068_g_uc | Bacteria;Bacteroidetes;Bacteroidia;Bacteroidales;Muribaculaceae;PAC001068_g;PAC001068_g_uc | 9 | 0.0227 |
| PAC000661_g EU842956_s | Bacteria;Firmicutes;Clostridia;Clostridiales;Ruminococcaceae;PAC000661_g;EU842956_s | 8 | 0.0202 |
| PAC000661_g PAC001182_s | Bacteria;Firmicutes;Clostridia;Clostridiales;Ruminococcaceae;PAC000661_g;PAC001182_s | 8 | 0.0202 |
| PAC001103_g PAC001724_s group | Bacteria;Firmicutes;Clostridia;Clostridiales;Lachnospiraceae;PAC001103_g;PAC001724_s group | 8 | 0.0202 |
| PAC002460_g PAC002460_s | Bacteria;Firmicutes;Clostridia;Clostridiales;Lachnospiraceae;PAC002460_g;PAC002460_s | 8 | 0.0202 |
| PAC002471_g PAC002471_s | Bacteria;Firmicutes;Clostridia;Clostridiales;Lachnospiraceae;PAC002471_g;PAC002471_s | 8 | 0.0202 |
| Parasutterella PAC002476_s | Bacteria;Proteobacteria;Betaproteobacteria;Burkholderiales;Sutterellaceae;Parasutterella;PAC002476_s | 8 | 0.0202 |
| PAC001228_g_uc | Bacteria;Firmicutes;Clostridia;Clostridiales;Lachnospiraceae;PAC001228_g;PAC001228_g_uc | 8 | 0.0202 |
| PAC000661_g AB185622_s | Bacteria;Firmicutes;Clostridia;Clostridiales;Ruminococcaceae;PAC000661_g;AB185622_s | 7 | 0.0177 |
| Pseudoflavonifractor HQ716472_s | Bacteria;Firmicutes;Clostridia;Clostridiales;Ruminococcaceae;Pseudoflavonifractor;HQ716472_s | 7 | 0.0177 |
| Pseudoflavonifractor LN866274_s | Bacteria;Firmicutes;Clostridia;Clostridiales;Ruminococcaceae;Pseudoflavonifractor;LN866274_s | 7 | 0.0177 |
| Anaerotignum PAC000685_s | Bacteria;Firmicutes;Clostridia;Clostridiales;Lachnospiraceae;Anaerotignum;PAC000685_s | 7 | 0.0177 |
| PAC001362_g PAC001362_s | Bacteria;Firmicutes;Erysipelotrichi;Erysipelotrichales;Erysipelotrichaceae;PAC001362_g;PAC001362_s | 7 | 0.0177 |
| PAC001528_g PAC001528_s | Bacteria;Firmicutes;Erysipelotrichi;Erysipelotrichales;Erysipelotrichaceae;PAC001528_g;PAC001528_s | 7 | 0.0177 |
| PAC001200_g PAC001554_s | Bacteria;Firmicutes;Clostridia;Clostridiales;Lachnospiraceae;PAC001200_g;PAC001554_s | 7 | 0.0177 |
| PAC001092_g PAC001710_s | Bacteria;Firmicutes;Clostridia;Clostridiales;Lachnospiraceae;PAC001092_g;PAC001710_s | 7 | 0.0177 |
| PAC001092_g PAC001742_s | Bacteria;Firmicutes;Clostridia;Clostridiales;Lachnospiraceae;PAC001092_g;PAC001742_s | 7 | 0.0177 |
| Paludicola PAC002097_s | Bacteria;Firmicutes;Clostridia;Clostridiales;Ruminococcaceae;Paludicola;PAC002097_s | 7 | 0.0177 |
| Acetatifactor PAC002332_s | Bacteria;Firmicutes;Clostridia;Clostridiales;Lachnospiraceae;Acetatifactor;PAC002332_s | 7 | 0.0177 |
| Pseudoflavonifractor PAC002490_s | Bacteria;Firmicutes;Clostridia;Clostridiales;Ruminococcaceae;Pseudoflavonifractor;PAC002490_s | 7 | 0.0177 |
| Bacteroidaceae_uc | Bacteria;Bacteroidetes;Bacteroidia;Bacteroidales;Bacteroidaceae;Bacteroidaceae_uc | 6 | 0.0151 |
| PAC000664_g AB702805_s | Bacteria;Firmicutes;Clostridia;Clostridiales;Lachnospiraceae;PAC000664_g;AB702805_s | 6 | 0.0151 |
| PAC000661_g EF434365_s | Bacteria;Firmicutes;Clostridia;Clostridiales;Ruminococcaceae;PAC000661_g;EF434365_s | 6 | 0.0151 |
| GQ451199_g EU472543_s | Bacteria;Firmicutes;Clostridia;Clostridiales;Ruminococcaceae;GQ451199_g;EU472543_s | 6 | 0.0151 |
| Eubacterium_g23 EU509480_s | Bacteria;Firmicutes;Clostridia;Clostridiales;Ruminococcaceae;Eubacterium_g23;EU509480_s | 6 | 0.0151 |
| Oscillibacter EU771312_s | Bacteria;Firmicutes;Clostridia;Clostridiales;Ruminococcaceae;Oscillibacter;EU771312_s | 6 | 0.0151 |
| KE159605_g KB822463_s | Bacteria;Firmicutes;Clostridia;Clostridiales;Lachnospiraceae;KE159605_g;KB822463_s | 6 | 0.0151 |
| PAC000186_g PAC001065_s | Bacteria;Bacteroidetes;Bacteroidia;Bacteroidales;Muribaculaceae;PAC000186_g;PAC001065_s | 6 | 0.0151 |
| Harryflintia PAC001713_s | Bacteria;Firmicutes;Clostridia;Clostridiales;Ruminococcaceae;Harryflintia;PAC001713_s | 6 | 0.0151 |
| PAC001573_g PAC001808_s | Bacteria;Firmicutes;Clostridia;Clostridiales;Mogibacterium_f;PAC001573_g;PAC001808_s | 6 | 0.0151 |
| LARJ_g_uc | Bacteria;Proteobacteria;Alphaproteobacteria;Rhodospirillales;Rhodospirillaceae;LARJ_g;LARJ_g_uc | 6 | 0.0151 |
| Pseudoflavonifractor ADDX_s | Bacteria;Firmicutes;Clostridia;Clostridiales;Ruminococcaceae;Pseudoflavonifractor;ADDX_s | 5 | 0.0126 |
| Pseudoflavonifractor EU460161_s | Bacteria;Firmicutes;Clostridia;Clostridiales;Ruminococcaceae;Pseudoflavonifractor;EU460161_s | 5 | 0.0126 |
| PAC000186_g PAC001064_s | Bacteria;Bacteroidetes;Bacteroidia;Bacteroidales;Muribaculaceae;PAC000186_g;PAC001064_s | 5 | 0.0126 |
| Christensenella PAC001140_s | Bacteria;Firmicutes;Clostridia;Clostridiales;Christensenellaceae;Christensenella;PAC001140_s | 5 | 0.0126 |
| PAC001301_g PAC001301_s | Bacteria;Firmicutes;Clostridia;Clostridiales;Christensenellaceae;PAC001301_g;PAC001301_s | 5 | 0.0126 |
| PAC001165_g PAC001366_s | Bacteria;Firmicutes;Clostridia;Clostridiales;Lachnospiraceae;PAC001165_g;PAC001366_s | 5 | 0.0126 |
| Pseudoflavonifractor PAC001547_s | Bacteria;Firmicutes;Clostridia;Clostridiales;Ruminococcaceae;Pseudoflavonifractor;PAC001547_s | 5 | 0.0126 |
| Oscillibacter PAC001551_s | Bacteria;Firmicutes;Clostridia;Clostridiales;Ruminococcaceae;Oscillibacter;PAC001551_s | 5 | 0.0126 |
| Acutalibacter PAC001594_s | Bacteria;Firmicutes;Clostridia;Clostridiales;Ruminococcaceae;Acutalibacter;PAC001594_s | 5 | 0.0126 |
| Clostridium_g21 PAC001757_s group | Bacteria;Firmicutes;Clostridia;Clostridiales;Lachnospiraceae;Clostridium_g21;PAC001757_s group | 5 | 0.0126 |
| KE159600_g_uc | Bacteria;Firmicutes;Clostridia;Clostridiales;Lachnospiraceae;KE159600_g;KE159600_g_uc | 5 | 0.0126 |
| PAC000197_g_uc | Bacteria;Tenericutes;Mollicutes;PAC001057_o;PAC000197_f;PAC000197_g;PAC000197_g_uc | 5 | 0.0126 |
| PAC001165_g_uc | Bacteria;Firmicutes;Clostridia;Clostridiales;Lachnospiraceae;PAC001165_g;PAC001165_g_uc | 5 | 0.0126 |
| Bacillus subtilis group | Bacteria;Firmicutes;Bacilli;Bacillales;Bacillaceae;Bacillus;Bacillus subtilis group | 4 | 0.0101 |
| Clostridium tertium group | Bacteria;Firmicutes;Clostridia;Clostridiales;Clostridiaceae;Clostridium;Clostridium tertium group | 4 | 0.0101 |
| Eubacterium plexicaudatum | Bacteria;Firmicutes;Clostridia;Clostridiales;Lachnospiraceae;Eubacterium_g6;Eubacterium plexicaudatum | 4 | 0.0101 |
| KE159600_g EF098562_s | Bacteria;Firmicutes;Clostridia;Clostridiales;Lachnospiraceae;KE159600_g;EF098562_s | 4 | 0.0101 |
| Clostridium_g24 EU504811_s | Bacteria;Firmicutes;Clostridia;Clostridiales;Lachnospiraceae;Clostridium_g24;EU504811_s | 4 | 0.0101 |
| Bacteroides JN559675_s | Bacteria;Bacteroidetes;Bacteroidia;Bacteroidales;Bacteroidaceae;Bacteroides;JN559675_s | 4 | 0.0101 |
| Acetatifactor PAC001373_s | Bacteria;Firmicutes;Clostridia;Clostridiales;Lachnospiraceae;Acetatifactor;PAC001373_s | 4 | 0.0101 |
| PAC001236_g PAC001767_s | Bacteria;Firmicutes;Clostridia;Clostridiales;Mogibacterium_f;PAC001236_g;PAC001767_s | 4 | 0.0101 |
| PAC001908_g PAC001908_s | Bacteria;Firmicutes;Clostridia;Clostridiales;Ruminococcaceae;PAC001908_g;PAC001908_s | 4 | 0.0101 |
| Pseudoflavonifractor PAC002119_s group | Bacteria;Firmicutes;Clostridia;Clostridiales;Ruminococcaceae;Pseudoflavonifractor;PAC002119_s group | 4 | 0.0101 |
| Christensenella PAC002165_s | Bacteria;Firmicutes;Clostridia;Clostridiales;Christensenellaceae;Christensenella;PAC002165_s | 4 | 0.0101 |
| Pseudoflavonifractor PAC002302_s | Bacteria;Firmicutes;Clostridia;Clostridiales;Ruminococcaceae;Pseudoflavonifractor;PAC002302_s | 4 | 0.0101 |
| PAC002454_g PAC002454_s | Bacteria;Firmicutes;Clostridia;Clostridiales;Lachnospiraceae;PAC002454_g;PAC002454_s | 4 | 0.0101 |
| Pseudoflavonifractor PAC002475_s | Bacteria;Firmicutes;Clostridia;Clostridiales;Ruminococcaceae;Pseudoflavonifractor;PAC002475_s | 4 | 0.0101 |
| Alistipes obesi | Bacteria;Bacteroidetes;Bacteroidia;Bacteroidales;Rikenellaceae;Alistipes;Alistipes obesi | 3 | 0.0076 |
| Bacteroides rodentium | Bacteria;Bacteroidetes;Bacteroidia;Bacteroidales;Bacteroidaceae;Bacteroides;Bacteroides rodentium | 3 | 0.0076 |
| Clostridium methylpentosum | Bacteria;Firmicutes;Clostridia;Clostridiales;Ruminococcaceae;Massilioclostridium;Clostridium methylpentosum | 3 | 0.0076 |
| Neisseria perflava | Bacteria;Proteobacteria;Betaproteobacteria;Neisseriales;Neisseriaceae;Neisseria;Neisseria perflava | 3 | 0.0076 |
| KE159538_g EF097676_s | Bacteria;Firmicutes;Clostridia;Clostridiales;Lachnospiraceae;KE159538_g;EF097676_s | 3 | 0.0076 |
| PAC001225_g EU504346_s | Bacteria;Firmicutes;Clostridia;Clostridiales;Lachnospiraceae;PAC001225_g;EU504346_s | 3 | 0.0076 |
| Acetatifactor FJ879577_s | Bacteria;Firmicutes;Clostridia;Clostridiales;Lachnospiraceae;Acetatifactor;FJ879577_s | 3 | 0.0076 |
| PAC001360_g GQ157859_s | Bacteria;Firmicutes;Clostridia;Clostridiales;Christensenellaceae;PAC001360_g;GQ157859_s | 3 | 0.0076 |
| PAC001219_g HM124151_s | Bacteria;Firmicutes;Clostridia;Clostridiales;Christensenellaceae;PAC001219_g;HM124151_s | 3 | 0.0076 |
| Emergencia PAC001235_s | Bacteria;Firmicutes;Clostridia;Clostridiales;Mogibacterium_f;Emergencia;PAC001235_s | 3 | 0.0076 |
| PAC001323_g PAC001323_s | Bacteria;Tenericutes;Mollicutes;PAC001057_o;PAC000197_f;PAC001323_g;PAC001323_s | 3 | 0.0076 |
| PAC000692_g PAC001387_s | Bacteria;Firmicutes;Clostridia;Clostridiales;Lachnospiraceae;PAC000692_g;PAC001387_s | 3 | 0.0076 |
| KE159605_g PAC001396_s | Bacteria;Firmicutes;Clostridia;Clostridiales;Lachnospiraceae;KE159605_g;PAC001396_s | 3 | 0.0076 |
| Pseudoflavonifractor PAC001542_s | Bacteria;Firmicutes;Clostridia;Clostridiales;Ruminococcaceae;Pseudoflavonifractor;PAC001542_s | 3 | 0.0076 |
| PAC001588_g PAC001588_s | Bacteria;Firmicutes;Clostridia;Clostridiales;Lachnospiraceae;PAC001588_g;PAC001588_s | 3 | 0.0076 |
| Oscillibacter PAC001706_s | Bacteria;Firmicutes;Clostridia;Clostridiales;Ruminococcaceae;Oscillibacter;PAC001706_s | 3 | 0.0076 |
| PAC001112_g PAC002441_s | Bacteria;Bacteroidetes;Bacteroidia;Bacteroidales;Muribaculaceae;PAC001112_g;PAC002441_s | 3 | 0.0076 |
| PAC001219_g_uc | Bacteria;Firmicutes;Clostridia;Clostridiales;Christensenellaceae;PAC001219_g;PAC001219_g_uc | 3 | 0.0076 |
| PAC001778_g_uc | Bacteria;Firmicutes;Clostridia;Clostridiales;Ruminococcaceae;PAC001778_g;PAC001778_g_uc | 3 | 0.0076 |
| Brucellaceae group | Bacteria;Proteobacteria;Alphaproteobacteria;Rhizobiales;Brucellaceae;Brucellaceae_g;Brucellaceae group | 2 | 0.005 |
| Paenibacillus lautus | Bacteria;Firmicutes;Bacilli;Bacillales;Paenibacillaceae;Paenibacillus;Paenibacillus lautus | 2 | 0.005 |
| PAC000664_g AB626958_s | Bacteria;Firmicutes;Clostridia;Clostridiales;Lachnospiraceae;PAC000664_g;AB626958_s | 2 | 0.005 |
| Bacteroides DQ799557_s | Bacteria;Bacteroidetes;Bacteroidia;Bacteroidales;Bacteroidaceae;Bacteroides;DQ799557_s | 2 | 0.005 |
| Bacteroides DQ805799_s | Bacteria;Bacteroidetes;Bacteroidia;Bacteroidales;Bacteroidaceae;Bacteroides;DQ805799_s | 2 | 0.005 |
| PAC000196_g EU466069_s | Bacteria;Firmicutes;Clostridia;Clostridiales;Lachnospiraceae;PAC000196_g;EU466069_s | 2 | 0.005 |
| PAC000692_g EU505164_s | Bacteria;Firmicutes;Clostridia;Clostridiales;Lachnospiraceae;PAC000692_g;EU505164_s | 2 | 0.005 |
| Pseudoflavonifractor FJ879507_s | Bacteria;Firmicutes;Clostridia;Clostridiales;Ruminococcaceae;Pseudoflavonifractor;FJ879507_s | 2 | 0.005 |
| Pseudoflavonifractor FJ879818_s | Bacteria;Firmicutes;Clostridia;Clostridiales;Ruminococcaceae;Pseudoflavonifractor;FJ879818_s | 2 | 0.005 |
| KE159571_g KE159641_s | Bacteria;Firmicutes;Clostridia;Clostridiales;Lachnospiraceae;KE159571_g;KE159641_s | 2 | 0.005 |
| Frisingicoccus PAC000688_s | Bacteria;Firmicutes;Clostridia;Clostridiales;Lachnospiraceae;Frisingicoccus;PAC000688_s | 2 | 0.005 |
| Eubacterium_g4 PAC001174_s | Bacteria;Firmicutes;Clostridia;Clostridiales;Lachnospiraceae;Eubacterium_g4;PAC001174_s | 2 | 0.005 |
| PAC001108_g PAC001193_s | Bacteria;Tenericutes;Mollicutes;PAC001057_o;PAC001057_f;PAC001108_g;PAC001193_s | 2 | 0.005 |
| PAC001219_g PAC001280_s | Bacteria;Firmicutes;Clostridia;Clostridiales;Christensenellaceae;PAC001219_g;PAC001280_s | 2 | 0.005 |
| PAC001092_g PAC001381_s | Bacteria;Firmicutes;Clostridia;Clostridiales;Lachnospiraceae;PAC001092_g;PAC001381_s | 2 | 0.005 |
| PAC001092_g PAC001388_s | Bacteria;Firmicutes;Clostridia;Clostridiales;Lachnospiraceae;PAC001092_g;PAC001388_s | 2 | 0.005 |
| Anaerotignum PAC001464_s | Bacteria;Firmicutes;Clostridia;Clostridiales;Lachnospiraceae;Anaerotignum;PAC001464_s | 2 | 0.005 |
| Agathobaculum PAC001476_s | Bacteria;Firmicutes;Clostridia;Clostridiales;Ruminococcaceae;Agathobaculum;PAC001476_s | 2 | 0.005 |
| PAC001092_g PAC001517_s | Bacteria;Firmicutes;Clostridia;Clostridiales;Lachnospiraceae;PAC001092_g;PAC001517_s | 2 | 0.005 |
| PAC000692_g PAC001577_s | Bacteria;Firmicutes;Clostridia;Clostridiales;Lachnospiraceae;PAC000692_g;PAC001577_s | 2 | 0.005 |
| Pseudoflavonifractor PAC001608_s | Bacteria;Firmicutes;Clostridia;Clostridiales;Ruminococcaceae;Pseudoflavonifractor;PAC001608_s | 2 | 0.005 |
| PAC001573_g PAC002010_s | Bacteria;Firmicutes;Clostridia;Clostridiales;Mogibacterium_f;PAC001573_g;PAC002010_s | 2 | 0.005 |
| PAC000661_g PAC002422_s | Bacteria;Firmicutes;Clostridia;Clostridiales;Ruminococcaceae;PAC000661_g;PAC002422_s | 2 | 0.005 |
| PAC000664_g PAC002486_s | Bacteria;Firmicutes;Clostridia;Clostridiales;Lachnospiraceae;PAC000664_g;PAC002486_s | 2 | 0.005 |
| Parabacteroides_uc | Bacteria;Bacteroidetes;Bacteroidia;Bacteroidales;Porphyromonadaceae;Parabacteroides;Parabacteroides_uc | 2 | 0.005 |
| PAC001362_g_uc | Bacteria;Firmicutes;Erysipelotrichi;Erysipelotrichales;Erysipelotrichaceae;PAC001362_g;PAC001362_g_uc | 2 | 0.005 |
| Bacteroides dorei | Bacteria;Bacteroidetes;Bacteroidia;Bacteroidales;Bacteroidaceae;Bacteroides;Bacteroides dorei | 1 | 0.0025 |
| Bacteroides xylanisolvens group | Bacteria;Bacteroidetes;Bacteroidia;Bacteroidales;Bacteroidaceae;Bacteroides;Bacteroides xylanisolvens group | 1 | 0.0025 |
| Clostridium symbiosum | Bacteria;Firmicutes;Clostridia;Clostridiales;Lachnospiraceae;Clostridium_g35;Clostridium symbiosum | 1 | 0.0025 |
| Enterorhabdus muris | Bacteria;Actinobacteria;Coriobacteriia;Coriobacteriales;Coriobacteriaceae;Enterorhabdus;Enterorhabdus muris | 1 | 0.0025 |
| Faecalimonas umbilicata | Bacteria;Firmicutes;Clostridia;Clostridiales;Lachnospiraceae;Faecalimonas;Faecalimonas umbilicata | 1 | 0.0025 |
| Prevotella salivae | Bacteria;Bacteroidetes;Bacteroidia;Bacteroidales;Prevotellaceae;Prevotella;Prevotella salivae | 1 | 0.0025 |
| Acutalibacter AB622826_s | Bacteria;Firmicutes;Clostridia;Clostridiales;Ruminococcaceae;Acutalibacter;AB622826_s | 1 | 0.0025 |
| PAC000661_g AY916208_s | Bacteria;Firmicutes;Clostridia;Clostridiales;Ruminococcaceae;PAC000661_g;AY916208_s | 1 | 0.0025 |
| Muribaculum DQ815542_s | Bacteria;Bacteroidetes;Bacteroidia;Bacteroidales;Muribaculaceae;Muribaculum;DQ815542_s | 1 | 0.0025 |
| Acetatifactor EU508111_s | Bacteria;Firmicutes;Clostridia;Clostridiales;Lachnospiraceae;Acetatifactor;EU508111_s | 1 | 0.0025 |
| Pseudoflavonifractor EU542517_s | Bacteria;Firmicutes;Clostridia;Clostridiales;Ruminococcaceae;Pseudoflavonifractor;EU542517_s | 1 | 0.0025 |
| PAC000661_g FJ681631_s | Bacteria;Firmicutes;Clostridia;Clostridiales;Ruminococcaceae;PAC000661_g;FJ681631_s | 1 | 0.0025 |
| Pseudoflavonifractor FJ879530_s | Bacteria;Firmicutes;Clostridia;Clostridiales;Ruminococcaceae;Pseudoflavonifractor;FJ879530_s | 1 | 0.0025 |
| Oscillibacter FJ880499_s | Bacteria;Firmicutes;Clostridia;Clostridiales;Ruminococcaceae;Oscillibacter;FJ880499_s | 1 | 0.0025 |
| PAC002042_g FJ951869_s | Bacteria;Firmicutes;Clostridia;Clostridiales;Lachnospiraceae;PAC002042_g;FJ951869_s | 1 | 0.0025 |
| Acetatifactor HM123975_s | Bacteria;Firmicutes;Clostridia;Clostridiales;Lachnospiraceae;Acetatifactor;HM123975_s | 1 | 0.0025 |
| KE159810_g KE159810_s | Bacteria;Firmicutes;Clostridia;Clostridiales;Lachnospiraceae;KE159810_g;KE159810_s | 1 | 0.0025 |
| Paludicola PAC000694_s | Bacteria;Firmicutes;Clostridia;Clostridiales;Ruminococcaceae;Paludicola;PAC000694_s | 1 | 0.0025 |
| PAC000661_g PAC001329_s | Bacteria;Firmicutes;Clostridia;Clostridiales;Ruminococcaceae;PAC000661_g;PAC001329_s | 1 | 0.0025 |
| Oscillibacter PAC001368_s | Bacteria;Firmicutes;Clostridia;Clostridiales;Ruminococcaceae;Oscillibacter;PAC001368_s | 1 | 0.0025 |
| PAC001389_g PAC001389_s | Bacteria;Actinobacteria;Coriobacteriia;Coriobacteriales;Coriobacteriaceae;PAC001389_g;PAC001389_s | 1 | 0.0025 |
| PAC000197_g PAC001409_s | Bacteria;Tenericutes;Mollicutes;PAC001057_o;PAC000197_f;PAC000197_g;PAC001409_s | 1 | 0.0025 |
| PAC001103_g PAC001487_s | Bacteria;Firmicutes;Clostridia;Clostridiales;Lachnospiraceae;PAC001103_g;PAC001487_s | 1 | 0.0025 |
| PAC000748_g PAC001489_s | Bacteria;Firmicutes;Clostridia;Clostridiales;Ruminococcaceae;PAC000748_g;PAC001489_s | 1 | 0.0025 |
| Acutalibacter PAC001519_s | Bacteria;Firmicutes;Clostridia;Clostridiales;Ruminococcaceae;Acutalibacter;PAC001519_s | 1 | 0.0025 |
| Oscillibacter PAC001638_s | Bacteria;Firmicutes;Clostridia;Clostridiales;Ruminococcaceae;Oscillibacter;PAC001638_s | 1 | 0.0025 |
| PAC001609_g PAC001650_s | Bacteria;Firmicutes;Clostridia;Clostridiales;Mogibacterium_f;PAC001609_g;PAC001650_s | 1 | 0.0025 |
| PAC001588_g PAC001669_s | Bacteria;Firmicutes;Clostridia;Clostridiales;Lachnospiraceae;PAC001588_g;PAC001669_s | 1 | 0.0025 |
| Anaerotignum PAC001709_s | Bacteria;Firmicutes;Clostridia;Clostridiales;Lachnospiraceae;Anaerotignum;PAC001709_s | 1 | 0.0025 |
| PAC001092_g PAC001726_s | Bacteria;Firmicutes;Clostridia;Clostridiales;Lachnospiraceae;PAC001092_g;PAC001726_s | 1 | 0.0025 |
| PAC001092_g PAC001811_s | Bacteria;Firmicutes;Clostridia;Clostridiales;Lachnospiraceae;PAC001092_g;PAC001811_s | 1 | 0.0025 |
| PAC001778_g PAC001909_s | Bacteria;Firmicutes;Clostridia;Clostridiales;Ruminococcaceae;PAC001778_g;PAC001909_s | 1 | 0.0025 |
| KE159797_g PAC001980_s | Bacteria;Firmicutes;Clostridia;Clostridiales;Lachnospiraceae;KE159797_g;PAC001980_s | 1 | 0.0025 |
| PAC001092_g PAC002030_s | Bacteria;Firmicutes;Clostridia;Clostridiales;Lachnospiraceae;PAC001092_g;PAC002030_s | 1 | 0.0025 |
| PAC002178_g PAC002178_s | Bacteria;Firmicutes;Clostridia;Clostridiales;Lachnospiraceae;PAC002178_g;PAC002178_s | 1 | 0.0025 |
| PAC001144_g PAC002359_s | Bacteria;Firmicutes;Clostridia;Clostridiales;Ruminococcaceae;PAC001144_g;PAC002359_s | 1 | 0.0025 |
| KE159571_g PAC002468_s | Bacteria;Firmicutes;Clostridia;Clostridiales;Lachnospiraceae;KE159571_g;PAC002468_s | 1 | 0.0025 |
| Frisingicoccus PAC002470_s | Bacteria;Firmicutes;Clostridia;Clostridiales;Lachnospiraceae;Frisingicoccus;PAC002470_s | 1 | 0.0025 |
| Corynebacterium Y12792_s | Bacteria;Actinobacteria;Actinobacteria_c;Corynebacteriales;Corynebacteriaceae;Corynebacterium;Y12792_s | 1 | 0.0025 |
| Eubacterium_g23_uc | Bacteria;Firmicutes;Clostridia;Clostridiales;Ruminococcaceae;Eubacterium_g23;Eubacterium_g23_uc | 1 | 0.0025 |
| KE159538_g_uc | Bacteria;Firmicutes;Clostridia;Clostridiales;Lachnospiraceae;KE159538_g;KE159538_g_uc | 1 | 0.0025 |

**Set 1_0%_P: Day 28_heme-SCP 0%_Phylum**

| Taxon name | Taxonomy | Count | Proportion(%) |
|---|---|---|---|
| Firmicutes | Bacteria;Firmicutes | 15547 | 65.406 |
| Bacteroidetes | Bacteria;Bacteroidetes | 6568 | 27.6315 |
| Verrucomicrobia | Bacteria;Verrucomicrobia | 1008 | 4.2406 |
| Proteobacteria | Bacteria;Proteobacteria | 553 | 2.3265 |
| Actinobacteria | Bacteria;Actinobacteria | 40 | 0.1683 |
| Cyanobacteria | Bacteria;Cyanobacteria | 22 | 0.0926 |
| Tenericutes | Bacteria;Tenericutes | 17 | 0.0715 |
| Saccharibacteria_TM7 | Bacteria;Saccharibacteria_TM7 | 11 | 0.0463 |
| Deferribacteres | Bacteria;Deferribacteres | 3 | 0.0126 |
| Acidobacteria | Bacteria;Acidobacteria | 1 | 0.0042 |

**Set 1_0.05%_P: Day 28_heme-SCP 0.05%_Phylum**

| Taxon name | Taxonomy | Count | Proportion(%) |
|---|---|---|---|
| Firmicutes | Bacteria;Firmicutes | 14065 | 63.2817 |
| Bacteroidetes | Bacteria;Bacteroidetes | 3906 | 17.574 |
| Verrucomicrobia | Bacteria;Verrucomicrobia | 3369 | 15.1579 |
| Proteobacteria | Bacteria;Proteobacteria | 728 | 3.2754 |
| Tenericutes | Bacteria;Tenericutes | 68 | 0.3059 |
| Actinobacteria | Bacteria;Actinobacteria | 59 | 0.2655 |
| Deferribacteres | Bacteria;Deferribacteres | 30 | 0.135 |
| Chloroflexi | Bacteria;Chloroflexi | 1 | 0.0045 |

**Set 1_0.5%_P: Day 28_heme-SCP 0.5%_Phylum**

| Taxon name | Taxonomy | Count | Proportion(%) |
|---|---|---|---|
| Firmicutes | Bacteria;Firmicutes | 11514 | 53.4069 |
| Bacteroidetes | Bacteria;Bacteroidetes | 6532 | 30.2983 |
| Verrucomicrobia | Bacteria;Verrucomicrobia | 2442 | 11.3271 |
| Proteobacteria | Bacteria;Proteobacteria | 598 | 2.7738 |
| Actinobacteria | Bacteria;Actinobacteria | 349 | 1.6188 |
| Tenericutes | Bacteria;Tenericutes | 94 | 0.436 |
| Deferribacteres | Bacteria;Deferribacteres | 25 | 0.116 |
| Chloroflexi | Bacteria;Chloroflexi | 2 | 0.0093 |
| Saccharibacteria_TM7 | Bacteria;Saccharibacteria_TM7 | 2 | 0.0093 |
| Acidobacteria | Bacteria;Acidobacteria | 1 | 0.0046 |

**Set 1_0%_G: Day 28_heme-SCP 0%_Genus**

| Taxon name | Taxonomy | Count | Proportion(%) |
|---|---|---|---|
| Lactobacillus | Bacteria;Firmicutes;Bacilli;Lactobacillales;Lactobacillaceae;Lactobacillus | 11419 | 48.0395 |
| Bacteroides | Bacteria;Bacteroidetes;Bacteroidia;Bacteroidales;Bacteroidaceae;Bacteroides | 4946 | 20.8077 |
| Akkermansia | Bacteria;Verrucomicrobia;Verrucomicrobiae;Verrucomicrobiales;Akkermansiaceae;Akkermansia | 1008 | 4.2406 |
| Lactococcus | Bacteria;Firmicutes;Bacilli;Lactobacillales;Streptococcaceae;Lactococcus | 928 | 3.9041 |
| PAC000198_g | Bacteria;Bacteroidetes;Bacteroidia;Bacteroidales;Muribaculaceae;PAC000198_g | 515 | 2.1666 |
| Bilophila | Bacteria;Proteobacteria;Deltaproteobacteria;Desulfovibrionales;Desulfovibrionaceae;Bilophila | 438 | 1.8427 |
| KE159600_g | Bacteria;Firmicutes;Clostridia;Clostridiales;Lachnospiraceae;KE159600_g | 389 | 1.6365 |
| PAC000661_g | Bacteria;Firmicutes;Clostridia;Clostridiales;Ruminococcaceae;PAC000661_g | 341 | 1.4346 |
| Pseudoflavonifractor | Bacteria;Firmicutes;Clostridia;Clostridiales;Ruminococcaceae;Pseudoflavonifractor | 323 | 1.3589 |
| PAC001112_g | Bacteria;Bacteroidetes;Bacteroidia;Bacteroidales;Muribaculaceae;PAC001112_g | 257 | 1.0812 |
| Oscillibacter | Bacteria;Firmicutes;Clostridia;Clostridiales;Ruminococcaceae;Oscillibacter | 238 | 1.0013 |
| PAC001068_g | Bacteria;Bacteroidetes;Bacteroidia;Bacteroidales;Muribaculaceae;PAC001068_g | 230 | 0.9676 |
| PAC001452_g | Bacteria;Firmicutes;Clostridia;Clostridiales;Lachnospiraceae;PAC001452_g | 200 | 0.8414 |
| PAC001141_g | Bacteria;Firmicutes;Clostridia;Clostridiales;Christensenellaceae;PAC001141_g | 163 | 0.6857 |
| PAC001516_g | Bacteria;Firmicutes;Clostridia;Clostridiales;Lachnospiraceae;PAC001516_g | 136 | 0.5721 |
| PAC001074_g | Bacteria;Bacteroidetes;Bacteroidia;Bacteroidales;Muribaculaceae;PAC001074_g | 135 | 0.5679 |
| PAC001165_g | Bacteria;Firmicutes;Clostridia;Clostridiales;Lachnospiraceae;PAC001165_g | 109 | 0.4586 |
| Alistipes | Bacteria;Bacteroidetes;Bacteroidia;Bacteroidales;Rikenellaceae;Alistipes | 104 | 0.4375 |
| PAC001066_g | Bacteria;Bacteroidetes;Bacteroidia;Bacteroidales;Muribaculaceae;PAC001066_g | 98 | 0.4123 |
| PAC001118_g | Bacteria;Firmicutes;Clostridia;Clostridiales;Lachnospiraceae;PAC001118_g | 85 | 0.3576 |
| Streptococcus | Bacteria;Firmicutes;Bacilli;Lactobacillales;Streptococcaceae;Streptococcus | 76 | 0.3197 |
| Eubacterium_g23 | Bacteria;Firmicutes;Clostridia;Clostridiales;Ruminococcaceae;Eubacterium_g23 | 72 | 0.3029 |
| Clostridium_g21 | Bacteria;Firmicutes;Clostridia;Clostridiales;Lachnospiraceae;Clostridium_g21 | 70 | 0.2945 |
| KE159571_g | Bacteria;Firmicutes;Clostridia;Clostridiales;Lachnospiraceae;KE159571_g | 63 | 0.265 |
| PAC000186_g | Bacteria;Bacteroidetes;Bacteroidia;Bacteroidales;Muribaculaceae;PAC000186_g | 63 | 0.265 |
| Acetatifactor | Bacteria;Firmicutes;Clostridia;Clostridiales;Lachnospiraceae;Acetatifactor | 57 | 0.2398 |
| PAC000664_g | Bacteria;Firmicutes;Clostridia;Clostridiales;Lachnospiraceae;PAC000664_g | 56 | 0.2356 |
| Sporobacter | Bacteria;Firmicutes;Clostridia;Clostridiales;Ruminococcaceae;Sporobacter | 54 | 0.2272 |
| PAC001402_g | Bacteria;Firmicutes;Clostridia;Clostridiales;Ruminococcaceae;PAC001402_g | 50 | 0.2103 |
| PAC001485_g | Bacteria;Bacteroidetes;Bacteroidia;Bacteroidales;Muribaculaceae;PAC001485_g | 49 | 0.2061 |
| Parabacteroides | Bacteria;Bacteroidetes;Bacteroidia;Bacteroidales;Porphyromonadaceae;Parabacteroides | 45 | 0.1893 |
| Anaerotignum | Bacteria;Firmicutes;Clostridia;Clostridiales;Lachnospiraceae;Anaerotignum | 43 | 0.1809 |
| PAC000748_g | Bacteria;Firmicutes;Clostridia;Clostridiales;Ruminococcaceae;PAC000748_g | 43 | 0.1809 |
| Paludicola | Bacteria;Firmicutes;Clostridia;Clostridiales;Ruminococcaceae;Paludicola | 40 | 0.1683 |
| Anaerotruncus | Bacteria;Firmicutes;Clostridia;Clostridiales;Ruminococcaceae;Anaerotruncus | 38 | 0.1599 |
| Desulfovibrio | Bacteria;Proteobacteria;Deltaproteobacteria;Desulfovibrionales;Desulfovibrionaceae;Desulfovibrio | 38 | 0.1599 |
| Odoribacter | Bacteria;Bacteroidetes;Bacteroidia;Bacteroidales;Odoribacteraceae;Odoribacter | 36 | 0.1515 |
| LARJ_g | Bacteria;Proteobacteria;Alphaproteobacteria;Rhodospirillales;Rhodospirillaceae;LARJ_g | 36 | 0.1515 |
| PAC002482_g | Bacteria;Bacteroidetes;Bacteroidia;Bacteroidales;AC160630_f;PAC002482_g | 36 | 0.1515 |
| Enterorhabdus | Bacteria;Actinobacteria;Coriobacteriia;Coriobacteriales;Coriobacteriaceae;Enterorhabdus | 34 | 0.143 |
| KE159797_g | Bacteria;Firmicutes;Clostridia;Clostridiales;Lachnospiraceae;KE159797_g | 33 | 0.1388 |
| PAC001092_g | Bacteria;Firmicutes;Clostridia;Clostridiales;Lachnospiraceae;PAC001092_g | 29 | 0.122 |
| Emergencia | Bacteria;Firmicutes;Clostridia;Clostridiales;Mogibacterium_f;Emergencia | 28 | 0.1178 |
| PAC001390_g | Bacteria;Firmicutes;Clostridia;Clostridiales;Lachnospiraceae;PAC001390_g | 27 | 0.1136 |
| PAC000683_g | Bacteria;Firmicutes;Clostridia;Clostridiales;Ruminococcaceae;PAC000683_g | 23 | 0.0968 |
| Clostridium_g24 | Bacteria;Firmicutes;Clostridia;Clostridiales;Lachnospiraceae;Clostridium_g24 | 22 | 0.0926 |
| FR888536_g | Bacteria;Cyanobacteria;Vampirovibrio_c;FR888536_o;FR888536_f;FR888536_g | 22 | 0.0926 |
| PAC001385_g | Bacteria;Firmicutes;Clostridia;Clostridiales;Lachnospiraceae;PAC001385_g | 21 | 0.0883 |
| Eubacterium_g6 | Bacteria;Firmicutes;Clostridia;Clostridiales;Lachnospiraceae;Eubacterium_g6 | 19 | 0.0799 |
| PAC001063_g | Bacteria;Bacteroidetes;Bacteroidia;Bacteroidales;Muribaculaceae;PAC001063_g | 19 | 0.0799 |
| PAC001138_g | Bacteria;Firmicutes;Clostridia;Clostridiales;Lachnospiraceae;PAC001138_g | 18 | 0.0757 |
| PAC001103_g | Bacteria;Firmicutes;Clostridia;Clostridiales;Lachnospiraceae;PAC001103_g | 17 | 0.0715 |
| Frisingicoccus | Bacteria;Firmicutes;Clostridia;Clostridiales;Lachnospiraceae;Frisingicoccus | 16 | 0.0673 |
| Staphylococcus | Bacteria;Firmicutes;Bacilli;Bacillales;Staphylococcaceae;Staphylococcus | 16 | 0.0673 |
| PAC001362_g | Bacteria;Firmicutes;Erysipelotrichi;Erysipelotrichales;Erysipelotrichaceae;PAC001362_g | 14 | 0.0589 |
| PAC001400_g | Bacteria;Firmicutes;Clostridia;Clostridiales;Peptococcaceae;PAC001400_g | 14 | 0.0589 |
| PAC002314_g | Bacteria;Firmicutes;Erysipelotrichi;Erysipelotrichales;Erysipelotrichaceae;PAC002314_g | 14 | 0.0589 |
| Lachnospiraceae_uc | Bacteria;Firmicutes;Clostridia;Clostridiales;Lachnospiraceae;Lachnospiraceae_uc | 14 | 0.0589 |
| PAC001221_g | Bacteria;Firmicutes;Clostridia;Clostridiales;Dehalobacterium_f;PAC001221_g | 12 | 0.0505 |
| Escherichia | Bacteria;Proteobacteria;Gammaproteobacteria;Enterobacterales;Enterobacteriaceae;Escherichia | 11 | 0.0463 |
| Helicobacter | Bacteria;Proteobacteria;Epsilonproteobacteria;Campylobacterales;Helicobacteraceae;Helicobacter | 11 | 0.0463 |
| PAC000677_g | Bacteria;Saccharibacteria_TM7;Saccharimonas_c;Saccharimonas_o;Saccharimonas_f;PAC000677_g | 11 | 0.0463 |
| PAC000671_g | Bacteria;Firmicutes;Clostridia;Clostridiales;Lachnospiraceae;PAC000671_g | 10 | 0.0421 |
| PAC001201_g | Bacteria;Firmicutes;Clostridia;Clostridiales;Lachnospiraceae;PAC001201_g | 10 | 0.0421 |
| PAC001524_g | Bacteria;Firmicutes;Clostridia;Clostridiales;Lachnospiraceae;PAC001524_g | 9 | 0.0379 |
| PAC001738_g | Bacteria;Firmicutes;Clostridia;Clostridiales;Lachnospiraceae;PAC001738_g | 9 | 0.0379 |
| Enterococcus | Bacteria;Firmicutes;Bacilli;Lactobacillales;Enterococcaceae;Enterococcus | 8 | 0.0337 |
| PAC000197_g | Bacteria;Tenericutes;Mollicutes;PAC001057_o;PAC000197_f;PAC000197_g | 8 | 0.0337 |
| Blautia | Bacteria;Firmicutes;Clostridia;Clostridiales;Lachnospiraceae;Blautia | 7 | 0.0294 |
| PAC001043_g | Bacteria;Firmicutes;Clostridia;Clostridiales;Lachnospiraceae;PAC001043_g | 7 | 0.0294 |
| PAC001144_g | Bacteria;Firmicutes;Clostridia;Clostridiales;Ruminococcaceae;PAC001144_g | 7 | 0.0294 |
| PAC001573_g | Bacteria;Firmicutes;Clostridia;Clostridiales;Mogibacterium_f;PAC001573_g | 7 | 0.0294 |
| PAC002375_g | Bacteria;Firmicutes;Clostridia;Clostridiales;Lachnospiraceae;PAC002375_g | 7 | 0.0294 |
| Clostridium_g6 | Bacteria;Firmicutes;Erysipelotrichi;Erysipelotrichales;Erysipelotrichaceae;Clostridium_g6 | 6 | 0.0252 |
| LT706945_g | Bacteria;Proteobacteria;Deltaproteobacteria;Desulfovibrionales;Desulfovibrionaceae;LT706945_g | 6 | 0.0252 |
| PAC001270_g | Bacteria;Firmicutes;Clostridia;Clostridiales;Lachnospiraceae;PAC001270_g | 6 | 0.0252 |
| PAC001323_g | Bacteria;Tenericutes;Mollicutes;PAC001057_o;PAC000197_f;PAC001323_g | 6 | 0.0252 |
| PAC001609_g | Bacteria;Firmicutes;Clostridia;Clostridiales;Mogibacterium_f;PAC001609_g | 6 | 0.0252 |
| PAC001692_g | Bacteria;Bacteroidetes;Bacteroidia;Bacteroidales;Muribaculaceae;PAC001692_g | 6 | 0.0252 |
| Proteus | Bacteria;Proteobacteria;Gammaproteobacteria;Enterobacterales;Morganellaceae;Proteus | 5 | 0.021 |
| Coprococcus_g2 | Bacteria;Firmicutes;Clostridia;Clostridiales;Lachnospiraceae;Coprococcus_g2 | 5 | 0.021 |
| KE159605_g | Bacteria;Firmicutes;Clostridia;Clostridiales;Lachnospiraceae;KE159605_g | 5 | 0.021 |
| LLKB_g | Bacteria;Firmicutes;Clostridia;Clostridiales;Lachnospiraceae;LLKB_g | 5 | 0.021 |
| PAC001313_g | Bacteria;Firmicutes;Clostridia;Clostridiales;Ruminococcaceae;PAC001313_g | 5 | 0.021 |
| Acutalibacter | Bacteria;Firmicutes;Clostridia;Clostridiales;Ruminococcaceae;Acutalibacter | 4 | 0.0168 |
| Arthromitus | Bacteria;Firmicutes;Clostridia;Clostridiales;Clostridiaceae;Arthromitus | 4 | 0.0168 |
| Caproiciproducens | Bacteria;Firmicutes;Clostridia;Clostridiales;Ruminococcaceae;Caproiciproducens | 4 | 0.0168 |
| FTRU_g | Bacteria;Firmicutes;Clostridia;Clostridiales;Ruminococcaceae;FTRU_g | 4 | 0.0168 |
| PAC001127_g | Bacteria;Bacteroidetes;Bacteroidia;Bacteroidales;Muribaculaceae;PAC001127_g | 4 | 0.0168 |
| PAC001219_g | Bacteria;Firmicutes;Clostridia;Clostridiales;Christensenellaceae;PAC001219_g | 4 | 0.0168 |
| PAC001228_g | Bacteria;Firmicutes;Clostridia;Clostridiales;Lachnospiraceae;PAC001228_g | 4 | 0.0168 |
| PAC001360_g | Bacteria;Firmicutes;Clostridia;Clostridiales;Christensenellaceae;PAC001360_g | 4 | 0.0168 |
| PAC001512_g | Bacteria;Bacteroidetes;Bacteroidia;Bacteroidales;Muribaculaceae;PAC001512_g | 4 | 0.0168 |
| PAC002039_g | Bacteria;Firmicutes;Clostridia;Clostridiales;Lachnospiraceae;PAC002039_g | 4 | 0.0168 |
| Christensenella | Bacteria;Firmicutes;Clostridia;Clostridiales;Christensenellaceae;Christensenella | 3 | 0.0126 |
| Clostridioides | Bacteria;Firmicutes;Clostridia;Clostridiales;Peptostreptococcaceae;Clostridioides | 3 | 0.0126 |
| Longicatena | Bacteria;Firmicutes;Erysipelotrichi;Erysipelotrichales;Erysipelotrichaceae;Longicatena | 3 | 0.0126 |
| Mucispirillum | Bacteria;Deferribacteres;Deferribacteres_c;Deferribacterales;Deferribacteraceae;Mucispirillum | 3 | 0.0126 |
| Muribaculum | Bacteria;Bacteroidetes;Bacteroidia;Bacteroidales;Muribaculaceae;Muribaculum | 3 | 0.0126 |
| PAC000692_g | Bacteria;Firmicutes;Clostridia;Clostridiales;Lachnospiraceae;PAC000692_g | 3 | 0.0126 |
| PAC001108_g | Bacteria;Tenericutes;Mollicutes;PAC001057_o;PAC001057_f;PAC001108_g | 3 | 0.0126 |
| PAC001199_g | Bacteria;Firmicutes;Clostridia;Clostridiales;Lachnospiraceae;PAC001199_g | 3 | 0.0126 |
| PAC001437_g | Bacteria;Firmicutes;Clostridia;Clostridiales;Christensenellaceae;PAC001437_g | 3 | 0.0126 |
| PAC001472_g | Bacteria;Bacteroidetes;Bacteroidia;Bacteroidales;Muribaculaceae;PAC001472_g | 3 | 0.0126 |
| PAC001528_g | Bacteria;Firmicutes;Erysipelotrichi;Erysipelotrichales;Erysipelotrichaceae;PAC001528_g | 3 | 0.0126 |
| PAC001765_g | Bacteria;Bacteroidetes;Bacteroidia;Bacteroidales;Muribaculaceae;PAC001765_g | 3 | 0.0126 |
| Corynebacterium | Bacteria;Actinobacteria;Actinobacteria_c;Corynebacteriales;Corynebacteriaceae;Corynebacterium | 2 | 0.0084 |
| Enterobacter | Bacteria;Proteobacteria;Gammaproteobacteria;Enterobacterales;Enterobacteriaceae;Enterobacter | 2 | 0.0084 |
| Harryflintia | Bacteria;Firmicutes;Clostridia;Clostridiales;Ruminococcaceae;Harryflintia | 2 | 0.0084 |
| Marvinbryantia | Bacteria;Firmicutes;Clostridia;Clostridiales;Lachnospiraceae;Marvinbryantia | 2 | 0.0084 |
| Romboutsia | Bacteria;Firmicutes;Clostridia;Clostridiales;Peptostreptococcaceae;Romboutsia | 2 | 0.0084 |
| PAC000196_g | Bacteria;Firmicutes;Clostridia;Clostridiales;Lachnospiraceae;PAC000196_g | 2 | 0.0084 |
| PAC001287_g | Bacteria;Firmicutes;Clostridia;Clostridiales;Lachnospiraceae;PAC001287_g | 2 | 0.0084 |
| PAC001372_g | Bacteria;Firmicutes;Clostridia;Clostridiales;Lachnospiraceae;PAC001372_g | 2 | 0.0084 |
| PAC001612_g | Bacteria;Firmicutes;Clostridia;Clostridiales;Mogibacterium_f;PAC001612_g | 2 | 0.0084 |
| PAC001651_g | Bacteria;Firmicutes;Clostridia;Clostridiales;Ruminococcaceae;PAC001651_g | 2 | 0.0084 |
| PAC002390_g | Bacteria;Firmicutes;Clostridia;Clostridiales;Dehalobacterium_f;PAC002390_g | 2 | 0.0084 |
| PAC002400_g | Bacteria;Bacteroidetes;Bacteroidia;Bacteroidales;Muribaculaceae;PAC002400_g | 2 | 0.0084 |
| Ruminococcaceae_uc | Bacteria;Firmicutes;Clostridia;Clostridiales;Ruminococcaceae;Ruminococcaceae_uc | 2 | 0.0084 |
| Rhizobiales_uc | Bacteria;Proteobacteria;Alphaproteobacteria;Rhizobiales;Rhizobiales_uc | 1 | 0.0042 |
| Acinetobacter | Bacteria;Proteobacteria;Gammaproteobacteria;Pseudomonadales;Moraxellaceae;Acinetobacter | 1 | 0.0042 |
| Agathobaculum | Bacteria;Firmicutes;Clostridia;Clostridiales;Ruminococcaceae;Agathobaculum | 1 | 0.0042 |
| Alloprevotella | Bacteria;Bacteroidetes;Bacteroidia;Bacteroidales;Prevotellaceae;Alloprevotella | 1 | 0.0042 |
| Anaerostipes | Bacteria;Firmicutes;Clostridia;Clostridiales;Lachnospiraceae;Anaerostipes | 1 | 0.0042 |
| Arachidicoccus | Bacteria;Bacteroidetes;Sphingobacteriia;Sphingobacteriales;Chitinophagaceae;Arachidicoccus | 1 | 0.0042 |
| Catenulispora | Bacteria;Actinobacteria;Actinobacteria_c;Catenulisporales;Catenulisporaceae;Catenulispora | 1 | 0.0042 |
| Faecalibacterium | Bacteria;Firmicutes;Clostridia;Clostridiales;Ruminococcaceae;Faecalibacterium | 1 | 0.0042 |
| Geobacillus | Bacteria;Firmicutes;Bacilli;Bacillales;Bacillaceae;Geobacillus | 1 | 0.0042 |
| Haemophilus | Bacteria;Proteobacteria;Gammaproteobacteria;Pasteurellales;Pasteurellaceae;Haemophilus | 1 | 0.0042 |
| Holdemanella | Bacteria;Firmicutes;Erysipelotrichi;Erysipelotrichales;Erysipelotrichaceae;Holdemanella | 1 | 0.0042 |
| Luteimicrobium | Bacteria;Actinobacteria;Actinobacteria_c;Micrococcales;Promicromonosporaceae;Luteimicrobium | 1 | 0.0042 |
| Lysinibacillus | Bacteria;Firmicutes;Bacilli;Bacillales;Planococcaceae;Lysinibacillus | 1 | 0.0042 |
| Methylotenera | Bacteria;Proteobacteria;Betaproteobacteria;Methylophilales;Methylophilaceae;Methylotenera | 1 | 0.0042 |
| Parapedobacter | Bacteria;Bacteroidetes;Sphingobacteriia;Sphingobacteriales;Sphingobacteriaceae;Parapedobacter | 1 | 0.0042 |
| Parasutterella | Bacteria;Proteobacteria;Betaproteobacteria;Burkholderiales;Sutterellaceae;Parasutterella | 1 | 0.0042 |
| Phocea | Bacteria;Firmicutes;Clostridia;Clostridiales;Ruminococcaceae;Phocea | 1 | 0.0042 |
| Prevotella | Bacteria;Bacteroidetes;Bacteroidia;Bacteroidales;Prevotellaceae;Prevotella | 1 | 0.0042 |
| Rhodococcus | Bacteria;Actinobacteria;Actinobacteria_c;Corynebacteriales;Nocardiaceae;Rhodococcus | 1 | 0.0042 |
| Rhodopseudomonas | Bacteria;Proteobacteria;Alphaproteobacteria;Rhizobiales;Bradyrhizobiaceae;Rhodopseudomonas | 1 | 0.0042 |
| Turicibacter | Bacteria;Firmicutes;Erysipelotrichi;Erysipelotrichales;Erysipelotrichaceae;Turicibacter | 1 | 0.0042 |
| Clostridium_g35 | Bacteria;Firmicutes;Clostridia;Clostridiales;Lachnospiraceae;Clostridium_g35 | 1 | 0.0042 |
| EU132518_g | Bacteria;Actinobacteria;Actinobacteria_c;Frankiales;Frankiaceae;EU132518_g | 1 | 0.0042 |
| Eubacterium_g17 | Bacteria;Firmicutes;Clostridia;Clostridiales;Lachnospiraceae;Eubacterium_g17 | 1 | 0.0042 |
| Eubacterium_g5 | Bacteria;Firmicutes;Clostridia;Clostridiales;Lachnospiraceae;Eubacterium_g5 | 1 | 0.0042 |
| GQ264307_g | Bacteria;Bacteroidetes;Sphingobacteriia;Sphingobacteriales;Chitinophagaceae;GQ264307_g | 1 | 0.0042 |
| GU454944_g | Bacteria;Bacteroidetes;Cytophagia;Cytophagales;Cytophagaceae;GU454944_g | 1 | 0.0042 |
| HM124077_g | Bacteria;Bacteroidetes;Bacteroidia;Bacteroidales;Muribaculaceae;HM124077_g | 1 | 0.0042 |
| HM748739_g | Bacteria;Acidobacteria;Acidobacteriia;Acidobacteriales;Acidobacteriaceae;HM748739_g | 1 | 0.0042 |
| HQ158649_g | Bacteria;Bacteroidetes;Sphingobacteriia;Saprospirales;Saprospiraceae;HQ158649_g | 1 | 0.0042 |
| KE159538_g | Bacteria;Firmicutes;Clostridia;Clostridiales;Lachnospiraceae;KE159538_g | 1 | 0.0042 |
| PAC000195_g | Bacteria;Firmicutes;Clostridia;Clostridiales;Lachnospiraceae;PAC000195_g | 1 | 0.0042 |
| PAC001062_g | Bacteria;Bacteroidetes;Bacteroidia;Bacteroidales;Muribaculaceae;PAC001062_g | 1 | 0.0042 |
| PAC001115_g | Bacteria;Firmicutes;Clostridia;Clostridiales;Christensenellaceae;PAC001115_g | 1 | 0.0042 |
| PAC001149_g | Bacteria;Firmicutes;Clostridia;Clostridiales;Lachnospiraceae;PAC001149_g | 1 | 0.0042 |
| PAC001212_g | Bacteria;Firmicutes;Clostridia;Clostridiales;Lachnospiraceae;PAC001212_g | 1 | 0.0042 |
| PAC001225_g | Bacteria;Firmicutes;Clostridia;Clostridiales;Lachnospiraceae;PAC001225_g | 1 | 0.0042 |
| PAC001301_g | Bacteria;Firmicutes;Clostridia;Clostridiales;Christensenellaceae;PAC001301_g | 1 | 0.0042 |
| PAC001375_g | Bacteria;Firmicutes;Clostridia;Clostridiales;Lachnospiraceae;PAC001375_g | 1 | 0.0042 |
| PAC001386_g | Bacteria;Firmicutes;Clostridia;Clostridiales;Lachnospiraceae;PAC001386_g | 1 | 0.0042 |
| PAC001408_g | Bacteria;Firmicutes;Clostridia;Clostridiales;Lachnospiraceae;PAC001408_g | 1 | 0.0042 |
| PAC001457_g | Bacteria;Firmicutes;Clostridia;Clostridiales;Lachnospiraceae;PAC001457_g | 1 | 0.0042 |
| PAC001525_g | Bacteria;Firmicutes;Clostridia;Clostridiales;Lachnospiraceae;PAC001525_g | 1 | 0.0042 |
| PAC002042_g | Bacteria;Firmicutes;Clostridia;Clostridiales;Lachnospiraceae;PAC002042_g | 1 | 0.0042 |
| PAC002331_g | Bacteria;Bacteroidetes;Sphingobacteriia;Sphingobacteriales;Chitinophagaceae;PAC002331_g | 1 | 0.0042 |
| Ruminococcus_g4 | Bacteria;Firmicutes;Clostridia;Clostridiales;Lachnospiraceae;Ruminococcus_g4 | 1 | 0.0042 |

**Set 1_0.05%_G: Day 28_heme-SCP 0.05%_Genus**

| Taxon name | Taxonomy | Count | Proportion(%) |
|---|---|---|---|
| Akkermansia | Bacteria;Verrucomicrobia;Verrucomicrobiae;Verrucomicrobiales;Akkermansiaceae;Akkermansia | 3369 | 15.1579 |
| Bacteroides | Bacteria;Bacteroidetes;Bacteroidia;Bacteroidales;Bacteroidaceae;Bacteroides | 2244 | 10.0963 |
| PAC001118_g | Bacteria;Firmicutes;Clostridia;Clostridiales;Lachnospiraceae;PAC001118_g | 1410 | 6.3439 |
| Pseudoflavonifractor | Bacteria;Firmicutes;Clostridia;Clostridiales;Ruminococcaceae;Pseudoflavonifractor | 1237 | 5.5656 |
| Lactobacillus | Bacteria;Firmicutes;Bacilli;Lactobacillales;Lactobacillaceae;Lactobacillus | 1189 | 5.3496 |
| KE159600_g | Bacteria;Firmicutes;Clostridia;Clostridiales;Lachnospiraceae;KE159600_g | 1070 | 4.8142 |
| Oscillibacter | Bacteria;Firmicutes;Clostridia;Clostridiales;Ruminococcaceae;Oscillibacter | 838 | 3.7704 |
| PAC001516_g | Bacteria;Firmicutes;Clostridia;Clostridiales;Lachnospiraceae;PAC001516_g | 770 | 3.4644 |
| Acetatifactor | Bacteria;Firmicutes;Clostridia;Clostridiales;Lachnospiraceae;Acetatifactor | 676 | 3.0415 |
| PAC000664_g | Bacteria;Firmicutes;Clostridia;Clostridiales;Lachnospiraceae;PAC000664_g | 568 | 2.5556 |
| PAC001402_g | Bacteria;Firmicutes;Clostridia;Clostridiales;Ruminococcaceae;PAC001402_g | 545 | 2.4521 |
| KE159810_g | Bacteria;Firmicutes;Clostridia;Clostridiales;Lachnospiraceae;KE159810_g | 515 | 2.3171 |
| Clostridium_g21 | Bacteria;Firmicutes;Clostridia;Clostridiales;Lachnospiraceae;Clostridium_g21 | 440 | 1.9797 |
| PAC000661_g | Bacteria;Firmicutes;Clostridia;Clostridiales;Ruminococcaceae;PAC000661_g | 410 | 1.8447 |
| Anaerotignum | Bacteria;Firmicutes;Clostridia;Clostridiales;Lachnospiraceae;Anaerotignum | 401 | 1.8042 |
| Lactococcus | Bacteria;Firmicutes;Bacilli;Lactobacillales;Streptococcaceae;Lactococcus | 372 | 1.6737 |
| PAC002482_g | Bacteria;Bacteroidetes;Bacteroidia;Bacteroidales;AC160630_f;PAC002482_g | 332 | 1.4937 |
| PAC001141_g | Bacteria;Firmicutes;Clostridia;Clostridiales;Christensenellaceae;PAC001141_g | 330 | 1.4847 |
| PAC001452_g | Bacteria;Firmicutes;Clostridia;Clostridiales;Lachnospiraceae;PAC001452_g | 277 | 1.2463 |
| Anaerotruncus | Bacteria;Firmicutes;Clostridia;Clostridiales;Ruminococcaceae;Anaerotruncus | 265 | 1.1923 |
| PAC001738_g | Bacteria;Firmicutes;Clostridia;Clostridiales;Lachnospiraceae;PAC001738_g | 263 | 1.1833 |
| Odoribacter | Bacteria;Bacteroidetes;Bacteroidia;Bacteroidales;Odoribacteraceae;Odoribacter | 259 | 1.1653 |
| PAC001074_g | Bacteria;Bacteroidetes;Bacteroidia;Bacteroidales;Muribaculaceae;PAC001074_g | 258 | 1.1608 |
| Helicobacter | Bacteria;Proteobacteria;Epsilonproteobacteria;Campylobacterales;Helicobacteraceae;Helicobacter | 254 | 1.1428 |
| Bilophila | Bacteria;Proteobacteria;Deltaproteobacteria;Desulfovibrionales;Desulfovibrionaceae;Bilophila | 246 | 1.1068 |
| KE159571_g | Bacteria;Firmicutes;Clostridia;Clostridiales;Lachnospiraceae;KE159571_g | 232 | 1.0438 |
| Alistipes | Bacteria;Bacteroidetes;Bacteroidia;Bacteroidales;Rikenellaceae;Alistipes | 211 | 0.9493 |
| Frisingicoccus | Bacteria;Firmicutes;Clostridia;Clostridiales;Lachnospiraceae;Frisingicoccus | 205 | 0.9223 |
| Eubacterium_g23 | Bacteria;Firmicutes;Clostridia;Clostridiales;Ruminococcaceae;Eubacterium_g23 | 162 | 0.7289 |
| PAC001112_g | Bacteria;Bacteroidetes;Bacteroidia;Bacteroidales;Muribaculaceae;PAC001112_g | 156 | 0.7019 |
| PAC000198_g | Bacteria;Bacteroidetes;Bacteroidia;Bacteroidales;Muribaculaceae;PAC000198_g | 140 | 0.6299 |
| PAC001544_g | Bacteria;Firmicutes;Clostridia;Clostridiales;Lachnospiraceae;PAC001544_g | 130 | 0.5849 |
| PAC001092_g | Bacteria;Firmicutes;Clostridia;Clostridiales;Lachnospiraceae;PAC001092_g | 114 | 0.5129 |
| KE159538_g | Bacteria;Firmicutes;Clostridia;Clostridiales;Lachnospiraceae;KE159538_g | 102 | 0.4589 |
| PAC001400_g | Bacteria;Firmicutes;Clostridia;Clostridiales;Peptococcaceae;PAC001400_g | 101 | 0.4544 |
| Desulfovibrio | Bacteria;Proteobacteria;Deltaproteobacteria;Desulfovibrionales;Desulfovibrionaceae;Desulfovibrio | 99 | 0.4454 |
| KE159797_g | Bacteria;Firmicutes;Clostridia;Clostridiales;Lachnospiraceae;KE159797_g | 93 | 0.4184 |
| PAC001068_g | Bacteria;Bacteroidetes;Bacteroidia;Bacteroidales;Muribaculaceae;PAC001068_g | 91 | 0.4094 |
| Eubacterium_g6 | Bacteria;Firmicutes;Clostridia;Clostridiales;Lachnospiraceae;Eubacterium_g6 | 82 | 0.3689 |
| PAC001165_g | Bacteria;Firmicutes;Clostridia;Clostridiales;Lachnospiraceae;PAC001165_g | 82 | 0.3689 |
| Paludicola | Bacteria;Firmicutes;Clostridia;Clostridiales;Ruminococcaceae;Paludicola | 77 | 0.3464 |
| PAC001221_g | Bacteria;Firmicutes;Clostridia;Clostridiales;Dehalobacterium_f;PAC001221_g | 73 | 0.3284 |
| Parabacteroides | Bacteria;Bacteroidetes;Bacteroidia;Bacteroidales;Porphyromonadaceae;Parabacteroides | 70 | 0.3149 |
| LT706945_g | Bacteria;Proteobacteria;Deltaproteobacteria;Desulfovibrionales;Desulfovibrionaceae;LT706945_g | 63 | 0.2835 |
| PAC001219_g | Bacteria;Firmicutes;Clostridia;Clostridiales;Christensenellaceae;PAC001219_g | 61 | 0.2745 |
| PAC001500_g | Bacteria;Firmicutes;Clostridia;Clostridiales;Peptococcaceae;PAC001500_g | 61 | 0.2745 |
| Clostridium_g6 | Bacteria;Firmicutes;Erysipelotrichi;Erysipelotrichales;Erysipelotrichaceae;Clostridium_g6 | 60 | 0.27 |
| Sporobacter | Bacteria;Firmicutes;Clostridia;Clostridiales;Ruminococcaceae;Sporobacter | 58 | 0.261 |
| PAC000186_g | Bacteria;Bacteroidetes;Bacteroidia;Bacteroidales;Muribaculaceae;PAC000186_g | 51 | 0.2295 |
| LARJ_g | Bacteria;Proteobacteria;Alphaproteobacteria;Rhodospirillales;Rhodospirillaceae;LARJ_g | 49 | 0.2205 |
| PAC001385_g | Bacteria;Firmicutes;Clostridia;Clostridiales;Lachnospiraceae;PAC001385_g | 48 | 0.216 |
| Corynebacterium | Bacteria;Actinobacteria;Actinobacteria_c;Corynebacteriales;Corynebacteriaceae;Corynebacterium | 45 | 0.2025 |
| PAC001103_g | Bacteria;Firmicutes;Clostridia;Clostridiales;Lachnospiraceae;PAC001103_g | 45 | 0.2025 |
| PAC001066_g | Bacteria;Bacteroidetes;Bacteroidia;Bacteroidales;Muribaculaceae;PAC001066_g | 43 | 0.1935 |
| PAC001043_g | Bacteria;Firmicutes;Clostridia;Clostridiales;Lachnospiraceae;PAC001043_g | 40 | 0.18 |
| PAC001524_g | Bacteria;Firmicutes;Clostridia;Clostridiales;Lachnospiraceae;PAC001524_g | 38 | 0.171 |
| PAC001138_g | Bacteria;Firmicutes;Clostridia;Clostridiales;Lachnospiraceae;PAC001138_g | 37 | 0.1665 |
| PAC001457_g | Bacteria;Firmicutes;Clostridia;Clostridiales;Lachnospiraceae;PAC001457_g | 36 | 0.162 |
| Caproiciproducens | Bacteria;Firmicutes;Clostridia;Clostridiales;Ruminococcaceae;Caproiciproducens | 33 | 0.1485 |
| PAC001485_g | Bacteria;Bacteroidetes;Bacteroidia;Bacteroidales;Muribaculaceae;PAC001485_g | 33 | 0.1485 |
| PAC000692_g | Bacteria;Firmicutes;Clostridia;Clostridiales;Lachnospiraceae;PAC000692_g | 32 | 0.144 |
| Mucispirillum | Bacteria;Deferribacteres;Deferribacteres_c;Deferribacterales;Deferribacteraceae;Mucispirillum | 30 | 0.135 |
| KE159605_g | Bacteria;Firmicutes;Clostridia;Clostridiales;Lachnospiraceae;KE159605_g | 30 | 0.135 |
| PAC000748_g | Bacteria;Firmicutes;Clostridia;Clostridiales;Ruminococcaceae;PAC000748_g | 30 | 0.135 |
| PAC001228_g | Bacteria;Firmicutes;Clostridia;Clostridiales;Lachnospiraceae;PAC001228_g | 30 | 0.135 |
| Clostridium_g24 | Bacteria;Firmicutes;Clostridia;Clostridiales;Lachnospiraceae;Clostridium_g24 | 28 | 0.126 |
| PAC000197_g | Bacteria;Tenericutes;Mollicutes;PAC001057_o;PAC000197_f;PAC000197_g | 27 | 0.1215 |
| Eubacterium_g4 | Bacteria;Firmicutes;Clostridia;Clostridiales;Lachnospiraceae;Eubacterium_g4 | 26 | 0.117 |
| PAC002039_g | Bacteria;Firmicutes;Clostridia;Clostridiales;Lachnospiraceae;PAC002039_g | 25 | 0.1125 |
| Harryflintia | Bacteria;Firmicutes;Clostridia;Clostridiales;Ruminococcaceae;Harryflintia | 21 | 0.0945 |
| PAC001372_g | Bacteria;Firmicutes;Clostridia;Clostridiales;Lachnospiraceae;PAC001372_g | 20 | 0.09 |
| Lachnospiraceae_uc | Bacteria;Firmicutes;Clostridia;Clostridiales;Lachnospiraceae;Lachnospiraceae_uc | 20 | 0.09 |
| PAC001108_g | Bacteria;Tenericutes;Mollicutes;PAC001057_o;PAC001057_f;PAC001108_g | 18 | 0.081 |
| PAC001778_g | Bacteria;Firmicutes;Clostridia;Clostridiales;Ruminococcaceae;PAC001778_g | 18 | 0.081 |
| Arthromitus | Bacteria;Firmicutes;Clostridia;Clostridiales;Clostridiaceae;Arthromitus | 15 | 0.0675 |
| PAC001308_g | Bacteria;Firmicutes;Clostridia;Clostridiales;Lachnospiraceae;PAC001308_g | 14 | 0.063 |
| Acutalibacter | Bacteria;Firmicutes;Clostridia;Clostridiales;Ruminococcaceae;Acutalibacter | 12 | 0.054 |
| Enterorhabdus | Bacteria;Actinobacteria;Coriobacteriia;Coriobacteriales;Coriobacteriaceae;Enterorhabdus | 11 | 0.0495 |
| Massilioclostridium | Bacteria;Firmicutes;Clostridia;Clostridiales;Ruminococcaceae;Massilioclostridium | 11 | 0.0495 |
| Escherichia | Bacteria;Proteobacteria;Gammaproteobacteria;Enterobacterales;Enterobacteriaceae;Escherichia | 9 | 0.0405 |
| Acholeplasma_g2 | Bacteria;Tenericutes;Mollicutes;Acholeplasmatales;Acholeplasmataceae;Acholeplasma_g2 | 9 | 0.0405 |
| PAC001109_g | Bacteria;Tenericutes;Mollicutes;PAC001057_o;PAC001057_f;PAC001109_g | 9 | 0.0405 |
| PAC001144_g | Bacteria;Firmicutes;Clostridia;Clostridiales;Ruminococcaceae;PAC001144_g | 9 | 0.0405 |
| Christensenella | Bacteria;Firmicutes;Clostridia;Clostridiales;Christensenellaceae;Christensenella | 8 | 0.036 |
| Coprococcus_g2 | Bacteria;Firmicutes;Clostridia;Clostridiales;Lachnospiraceae;Coprococcus_g2 | 7 | 0.0315 |
| PAC002314_g | Bacteria;Firmicutes;Erysipelotrichi;Erysipelotrichales;Erysipelotrichaceae;PAC002314_g | 7 | 0.0315 |
| PAC001063_g | Bacteria;Bacteroidetes;Bacteroidia;Bacteroidales;Muribaculaceae;PAC001063_g | 6 | 0.027 |
| PAC001199_g | Bacteria;Firmicutes;Clostridia;Clostridiales;Lachnospiraceae;PAC001199_g | 6 | 0.027 |
| PAC001270_g | Bacteria;Firmicutes;Clostridia;Clostridiales;Lachnospiraceae;PAC001270_g | 6 | 0.027 |
| PAC001360_g | Bacteria;Firmicutes;Clostridia;Clostridiales;Christensenellaceae;PAC001360_g | 6 | 0.027 |
| PAC001573_g | Bacteria;Firmicutes;Clostridia;Clostridiales;Mogibacterium_f;PAC001573_g | 6 | 0.027 |
| PAC001609_g | Bacteria;Firmicutes;Clostridia;Clostridiales;Mogibacterium_f;PAC001609_g | 6 | 0.027 |
| Ruminococcaceae_uc | Bacteria;Firmicutes;Clostridia;Clostridiales;Ruminococcaceae;Ruminococcaceae_uc | 6 | 0.027 |
| Agathobaculum | Bacteria;Firmicutes;Clostridia;Clostridiales;Ruminococcaceae;Agathobaculum | 5 | 0.0225 |
| Streptococcus | Bacteria;Firmicutes;Bacilli;Lactobacillales;Streptococcaceae;Streptococcus | 5 | 0.0225 |
| Clostridium_g35 | Bacteria;Firmicutes;Clostridia;Clostridiales;Lachnospiraceae;Clostridium_g35 | 5 | 0.0225 |
| PAC001201_g | Bacteria;Firmicutes;Clostridia;Clostridiales;Lachnospiraceae;PAC001201_g | 5 | 0.0225 |
| PAC001225_g | Bacteria;Firmicutes;Clostridia;Clostridiales;Lachnospiraceae;PAC001225_g | 5 | 0.0225 |
| PAC001362_g | Bacteria;Firmicutes;Erysipelotrichi;Erysipelotrichales;Erysipelotrichaceae;PAC001362_g | 5 | 0.0225 |
| PAC001528_g | Bacteria;Firmicutes;Erysipelotrichi;Erysipelotrichales;Erysipelotrichaceae;PAC001528_g | 5 | 0.0225 |
| Emergencia | Bacteria;Firmicutes;Clostridia;Clostridiales;Mogibacterium_f;Emergencia | 4 | 0.018 |
| PAC000671_g | Bacteria;Firmicutes;Clostridia;Clostridiales;Lachnospiraceae;PAC000671_g | 4 | 0.018 |
| PAC001588_g | Bacteria;Firmicutes;Clostridia;Clostridiales;Lachnospiraceae;PAC001588_g | 4 | 0.018 |
| PAC001612_g | Bacteria;Firmicutes;Clostridia;Clostridiales;Mogibacterium_f;PAC001612_g | 4 | 0.018 |
| PAC002460_g | Bacteria;Firmicutes;Clostridia;Clostridiales;Lachnospiraceae;PAC002460_g | 4 | 0.018 |
| Blautia | Bacteria;Firmicutes;Clostridia;Clostridiales;Lachnospiraceae;Blautia | 3 | 0.0135 |
| Faecalibacterium | Bacteria;Firmicutes;Clostridia;Clostridiales;Ruminococcaceae;Faecalibacterium | 3 | 0.0135 |
| EU461719_g | Bacteria;Firmicutes;Clostridia;Clostridiales;Lachnospiraceae;EU461719_g | 3 | 0.0135 |
| PAC000196_g | Bacteria;Firmicutes;Clostridia;Clostridiales;Lachnospiraceae;PAC000196_g | 3 | 0.0135 |
| PAC001116_g | Bacteria;Firmicutes;Clostridia;Clostridiales;Lachnospiraceae;PAC001116_g | 3 | 0.0135 |
| PAC001149_g | Bacteria;Firmicutes;Clostridia;Clostridiales;Lachnospiraceae;PAC001149_g | 3 | 0.0135 |
| PAC001313_g | Bacteria;Firmicutes;Clostridia;Clostridiales;Ruminococcaceae;PAC001313_g | 3 | 0.0135 |
| PAC001386_g | Bacteria;Firmicutes;Clostridia;Clostridiales;Lachnospiraceae;PAC001386_g | 3 | 0.0135 |
| PAC002196_g | Bacteria;Firmicutes;Clostridia;Clostridiales;Lachnospiraceae;PAC002196_g | 3 | 0.0135 |
| PAC002471_g | Bacteria;Firmicutes;Clostridia;Clostridiales;Lachnospiraceae;PAC002471_g | 3 | 0.0135 |
| Agathobacter | Bacteria;Firmicutes;Clostridia;Clostridiales;Lachnospiraceae;Agathobacter | 2 | 0.009 |
| Bacillus | Bacteria;Firmicutes;Bacilli;Bacillales;Bacillaceae;Bacillus | 2 | 0.009 |
| Longicatena | Bacteria;Firmicutes;Erysipelotrichi;Erysipelotrichales;Erysipelotrichaceae;Longicatena | 2 | 0.009 |
| Monoglobus | Bacteria;Firmicutes;Clostridia;Clostridiales;Ruminococcaceae;Monoglobus | 2 | 0.009 |
| Parasutterella | Bacteria;Proteobacteria;Betaproteobacteria;Burkholderiales;Sutterellaceae;Parasutterella | 2 | 0.009 |
| Sphingomonas | Bacteria;Proteobacteria;Alphaproteobacteria;Sphingomonadales;Sphingomonadaceae;Sphingomonas | 2 | 0.009 |
| Eubacterium_g17 | Bacteria;Firmicutes;Clostridia;Clostridiales;Lachnospiraceae;Eubacterium_g17 | 2 | 0.009 |
| PAC001296_g | Bacteria;Firmicutes;Clostridia;Clostridiales;Lachnospiraceae;PAC001296_g | 2 | 0.009 |
| PAC001323_g | Bacteria;Tenericutes;Mollicutes;PAC001057_o;PAC000197_f;PAC001323_g | 2 | 0.009 |
| PAC001651_g | Bacteria;Firmicutes;Clostridia;Clostridiales;Ruminococcaceae;PAC001651_g | 2 | 0.009 |
| PAC002042_g | Bacteria;Firmicutes;Clostridia;Clostridiales;Lachnospiraceae;PAC002042_g | 2 | 0.009 |
| PAC000197_f_uc | Bacteria;Tenericutes;Mollicutes;PAC001057_o;PAC000197_f;PAC000197_f_uc | 2 | 0.009 |
| Cloacibacterium | Bacteria;Bacteroidetes;Flavobacteria;Flavobacteriales;Flavobacteriaceae;Cloacibacterium | 1 | 0.0045 |
| Clostridium | Bacteria;Firmicutes;Clostridia;Clostridiales;Clostridiaceae;Clostridium | 1 | 0.0045 |
| Enterococcus | Bacteria;Firmicutes;Bacilli;Lactobacillales;Enterococcaceae;Enterococcus | 1 | 0.0045 |
| Flavobacterium | Bacteria;Bacteroidetes;Flavobacteria;Flavobacteriales;Flavobacteriaceae;Flavobacterium | 1 | 0.0045 |
| Haemophilus | Bacteria;Proteobacteria;Gammaproteobacteria;Pasteurellales;Pasteurellaceae;Haemophilus | 1 | 0.0045 |
| Leifsonia | Bacteria;Actinobacteria;Actinobacteria_c;Micrococcales;Microbacteriaceae;Leifsonia | 1 | 0.0045 |
| Lysinibacillus | Bacteria;Firmicutes;Bacilli;Bacillales;Planococcaceae;Lysinibacillus | 1 | 0.0045 |
| Marvinbryantia | Bacteria;Firmicutes;Clostridia;Clostridiales;Lachnospiraceae;Marvinbryantia | 1 | 0.0045 |
| Muribaculum | Bacteria;Bacteroidetes;Bacteroidia;Bacteroidales;Muribaculaceae;Muribaculum | 1 | 0.0045 |
| Nakamurella | Bacteria;Actinobacteria;Actinobacteria_c;Frankiales;Nakamurellaceae;Nakamurella | 1 | 0.0045 |
| Neisseria | Bacteria;Proteobacteria;Betaproteobacteria;Neisseriales;Neisseriaceae;Neisseria | 1 | 0.0045 |
| Paenibacillus | Bacteria;Firmicutes;Bacilli;Bacillales;Paenibacillaceae;Paenibacillus | 1 | 0.0045 |
| Parafilimonas | Bacteria;Bacteroidetes;Sphingobacteriia;Sphingobacteriales;Chitinophagaceae;Parafilimonas | 1 | 0.0045 |
| Prevotella | Bacteria;Bacteroidetes;Bacteroidia;Bacteroidales;Prevotellaceae;Prevotella | 1 | 0.0045 |
| Rhodococcus | Bacteria;Actinobacteria;Actinobacteria_c;Corynebacteriales;Nocardiaceae;Rhodococcus | 1 | 0.0045 |
| Romboutsia | Bacteria;Firmicutes;Clostridia;Clostridiales;Peptostreptococcaceae;Romboutsia | 1 | 0.0045 |
| Terrimonas | Bacteria;Bacteroidetes;Sphingobacteriia;Sphingobacteriales;Chitinophagaceae;Terrimonas | 1 | 0.0045 |
| AP011765_g | Bacteria;Proteobacteria;Deltaproteobacteria;Myxococcales;Polyangiaceae;AP011765_g | 1 | 0.0045 |
| DQ129389_g | Bacteria;Chloroflexi;Thermomicrobia;DQ129389_o;DQ129389_f;DQ129389_g | 1 | 0.0045 |
| GQ451199_g | Bacteria;Firmicutes;Clostridia;Clostridiales;Ruminococcaceae;GQ451199_g | 1 | 0.0045 |
| HM036033_g | Bacteria;Firmicutes;Bacilli;Bacillales;Thermoactinomycetaceae;HM036033_g | 1 | 0.0045 |
| HQ158649_g | Bacteria;Bacteroidetes;Sphingobacteriia;Saprospirales;Saprospiraceae;HQ158649_g | 1 | 0.0045 |
| LLKB_g | Bacteria;Firmicutes;Clostridia;Clostridiales;Lachnospiraceae;LLKB_g | 1 | 0.0045 |
| Mycoplasma_g10 | Bacteria;Tenericutes;Mollicutes;Mycoplasmatales;Mycoplasmataceae;Mycoplasma_g10 | 1 | 0.0045 |
| PAC000244_g | Bacteria;Proteobacteria;Deltaproteobacteria;Myxococcales;Polyangiaceae;PAC000244_g | 1 | 0.0045 |
| PAC000683_g | Bacteria;Firmicutes;Clostridia;Clostridiales;Ruminococcaceae;PAC000683_g | 1 | 0.0045 |
| PAC001062_g | Bacteria;Bacteroidetes;Bacteroidia;Bacteroidales;Muribaculaceae;PAC001062_g | 1 | 0.0045 |
| PAC001091_g | Bacteria;Firmicutes;Clostridia;Clostridiales;Lachnospiraceae;PAC001091_g | 1 | 0.0045 |
| PAC001127_g | Bacteria;Bacteroidetes;Bacteroidia;Bacteroidales;Muribaculaceae;PAC001127_g | 1 | 0.0045 |
| PAC001212_g | Bacteria;Firmicutes;Clostridia;Clostridiales;Lachnospiraceae;PAC001212_g | 1 | 0.0045 |
| PAC001236_g | Bacteria;Firmicutes;Clostridia;Clostridiales;Mogibacterium_f;PAC001236_g | 1 | 0.0045 |
| PAC001375_g | Bacteria;Firmicutes;Clostridia;Clostridiales;Lachnospiraceae;PAC001375_g | 1 | 0.0045 |
| PAC001456_g | Bacteria;Firmicutes;Clostridia;Clostridiales;Lachnospiraceae;PAC001456_g | 1 | 0.0045 |
| PAC001512_g | Bacteria;Bacteroidetes;Bacteroidia;Bacteroidales;Muribaculaceae;PAC001512_g | 1 | 0.0045 |
| PAC001525_g | Bacteria;Firmicutes;Clostridia;Clostridiales;Lachnospiraceae;PAC001525_g | 1 | 0.0045 |
| PAC001599_g | Bacteria;Firmicutes;Clostridia;Clostridiales;Ruminococcaceae;PAC001599_g | 1 | 0.0045 |
| PAC001681_g | Bacteria;Firmicutes;Clostridia;Clostridiales;Lachnospiraceae;PAC001681_g | 1 | 0.0045 |
| PAC001765_g | Bacteria;Bacteroidetes;Bacteroidia;Bacteroidales;Muribaculaceae;PAC001765_g | 1 | 0.0045 |
| PAC002331_g | Bacteria;Bacteroidetes;Sphingobacteriia;Sphingobacteriales;Chitinophagaceae;PAC002331_g | 1 | 0.0045 |
| PAC002390_g | Bacteria;Firmicutes;Clostridia;Clostridiales;Dehalobacterium_f;PAC002390_g | 1 | 0.0045 |

**Set 1_0.5%_G: Day 28_heme-SCP 0.5%_Genus**

| Taxon name | Taxonomy | Count | Proportion(%) |
|---|---|---|---|
| Bacteroides | Bacteria;Bacteroidetes;Bacteroidia;Bacteroidales;Bacteroidaceae;Bacteroides | 4281 | 19.8571 |
| Lactobacillus | Bacteria;Firmicutes;Bacilli;Lactobacillales;Lactobacillaceae;Lactobacillus | 3956 | 18.3496 |
| Akkermansia | Bacteria;Verrucomicrobia;Verrucomicrobiae;Verrucomicrobiales;Akkermansiaceae;Akkermansia | 2442 | 11.3271 |
| KE159600_g | Bacteria;Firmicutes;Clostridia;Clostridiales;Lachnospiraceae;KE159600_g | 621 | 2.8805 |
| Oscillibacter | Bacteria;Firmicutes;Clostridia;Clostridiales;Ruminococcaceae;Oscillibacter | 575 | 2.6671 |
| Pseudoflavonifractor | Bacteria;Firmicutes;Clostridia;Clostridiales;Ruminococcaceae;Pseudoflavonifractor | 564 | 2.6161 |
| PAC000198_g | Bacteria;Bacteroidetes;Bacteroidia;Bacteroidales;Muribaculaceae;PAC000198_g | 505 | 2.3424 |
| PAC001141_g | Bacteria;Firmicutes;Clostridia;Clostridiales;Christensenellaceae;PAC001141_g | 493 | 2.2867 |
| PAC001118_g | Bacteria;Firmicutes;Clostridia;Clostridiales;Lachnospiraceae;PAC001118_g | 482 | 2.2357 |
| Alistipes | Bacteria;Bacteroidetes;Bacteroidia;Bacteroidales;Rikenellaceae;Alistipes | 402 | 1.8647 |
| PAC001074_g | Bacteria;Bacteroidetes;Bacteroidia;Bacteroidales;Muribaculaceae;PAC001074_g | 339 | 1.5724 |
| PAC000661_g | Bacteria;Firmicutes;Clostridia;Clostridiales;Ruminococcaceae;PAC000661_g | 315 | 1.4611 |
| Corynebacterium | Bacteria;Actinobacteria;Actinobacteria_c;Corynebacteriales;Corynebacteriaceae;Corynebacterium | 307 | 1.424 |
| KE159571_g | Bacteria;Firmicutes;Clostridia;Clostridiales;Lachnospiraceae;KE159571_g | 306 | 1.4194 |
| Lactococcus | Bacteria;Firmicutes;Bacilli;Lactobacillales;Streptococcaceae;Lactococcus | 272 | 1.2617 |
| Clostridium_g21 | Bacteria;Firmicutes;Clostridia;Clostridiales;Lachnospiraceae;Clostridium_g21 | 261 | 1.2106 |
| PAC002482_g | Bacteria;Bacteroidetes;Bacteroidia;Bacteroidales;AC160630_f;PAC002482_g | 239 | 1.1086 |
| Anaerotignum | Bacteria;Firmicutes;Clostridia;Clostridiales;Lachnospiraceae;Anaerotignum | 223 | 1.0344 |
| Bilophila | Bacteria;Proteobacteria;Deltaproteobacteria;Desulfovibrionales;Desulfovibrionaceae;Bilophila | 218 | 1.0112 |
| Odoribacter | Bacteria;Bacteroidetes;Bacteroidia;Bacteroidales;Odoribacteraceae;Odoribacter | 209 | 0.9694 |
| PAC001112_g | Bacteria;Bacteroidetes;Bacteroidia;Bacteroidales;Muribaculaceae;PAC001112_g | 187 | 0.8674 |
| PAC001516_g | Bacteria;Firmicutes;Clostridia;Clostridiales;Lachnospiraceae;PAC001516_g | 185 | 0.8581 |
| PAC001400_g | Bacteria;Firmicutes;Clostridia;Clostridiales;Peptococcaceae;PAC001400_g | 169 | 0.7839 |
| PAC001738_g | Bacteria;Firmicutes;Clostridia;Clostridiales;Lachnospiraceae;PAC001738_g | 161 | 0.7468 |
| Clostridium_g6 | Bacteria;Firmicutes;Erysipelotrichi;Erysipelotrichales;Erysipelotrichaceae;Clostridium_g6 | 160 | 0.7421 |
| Frisingicoccus | Bacteria;Firmicutes;Clostridia;Clostridiales;Lachnospiraceae;Frisingicoccus | 159 | 0.7375 |
| PAC001402_g | Bacteria;Firmicutes;Clostridia;Clostridiales;Ruminococcaceae;PAC001402_g | 154 | 0.7143 |
| Acetatifactor | Bacteria;Firmicutes;Clostridia;Clostridiales;Lachnospiraceae;Acetatifactor | 148 | 0.6865 |
| PAC000683_g | Bacteria;Firmicutes;Clostridia;Clostridiales;Ruminococcaceae;PAC000683_g | 140 | 0.6494 |
| PAC001452_g | Bacteria;Firmicutes;Clostridia;Clostridiales;Lachnospiraceae;PAC001452_g | 138 | 0.6401 |
| Anaerotruncus | Bacteria;Firmicutes;Clostridia;Clostridiales;Ruminococcaceae;Anaerotruncus | 128 | 0.5937 |
| PAC001092_g | Bacteria;Firmicutes;Clostridia;Clostridiales;Lachnospiraceae;PAC001092_g | 115 | 0.5334 |
| PAC001219_g | Bacteria;Firmicutes;Clostridia;Clostridiales;Christensenellaceae;PAC001219_g | 102 | 0.4731 |
| Paludicola | Bacteria;Firmicutes;Clostridia;Clostridiales;Ruminococcaceae;Paludicola | 93 | 0.4314 |
| KE159538_g | Bacteria;Firmicutes;Clostridia;Clostridiales;Lachnospiraceae;KE159538_g | 89 | 0.4128 |
| Sporobacter | Bacteria;Firmicutes;Clostridia;Clostridiales;Ruminococcaceae;Sporobacter | 85 | 0.3943 |
| Helicobacter | Bacteria;Proteobacteria;Epsilonproteobacteria;Campylobacterales;Helicobacteraceae;Helicobacter | 84 | 0.3896 |
| PAC000664_g | Bacteria;Firmicutes;Clostridia;Clostridiales;Lachnospiraceae;PAC000664_g | 77 | 0.3572 |
| PAC001068_g | Bacteria;Bacteroidetes;Bacteroidia;Bacteroidales;Muribaculaceae;PAC001068_g | 76 | 0.3525 |
| PAC001457_g | Bacteria;Firmicutes;Clostridia;Clostridiales;Lachnospiraceae;PAC001457_g | 74 | 0.3432 |
| Faecalibacterium | Bacteria;Firmicutes;Clostridia;Clostridiales;Ruminococcaceae;Faecalibacterium | 69 | 0.3201 |
| PAC001228_g | Bacteria;Firmicutes;Clostridia;Clostridiales;Lachnospiraceae;PAC001228_g | 67 | 0.3108 |
| Parabacteroides | Bacteria;Bacteroidetes;Bacteroidia;Bacteroidales;Porphyromonadaceae;Parabacteroides | 66 | 0.3061 |
| Citrobacter | Bacteria;Proteobacteria;Gammaproteobacteria;Enterobacterales;Enterobacteriaceae;Citrobacter | 62 | 0.2876 |
| PAC000186_g | Bacteria;Bacteroidetes;Bacteroidia;Bacteroidales;Muribaculaceae;PAC000186_g | 62 | 0.2876 |
| Prevotella | Bacteria;Bacteroidetes;Bacteroidia;Bacteroidales;Prevotellaceae;Prevotella | 59 | 0.2737 |
| PAC000197_g | Bacteria;Tenericutes;Mollicutes;PAC001057_o;PAC000197_f;PAC000197_g | 54 | 0.2505 |
| PAC001500_g | Bacteria;Firmicutes;Clostridia;Clostridiales;Peptococcaceae;PAC001500_g | 54 | 0.2505 |
| LARJ_g | Bacteria;Proteobacteria;Alphaproteobacteria;Rhodospirillales;Rhodospirillaceae;LARJ_g | 53 | 0.2458 |
| Escherichia | Bacteria;Proteobacteria;Gammaproteobacteria;Enterobacterales;Enterobacteriaceae;Escherichia | 51 | 0.2366 |
| Blautia | Bacteria;Firmicutes;Clostridia;Clostridiales;Lachnospiraceae;Blautia | 50 | 0.2319 |
| PAC001360_g | Bacteria;Firmicutes;Clostridia;Clostridiales;Christensenellaceae;PAC001360_g | 50 | 0.2319 |
| PAC001063_g | Bacteria;Bacteroidetes;Bacteroidia;Bacteroidales;Muribaculaceae;PAC001063_g | 48 | 0.2226 |
| PAC001165_g | Bacteria;Firmicutes;Clostridia;Clostridiales;Lachnospiraceae;PAC001165_g | 48 | 0.2226 |
| Desulfovibrio | Bacteria;Proteobacteria;Deltaproteobacteria;Desulfovibrionales;Desulfovibrionaceae;Desulfovibrio | 41 | 0.1902 |
| PAC000748_g | Bacteria;Firmicutes;Clostridia;Clostridiales;Ruminococcaceae;PAC000748_g | 37 | 0.1716 |
| PAC001221_g | Bacteria;Firmicutes;Clostridia;Clostridiales;Dehalobacterium_f;PAC001221_g | 36 | 0.167 |
| PAC001544_g | Bacteria;Firmicutes;Clostridia;Clostridiales;Lachnospiraceae;PAC001544_g | 35 | 0.1623 |
| Caproiciproducens | Bacteria;Firmicutes;Clostridia;Clostridiales;Ruminococcaceae;Caproiciproducens | 34 | 0.1577 |
| Enterobacteriaceae_g | Bacteria;Proteobacteria;Gammaproteobacteria;Enterobacterales;Enterobacteriaceae;Enterobacteriaceae_g | 33 | 0.1531 |
| Clostridium_g24 | Bacteria;Firmicutes;Clostridia;Clostridiales;Lachnospiraceae;Clostridium_g24 | 32 | 0.1484 |
| Eubacterium_g6 | Bacteria;Firmicutes;Clostridia;Clostridiales;Lachnospiraceae;Eubacterium_g6 | 31 | 0.1438 |
| KE159797_g | Bacteria;Firmicutes;Clostridia;Clostridiales;Lachnospiraceae;KE159797_g | 28 | 0.1299 |
| LT706945_g | Bacteria;Proteobacteria;Deltaproteobacteria;Desulfovibrionales;Desulfovibrionaceae;LT706945_g | 28 | 0.1299 |
| PAC002314_g | Bacteria;Firmicutes;Erysipelotrichi;Erysipelotrichales;Erysipelotrichaceae;PAC002314_g | 26 | 0.1206 |
| Mucispirillum | Bacteria;Deferribacteres;Deferribacteres_c;Deferribacterales;Deferribacteraceae;Mucispirillum | 25 | 0.116 |
| Streptococcus | Bacteria;Firmicutes;Bacilli;Lactobacillales;Streptococcaceae;Streptococcus | 25 | 0.116 |
| PAC000195_g | Bacteria;Firmicutes;Clostridia;Clostridiales;Lachnospiraceae;PAC000195_g | 25 | 0.116 |
| Faecalibaculum | Bacteria;Firmicutes;Erysipelotrichi;Erysipelotrichales;Erysipelotrichaceae;Faecalibaculum | 23 | 0.1067 |
| Acholeplasma_g2 | Bacteria;Tenericutes;Mollicutes;Acholeplasmatales;Acholeplasmataceae;Acholeplasma_g2 | 23 | 0.1067 |
| PAC001043_g | Bacteria;Firmicutes;Clostridia;Clostridiales;Lachnospiraceae;PAC001043_g | 23 | 0.1067 |
| PAC001066_g | Bacteria;Bacteroidetes;Bacteroidia;Bacteroidales;Muribaculaceae;PAC001066_g | 22 | 0.102 |
| PAC001385_g | Bacteria;Firmicutes;Clostridia;Clostridiales;Lachnospiraceae;PAC001385_g | 22 | 0.102 |
| PAC001778_g | Bacteria;Firmicutes;Clostridia;Clostridiales;Ruminococcaceae;PAC001778_g | 21 | 0.0974 |
| PAC001144_g | Bacteria;Firmicutes;Clostridia;Clostridiales;Ruminococcaceae;PAC001144_g | 18 | 0.0835 |
| PAC001138_g | Bacteria;Firmicutes;Clostridia;Clostridiales;Lachnospiraceae;PAC001138_g | 17 | 0.0789 |
| PAC001270_g | Bacteria;Firmicutes;Clostridia;Clostridiales;Lachnospiraceae;PAC001270_g | 16 | 0.0742 |
| Lachnospira | Bacteria;Firmicutes;Clostridia;Clostridiales;Lachnospiraceae;Lachnospira | 15 | 0.0696 |
| GQ451199_g | Bacteria;Firmicutes;Clostridia;Clostridiales;Ruminococcaceae;GQ451199_g | 15 | 0.0696 |
| Bifidobacterium | Bacteria;Actinobacteria;Actinobacteria_c;Bifidobacteriales;Bifidobacteriaceae;Bifidobacterium | 14 | 0.0649 |
| Enterorhabdus | Bacteria;Actinobacteria;Coriobacteriia;Coriobacteriales;Coriobacteriaceae;Enterorhabdus | 14 | 0.0649 |
| Massilioclostridium | Bacteria;Firmicutes;Clostridia;Clostridiales;Ruminococcaceae;Massilioclostridium | 14 | 0.0649 |
| Megamonas | Bacteria;Firmicutes;Negativicutes;Selenomonadales;Selenomonadaceae;Megamonas | 14 | 0.0649 |
| Subdoligranulum | Bacteria;Firmicutes;Clostridia;Clostridiales;Ruminococcaceae;Subdoligranulum | 14 | 0.0649 |
| Eubacterium_g23 | Bacteria;Firmicutes;Clostridia;Clostridiales;Ruminococcaceae;Eubacterium_g23 | 14 | 0.0649 |
| PAC001524_g | Bacteria;Firmicutes;Clostridia;Clostridiales;Lachnospiraceae;PAC001524_g | 14 | 0.0649 |
| Clostridium | Bacteria;Firmicutes;Clostridia;Clostridiales;Clostridiaceae;Clostridium | 13 | 0.0603 |
| PAC001103_g | Bacteria;Firmicutes;Clostridia;Clostridiales;Lachnospiraceae;PAC001103_g | 13 | 0.0603 |
| FTRU_g | Bacteria;Firmicutes;Clostridia;Clostridiales;Ruminococcaceae;FTRU_g | 12 | 0.0557 |
| Bacillus | Bacteria;Firmicutes;Bacilli;Bacillales;Bacillaceae;Bacillus | 11 | 0.051 |
| Phascolarctobacterium | Bacteria;Firmicutes;Negativicutes;Acidaminococcales;Acidaminococcaceae;Phascolarctobacterium | 11 | 0.051 |
| Ruminococcus | Bacteria;Firmicutes;Clostridia;Clostridiales;Ruminococcaceae;Ruminococcus | 11 | 0.051 |
| Eubacterium_g5 | Bacteria;Firmicutes;Clostridia;Clostridiales;Lachnospiraceae;Eubacterium_g5 | 11 | 0.051 |
| PAC001201_g | Bacteria;Firmicutes;Clostridia;Clostridiales;Lachnospiraceae;PAC001201_g | 11 | 0.051 |
| Acutalibacter | Bacteria;Firmicutes;Clostridia;Clostridiales;Ruminococcaceae;Acutalibacter | 10 | 0.0464 |
| Anaerostipes | Bacteria;Firmicutes;Clostridia;Clostridiales;Lachnospiraceae;Anaerostipes | 10 | 0.0464 |
| Emergencia | Bacteria;Firmicutes;Clostridia;Clostridiales;Mogibacterium_f;Emergencia | 10 | 0.0464 |
| Roseburia | Bacteria;Firmicutes;Clostridia;Clostridiales;Lachnospiraceae;Roseburia | 10 | 0.0464 |
| PAC001573_g | Bacteria;Firmicutes;Clostridia;Clostridiales;Mogibacterium_f;PAC001573_g | 10 | 0.0464 |
| Dialister | Bacteria;Firmicutes;Negativicutes;Veillonellales;Veillonellaceae;Dialister | 9 | 0.0417 |
| Harryflintia | Bacteria;Firmicutes;Clostridia;Clostridiales;Ruminococcaceae;Harryflintia | 9 | 0.0417 |
| PAC001485_g | Bacteria;Bacteroidetes;Bacteroidia;Bacteroidales;Muribaculaceae;PAC001485_g | 9 | 0.0417 |
| Christensenella | Bacteria;Firmicutes;Clostridia;Clostridiales;Christensenellaceae;Christensenella | 8 | 0.0371 |
| Monoglobus | Bacteria;Firmicutes;Clostridia;Clostridiales;Ruminococcaceae;Monoglobus | 8 | 0.0371 |
| Veillonella | Bacteria;Firmicutes;Negativicutes;Veillonellales;Veillonellaceae;Veillonella | 8 | 0.0371 |
| GQ458233_g | Bacteria;Firmicutes;Clostridia;Clostridiales;Ruminococcaceae;GQ458233_g | 8 | 0.0371 |
| PAC001109_g | Bacteria;Tenericutes;Mollicutes;PAC001057_o;PAC001057_f;PAC001109_g | 8 | 0.0371 |
| PAC001301_g | Bacteria;Firmicutes;Clostridia;Clostridiales;Christensenellaceae;PAC001301_g | 8 | 0.0371 |
| Agathobaculum | Bacteria;Firmicutes;Clostridia;Clostridiales;Ruminococcaceae;Agathobaculum | 7 | 0.0325 |
| Catenibacterium | Bacteria;Firmicutes;Erysipelotrichi;Erysipelotrichales;Erysipelotrichaceae;Catenibacterium | 7 | 0.0325 |
| Fusicatenibacter | Bacteria;Firmicutes;Clostridia;Clostridiales;Lachnospiraceae;Fusicatenibacter | 7 | 0.0325 |
| PAC000692_g | Bacteria;Firmicutes;Clostridia;Clostridiales;Lachnospiraceae;PAC000692_g | 7 | 0.0325 |
| PAC001062_g | Bacteria;Bacteroidetes;Bacteroidia;Bacteroidales;Muribaculaceae;PAC001062_g | 7 | 0.0325 |
| Collinsella | Bacteria;Actinobacteria;Coriobacteriia;Coriobacteriales;Coriobacteriaceae;Collinsella | 6 | 0.0278 |
| PAC001528_g | Bacteria;Firmicutes;Erysipelotrichi;Erysipelotrichales;Erysipelotrichaceae;PAC001528_g | 6 | 0.0278 |
| Ruminococcus_g5 | Bacteria;Firmicutes;Clostridia;Clostridiales;Lachnospiraceae;Ruminococcus_g5 | 6 | 0.0278 |
| Enterobacter | Bacteria;Proteobacteria;Gammaproteobacteria;Enterobacterales;Enterobacteriaceae;Enterobacter | 5 | 0.0232 |
| Mitsuokella | Bacteria;Firmicutes;Negativicutes;Selenomonadales;Selenomonadaceae;Mitsuokella | 5 | 0.0232 |
| Proteus | Bacteria;Proteobacteria;Gammaproteobacteria;Enterobacterales;Morganellaceae;Proteus | 5 | 0.0232 |
| Eubacterium_g17 | Bacteria;Firmicutes;Clostridia;Clostridiales;Lachnospiraceae;Eubacterium_g17 | 5 | 0.0232 |
| KE159605_g | Bacteria;Firmicutes;Clostridia;Clostridiales;Lachnospiraceae;KE159605_g | 5 | 0.0232 |
| PAC000671_g | Bacteria;Firmicutes;Clostridia;Clostridiales;Lachnospiraceae;PAC000671_g | 5 | 0.0232 |
| PAC001149_g | Bacteria;Firmicutes;Clostridia;Clostridiales;Lachnospiraceae;PAC001149_g | 5 | 0.0232 |
| PAC001177_g | Bacteria;Firmicutes;Clostridia;Clostridiales;Lachnospiraceae;PAC001177_g | 5 | 0.0232 |
| PAC001323_g | Bacteria;Tenericutes;Mollicutes;PAC001057_o;PAC000197_f;PAC001323_g | 5 | 0.0232 |
| Acidaminococcus | Bacteria;Firmicutes;Negativicutes;Acidaminococcales;Acidaminococcaceae;Acidaminococcus | 4 | 0.0186 |
| PAC001032_g | Bacteria;Firmicutes;Clostridia;Clostridiales;Lachnospiraceae;PAC001032_g | 4 | 0.0186 |
| PAC001108_g | Bacteria;Tenericutes;Mollicutes;PAC001057_o;PAC001057_f;PAC001108_g | 4 | 0.0186 |
| PAC001308_g | Bacteria;Firmicutes;Clostridia;Clostridiales;Lachnospiraceae;PAC001308_g | 4 | 0.0186 |
| PAC001313_g | Bacteria;Firmicutes;Clostridia;Clostridiales;Ruminococcaceae;PAC001313_g | 4 | 0.0186 |
| PAC001599_g | Bacteria;Firmicutes;Clostridia;Clostridiales;Ruminococcaceae;PAC001599_g | 4 | 0.0186 |
| PAC002390_g | Bacteria;Firmicutes;Clostridia;Clostridiales;Dehalobacterium_f;PAC002390_g | 4 | 0.0186 |
| Bacteroidaceae_uc | Bacteria;Bacteroidetes;Bacteroidia;Bacteroidales;Bacteroidaceae;Bacteroidaceae_uc | 4 | 0.0186 |
| Enterobacteriaceae_uc | Bacteria;Proteobacteria;Gammaproteobacteria;Enterobacterales;Enterobacteriaceae;Enterobacteriaceae_uc | 4 | 0.0186 |
| Agathobacter | Bacteria;Firmicutes;Clostridia;Clostridiales;Lachnospiraceae;Agathobacter | 3 | 0.0139 |
| Enterococcus | Bacteria;Firmicutes;Bacilli;Lactobacillales;Enterococcaceae;Enterococcus | 3 | 0.0139 |
| Parasutterella | Bacteria;Proteobacteria;Betaproteobacteria;Burkholderiales;Sutterellaceae;Parasutterella | 3 | 0.0139 |
| Raoultella | Bacteria;Proteobacteria;Gammaproteobacteria;Enterobacterales;Enterobacteriaceae;Raoultella | 3 | 0.0139 |
| Terrisporobacter | Bacteria;Firmicutes;Clostridia;Clostridiales;Peptostreptococcaceae;Terrisporobacter | 3 | 0.0139 |
| EU461719_g | Bacteria;Firmicutes;Clostridia;Clostridiales;Lachnospiraceae;EU461719_g | 3 | 0.0139 |
| Eubacterium_g4 | Bacteria;Firmicutes;Clostridia;Clostridiales;Lachnospiraceae;Eubacterium_g4 | 3 | 0.0139 |
| PAC000672_g | Bacteria;Firmicutes;Clostridia;Clostridiales;Ruminococcaceae;PAC000672_g | 3 | 0.0139 |
| PAC001116_g | Bacteria;Firmicutes;Clostridia;Clostridiales;Lachnospiraceae;PAC001116_g | 3 | 0.0139 |
| PAC001225_g | Bacteria;Firmicutes;Clostridia;Clostridiales;Lachnospiraceae;PAC001225_g | 3 | 0.0139 |
| PAC001612_g | Bacteria;Firmicutes;Clostridia;Clostridiales;Mogibacterium_f;PAC001612_g | 3 | 0.0139 |
| Lachnospiraceae_uc | Bacteria;Firmicutes;Clostridia;Clostridiales;Lachnospiraceae;Lachnospiraceae_uc | 3 | 0.0139 |
| Alloprevotella | Bacteria;Bacteroidetes;Bacteroidia;Bacteroidales;Prevotellaceae;Alloprevotella | 2 | 0.0093 |
| Arthromitus | Bacteria;Firmicutes;Clostridia;Clostridiales;Clostridiaceae;Arthromitus | 2 | 0.0093 |
| Coprobacter | Bacteria;Bacteroidetes;Bacteroidia;Bacteroidales;Barnesiellaceae;Coprobacter | 2 | 0.0093 |
| Dorea | Bacteria;Firmicutes;Clostridia;Clostridiales;Lachnospiraceae;Dorea | 2 | 0.0093 |
| Flavobacterium | Bacteria;Bacteroidetes;Flavobacteria;Flavobacteriales;Flavobacteriaceae;Flavobacterium | 2 | 0.0093 |
| Gemella | Bacteria;Firmicutes;Bacilli;Bacillales;Gemella_f;Gemella | 2 | 0.0093 |
| Gordonia | Bacteria;Actinobacteria;Actinobacteria_c;Corynebacteriales;Nocardiaceae;Gordonia | 2 | 0.0093 |
| Longicatena | Bacteria;Firmicutes;Erysipelotrichi;Erysipelotrichales;Erysipelotrichaceae;Longicatena | 2 | 0.0093 |
| Megasphaera | Bacteria;Firmicutes;Negativicutes;Veillonellales;Veillonellaceae;Megasphaera | 2 | 0.0093 |
| Muribaculum | Bacteria;Bacteroidetes;Bacteroidia;Bacteroidales;Muribaculaceae;Muribaculum | 2 | 0.0093 |
| Paenibacillus | Bacteria;Firmicutes;Bacilli;Bacillales;Paenibacillaceae;Paenibacillus | 2 | 0.0093 |
| Romboutsia | Bacteria;Firmicutes;Clostridia;Clostridiales;Peptostreptococcaceae;Romboutsia | 2 | 0.0093 |
| Sutterella | Bacteria;Proteobacteria;Betaproteobacteria;Burkholderiales;Sutterellaceae;Sutterella | 2 | 0.0093 |
| KE993550_g | Bacteria;Firmicutes;Clostridia;Clostridiales;Ruminococcaceae;KE993550_g | 2 | 0.0093 |
| PAC000196_g | Bacteria;Firmicutes;Clostridia;Clostridiales;Lachnospiraceae;PAC000196_g | 2 | 0.0093 |
| PAC000677_g | Bacteria;Saccharibacteria_TM7;Saccharimonas_c;Saccharimonas_o;Saccharimonas_f;PAC000677_g | 2 | 0.0093 |
| PAC001100_g | Bacteria;Firmicutes;Clostridia;Clostridiales;Ruminococcaceae;PAC001100_g | 2 | 0.0093 |
| PAC001199_g | Bacteria;Firmicutes;Clostridia;Clostridiales;Lachnospiraceae;PAC001199_g | 2 | 0.0093 |
| PAC001212_g | Bacteria;Firmicutes;Clostridia;Clostridiales;Lachnospiraceae;PAC001212_g | 2 | 0.0093 |
| PAC001512_g | Bacteria;Bacteroidetes;Bacteroidia;Bacteroidales;Muribaculaceae;PAC001512_g | 2 | 0.0093 |
| PAC001609_g | Bacteria;Firmicutes;Clostridia;Clostridiales;Mogibacterium_f;PAC001609_g | 2 | 0.0093 |
| PAC002039_g | Bacteria;Firmicutes;Clostridia;Clostridiales;Lachnospiraceae;PAC002039_g | 2 | 0.0093 |
| PAC002409_g | Bacteria;Firmicutes;Clostridia;Clostridiales;Ruminococcaceae;PAC002409_g | 2 | 0.0093 |
| PAC002454_g | Bacteria;Firmicutes;Clostridia;Clostridiales;Lachnospiraceae;PAC002454_g | 2 | 0.0093 |
| Acrocarpospora | Bacteria;Actinobacteria;Actinobacteria_c;Streptosporangiales;Streptosporangiaceae;Acrocarpospora | 1 | 0.0046 |
| Aggregatibacter | Bacteria;Proteobacteria;Gammaproteobacteria;Pasteurellales;Pasteurellaceae;Aggregatibacter | 1 | 0.0046 |
| Caenibacillus | Bacteria;Firmicutes;Bacilli;Bacillales;Sporolactobacillaceae;Caenibacillus | 1 | 0.0046 |
| Caldibacillus | Bacteria;Firmicutes;Bacilli;Bacillales;Bacillaceae;Caldibacillus | 1 | 0.0046 |
| Campylobacter | Bacteria;Proteobacteria;Epsilonproteobacteria;Campylobacterales;Campylobacteraceae;Campylobacter | 1 | 0.0046 |
| Cellulomonas | Bacteria;Actinobacteria;Actinobacteria_c;Micrococcales;Cellulomonadaceae;Cellulomonas | 1 | 0.0046 |
| Clostridioides | Bacteria;Firmicutes;Clostridia;Clostridiales;Peptostreptococcaceae;Clostridioides | 1 | 0.0046 |
| Haemophilus | Bacteria;Proteobacteria;Gammaproteobacteria;Pasteurellales;Pasteurellaceae;Haemophilus | 1 | 0.0046 |
| Herbiconiux | Bacteria;Actinobacteria;Actinobacteria_c;Micrococcales;Microbacteriaceae;Herbiconiux | 1 | 0.0046 |
| Holdemanella | Bacteria;Firmicutes;Erysipelotrichi;Erysipelotrichales;Erysipelotrichaceae;Holdemanella | 1 | 0.0046 |
| Intestinibacter | Bacteria;Firmicutes;Clostridia;Clostridiales;Peptostreptococcaceae;Intestinibacter | 1 | 0.0046 |
| Lysinibacillus | Bacteria;Firmicutes;Bacilli;Bacillales;Planococcaceae;Lysinibacillus | 1 | 0.0046 |
| Mogibacterium | Bacteria;Firmicutes;Clostridia;Clostridiales;Mogibacterium_f;Mogibacterium | 1 | 0.0046 |
| Morganella | Bacteria;Proteobacteria;Gammaproteobacteria;Enterobacterales;Morganellaceae;Morganella | 1 | 0.0046 |
| Mucilaginibacter | Bacteria;Bacteroidetes;Sphingobacteriia;Sphingobacteriales;Sphingobacteriaceae;Mucilaginibacter | 1 | 0.0046 |
| Paludibacter | Bacteria;Bacteroidetes;Bacteroidia;Bacteroidales;Porphyromonadaceae;Paludibacter | 1 | 0.0046 |
| Paraprevotella | Bacteria;Bacteroidetes;Bacteroidia;Bacteroidales;Prevotellaceae;Paraprevotella | 1 | 0.0046 |
| Phocea | Bacteria;Firmicutes;Clostridia;Clostridiales;Ruminococcaceae;Phocea | 1 | 0.0046 |
| Pseudactinotalea | Bacteria;Actinobacteria;Actinobacteria_c;Micrococcales;Cellulomonadaceae;Pseudactinotalea | 1 | 0.0046 |
| Rhizobium | Bacteria;Proteobacteria;Alphaproteobacteria;Rhizobiales;Rhizobiaceae;Rhizobium | 1 | 0.0046 |
| Rothia | Bacteria;Actinobacteria;Actinobacteria_c;Micrococcales;Micrococcaceae;Rothia | 1 | 0.0046 |
| Staphylococcus | Bacteria;Firmicutes;Bacilli;Bacillales;Staphylococcaceae;Staphylococcus | 1 | 0.0046 |
| Stenotrophobacter | Bacteria;Acidobacteria;Blastocatellia;Blastocatellales;Blastocatellaceae;Stenotrophobacter | 1 | 0.0046 |
| Weissella | Bacteria;Firmicutes;Bacilli;Lactobacillales;Leuconostocaceae;Weissella | 1 | 0.0046 |
| AB021325_g | Bacteria;Actinobacteria;Actinobacteria_c;Frankiales;AB021325_f;AB021325_g | 1 | 0.0046 |
| Brucellaceae_g | Bacteria;Proteobacteria;Alphaproteobacteria;Rhizobiales;Brucellaceae;Brucellaceae_g | 1 | 0.0046 |
| CCMM_g | Bacteria;Firmicutes;Erysipelotrichi;Erysipelotrichales;Erysipelotrichaceae;CCMM_g | 1 | 0.0046 |
| Clostridium_g35 | Bacteria;Firmicutes;Clostridia;Clostridiales;Lachnospiraceae;Clostridium_g35 | 1 | 0.0046 |
| Coprococcus_g2 | Bacteria;Firmicutes;Clostridia;Clostridiales;Lachnospiraceae;Coprococcus_g2 | 1 | 0.0046 |
| DQ129389_g | Bacteria;Chloroflexi;Thermomicrobia;DQ129389_o;DQ129389_f;DQ129389_g | 1 | 0.0046 |
| GQ500701_g | Bacteria;Chloroflexi;Anaerolineae;Anaerolinaeles;Anaerolinaceae;GQ500701_g | 1 | 0.0046 |
| HM124015_g | Bacteria;Firmicutes;Clostridia;Clostridiales;Ruminococcaceae;HM124015_g | 1 | 0.0046 |
| HQ158649_g | Bacteria;Bacteroidetes;Sphingobacteriia;Saprospirales;Saprospiraceae;HQ158649_g | 1 | 0.0046 |
| LLKB_g | Bacteria;Firmicutes;Clostridia;Clostridiales;Lachnospiraceae;LLKB_g | 1 | 0.0046 |
| PAC000720_g | Bacteria;Firmicutes;Clostridia;Clostridiales;Lachnospiraceae;PAC000720_g | 1 | 0.0046 |
| PAC000740_g | Bacteria;Firmicutes;Clostridia;Clostridiales;Lachnospiraceae;PAC000740_g | 1 | 0.0046 |
| PAC001127_g | Bacteria;Bacteroidetes;Bacteroidia;Bacteroidales;Muribaculaceae;PAC001127_g | 1 | 0.0046 |
| PAC001276_g | Bacteria;Bacteroidetes;Bacteroidia;Bacteroidales;Muribaculaceae;PAC001276_g | 1 | 0.0046 |
| PAC001372_g | Bacteria;Firmicutes;Clostridia;Clostridiales;Lachnospiraceae;PAC001372_g | 1 | 0.0046 |
| PAC001386_g | Bacteria;Firmicutes;Clostridia;Clostridiales;Lachnospiraceae;PAC001386_g | 1 | 0.0046 |
| PAC001588_g | Bacteria;Firmicutes;Clostridia;Clostridiales;Lachnospiraceae;PAC001588_g | 1 | 0.0046 |
| PAC001637_g | Bacteria;Firmicutes;Clostridia;Clostridiales;Ruminococcaceae;PAC001637_g | 1 | 0.0046 |
| PAC001723_g | Bacteria;Firmicutes;Clostridia;Clostridiales;Lachnospiraceae;PAC001723_g | 1 | 0.0046 |
| PAC001765_g | Bacteria;Bacteroidetes;Bacteroidia;Bacteroidales;Muribaculaceae;PAC001765_g | 1 | 0.0046 |
| PAC002152_g | Bacteria;Firmicutes;Clostridia;Clostridiales;Lachnospiraceae;PAC002152_g | 1 | 0.0046 |
| Ruminococcus_g2 | Bacteria;Firmicutes;Clostridia;Clostridiales;Ruminococcaceae;Ruminococcus_g2 | 1 | 0.0046 |
| Ruminococcus_g4 | Bacteria;Firmicutes;Clostridia;Clostridiales;Lachnospiraceae;Ruminococcus_g4 | 1 | 0.0046 |

**Set 1_0%_S: Day 28_heme-SCP 0%_Species**

| Taxon name | Taxonomy | Count | Proportion(%) |
|---|---|---|---|
| Lactobacillus gasseri group | Bacteria;Firmicutes;Bacilli;Lactobacillales;Lactobacillaceae;Lactobacillus;Lactobacillus gasseri group | 7292 | 30.6773 |
| Bacteroides HM124113_s | Bacteria;Bacteroidetes;Bacteroidia;Bacteroidales;Bacteroidaceae;Bacteroides;HM124113_s | 3546 | 14.918 |
| Lactobacillus reuteri group | Bacteria;Firmicutes;Bacilli;Lactobacillales;Lactobacillaceae;Lactobacillus;Lactobacillus reuteri group | 2797 | 11.7669 |
| Lactobacillus murinus group | Bacteria;Firmicutes;Bacilli;Lactobacillales;Lactobacillaceae;Lactobacillus;Lactobacillus murinus group | 1281 | 5.3891 |
| Akkermansia muciniphila | Bacteria;Verrucomicrobia;Verrucomicrobiae;Verrucomicrobiales;Akkermansiaceae;Akkermansia;Akkermansia muciniphila | 1008 | 4.2406 |
| Lactococcus lactis group | Bacteria;Firmicutes;Bacilli;Lactobacillales;Streptococcaceae;Lactococcus;Lactococcus lactis group | 928 | 3.9041 |
| Bacteroides vulgatus | Bacteria;Bacteroidetes;Bacteroidia;Bacteroidales;Bacteroidaceae;Bacteroides;Bacteroides vulgatus | 592 | 2.4905 |
| PAC000198_g PAC001075_s | Bacteria;Bacteroidetes;Bacteroidia;Bacteroidales;Muribaculaceae;PAC000198_g;PAC001075_s | 515 | 2.1666 |
| Bilophila wadsworthia | Bacteria;Proteobacteria;Deltaproteobacteria;Desulfovibrionales;Desulfovibrionaceae;Bilophila;Bilophila wadsworthia | 438 | 1.8427 |
| Bacteroides_uc | Bacteria;Bacteroidetes;Bacteroidia;Bacteroidales;Bacteroidaceae;Bacteroides;Bacteroides_uc | 393 | 1.6533 |
| KE159600_g PAC001232_s | Bacteria;Firmicutes;Clostridia;Clostridiales;Lachnospiraceae;KE159600_g;PAC001232_s | 362 | 1.5229 |
| PAC000661_g PAC001095_s | Bacteria;Firmicutes;Clostridia;Clostridiales;Ruminococcaceae;PAC000661_g;PAC001095_s | 240 | 1.0097 |
| Bacteroides stercorirosoris | Bacteria;Bacteroidetes;Bacteroidia;Bacteroidales;Bacteroidaceae;Bacteroides;Bacteroides stercorirosoris | 212 | 0.8919 |
| PAC001452_g PAC001981_s group | Bacteria;Firmicutes;Clostridia;Clostridiales;Lachnospiraceae;PAC001452_g;PAC001981_s group | 200 | 0.8414 |
| PAC001112_g PAC001267_s | Bacteria;Bacteroidetes;Bacteroidia;Bacteroidales;Muribaculaceae;PAC001112_g;PAC001267_s | 199 | 0.8372 |
| PAC001141_g PAC001141_s | Bacteria;Firmicutes;Clostridia;Clostridiales;Christensenellaceae;PAC001141_g;PAC001141_s | 163 | 0.6857 |
| PAC001074_g PAC001074_s | Bacteria;Bacteroidetes;Bacteroidia;Bacteroidales;Muribaculaceae;PAC001074_g;PAC001074_s | 135 | 0.5679 |
| PAC001068_g PAC001068_s | Bacteria;Bacteroidetes;Bacteroidia;Bacteroidales;Muribaculaceae;PAC001068_g;PAC001068_s | 124 | 0.5217 |
| Bacteroides uniformis | Bacteria;Bacteroidetes;Bacteroidia;Bacteroidales;Bacteroidaceae;Bacteroides;Bacteroides uniformis | 99 | 0.4165 |
| Oscillibacter PAC001186_s group | Bacteria;Firmicutes;Clostridia;Clostridiales;Ruminococcaceae;Oscillibacter;PAC001186_s group | 97 | 0.4081 |
| PAC001516_g PAC002384_s | Bacteria;Firmicutes;Clostridia;Clostridiales;Lachnospiraceae;PAC001516_g;PAC002384_s | 93 | 0.3912 |
| PAC001066_g PAC001066_s | Bacteria;Bacteroidetes;Bacteroidia;Bacteroidales;Muribaculaceae;PAC001066_g;PAC001066_s | 90 | 0.3786 |
| PAC001118_g PAC002344_s | Bacteria;Firmicutes;Clostridia;Clostridiales;Lachnospiraceae;PAC001118_g;PAC002344_s | 85 | 0.3576 |
| PAC001165_g EU622677_s | Bacteria;Firmicutes;Clostridia;Clostridiales;Lachnospiraceae;PAC001165_g;EU622677_s | 81 | 0.3408 |
| Alistipes PAC001061_s | Bacteria;Bacteroidetes;Bacteroidia;Bacteroidales;Rikenellaceae;Alistipes;PAC001061_s | 77 | 0.3239 |
| Eubacterium_g23 PAC001121_s group | Bacteria;Firmicutes;Clostridia;Clostridiales;Ruminococcaceae;Eubacterium_g23;PAC001121_s group | 71 | 0.2987 |
| Streptococcus danieliae | Bacteria;Firmicutes;Bacilli;Lactobacillales;Streptococcaceae;Streptococcus;Streptococcus danieliae | 66 | 0.2777 |
| PAC000661_g_uc | Bacteria;Firmicutes;Clostridia;Clostridiales;Ruminococcaceae;PAC000661_g;PAC000661_g_uc | 61 | 0.2566 |
| PAC001112_g PAC002441_s | Bacteria;Bacteroidetes;Bacteroidia;Bacteroidales;Muribaculaceae;PAC001112_g;PAC002441_s | 58 | 0.244 |
| Bacteroides acidifaciens | Bacteria;Bacteroidetes;Bacteroidia;Bacteroidales;Bacteroidaceae;Bacteroides;Bacteroides acidifaciens | 55 | 0.2314 |
| Sporobacter PAC001096_s | Bacteria;Firmicutes;Clostridia;Clostridiales;Ruminococcaceae;Sporobacter;PAC001096_s | 51 | 0.2146 |
| Flintibacter butyricus group | Bacteria;Firmicutes;Clostridia;Clostridiales;Ruminococcaceae;Pseudoflavonifractor;Flintibacter butyricus group | 49 | 0.2061 |
| PAC000664_g PAC001104_s | Bacteria;Firmicutes;Clostridia;Clostridiales;Lachnospiraceae;PAC000664_g;PAC001104_s | 46 | 0.1935 |
| Lactobacillus_uc | Bacteria;Firmicutes;Bacilli;Lactobacillales;Lactobacillaceae;Lactobacillus;Lactobacillus_uc | 46 | 0.1935 |
| Parabacteroides goldsteinii | Bacteria;Bacteroidetes;Bacteroidia;Bacteroidales;Porphyromonadaceae;Parabacteroides;Parabacteroides goldsteinii | 45 | 0.1893 |
| PAC001068_g PAC001071_s | Bacteria;Bacteroidetes;Bacteroidia;Bacteroidales;Muribaculaceae;PAC001068_g;PAC001071_s | 42 | 0.1767 |
| Pseudoflavonifractor PAC001087_s | Bacteria;Firmicutes;Clostridia;Clostridiales;Ruminococcaceae;Pseudoflavonifractor;PAC001087_s | 40 | 0.1683 |
| Anaerotignum PAC001740_s | Bacteria;Firmicutes;Clostridia;Clostridiales;Lachnospiraceae;Anaerotignum;PAC001740_s | 39 | 0.1641 |
| Pseudoflavonifractor PAC002397_s | Bacteria;Firmicutes;Clostridia;Clostridiales;Ruminococcaceae;Pseudoflavonifractor;PAC002397_s | 39 | 0.1641 |
| Desulfovibrio PAC001079_s | Bacteria;Proteobacteria;Deltaproteobacteria;Desulfovibrionales;Desulfovibrionaceae;Desulfovibrio;PAC001079_s | 38 | 0.1599 |
| Anaerotruncus KE159677_s | Bacteria;Firmicutes;Clostridia;Clostridiales;Ruminococcaceae;Anaerotruncus;KE159677_s | 37 | 0.1557 |
| Oscillibacter PAC001501_s | Bacteria;Firmicutes;Clostridia;Clostridiales;Ruminococcaceae;Oscillibacter;PAC001501_s | 37 | 0.1557 |
| Oscillibacter PAC001638_s | Bacteria;Firmicutes;Clostridia;Clostridiales;Ruminococcaceae;Oscillibacter;PAC001638_s | 37 | 0.1557 |
| Odoribacter DQ815763_s | Bacteria;Bacteroidetes;Bacteroidia;Bacteroidales;Odoribacteraceae;Odoribacter;DQ815763_s | 36 | 0.1515 |
| PAC002482_g EF603735_s | Bacteria;Bacteroidetes;Bacteroidia;Bacteroidales;AC160630_f;PAC002482_g;EF603735_s | 36 | 0.1515 |
| PAC001485_g PAC001485_s | Bacteria;Bacteroidetes;Bacteroidia;Bacteroidales;Muribaculaceae;PAC001485_g;PAC001485_s | 35 | 0.1472 |
| PAC001402_g PAC001402_s | Bacteria;Firmicutes;Clostridia;Clostridiales;Ruminococcaceae;PAC001402_g;PAC001402_s | 33 | 0.1388 |
| PAC000748_g PAC001099_s | Bacteria;Firmicutes;Clostridia;Clostridiales;Ruminococcaceae;PAC000748_g;PAC001099_s | 32 | 0.1346 |
| Enterorhabdus caecimuris | Bacteria;Actinobacteria;Coriobacteriia;Coriobacteriales;Coriobacteriaceae;Enterorhabdus;Enterorhabdus caecimuris | 31 | 0.1304 |
| Paludicola PAC001508_s | Bacteria;Firmicutes;Clostridia;Clostridiales;Ruminococcaceae;Paludicola;PAC001508_s | 30 | 0.1262 |
| Pseudoflavonifractor PAC001379_s | Bacteria;Firmicutes;Clostridia;Clostridiales;Ruminococcaceae;Pseudoflavonifractor;PAC001379_s | 29 | 0.122 |
| PAC001516_g PAC001516_s | Bacteria;Firmicutes;Clostridia;Clostridiales;Lachnospiraceae;PAC001516_g;PAC001516_s | 29 | 0.122 |
| PAC000186_g PAC001064_s | Bacteria;Bacteroidetes;Bacteroidia;Bacteroidales;Muribaculaceae;PAC000186_g;PAC001064_s | 28 | 0.1178 |
| Emergencia PAC001235_s | Bacteria;Firmicutes;Clostridia;Clostridiales;Mogibacterium_f;Emergencia;PAC001235_s | 28 | 0.1178 |
| Pseudoflavonifractor PAC001549_s | Bacteria;Firmicutes;Clostridia;Clostridiales;Ruminococcaceae;Pseudoflavonifractor;PAC001549_s | 28 | 0.1178 |
| Pseudoflavonifractor PAC001370_s | Bacteria;Firmicutes;Clostridia;Clostridiales;Ruminococcaceae;Pseudoflavonifractor;PAC001370_s | 27 | 0.1136 |
| PAC001390_g PAC001390_s | Bacteria;Firmicutes;Clostridia;Clostridiales;Lachnospiraceae;PAC001390_g;PAC001390_s | 27 | 0.1136 |
| KE159571_g PAC001776_s group | Bacteria;Firmicutes;Clostridia;Clostridiales;Lachnospiraceae;KE159571_g;PAC001776_s group | 27 | 0.1136 |
| Clostridium_g21 PAC001294_s | Bacteria;Firmicutes;Clostridia;Clostridiales;Lachnospiraceae;Clostridium_g21;PAC001294_s | 26 | 0.1094 |
| PAC001068_g PAC001568_s | Bacteria;Bacteroidetes;Bacteroidia;Bacteroidales;Muribaculaceae;PAC001068_g;PAC001568_s | 26 | 0.1094 |
| LARJ_g PAC001927_s | Bacteria;Proteobacteria;Alphaproteobacteria;Rhodospirillales;Rhodospirillaceae;LARJ_g;PAC001927_s | 26 | 0.1094 |
| Pseudoflavonifractor_uc | Bacteria;Firmicutes;Clostridia;Clostridiales;Ruminococcaceae;Pseudoflavonifractor;Pseudoflavonifractor_uc | 24 | 0.101 |
| Alistipes PAC001471_s | Bacteria;Bacteroidetes;Bacteroidia;Bacteroidales;Rikenellaceae;Alistipes;PAC001471_s | 23 | 0.0968 |
| PAC000683_g_uc | Bacteria;Firmicutes;Clostridia;Clostridiales;Ruminococcaceae;PAC000683_g;PAC000683_g_uc | 23 | 0.0968 |
| KE159600_g KE159600_s | Bacteria;Firmicutes;Clostridia;Clostridiales;Lachnospiraceae;KE159600_g;KE159600_s | 22 | 0.0926 |
| Pseudoflavonifractor KI535319_s | Bacteria;Firmicutes;Clostridia;Clostridiales;Ruminococcaceae;Pseudoflavonifractor;KI535319_s | 22 | 0.0926 |
| FR888536_g PAC001268_s | Bacteria;Cyanobacteria;Vampirovibrio_c;FR888536_o;FR888536_f;FR888536_g;PAC001268_s | 22 | 0.0926 |
| PAC001385_g PAC001727_s group | Bacteria;Firmicutes;Clostridia;Clostridiales;Lachnospiraceae;PAC001385_g;PAC001727_s group | 21 | 0.0883 |
| Acetatifactor PAC001801_s | Bacteria;Firmicutes;Clostridia;Clostridiales;Lachnospiraceae;Acetatifactor;PAC001801_s | 21 | 0.0883 |
| PAC000186_g PAC001806_s | Bacteria;Bacteroidetes;Bacteroidia;Bacteroidales;Muribaculaceae;PAC000186_g;PAC001806_s | 21 | 0.0883 |
| PAC000661_g PAC001539_s | Bacteria;Firmicutes;Clostridia;Clostridiales;Ruminococcaceae;PAC000661_g;PAC001539_s | 20 | 0.0841 |
| Clostridium_g24 AF371567_s | Bacteria;Firmicutes;Clostridia;Clostridiales;Lachnospiraceae;Clostridium_g24;AF371567_s | 19 | 0.0799 |
| PAC001063_g PAC001063_s | Bacteria;Bacteroidetes;Bacteroidia;Bacteroidales;Muribaculaceae;PAC001063_g;PAC001063_s | 19 | 0.0799 |
| Staphylococcus aureus group | Bacteria;Firmicutes;Bacilli;Bacillales;Staphylococcaceae;Staphylococcus;Staphylococcus aureus group | 16 | 0.0673 |
| Oscillibacter PAC001188_s | Bacteria;Firmicutes;Clostridia;Clostridiales;Ruminococcaceae;Oscillibacter;PAC001188_s | 16 | 0.0673 |
| KE159797_g PAC001222_s | Bacteria;Firmicutes;Clostridia;Clostridiales;Lachnospiraceae;KE159797_g;PAC001222_s | 16 | 0.0673 |
| PAC001068_g PAC001751_s | Bacteria;Bacteroidetes;Bacteroidia;Bacteroidales;Muribaculaceae;PAC001068_g;PAC001751_s | 16 | 0.0673 |
| PAC001138_g PAC001775_s | Bacteria;Firmicutes;Clostridia;Clostridiales;Lachnospiraceae;PAC001138_g;PAC001775_s | 16 | 0.0673 |
| PAC001165_g PAC001384_s | Bacteria;Firmicutes;Clostridia;Clostridiales;Lachnospiraceae;PAC001165_g;PAC001384_s | 15 | 0.0631 |
| Oscillibacter PAC001584_s group | Bacteria;Firmicutes;Clostridia;Clostridiales;Ruminococcaceae;Oscillibacter;PAC001584_s group | 15 | 0.0631 |
| Lachnospiraceae_uc | Bacteria;Firmicutes;Clostridia;Clostridiales;Lachnospiraceae;Lachnospiraceae_uc | 14 | 0.0589 |
| Bacteroides ovatus group | Bacteria;Bacteroidetes;Bacteroidia;Bacteroidales;Bacteroidaceae;Bacteroides;Bacteroides ovatus group | 14 | 0.0589 |
| PAC001362_g PAC001362_s | Bacteria;Firmicutes;Erysipelotrichi;Erysipelotrichales;Erysipelotrichaceae;PAC001362_g;PAC001362_s | 14 | 0.0589 |
| PAC001400_g PAC001400_s | Bacteria;Firmicutes;Clostridia;Clostridiales;Peptococcaceae;PAC001400_g;PAC001400_s | 14 | 0.0589 |
| PAC002314_g PAC002314_s | Bacteria;Firmicutes;Erysipelotrichi;Erysipelotrichales;Erysipelotrichaceae;PAC002314_g;PAC002314_s | 14 | 0.0589 |
| KE159797_g_uc | Bacteria;Firmicutes;Clostridia;Clostridiales;Lachnospiraceae;KE159797_g;KE159797_g_uc | 14 | 0.0589 |
| PAC001485_g_uc | Bacteria;Bacteroidetes;Bacteroidia;Bacteroidales;Muribaculaceae;PAC001485_g;PAC001485_g_uc | 14 | 0.0589 |
| Pseudoflavonifractor PAC001547_s | Bacteria;Firmicutes;Clostridia;Clostridiales;Ruminococcaceae;Pseudoflavonifractor;PAC001547_s | 13 | 0.0547 |
| Pseudoflavonifractor PAC001550_s | Bacteria;Firmicutes;Clostridia;Clostridiales;Ruminococcaceae;Pseudoflavonifractor;PAC001550_s | 13 | 0.0547 |
| Pseudoflavonifractor PAC001574_s group | Bacteria;Firmicutes;Clostridia;Clostridiales;Ruminococcaceae;Pseudoflavonifractor;PAC001574_s group | 13 | 0.0547 |
| PAC001516_g PAC001741_s | Bacteria;Firmicutes;Clostridia;Clostridiales;Lachnospiraceae;PAC001516_g;PAC001741_s | 13 | 0.0547 |
| PAC001092_g_uc | Bacteria;Firmicutes;Clostridia;Clostridiales;Lachnospiraceae;PAC001092_g;PAC001092_g_uc | 13 | 0.0547 |
| Bacteroides faecichinchillae | Bacteria;Bacteroidetes;Bacteroidia;Bacteroidales;Bacteroidaceae;Bacteroides;Bacteroides faecichinchillae | 12 | 0.0505 |
| Clostridium_g21 EU506235_s | Bacteria;Firmicutes;Clostridia;Clostridiales;Lachnospiraceae;Clostridium_g21;EU506235_s | 12 | 0.0505 |
| PAC001068_g PAC001069_s | Bacteria;Bacteroidetes;Bacteroidia;Bacteroidales;Muribaculaceae;PAC001068_g;PAC001069_s | 12 | 0.0505 |
| PAC001221_g PAC001221_s | Bacteria;Firmicutes;Clostridia;Clostridiales;Dehalobacterium_f;PAC001221_g;PAC001221_s | 12 | 0.0505 |
| Acetatifactor PAC001373_s | Bacteria;Firmicutes;Clostridia;Clostridiales;Lachnospiraceae;Acetatifactor;PAC001373_s | 12 | 0.0505 |
| Frisingicoccus PAC001481_s | Bacteria;Firmicutes;Clostridia;Clostridiales;Lachnospiraceae;Frisingicoccus;PAC001481_s | 12 | 0.0505 |
| Acetatifactor PAC002332_s | Bacteria;Firmicutes;Clostridia;Clostridiales;Lachnospiraceae;Acetatifactor;PAC002332_s | 12 | 0.0505 |
| Escherichia coli group | Bacteria;Proteobacteria;Gammaproteobacteria;Enterobacterales;Enterobacteriaceae;Escherichia;Escherichia coli group | 11 | 0.0463 |
| PAC000677_g PAC001192_s group | Bacteria;Saccharibacteria_TM7;Saccharimonas_c;Saccharimonas_o;Saccharimonas_f;PAC000677_g;PAC001192_s group | 11 | 0.0463 |
| PAC001165_g PAC001796_s | Bacteria;Firmicutes;Clostridia;Clostridiales;Lachnospiraceae;PAC001165_g;PAC001796_s | 11 | 0.0463 |
| KE159571_g PAC002035_s | Bacteria;Firmicutes;Clostridia;Clostridiales;Lachnospiraceae;KE159571_g;PAC002035_s | 11 | 0.0463 |
| Streptococcus acidominimus group | Bacteria;Firmicutes;Bacilli;Lactobacillales;Streptococcaceae;Streptococcus;Streptococcus acidominimus group | 10 | 0.0421 |
| Eubacterium_g6 JQ083736_s | Bacteria;Firmicutes;Clostridia;Clostridiales;Lachnospiraceae;Eubacterium_g6;JQ083736_s | 10 | 0.0421 |
| PAC000661_g PAC001182_s | Bacteria;Firmicutes;Clostridia;Clostridiales;Ruminococcaceae;PAC000661_g;PAC001182_s | 10 | 0.0421 |
| PAC001201_g PAC001201_s group | Bacteria;Firmicutes;Clostridia;Clostridiales;Lachnospiraceae;PAC001201_g;PAC001201_s group | 10 | 0.0421 |
| PAC001103_g PAC001361_s | Bacteria;Firmicutes;Clostridia;Clostridiales;Lachnospiraceae;PAC001103_g;PAC001361_s | 10 | 0.0421 |
| PAC000671_g PAC001473_s | Bacteria;Firmicutes;Clostridia;Clostridiales;Lachnospiraceae;PAC000671_g;PAC001473_s | 10 | 0.0421 |
| KE159571_g KE159641_s | Bacteria;Firmicutes;Clostridia;Clostridiales;Lachnospiraceae;KE159571_g;KE159641_s | 9 | 0.0379 |
| Clostridium_g21 KE159781_s | Bacteria;Firmicutes;Clostridia;Clostridiales;Lachnospiraceae;Clostridium_g21;KE159781_s | 9 | 0.0379 |
| PAC000186_g PAC001065_s | Bacteria;Bacteroidetes;Bacteroidia;Bacteroidales;Muribaculaceae;PAC000186_g;PAC001065_s | 9 | 0.0379 |
| PAC001524_g PAC001524_s | Bacteria;Firmicutes;Clostridia;Clostridiales;Lachnospiraceae;PAC001524_g;PAC001524_s | 9 | 0.0379 |
| Oscillibacter PAC001526_s | Bacteria;Firmicutes;Clostridia;Clostridiales;Ruminococcaceae;Oscillibacter;PAC001526_s | 9 | 0.0379 |
| PAC001738_g PAC001738_s | Bacteria;Firmicutes;Clostridia;Clostridiales;Lachnospiraceae;PAC001738_g;PAC001738_s | 9 | 0.0379 |
| Pseudoflavonifractor PAC001786_s | Bacteria;Firmicutes;Clostridia;Clostridiales;Ruminococcaceae;Pseudoflavonifractor;PAC001786_s | 9 | 0.0379 |
| Acetatifactor PAC001773_s | Bacteria;Firmicutes;Clostridia;Clostridiales;Lachnospiraceae;Acetatifactor;PAC001773_s | 8 | 0.0337 |
| Paludicola PAC001784_s | Bacteria;Firmicutes;Clostridia;Clostridiales;Ruminococcaceae;Paludicola;PAC001784_s | 8 | 0.0337 |
| PAC001066_g_uc | Bacteria;Bacteroidetes;Bacteroidia;Bacteroidales;Muribaculaceae;PAC001066_g;PAC001066_g_uc | 8 | 0.0337 |
| Enterococcus faecalis | Bacteria;Firmicutes;Bacilli;Lactobacillales;Enterococcaceae;Enterococcus;Enterococcus faecalis | 7 | 0.0294 |
| Helicobacter japonicus | Bacteria;Proteobacteria;Epsilonproteobacteria;Campylobacterales;Helicobacteraceae;Helicobacter;Helicobacter japonicus | 7 | 0.0294 |
| Eubacterium_g6 PAC001586_s | Bacteria;Firmicutes;Clostridia;Clostridiales;Lachnospiraceae;Eubacterium_g6;PAC001586_s | 7 | 0.0294 |
| PAC001402_g PAC001764_s | Bacteria;Firmicutes;Clostridia;Clostridiales;Ruminococcaceae;PAC001402_g;PAC001764_s | 7 | 0.0294 |
| PAC002375_g PAC002375_s | Bacteria;Firmicutes;Clostridia;Clostridiales;Lachnospiraceae;PAC002375_g;PAC002375_s | 7 | 0.0294 |
| Clostridium_g21 PAC002492_s | Bacteria;Firmicutes;Clostridia;Clostridiales;Lachnospiraceae;Clostridium_g21;PAC002492_s | 7 | 0.0294 |
| Bacteroides oleiciplenus | Bacteria;Bacteroidetes;Bacteroidia;Bacteroidales;Bacteroidaceae;Bacteroides;Bacteroides oleiciplenus | 6 | 0.0252 |
| Blautia hansenii group | Bacteria;Firmicutes;Clostridia;Clostridiales;Lachnospiraceae;Blautia;Blautia hansenii group | 6 | 0.0252 |
| Clostridium cocleatum | Bacteria;Firmicutes;Erysipelotrichi;Erysipelotrichales;Erysipelotrichaceae;Clostridium_g6;Clostridium cocleatum | 6 | 0.0252 |
| LARJ_g PAC000185_s | Bacteria;Proteobacteria;Alphaproteobacteria;Rhodospirillales;Rhodospirillaceae;LARJ_g;PAC000185_s | 6 | 0.0252 |
| Oscillibacter PAC001156_s | Bacteria;Firmicutes;Clostridia;Clostridiales;Ruminococcaceae;Oscillibacter;PAC001156_s | 6 | 0.0252 |
| PAC001323_g PAC001323_s | Bacteria;Tenericutes;Mollicutes;PAC001057_o;PAC000197_f;PAC001323_g;PAC001323_s | 6 | 0.0252 |
| KE159571_g PAC001555_s | Bacteria;Firmicutes;Clostridia;Clostridiales;Lachnospiraceae;KE159571_g;PAC001555_s | 6 | 0.0252 |
| PAC001609_g PAC001650_s | Bacteria;Firmicutes;Clostridia;Clostridiales;Mogibacterium_f;PAC001609_g;PAC001650_s | 6 | 0.0252 |
| PAC001068_g PAC001736_s | Bacteria;Bacteroidetes;Bacteroidia;Bacteroidales;Muribaculaceae;PAC001068_g;PAC001736_s | 6 | 0.0252 |
| LT706945_g PAC001747_s | Bacteria;Proteobacteria;Deltaproteobacteria;Desulfovibrionales;Desulfovibrionaceae;LT706945_g;PAC001747_s | 6 | 0.0252 |
| PAC001573_g PAC001808_s | Bacteria;Firmicutes;Clostridia;Clostridiales;Mogibacterium_f;PAC001573_g;PAC001808_s | 6 | 0.0252 |
| Oscillibacter PAC002372_s | Bacteria;Firmicutes;Clostridia;Clostridiales;Ruminococcaceae;Oscillibacter;PAC002372_s | 6 | 0.0252 |
| PAC001270_g PAC002420_s | Bacteria;Firmicutes;Clostridia;Clostridiales;Lachnospiraceae;PAC001270_g;PAC002420_s | 6 | 0.0252 |
| PAC001692_g PAC002446_s | Bacteria;Bacteroidetes;Bacteroidia;Bacteroidales;Muribaculaceae;PAC001692_g;PAC002446_s | 6 | 0.0252 |
| PAC001043_g PAC002511_s group | Bacteria;Firmicutes;Clostridia;Clostridiales;Lachnospiraceae;PAC001043_g;PAC002511_s group | 6 | 0.0252 |
| Clostridium_g21 PAC002512_s group | Bacteria;Firmicutes;Clostridia;Clostridiales;Lachnospiraceae;Clostridium_g21;PAC002512_s group | 6 | 0.0252 |
| PAC000197_g_uc | Bacteria;Tenericutes;Mollicutes;PAC001057_o;PAC000197_f;PAC000197_g;PAC000197_g_uc | 6 | 0.0252 |
| PAC001103_g_uc | Bacteria;Firmicutes;Clostridia;Clostridiales;Lachnospiraceae;PAC001103_g;PAC001103_g_uc | 6 | 0.0252 |
| Bacteroides intestinalis | Bacteria;Bacteroidetes;Bacteroidia;Bacteroidales;Bacteroidaceae;Bacteroides;Bacteroides intestinalis | 5 | 0.021 |
| Proteus mirabilis | Bacteria;Proteobacteria;Gammaproteobacteria;Enterobacterales;Morganellaceae;Proteus;Proteus mirabilis | 5 | 0.021 |
| PAC000661_g EU842956_s | Bacteria;Firmicutes;Clostridia;Clostridiales;Ruminococcaceae;PAC000661_g;EU842956_s | 5 | 0.021 |
| Pseudoflavonifractor FJ879979_s | Bacteria;Firmicutes;Clostridia;Clostridiales;Ruminococcaceae;Pseudoflavonifractor;FJ879979_s | 5 | 0.021 |
| Oscillibacter KE159714_s group | Bacteria;Firmicutes;Clostridia;Clostridiales;Ruminococcaceae;Oscillibacter;KE159714_s group | 5 | 0.021 |
| Clostridium_g21 PAC001085_s | Bacteria;Firmicutes;Clostridia;Clostridiales;Lachnospiraceae;Clostridium_g21;PAC001085_s | 5 | 0.021 |
| PAC000186_g PAC001128_s | Bacteria;Bacteroidetes;Bacteroidia;Bacteroidales;Muribaculaceae;PAC000186_g;PAC001128_s | 5 | 0.021 |
| PAC001313_g PAC001313_s | Bacteria;Firmicutes;Clostridia;Clostridiales;Ruminococcaceae;PAC001313_g;PAC001313_s | 5 | 0.021 |
| PAC000748_g PAC001496_s | Bacteria;Firmicutes;Clostridia;Clostridiales;Ruminococcaceae;PAC000748_g;PAC001496_s | 5 | 0.021 |
| Coprococcus_g2 PAC001722_s | Bacteria;Firmicutes;Clostridia;Clostridiales;Lachnospiraceae;Coprococcus_g2;PAC001722_s | 5 | 0.021 |
| PAC001092_g PAC001742_s | Bacteria;Firmicutes;Clostridia;Clostridiales;Lachnospiraceae;PAC001092_g;PAC001742_s | 5 | 0.021 |
| Arthromitus AP012202_s group | Bacteria;Firmicutes;Clostridia;Clostridiales;Clostridiaceae;Arthromitus;AP012202_s group | 4 | 0.0168 |
| KE159605_g KE159605_s | Bacteria;Firmicutes;Clostridia;Clostridiales;Lachnospiraceae;KE159605_g;KE159605_s | 4 | 0.0168 |
| Pseudoflavonifractor PAC001318_s | Bacteria;Firmicutes;Clostridia;Clostridiales;Ruminococcaceae;Pseudoflavonifractor;PAC001318_s | 4 | 0.0168 |
| FTRU_g PAC001367_s | Bacteria;Firmicutes;Clostridia;Clostridiales;Ruminococcaceae;FTRU_g;PAC001367_s | 4 | 0.0168 |
| PAC000748_g PAC001489_s | Bacteria;Firmicutes;Clostridia;Clostridiales;Ruminococcaceae;PAC000748_g;PAC001489_s | 4 | 0.0168 |
| Oscillibacter PAC001502_s | Bacteria;Firmicutes;Clostridia;Clostridiales;Ruminococcaceae;Oscillibacter;PAC001502_s | 4 | 0.0168 |
| PAC001512_g PAC001512_s | Bacteria;Bacteroidetes;Bacteroidia;Bacteroidales;Muribaculaceae;PAC001512_g;PAC001512_s | 4 | 0.0168 |
| Anaerotignum PAC001540_s | Bacteria;Firmicutes;Clostridia;Clostridiales;Lachnospiraceae;Anaerotignum;PAC001540_s | 4 | 0.0168 |
| LLKB_g PAC001694_s | Bacteria;Firmicutes;Clostridia;Clostridiales;Lachnospiraceae;LLKB_g;PAC001694_s | 4 | 0.0168 |
| KE159571_g PAC001705_s | Bacteria;Firmicutes;Clostridia;Clostridiales;Lachnospiraceae;KE159571_g;PAC001705_s | 4 | 0.0168 |
| PAC001402_g PAC001748_s | Bacteria;Firmicutes;Clostridia;Clostridiales;Ruminococcaceae;PAC001402_g;PAC001748_s | 4 | 0.0168 |
| KE159571_g PAC002009_s group | Bacteria;Firmicutes;Clostridia;Clostridiales;Lachnospiraceae;KE159571_g;PAC002009_s group | 4 | 0.0168 |
| PAC001228_g PAC002027_s | Bacteria;Firmicutes;Clostridia;Clostridiales;Lachnospiraceae;PAC001228_g;PAC002027_s | 4 | 0.0168 |
| PAC002039_g PAC002039_s | Bacteria;Firmicutes;Clostridia;Clostridiales;Lachnospiraceae;PAC002039_g;PAC002039_s | 4 | 0.0168 |
| PAC001127_g PAC002399_s | Bacteria;Bacteroidetes;Bacteroidia;Bacteroidales;Muribaculaceae;PAC001127_g;PAC002399_s | 4 | 0.0168 |
| LARJ_g_uc | Bacteria;Proteobacteria;Alphaproteobacteria;Rhodospirillales;Rhodospirillaceae;LARJ_g;LARJ_g_uc | 4 | 0.0168 |
| Bacteroides thetaiotaomicron | Bacteria;Bacteroidetes;Bacteroidia;Bacteroidales;Bacteroidaceae;Bacteroides;Bacteroides thetaiotaomicron | 3 | 0.0126 |
| Clostridioides difficile group | Bacteria;Firmicutes;Clostridia;Clostridiales;Peptostreptococcaceae;Clostridioides;Clostridioides difficile group | 3 | 0.0126 |
| Clostridium innocuum group | Bacteria;Firmicutes;Erysipelotrichi;Erysipelotrichales;Erysipelotrichaceae;Longicatena;Clostridium innocuum group | 3 | 0.0126 |
| Helicobacter mesocricetorum group | Bacteria;Proteobacteria;Epsilonproteobacteria;Campylobacterales;Helicobacteraceae;Helicobacter;Helicobacter mesocricetorum group | 3 | 0.0126 |
| Mucispirillum schaedleri | Bacteria;Deferribacteres;Deferribacteres_c;Deferribacterales;Deferribacteraceae;Mucispirillum;Mucispirillum schaedleri | 3 | 0.0126 |
| Bacteroides DQ805799_s | Bacteria;Bacteroidetes;Bacteroidia;Bacteroidales;Bacteroidaceae;Bacteroides;DQ805799_s | 3 | 0.0126 |
| Muribaculum DQ815542_s | Bacteria;Bacteroidetes;Bacteroidia;Bacteroidales;Muribaculaceae;Muribaculum;DQ815542_s | 3 | 0.0126 |
| PAC001472_g EU510580_s | Bacteria;Bacteroidetes;Bacteroidia;Bacteroidales;Muribaculaceae;PAC001472_g;EU510580_s | 3 | 0.0126 |
| Caproiciproducens EU622658_s | Bacteria;Firmicutes;Clostridia;Clostridiales;Ruminococcaceae;Caproiciproducens;EU622658_s | 3 | 0.0126 |
| KE159797_g KE159797_s | Bacteria;Firmicutes;Clostridia;Clostridiales;Lachnospiraceae;KE159797_g;KE159797_s | 3 | 0.0126 |
| PAC000664_g PAC001226_s | Bacteria;Firmicutes;Clostridia;Clostridiales;Lachnospiraceae;PAC000664_g;PAC001226_s | 3 | 0.0126 |
| PAC001360_g PAC001371_s | Bacteria;Firmicutes;Clostridia;Clostridiales;Christensenellaceae;PAC001360_g;PAC001371_s | 3 | 0.0126 |
| PAC000664_g PAC001515_s | Bacteria;Firmicutes;Clostridia;Clostridiales;Lachnospiraceae;PAC000664_g;PAC001515_s | 3 | 0.0126 |
| PAC001528_g PAC001528_s | Bacteria;Firmicutes;Erysipelotrichi;Erysipelotrichales;Erysipelotrichaceae;PAC001528_g;PAC001528_s | 3 | 0.0126 |
| PAC001437_g PAC001533_s | Bacteria;Firmicutes;Clostridia;Clostridiales;Christensenellaceae;PAC001437_g;PAC001533_s | 3 | 0.0126 |
| Pseudoflavonifractor PAC001543_s | Bacteria;Firmicutes;Clostridia;Clostridiales;Ruminococcaceae;Pseudoflavonifractor;PAC001543_s | 3 | 0.0126 |
| PAC001402_g PAC001546_s | Bacteria;Firmicutes;Clostridia;Clostridiales;Ruminococcaceae;PAC001402_g;PAC001546_s | 3 | 0.0126 |
| PAC001402_g PAC001561_s | Bacteria;Firmicutes;Clostridia;Clostridiales;Ruminococcaceae;PAC001402_g;PAC001561_s | 3 | 0.0126 |
| PAC001144_g PAC001593_s | Bacteria;Firmicutes;Clostridia;Clostridiales;Ruminococcaceae;PAC001144_g;PAC001593_s | 3 | 0.0126 |
| Sporobacter PAC001659_s | Bacteria;Firmicutes;Clostridia;Clostridiales;Ruminococcaceae;Sporobacter;PAC001659_s | 3 | 0.0126 |
| PAC001092_g PAC001668_s | Bacteria;Firmicutes;Clostridia;Clostridiales;Lachnospiraceae;PAC001092_g;PAC001668_s | 3 | 0.0126 |
| PAC001092_g PAC001710_s | Bacteria;Firmicutes;Clostridia;Clostridiales;Lachnospiraceae;PAC001092_g;PAC001710_s | 3 | 0.0126 |
| Frisingicoccus PAC001744_s group | Bacteria;Firmicutes;Clostridia;Clostridiales;Lachnospiraceae;Frisingicoccus;PAC001744_s group | 3 | 0.0126 |
| PAC001765_g PAC001765_s | Bacteria;Bacteroidetes;Bacteroidia;Bacteroidales;Muribaculaceae;PAC001765_g;PAC001765_s | 3 | 0.0126 |
| PAC001108_g PAC002140_s | Bacteria;Tenericutes;Mollicutes;PAC001057_o;PAC001057_f;PAC001108_g;PAC002140_s | 3 | 0.0126 |
| Acutalibacter PAC002354_s | Bacteria;Firmicutes;Clostridia;Clostridiales;Ruminococcaceae;Acutalibacter;PAC002354_s | 3 | 0.0126 |
| PAC001199_g PAC002391_s | Bacteria;Firmicutes;Clostridia;Clostridiales;Lachnospiraceae;PAC001199_g;PAC002391_s | 3 | 0.0126 |
| Clostridium_g21_uc | Bacteria;Firmicutes;Clostridia;Clostridiales;Lachnospiraceae;Clostridium_g21;Clostridium_g21_uc | 3 | 0.0126 |
| PAC001068_g_uc | Bacteria;Bacteroidetes;Bacteroidia;Bacteroidales;Muribaculaceae;PAC001068_g;PAC001068_g_uc | 3 | 0.0126 |
| Ruminococcaceae_uc | Bacteria;Firmicutes;Clostridia;Clostridiales;Ruminococcaceae;Ruminococcaceae_uc | 2 | 0.0084 |
| Alistipes ihumii | Bacteria;Bacteroidetes;Bacteroidia;Bacteroidales;Rikenellaceae;Alistipes;Alistipes ihumii | 2 | 0.0084 |
| Corynebacterium glutamicum | Bacteria;Actinobacteria;Actinobacteria_c;Corynebacteriales;Corynebacteriaceae;Corynebacterium;Corynebacterium glutamicum | 2 | 0.0084 |
| Enterorhabdus muris | Bacteria;Actinobacteria;Coriobacteriia;Coriobacteriales;Coriobacteriaceae;Enterorhabdus;Enterorhabdus muris | 2 | 0.0084 |
| Romboutsia timonensis | Bacteria;Firmicutes;Clostridia;Clostridiales;Peptostreptococcaceae;Romboutsia;Romboutsia timonensis | 2 | 0.0084 |
| PAC002400_g EF096814_s | Bacteria;Bacteroidetes;Bacteroidia;Bacteroidales;Muribaculaceae;PAC002400_g;EF096814_s | 2 | 0.0084 |
| PAC001219_g EF603174_s | Bacteria;Firmicutes;Clostridia;Clostridiales;Christensenellaceae;PAC001219_g;EF603174_s | 2 | 0.0084 |
| Christensenella EU508399_s | Bacteria;Firmicutes;Clostridia;Clostridiales;Christensenellaceae;Christensenella;EU508399_s | 2 | 0.0084 |
| Enterobacter KQ235774_s | Bacteria;Proteobacteria;Gammaproteobacteria;Enterobacterales;Enterobacteriaceae;Enterobacter;KQ235774_s | 2 | 0.0084 |
| PAC000664_g PAC001083_s | Bacteria;Firmicutes;Clostridia;Clostridiales;Lachnospiraceae;PAC000664_g;PAC001083_s | 2 | 0.0084 |
| Pseudoflavonifractor PAC001131_s | Bacteria;Firmicutes;Clostridia;Clostridiales;Ruminococcaceae;Pseudoflavonifractor;PAC001131_s | 2 | 0.0084 |
| PAC001144_g PAC001144_s | Bacteria;Firmicutes;Clostridia;Clostridiales;Ruminococcaceae;PAC001144_g;PAC001144_s | 2 | 0.0084 |
| PAC001287_g PAC001287_s | Bacteria;Firmicutes;Clostridia;Clostridiales;Lachnospiraceae;PAC001287_g;PAC001287_s | 2 | 0.0084 |
| PAC000661_g PAC001329_s | Bacteria;Firmicutes;Clostridia;Clostridiales;Ruminococcaceae;PAC000661_g;PAC001329_s | 2 | 0.0084 |
| Oscillibacter PAC001368_s | Bacteria;Firmicutes;Clostridia;Clostridiales;Ruminococcaceae;Oscillibacter;PAC001368_s | 2 | 0.0084 |
| Oscillibacter PAC001369_s | Bacteria;Firmicutes;Clostridia;Clostridiales;Ruminococcaceae;Oscillibacter;PAC001369_s | 2 | 0.0084 |
| PAC001372_g PAC001372_s | Bacteria;Firmicutes;Clostridia;Clostridiales;Lachnospiraceae;PAC001372_g;PAC001372_s | 2 | 0.0084 |
| PAC000748_g PAC001412_s | Bacteria;Firmicutes;Clostridia;Clostridiales;Ruminococcaceae;PAC000748_g;PAC001412_s | 2 | 0.0084 |
| Paludicola PAC001507_s | Bacteria;Firmicutes;Clostridia;Clostridiales;Ruminococcaceae;Paludicola;PAC001507_s | 2 | 0.0084 |
| PAC001138_g PAC001518_s | Bacteria;Firmicutes;Clostridia;Clostridiales;Lachnospiraceae;PAC001138_g;PAC001518_s | 2 | 0.0084 |
| Pseudoflavonifractor PAC001542_s | Bacteria;Firmicutes;Clostridia;Clostridiales;Ruminococcaceae;Pseudoflavonifractor;PAC001542_s | 2 | 0.0084 |
| PAC001612_g PAC001612_s | Bacteria;Firmicutes;Clostridia;Clostridiales;Mogibacterium_f;PAC001612_g;PAC001612_s | 2 | 0.0084 |
| PAC001144_g PAC001626_s | Bacteria;Firmicutes;Clostridia;Clostridiales;Ruminococcaceae;PAC001144_g;PAC001626_s | 2 | 0.0084 |
| PAC001651_g PAC001651_s | Bacteria;Firmicutes;Clostridia;Clostridiales;Ruminococcaceae;PAC001651_g;PAC001651_s | 2 | 0.0084 |
| PAC000692_g PAC001666_s | Bacteria;Firmicutes;Clostridia;Clostridiales;Lachnospiraceae;PAC000692_g;PAC001666_s | 2 | 0.0084 |
| Harryflintia PAC001713_s | Bacteria;Firmicutes;Clostridia;Clostridiales;Ruminococcaceae;Harryflintia;PAC001713_s | 2 | 0.0084 |
| Marvinbryantia PAC001753_s | Bacteria;Firmicutes;Clostridia;Clostridiales;Lachnospiraceae;Marvinbryantia;PAC001753_s | 2 | 0.0084 |
| PAC001092_g PAC001774_s | Bacteria;Firmicutes;Clostridia;Clostridiales;Lachnospiraceae;PAC001092_g;PAC001774_s | 2 | 0.0084 |
| Eubacterium_g6 PAC002177_s | Bacteria;Firmicutes;Clostridia;Clostridiales;Lachnospiraceae;Eubacterium_g6;PAC002177_s | 2 | 0.0084 |
| KE159600_g PAC002179_s | Bacteria;Firmicutes;Clostridia;Clostridiales;Lachnospiraceae;KE159600_g;PAC002179_s | 2 | 0.0084 |
| PAC002390_g PAC002390_s | Bacteria;Firmicutes;Clostridia;Clostridiales;Dehalobacterium_f;PAC002390_g;PAC002390_s | 2 | 0.0084 |
| Acetatifactor_uc | Bacteria;Firmicutes;Clostridia;Clostridiales;Lachnospiraceae;Acetatifactor;Acetatifactor_uc | 2 | 0.0084 |
| Clostridium_g24_uc | Bacteria;Firmicutes;Clostridia;Clostridiales;Lachnospiraceae;Clostridium_g24;Clostridium_g24_uc | 2 | 0.0084 |
| PAC000196_g_uc | Bacteria;Firmicutes;Clostridia;Clostridiales;Lachnospiraceae;PAC000196_g;PAC000196_g_uc | 2 | 0.0084 |
| Rhizobiales_uc | Bacteria;Proteobacteria;Alphaproteobacteria;Rhizobiales;Rhizobiales_uc | 1 | 0.0042 |
| Acetatifactor muris | Bacteria;Firmicutes;Clostridia;Clostridiales;Lachnospiraceae;Acetatifactor;Acetatifactor muris | 1 | 0.0042 |
| Acinetobacter pittii group | Bacteria;Proteobacteria;Gammaproteobacteria;Pseudomonadales;Moraxellaceae;Acinetobacter;Acinetobacter pittii group | 1 | 0.0042 |
| Acutalibacter muris | Bacteria;Firmicutes;Clostridia;Clostridiales;Ruminococcaceae;Acutalibacter;Acutalibacter muris | 1 | 0.0042 |
| Alistipes finegoldii | Bacteria;Bacteroidetes;Bacteroidia;Bacteroidales;Rikenellaceae;Alistipes;Alistipes finegoldii | 1 | 0.0042 |
| Alistipes shahii | Bacteria;Bacteroidetes;Bacteroidia;Bacteroidales;Rikenellaceae;Alistipes;Alistipes shahii | 1 | 0.0042 |
| Anaerostipes hadrus group | Bacteria;Firmicutes;Clostridia;Clostridiales;Lachnospiraceae;Anaerostipes;Anaerostipes hadrus group | 1 | 0.0042 |
| Anaerotruncus colihominis | Bacteria;Firmicutes;Clostridia;Clostridiales;Ruminococcaceae;Anaerotruncus;Anaerotruncus colihominis | 1 | 0.0042 |
| Arachidicoccus rhizosphaerae | Bacteria;Bacteroidetes;Sphingobacteriia;Sphingobacteriales;Chitinophagaceae;Arachidicoccus;Arachidicoccus rhizosphaerae | 1 | 0.0042 |
| Bacteroides caecimuris | Bacteria;Bacteroidetes;Bacteroidia;Bacteroidales;Bacteroidaceae;Bacteroides;Bacteroides caecimuris | 1 | 0.0042 |
| Bacteroides plebeius | Bacteria;Bacteroidetes;Bacteroidia;Bacteroidales;Bacteroidaceae;Bacteroides;Bacteroides plebeius | 1 | 0.0042 |
| Catenulispora acidiphila group | Bacteria;Actinobacteria;Actinobacteria_c;Catenulisporales;Catenulisporaceae;Catenulispora;Catenulispora acidiphila group | 1 | 0.0042 |
| Clostridium symbiosum | Bacteria;Firmicutes;Clostridia;Clostridiales;Lachnospiraceae;Clostridium_g35;Clostridium symbiosum | 1 | 0.0042 |
| Enterococcus faecium group | Bacteria;Firmicutes;Bacilli;Lactobacillales;Enterococcaceae;Enterococcus;Enterococcus faecium group | 1 | 0.0042 |
| Enterorhabdus mucosicola | Bacteria;Actinobacteria;Coriobacteriia;Coriobacteriales;Coriobacteriaceae;Enterorhabdus;Enterorhabdus mucosicola | 1 | 0.0042 |
| Eubacterium hallii | Bacteria;Firmicutes;Clostridia;Clostridiales;Lachnospiraceae;Eubacterium_g5;Eubacterium hallii | 1 | 0.0042 |
| Faecalibacterium prausnitzii group | Bacteria;Firmicutes;Clostridia;Clostridiales;Ruminococcaceae;Faecalibacterium;Faecalibacterium prausnitzii group | 1 | 0.0042 |
| Geobacillus thermodenitrificans group | Bacteria;Firmicutes;Bacilli;Bacillales;Bacillaceae;Geobacillus;Geobacillus thermodenitrificans group | 1 | 0.0042 |
| Haemophilus parainfluenzae group | Bacteria;Proteobacteria;Gammaproteobacteria;Pasteurellales;Pasteurellaceae;Haemophilus;Haemophilus parainfluenzae group | 1 | 0.0042 |
| Helicobacter hepaticus | Bacteria;Proteobacteria;Epsilonproteobacteria;Campylobacterales;Helicobacteraceae;Helicobacter;Helicobacter hepaticus | 1 | 0.0042 |
| Holdemanella biformis | Bacteria;Firmicutes;Erysipelotrichi;Erysipelotrichales;Erysipelotrichaceae;Holdemanella;Holdemanella biformis | 1 | 0.0042 |
| Lactobacillus intestinalis | Bacteria;Firmicutes;Bacilli;Lactobacillales;Lactobacillaceae;Lactobacillus;Lactobacillus intestinalis | 1 | 0.0042 |
| Lactobacillus satsumensis | Bacteria;Firmicutes;Bacilli;Lactobacillales;Lactobacillaceae;Lactobacillus;Lactobacillus satsumensis | 1 | 0.0042 |
| Lactobacillus vaginalis | Bacteria;Firmicutes;Bacilli;Lactobacillales;Lactobacillaceae;Lactobacillus;Lactobacillus vaginalis | 1 | 0.0042 |
| Luteimicrobium album group | Bacteria;Actinobacteria;Actinobacteria_c;Micrococcales;Promicromonosporaceae;Luteimicrobium;Luteimicrobium album group | 1 | 0.0042 |
| Lysinibacillus meyeri group | Bacteria;Firmicutes;Bacilli;Bacillales;Planococcaceae;Lysinibacillus;Lysinibacillus meyeri group | 1 | 0.0042 |
| Parapedobacter indicus | Bacteria;Bacteroidetes;Sphingobacteriia;Sphingobacteriales;Sphingobacteriaceae;Parapedobacter;Parapedobacter indicus | 1 | 0.0042 |
| Rhodococcus erythropolis group | Bacteria;Actinobacteria;Actinobacteria_c;Corynebacteriales;Nocardiaceae;Rhodococcus;Rhodococcus erythropolis group | 1 | 0.0042 |
| Rhodopseudomonas harwoodiae group | Bacteria;Proteobacteria;Alphaproteobacteria;Rhizobiales;Bradyrhizobiaceae;Rhodopseudomonas;Rhodopseudomonas harwoodiae group | 1 | 0.0042 |
| Ruminococcus faecis | Bacteria;Firmicutes;Clostridia;Clostridiales;Lachnospiraceae;Ruminococcus_g4;Ruminococcus faecis | 1 | 0.0042 |
| PAC000661_g AB185622_s | Bacteria;Firmicutes;Clostridia;Clostridiales;Ruminococcaceae;PAC000661_g;AB185622_s | 1 | 0.0042 |
| Bacteroides AB599946_s | Bacteria;Bacteroidetes;Bacteroidia;Bacteroidales;Bacteroidaceae;Bacteroides;AB599946_s | 1 | 0.0042 |
| Blautia AJ408961_s | Bacteria;Firmicutes;Clostridia;Clostridiales;Lachnospiraceae;Blautia;AJ408961_s | 1 | 0.0042 |
| Turicibacter AJ576409_s | Bacteria;Firmicutes;Erysipelotrichi;Erysipelotrichales;Erysipelotrichaceae;Turicibacter;AJ576409_s | 1 | 0.0042 |
| PAC001115_g AY858528_s | Bacteria;Firmicutes;Clostridia;Clostridiales;Christensenellaceae;PAC001115_g;AY858528_s | 1 | 0.0042 |
| Caproiciproducens DQ777923_s | Bacteria;Firmicutes;Clostridia;Clostridiales;Ruminococcaceae;Caproiciproducens;DQ777923_s | 1 | 0.0042 |
| Bacteroides DQ799557_s | Bacteria;Bacteroidetes;Bacteroidia;Bacteroidales;Bacteroidaceae;Bacteroides;DQ799557_s | 1 | 0.0042 |
| KE159571_g DQ815838_s | Bacteria;Firmicutes;Clostridia;Clostridiales;Lachnospiraceae;KE159571_g;DQ815838_s | 1 | 0.0042 |
| KE159600_g EF098562_s | Bacteria;Firmicutes;Clostridia;Clostridiales;Lachnospiraceae;KE159600_g;EF098562_s | 1 | 0.0042 |
| PAC001219_g EF602805_s | Bacteria;Firmicutes;Clostridia;Clostridiales;Christensenellaceae;PAC001219_g;EF602805_s | 1 | 0.0042 |
| PAC001165_g EF603797_s | Bacteria;Firmicutes;Clostridia;Clostridiales;Lachnospiraceae;PAC001165_g;EF603797_s | 1 | 0.0042 |
| EU132518_g EU132518_s | Bacteria;Actinobacteria;Actinobacteria_c;Frankiales;Frankiaceae;EU132518_g;EU132518_s | 1 | 0.0042 |
| Oscillibacter EU454374_s | Bacteria;Firmicutes;Clostridia;Clostridiales;Ruminococcaceae;Oscillibacter;EU454374_s | 1 | 0.0042 |
| Phocea EU457191_s | Bacteria;Firmicutes;Clostridia;Clostridiales;Ruminococcaceae;Phocea;EU457191_s | 1 | 0.0042 |
| PAC000664_g EU504374_s | Bacteria;Firmicutes;Clostridia;Clostridiales;Lachnospiraceae;PAC000664_g;EU504374_s | 1 | 0.0042 |
| PAC001573_g EU509323_s | Bacteria;Firmicutes;Clostridia;Clostridiales;Mogibacterium_f;PAC001573_g;EU509323_s | 1 | 0.0042 |
| Eubacterium_g23 EU509480_s | Bacteria;Firmicutes;Clostridia;Clostridiales;Ruminococcaceae;Eubacterium_g23;EU509480_s | 1 | 0.0042 |
| LLKB_g EU510160_s | Bacteria;Firmicutes;Clostridia;Clostridiales;Lachnospiraceae;LLKB_g;EU510160_s | 1 | 0.0042 |
| KE159600_g EU511097_s | Bacteria;Firmicutes;Clostridia;Clostridiales;Lachnospiraceae;KE159600_g;EU511097_s | 1 | 0.0042 |
| KE159600_g EU511112_s | Bacteria;Firmicutes;Clostridia;Clostridiales;Lachnospiraceae;KE159600_g;EU511112_s | 1 | 0.0042 |
| Bacteroides FQXY_s | Bacteria;Bacteroidetes;Bacteroidia;Bacteroidales;Bacteroidaceae;Bacteroides;FQXY_s | 1 | 0.0042 |
| HQ158649_g GU454872_s | Bacteria;Bacteroidetes;Sphingobacteriia;Saprospirales;Saprospiraceae;HQ158649_g;GU454872_s | 1 | 0.0042 |
| GU454944_g GU454944_s | Bacteria;Bacteroidetes;Cytophagia;Cytophagales;Cytophagaceae;GU454944_g;GU454944_s | 1 | 0.0042 |
| HM124077_g HM124077_s | Bacteria;Bacteroidetes;Bacteroidia;Bacteroidales;Muribaculaceae;HM124077_g;HM124077_s | 1 | 0.0042 |
| PAC001219_g HM124151_s | Bacteria;Firmicutes;Clostridia;Clostridiales;Christensenellaceae;PAC001219_g;HM124151_s | 1 | 0.0042 |
| Bacteroides JN559675_s | Bacteria;Bacteroidetes;Bacteroidia;Bacteroidales;Bacteroidaceae;Bacteroides;JN559675_s | 1 | 0.0042 |
| Methylotenera JUHH_s | Bacteria;Proteobacteria;Betaproteobacteria;Methylophilales;Methylophilaceae;Methylotenera;JUHH_s | 1 | 0.0042 |
| KE159605_g KB822463_s | Bacteria;Firmicutes;Clostridia;Clostridiales;Lachnospiraceae;KE159605_g;KB822463_s | 1 | 0.0042 |
| KE159571_g KE159567_s | Bacteria;Firmicutes;Clostridia;Clostridiales;Lachnospiraceae;KE159571_g;KE159567_s | 1 | 0.0042 |
| PAC000195_g PAC000195_s | Bacteria;Firmicutes;Clostridia;Clostridiales;Lachnospiraceae;PAC000195_g;PAC000195_s | 1 | 0.0042 |
| PAC000197_g PAC000197_s | Bacteria;Tenericutes;Mollicutes;PAC001057_o;PAC000197_f;PAC000197_g;PAC000197_s | 1 | 0.0042 |
| Frisingicoccus PAC000700_s | Bacteria;Firmicutes;Clostridia;Clostridiales;Lachnospiraceae;Frisingicoccus;PAC000700_s | 1 | 0.0042 |
| PAC001062_g PAC001062_s | Bacteria;Bacteroidetes;Bacteroidia;Bacteroidales;Muribaculaceae;PAC001062_g;PAC001062_s | 1 | 0.0042 |
| Alloprevotella PAC001102_s | Bacteria;Bacteroidetes;Bacteroidia;Bacteroidales;Prevotellaceae;Alloprevotella;PAC001102_s | 1 | 0.0042 |
| Eubacterium_g17 PAC001119_s | Bacteria;Firmicutes;Clostridia;Clostridiales;Lachnospiraceae;Eubacterium_g17;PAC001119_s | 1 | 0.0042 |
| PAC000197_g PAC001123_s | Bacteria;Tenericutes;Mollicutes;PAC001057_o;PAC000197_f;PAC000197_g;PAC001123_s | 1 | 0.0042 |
| Christensenella PAC001140_s | Bacteria;Firmicutes;Clostridia;Clostridiales;Christensenellaceae;Christensenella;PAC001140_s | 1 | 0.0042 |
| PAC001149_g PAC001149_s | Bacteria;Firmicutes;Clostridia;Clostridiales;Lachnospiraceae;PAC001149_g;PAC001149_s | 1 | 0.0042 |
| PAC000661_g PAC001154_s | Bacteria;Firmicutes;Clostridia;Clostridiales;Ruminococcaceae;PAC000661_g;PAC001154_s | 1 | 0.0042 |
| PAC001301_g PAC001301_s | Bacteria;Firmicutes;Clostridia;Clostridiales;Christensenellaceae;PAC001301_g;PAC001301_s | 1 | 0.0042 |
| Prevotella PAC001304_s | Bacteria;Bacteroidetes;Bacteroidia;Bacteroidales;Prevotellaceae;Prevotella;PAC001304_s | 1 | 0.0042 |
| PAC001165_g PAC001366_s | Bacteria;Firmicutes;Clostridia;Clostridiales;Lachnospiraceae;PAC001165_g;PAC001366_s | 1 | 0.0042 |
| Clostridium_g24 PAC001374_s | Bacteria;Firmicutes;Clostridia;Clostridiales;Lachnospiraceae;Clostridium_g24;PAC001374_s | 1 | 0.0042 |
| PAC001375_g PAC001375_s | Bacteria;Firmicutes;Clostridia;Clostridiales;Lachnospiraceae;PAC001375_g;PAC001375_s | 1 | 0.0042 |
| PAC001386_g PAC001386_s | Bacteria;Firmicutes;Clostridia;Clostridiales;Lachnospiraceae;PAC001386_g;PAC001386_s | 1 | 0.0042 |
| PAC000692_g PAC001387_s | Bacteria;Firmicutes;Clostridia;Clostridiales;Lachnospiraceae;PAC000692_g;PAC001387_s | 1 | 0.0042 |
| PAC001092_g PAC001388_s | Bacteria;Firmicutes;Clostridia;Clostridiales;Lachnospiraceae;PAC001092_g;PAC001388_s | 1 | 0.0042 |
| PAC001408_g PAC001408_s | Bacteria;Firmicutes;Clostridia;Clostridiales;Lachnospiraceae;PAC001408_g;PAC001408_s | 1 | 0.0042 |
| Acetatifactor PAC001463_s | Bacteria;Firmicutes;Clostridia;Clostridiales;Lachnospiraceae;Acetatifactor;PAC001463_s | 1 | 0.0042 |
| PAC001457_g PAC001495_s group | Bacteria;Firmicutes;Clostridia;Clostridiales;Lachnospiraceae;PAC001457_g;PAC001495_s group | 1 | 0.0042 |
| PAC001092_g PAC001517_s | Bacteria;Firmicutes;Clostridia;Clostridiales;Lachnospiraceae;PAC001092_g;PAC001517_s | 1 | 0.0042 |
| Oscillibacter PAC001522_s | Bacteria;Firmicutes;Clostridia;Clostridiales;Ruminococcaceae;Oscillibacter;PAC001522_s | 1 | 0.0042 |
| PAC001525_g PAC001525_s | Bacteria;Firmicutes;Clostridia;Clostridiales;Lachnospiraceae;PAC001525_g;PAC001525_s | 1 | 0.0042 |
| Agathobaculum PAC001688_s | Bacteria;Firmicutes;Clostridia;Clostridiales;Ruminococcaceae;Agathobaculum;PAC001688_s | 1 | 0.0042 |
| KE159538_g PAC001696_s | Bacteria;Firmicutes;Clostridia;Clostridiales;Lachnospiraceae;KE159538_g;PAC001696_s | 1 | 0.0042 |
| PAC000661_g PAC001716_s | Bacteria;Firmicutes;Clostridia;Clostridiales;Ruminococcaceae;PAC000661_g;PAC001716_s | 1 | 0.0042 |
| PAC001103_g PAC001724_s group | Bacteria;Firmicutes;Clostridia;Clostridiales;Lachnospiraceae;PAC001103_g;PAC001724_s group | 1 | 0.0042 |
| PAC001516_g PAC001725_s | Bacteria;Firmicutes;Clostridia;Clostridiales;Lachnospiraceae;PAC001516_g;PAC001725_s | 1 | 0.0042 |
| PAC001092_g PAC001726_s | Bacteria;Firmicutes;Clostridia;Clostridiales;Lachnospiraceae;PAC001092_g;PAC001726_s | 1 | 0.0042 |
| PAC001043_g PAC001752_s | Bacteria;Firmicutes;Clostridia;Clostridiales;Lachnospiraceae;PAC001043_g;PAC001752_s | 1 | 0.0042 |
| Clostridium_g21 PAC001757_s group | Bacteria;Firmicutes;Clostridia;Clostridiales;Lachnospiraceae;Clostridium_g21;PAC001757_s group | 1 | 0.0042 |
| PAC001068_g PAC001766_s | Bacteria;Bacteroidetes;Bacteroidia;Bacteroidales;Muribaculaceae;PAC001068_g;PAC001766_s | 1 | 0.0042 |
| Clostridium_g21 PAC001772_s | Bacteria;Firmicutes;Clostridia;Clostridiales;Lachnospiraceae;Clostridium_g21;PAC001772_s | 1 | 0.0042 |
| PAC001212_g PAC001809_s | Bacteria;Firmicutes;Clostridia;Clostridiales;Lachnospiraceae;PAC001212_g;PAC001809_s | 1 | 0.0042 |
| HM748739_g PAC001826_s group | Bacteria;Acidobacteria;Acidobacteriia;Acidobacteriales;Acidobacteriaceae;HM748739_g;PAC001826_s group | 1 | 0.0042 |
| PAC001225_g PAC002005_s | Bacteria;Firmicutes;Clostridia;Clostridiales;Lachnospiraceae;PAC001225_g;PAC002005_s | 1 | 0.0042 |
| PAC002042_g PAC002042_s | Bacteria;Firmicutes;Clostridia;Clostridiales;Lachnospiraceae;PAC002042_g;PAC002042_s | 1 | 0.0042 |
| Pseudoflavonifractor PAC002302_s | Bacteria;Firmicutes;Clostridia;Clostridiales;Ruminococcaceae;Pseudoflavonifractor;PAC002302_s | 1 | 0.0042 |
| PAC002331_g PAC002430_s | Bacteria;Bacteroidetes;Sphingobacteriia;Sphingobacteriales;Chitinophagaceae;PAC002331_g;PAC002430_s | 1 | 0.0042 |
| Parasutterella PAC002476_s | Bacteria;Proteobacteria;Betaproteobacteria;Burkholderiales;Sutterellaceae;Parasutterella;PAC002476_s | 1 | 0.0042 |
| PAC001360_g PAC002505_s | Bacteria;Firmicutes;Clostridia;Clostridiales;Christensenellaceae;PAC001360_g;PAC002505_s | 1 | 0.0042 |
| GQ264307_g_uc | Bacteria;Bacteroidetes;Sphingobacteriia;Sphingobacteriales;Chitinophagaceae;GQ264307_g;GQ264307_g_uc | 1 | 0.0042 |
| PAC000664_g_uc | Bacteria;Firmicutes;Clostridia;Clostridiales;Lachnospiraceae;PAC000664_g;PAC000664_g_uc | 1 | 0.0042 |

**Set 1_0.05%_S: Day 28_heme-SCP 0.05%_Species**

| Taxon name | Taxonomy | Count | Proportion(%) |
|---|---|---|---|
| Akkermansia muciniphila | Bacteria;Verrucomicrobia;Verrucomicrobiae;Verrucomicrobiales;Akkermansiaceae;Akkermansia;Akkermansia muciniphila | 3369 | 15.1579 |
| Bacteroides HM124113_s | Bacteria;Bacteroidetes;Bacteroidia;Bacteroidales;Bacteroidaceae;Bacteroides;HM124113_s | 1527 | 6.8703 |
| PAC001118_g PAC002344_s | Bacteria;Firmicutes;Clostridia;Clostridiales;Lachnospiraceae;PAC001118_g;PAC002344_s | 1410 | 6.3439 |
| KE159600_g KE159600_s | Bacteria;Firmicutes;Clostridia;Clostridiales;Lachnospiraceae;KE159600_g;KE159600_s | 681 | 3.064 |
| Lactobacillus gasseri group | Bacteria;Firmicutes;Bacilli;Lactobacillales;Lactobacillaceae;Lactobacillus;Lactobacillus gasseri group | 640 | 2.8795 |
| KE159810_g KE159810_s | Bacteria;Firmicutes;Clostridia;Clostridiales;Lachnospiraceae;KE159810_g;KE159810_s | 515 | 2.3171 |
| PAC001516_g PAC001725_s | Bacteria;Firmicutes;Clostridia;Clostridiales;Lachnospiraceae;PAC001516_g;PAC001725_s | 505 | 2.2721 |
| Flintibacter butyricus group | Bacteria;Firmicutes;Clostridia;Clostridiales;Ruminococcaceae;Pseudoflavonifractor;Flintibacter butyricus group | 475 | 2.1371 |
| Anaerotignum PAC001740_s | Bacteria;Firmicutes;Clostridia;Clostridiales;Lachnospiraceae;Anaerotignum;PAC001740_s | 400 | 1.7997 |
| PAC000664_g PAC001104_s | Bacteria;Firmicutes;Clostridia;Clostridiales;Lachnospiraceae;PAC000664_g;PAC001104_s | 380 | 1.7097 |
| Lactococcus lactis group | Bacteria;Firmicutes;Bacilli;Lactobacillales;Streptococcaceae;Lactococcus;Lactococcus lactis group | 370 | 1.6647 |
| Oscillibacter PAC001186_s group | Bacteria;Firmicutes;Clostridia;Clostridiales;Ruminococcaceae;Oscillibacter;PAC001186_s group | 352 | 1.5837 |
| Lactobacillus reuteri group | Bacteria;Firmicutes;Bacilli;Lactobacillales;Lactobacillaceae;Lactobacillus;Lactobacillus reuteri group | 345 | 1.5522 |
| PAC001141_g PAC001141_s | Bacteria;Firmicutes;Clostridia;Clostridiales;Christensenellaceae;PAC001141_g;PAC001141_s | 330 | 1.4847 |
| PAC002482_g EF603735_s | Bacteria;Bacteroidetes;Bacteroidia;Bacteroidales;AC160630_f;PAC002482_g;EF603735_s | 328 | 1.4757 |
| KE159600_g PAC001232_s | Bacteria;Firmicutes;Clostridia;Clostridiales;Lachnospiraceae;KE159600_g;PAC001232_s | 308 | 1.3858 |
| PAC001402_g PAC001402_s | Bacteria;Firmicutes;Clostridia;Clostridiales;Ruminococcaceae;PAC001402_g;PAC001402_s | 288 | 1.2958 |
| Bacteroides acidifaciens | Bacteria;Bacteroidetes;Bacteroidia;Bacteroidales;Bacteroidaceae;Bacteroides;Bacteroides acidifaciens | 279 | 1.2553 |
| PAC001452_g PAC001981_s group | Bacteria;Firmicutes;Clostridia;Clostridiales;Lachnospiraceae;PAC001452_g;PAC001981_s group | 277 | 1.2463 |
| PAC001738_g PAC001738_s | Bacteria;Firmicutes;Clostridia;Clostridiales;Lachnospiraceae;PAC001738_g;PAC001738_s | 263 | 1.1833 |
| Odoribacter DQ815763_s | Bacteria;Bacteroidetes;Bacteroidia;Bacteroidales;Odoribacteraceae;Odoribacter;DQ815763_s | 259 | 1.1653 |
| PAC001074_g PAC001074_s | Bacteria;Bacteroidetes;Bacteroidia;Bacteroidales;Muribaculaceae;PAC001074_g;PAC001074_s | 258 | 1.1608 |
| Helicobacter japonicus | Bacteria;Proteobacteria;Epsilonproteobacteria;Campylobacterales;Helicobacteraceae;Helicobacter;Helicobacter japonicus | 254 | 1.1428 |
| Acetatifactor HM124297_s | Bacteria;Firmicutes;Clostridia;Clostridiales;Lachnospiraceae;Acetatifactor;HM124297_s | 254 | 1.1428 |
| Anaerotruncus KE159677_s | Bacteria;Firmicutes;Clostridia;Clostridiales;Ruminococcaceae;Anaerotruncus;KE159677_s | 250 | 1.1248 |
| Bilophila wadsworthia | Bacteria;Proteobacteria;Deltaproteobacteria;Desulfovibrionales;Desulfovibrionaceae;Bilophila;Bilophila wadsworthia | 244 | 1.0978 |
| Clostridium_g21 PAC001294_s | Bacteria;Firmicutes;Clostridia;Clostridiales;Lachnospiraceae;Clostridium_g21;PAC001294_s | 234 | 1.0528 |
| Pseudoflavonifractor PAC001574_s group | Bacteria;Firmicutes;Clostridia;Clostridiales;Ruminococcaceae;Pseudoflavonifractor;PAC001574_s group | 207 | 0.9313 |
| Lactobacillus murinus group | Bacteria;Firmicutes;Bacilli;Lactobacillales;Lactobacillaceae;Lactobacillus;Lactobacillus murinus group | 204 | 0.9178 |
| PAC001516_g PAC001516_s | Bacteria;Firmicutes;Clostridia;Clostridiales;Lachnospiraceae;PAC001516_g;PAC001516_s | 188 | 0.8459 |
| PAC000661_g PAC001716_s | Bacteria;Firmicutes;Clostridia;Clostridiales;Ruminococcaceae;PAC000661_g;PAC001716_s | 169 | 0.7604 |
| Oscillibacter PAC001501_s | Bacteria;Firmicutes;Clostridia;Clostridiales;Ruminococcaceae;Oscillibacter;PAC001501_s | 163 | 0.7334 |
| Eubacterium_g23 PAC001121_s group | Bacteria;Firmicutes;Clostridia;Clostridiales;Ruminococcaceae;Eubacterium_g23;PAC001121_s group | 161 | 0.7244 |
| PAC000664_g EU504374_s | Bacteria;Firmicutes;Clostridia;Clostridiales;Lachnospiraceae;PAC000664_g;EU504374_s | 157 | 0.7064 |
| Bacteroides_uc | Bacteria;Bacteroidetes;Bacteroidia;Bacteroidales;Bacteroidaceae;Bacteroides;Bacteroides_uc | 156 | 0.7019 |
| Alistipes PAC001061_s | Bacteria;Bacteroidetes;Bacteroidia;Bacteroidales;Rikenellaceae;Alistipes;PAC001061_s | 154 | 0.6929 |
| PAC001402_g PAC001748_s | Bacteria;Firmicutes;Clostridia;Clostridiales;Ruminococcaceae;PAC001402_g;PAC001748_s | 152 | 0.6839 |
| PAC001112_g PAC001267_s | Bacteria;Bacteroidetes;Bacteroidia;Bacteroidales;Muribaculaceae;PAC001112_g;PAC001267_s | 140 | 0.6299 |
| PAC000198_g PAC001075_s | Bacteria;Bacteroidetes;Bacteroidia;Bacteroidales;Muribaculaceae;PAC000198_g;PAC001075_s | 138 | 0.6209 |
| Bacteroides vulgatus | Bacteria;Bacteroidetes;Bacteroidia;Bacteroidales;Bacteroidaceae;Bacteroides;Bacteroides vulgatus | 131 | 0.5894 |
| Frisingicoccus PAC001744_s group | Bacteria;Firmicutes;Clostridia;Clostridiales;Lachnospiraceae;Frisingicoccus;PAC001744_s group | 129 | 0.5804 |
| PAC001544_g PAC001544_s | Bacteria;Firmicutes;Clostridia;Clostridiales;Lachnospiraceae;PAC001544_g;PAC001544_s | 126 | 0.5669 |
| Acetatifactor PAC001801_s | Bacteria;Firmicutes;Clostridia;Clostridiales;Lachnospiraceae;Acetatifactor;PAC001801_s | 126 | 0.5669 |
| Clostridium_g21 PAC001085_s | Bacteria;Firmicutes;Clostridia;Clostridiales;Lachnospiraceae;Clostridium_g21;PAC001085_s | 118 | 0.5309 |
| Bacteroides stercorirosoris | Bacteria;Bacteroidetes;Bacteroidia;Bacteroidales;Bacteroidaceae;Bacteroides;Bacteroides stercorirosoris | 115 | 0.5174 |
| Acetatifactor PAC001463_s | Bacteria;Firmicutes;Clostridia;Clostridiales;Lachnospiraceae;Acetatifactor;PAC001463_s | 115 | 0.5174 |
| PAC001400_g PAC001400_s | Bacteria;Firmicutes;Clostridia;Clostridiales;Peptococcaceae;PAC001400_g;PAC001400_s | 101 | 0.4544 |
| Desulfovibrio PAC001079_s | Bacteria;Proteobacteria;Deltaproteobacteria;Desulfovibrionales;Desulfovibrionaceae;Desulfovibrio;PAC001079_s | 99 | 0.4454 |
| KE159571_g PAC001497_s | Bacteria;Firmicutes;Clostridia;Clostridiales;Lachnospiraceae;KE159571_g;PAC001497_s | 99 | 0.4454 |
| Pseudoflavonifractor PAC001550_s | Bacteria;Firmicutes;Clostridia;Clostridiales;Ruminococcaceae;Pseudoflavonifractor;PAC001550_s | 88 | 0.3959 |
| Pseudoflavonifractor PAC002397_s | Bacteria;Firmicutes;Clostridia;Clostridiales;Ruminococcaceae;Pseudoflavonifractor;PAC002397_s | 87 | 0.3914 |
| Acetatifactor PAC001773_s | Bacteria;Firmicutes;Clostridia;Clostridiales;Lachnospiraceae;Acetatifactor;PAC001773_s | 85 | 0.3824 |
| PAC001402_g PAC001764_s | Bacteria;Firmicutes;Clostridia;Clostridiales;Ruminococcaceae;PAC001402_g;PAC001764_s | 83 | 0.3734 |
| KE159797_g_uc | Bacteria;Firmicutes;Clostridia;Clostridiales;Lachnospiraceae;KE159797_g;KE159797_g_uc | 76 | 0.3419 |
| PAC000661_g EU843173_s | Bacteria;Firmicutes;Clostridia;Clostridiales;Ruminococcaceae;PAC000661_g;EU843173_s | 75 | 0.3374 |
| KE159538_g PAC001093_s | Bacteria;Firmicutes;Clostridia;Clostridiales;Lachnospiraceae;KE159538_g;PAC001093_s | 75 | 0.3374 |
| Pseudoflavonifractor PAC001543_s | Bacteria;Firmicutes;Clostridia;Clostridiales;Ruminococcaceae;Pseudoflavonifractor;PAC001543_s | 74 | 0.3329 |
| PAC001221_g PAC001221_s | Bacteria;Firmicutes;Clostridia;Clostridiales;Dehalobacterium_f;PAC001221_g;PAC001221_s | 73 | 0.3284 |
| Pseudoflavonifractor_uc | Bacteria;Firmicutes;Clostridia;Clostridiales;Ruminococcaceae;Pseudoflavonifractor;Pseudoflavonifractor_uc | 70 | 0.3149 |
| PAC000661_g_uc | Bacteria;Firmicutes;Clostridia;Clostridiales;Ruminococcaceae;PAC000661_g;PAC000661_g_uc | 68 | 0.3059 |
| Oscillibacter PAC001188_s | Bacteria;Firmicutes;Clostridia;Clostridiales;Ruminococcaceae;Oscillibacter;PAC001188_s | 64 | 0.288 |
| PAC001165_g PAC001384_s | Bacteria;Firmicutes;Clostridia;Clostridiales;Lachnospiraceae;PAC001165_g;PAC001384_s | 64 | 0.288 |
| LT706945_g PAC001747_s | Bacteria;Proteobacteria;Deltaproteobacteria;Desulfovibrionales;Desulfovibrionaceae;LT706945_g;PAC001747_s | 63 | 0.2835 |
| PAC001500_g PAC001500_s | Bacteria;Firmicutes;Clostridia;Clostridiales;Peptococcaceae;PAC001500_g;PAC001500_s | 61 | 0.2745 |
| Clostridium cocleatum | Bacteria;Firmicutes;Erysipelotrichi;Erysipelotrichales;Erysipelotrichaceae;Clostridium_g6;Clostridium cocleatum | 60 | 0.27 |
| Parabacteroides goldsteinii | Bacteria;Bacteroidetes;Bacteroidia;Bacteroidales;Porphyromonadaceae;Parabacteroides;Parabacteroides goldsteinii | 58 | 0.261 |
| PAC001068_g PAC001068_s | Bacteria;Bacteroidetes;Bacteroidia;Bacteroidales;Muribaculaceae;PAC001068_g;PAC001068_s | 58 | 0.261 |
| PAC001516_g PAC002384_s | Bacteria;Firmicutes;Clostridia;Clostridiales;Lachnospiraceae;PAC001516_g;PAC002384_s | 57 | 0.2565 |
| PAC000661_g PAC001095_s | Bacteria;Firmicutes;Clostridia;Clostridiales;Ruminococcaceae;PAC000661_g;PAC001095_s | 54 | 0.243 |
| Sporobacter PAC001096_s | Bacteria;Firmicutes;Clostridia;Clostridiales;Ruminococcaceae;Sporobacter;PAC001096_s | 54 | 0.243 |
| KE159600_g PAC001901_s | Bacteria;Firmicutes;Clostridia;Clostridiales;Lachnospiraceae;KE159600_g;PAC001901_s | 51 | 0.2295 |
| PAC001385_g PAC001727_s group | Bacteria;Firmicutes;Clostridia;Clostridiales;Lachnospiraceae;PAC001385_g;PAC001727_s group | 48 | 0.216 |
| Pseudoflavonifractor PAC001379_s | Bacteria;Firmicutes;Clostridia;Clostridiales;Ruminococcaceae;Pseudoflavonifractor;PAC001379_s | 46 | 0.207 |
| Corynebacterium glutamicum | Bacteria;Actinobacteria;Actinobacteria_c;Corynebacteriales;Corynebacteriaceae;Corynebacterium;Corynebacterium glutamicum | 45 | 0.2025 |
| Pseudoflavonifractor PAC001087_s | Bacteria;Firmicutes;Clostridia;Clostridiales;Ruminococcaceae;Pseudoflavonifractor;PAC001087_s | 45 | 0.2025 |
| PAC001066_g PAC001066_s | Bacteria;Bacteroidetes;Bacteroidia;Bacteroidales;Muribaculaceae;PAC001066_g;PAC001066_s | 43 | 0.1935 |
| LARJ_g PAC001927_s | Bacteria;Proteobacteria;Alphaproteobacteria;Rhodospirillales;Rhodospirillaceae;LARJ_g;PAC001927_s | 41 | 0.1845 |
| Acetatifactor PAC002332_s | Bacteria;Firmicutes;Clostridia;Clostridiales;Lachnospiraceae;Acetatifactor;PAC002332_s | 39 | 0.1755 |
| PAC001524_g PAC001524_s | Bacteria;Firmicutes;Clostridia;Clostridiales;Lachnospiraceae;PAC001524_g;PAC001524_s | 38 | 0.171 |
| Paludicola PAC001784_s | Bacteria;Firmicutes;Clostridia;Clostridiales;Ruminococcaceae;Paludicola;PAC001784_s | 37 | 0.1665 |
| KE159571_g KE159567_s | Bacteria;Firmicutes;Clostridia;Clostridiales;Lachnospiraceae;KE159571_g;KE159567_s | 35 | 0.1575 |
| Oscillibacter PAC001156_s | Bacteria;Firmicutes;Clostridia;Clostridiales;Ruminococcaceae;Oscillibacter;PAC001156_s | 35 | 0.1575 |
| PAC001138_g PAC001518_s | Bacteria;Firmicutes;Clostridia;Clostridiales;Lachnospiraceae;PAC001138_g;PAC001518_s | 35 | 0.1575 |
| Oscillibacter PAC001526_s | Bacteria;Firmicutes;Clostridia;Clostridiales;Ruminococcaceae;Oscillibacter;PAC001526_s | 35 | 0.1575 |
| Oscillibacter PAC001584_s group | Bacteria;Firmicutes;Clostridia;Clostridiales;Ruminococcaceae;Oscillibacter;PAC001584_s group | 35 | 0.1575 |
| KE159571_g PAC001776_s group | Bacteria;Firmicutes;Clostridia;Clostridiales;Lachnospiraceae;KE159571_g;PAC001776_s group | 35 | 0.1575 |
| Clostridium_g21 KE159781_s | Bacteria;Firmicutes;Clostridia;Clostridiales;Lachnospiraceae;Clostridium_g21;KE159781_s | 34 | 0.153 |
| PAC001103_g PAC001361_s | Bacteria;Firmicutes;Clostridia;Clostridiales;Lachnospiraceae;PAC001103_g;PAC001361_s | 34 | 0.153 |
| Oscillibacter PAC001522_s | Bacteria;Firmicutes;Clostridia;Clostridiales;Ruminococcaceae;Oscillibacter;PAC001522_s | 34 | 0.153 |
| PAC001092_g PAC001668_s | Bacteria;Firmicutes;Clostridia;Clostridiales;Lachnospiraceae;PAC001092_g;PAC001668_s | 34 | 0.153 |
| Frisingicoccus EF409953_s | Bacteria;Firmicutes;Clostridia;Clostridiales;Lachnospiraceae;Frisingicoccus;EF409953_s | 32 | 0.144 |
| PAC001043_g PAC002511_s group | Bacteria;Firmicutes;Clostridia;Clostridiales;Lachnospiraceae;PAC001043_g;PAC002511_s group | 32 | 0.144 |
| Mucispirillum schaedleri | Bacteria;Deferribacteres;Deferribacteres_c;Deferribacterales;Deferribacteraceae;Mucispirillum;Mucispirillum schaedleri | 30 | 0.135 |
| Oscillibacter KE159714_s group | Bacteria;Firmicutes;Clostridia;Clostridiales;Ruminococcaceae;Oscillibacter;KE159714_s group | 30 | 0.135 |
| PAC001228_g PAC002027_s | Bacteria;Firmicutes;Clostridia;Clostridiales;Lachnospiraceae;PAC001228_g;PAC002027_s | 30 | 0.135 |
| Acetatifactor_uc | Bacteria;Firmicutes;Clostridia;Clostridiales;Lachnospiraceae;Acetatifactor;Acetatifactor_uc | 30 | 0.135 |
| Alistipes PAC001471_s | Bacteria;Bacteroidetes;Bacteroidia;Bacteroidales;Rikenellaceae;Alistipes;PAC001471_s | 29 | 0.1305 |
| PAC001457_g PAC001495_s group | Bacteria;Firmicutes;Clostridia;Clostridiales;Lachnospiraceae;PAC001457_g;PAC001495_s group | 29 | 0.1305 |
| Oscillibacter PAC001638_s | Bacteria;Firmicutes;Clostridia;Clostridiales;Ruminococcaceae;Oscillibacter;PAC001638_s | 28 | 0.126 |
| Eubacterium_g6 JQ083736_s | Bacteria;Firmicutes;Clostridia;Clostridiales;Lachnospiraceae;Eubacterium_g6;JQ083736_s | 27 | 0.1215 |
| KE159600_g EU511097_s | Bacteria;Firmicutes;Clostridia;Clostridiales;Lachnospiraceae;KE159600_g;EU511097_s | 26 | 0.117 |
| Eubacterium_g4 PAC001174_s | Bacteria;Firmicutes;Clostridia;Clostridiales;Lachnospiraceae;Eubacterium_g4;PAC001174_s | 26 | 0.117 |
| PAC001485_g PAC001485_s | Bacteria;Bacteroidetes;Bacteroidia;Bacteroidales;Muribaculaceae;PAC001485_g;PAC001485_s | 26 | 0.117 |
| PAC001092_g PAC001774_s | Bacteria;Firmicutes;Clostridia;Clostridiales;Lachnospiraceae;PAC001092_g;PAC001774_s | 25 | 0.1125 |
| PAC002039_g PAC002039_s | Bacteria;Firmicutes;Clostridia;Clostridiales;Lachnospiraceae;PAC002039_g;PAC002039_s | 25 | 0.1125 |
| Eubacterium_g6 EU510495_s | Bacteria;Firmicutes;Clostridia;Clostridiales;Lachnospiraceae;Eubacterium_g6;EU510495_s | 24 | 0.108 |
| PAC000748_g PAC001099_s | Bacteria;Firmicutes;Clostridia;Clostridiales;Ruminococcaceae;PAC000748_g;PAC001099_s | 23 | 0.1035 |
| Pseudoflavonifractor PAC001370_s | Bacteria;Firmicutes;Clostridia;Clostridiales;Ruminococcaceae;Pseudoflavonifractor;PAC001370_s | 23 | 0.1035 |
| Caproiciproducens PAC001505_s | Bacteria;Firmicutes;Clostridia;Clostridiales;Ruminococcaceae;Caproiciproducens;PAC001505_s | 23 | 0.1035 |
| Pseudoflavonifractor PAC001549_s | Bacteria;Firmicutes;Clostridia;Clostridiales;Ruminococcaceae;Pseudoflavonifractor;PAC001549_s | 23 | 0.1035 |
| PAC000186_g PAC001806_s | Bacteria;Bacteroidetes;Bacteroidia;Bacteroidales;Muribaculaceae;PAC000186_g;PAC001806_s | 23 | 0.1035 |
| Alistipes PAC001060_s | Bacteria;Bacteroidetes;Bacteroidia;Bacteroidales;Rikenellaceae;Alistipes;PAC001060_s | 22 | 0.099 |
| Pseudoflavonifractor PAC001547_s | Bacteria;Firmicutes;Clostridia;Clostridiales;Ruminococcaceae;Pseudoflavonifractor;PAC001547_s | 22 | 0.099 |
| PAC001219_g HM124151_s | Bacteria;Firmicutes;Clostridia;Clostridiales;Christensenellaceae;PAC001219_g;HM124151_s | 21 | 0.0945 |
| Pseudoflavonifractor KJ535319_s | Bacteria;Firmicutes;Clostridia;Clostridiales;Ruminococcaceae;Pseudoflavonifractor;KJ535319_s | 21 | 0.0945 |
| PAC001219_g PAC001280_s | Bacteria;Firmicutes;Clostridia;Clostridiales;Christensenellaceae;PAC001219_g;PAC001280_s | 21 | 0.0945 |
| PAC001092_g PAC001710_s | Bacteria;Firmicutes;Clostridia;Clostridiales;Lachnospiraceae;PAC001092_g;PAC001710_s | 21 | 0.0945 |
| Lachnospiraceae_uc | Bacteria;Firmicutes;Clostridia;Clostridiales;Lachnospiraceae;Lachnospiraceae_uc | 20 | 0.09 |
| PAC001372_g PAC001372_s | Bacteria;Firmicutes;Clostridia;Clostridiales;Lachnospiraceae;PAC001372_g;PAC001372_s | 20 | 0.09 |
| Pseudoflavonifractor PAC001548_s | Bacteria;Firmicutes;Clostridia;Clostridiales;Ruminococcaceae;Pseudoflavonifractor;PAC001548_s | 20 | 0.09 |
| PAC001516_g_uc | Bacteria;Firmicutes;Clostridia;Clostridiales;Lachnospiraceae;PAC001516_g;PAC001516_g_uc | 20 | 0.09 |
| Oscillibacter PAC001369_s | Bacteria;Firmicutes;Clostridia;Clostridiales;Ruminococcaceae;Oscillibacter;PAC001369_s | 18 | 0.081 |
| KE159571_g PAC001555_s | Bacteria;Firmicutes;Clostridia;Clostridiales;Lachnospiraceae;KE159571_g;PAC001555_s | 18 | 0.081 |
| Frisingicoccus PAC001728_s group | Bacteria;Firmicutes;Clostridia;Clostridiales;Lachnospiraceae;Frisingicoccus;PAC001728_s group | 18 | 0.081 |
| Clostridium_g21 EU506235_s | Bacteria;Firmicutes;Clostridia;Clostridiales;Lachnospiraceae;Clostridium_g21;EU506235_s | 17 | 0.0765 |
| PAC001092_g PAC001230_s | Bacteria;Firmicutes;Clostridia;Clostridiales;Lachnospiraceae;PAC001092_g;PAC001230_s | 17 | 0.0765 |
| PAC001108_g PAC001531_s | Bacteria;Tenericutes;Mollicutes;PAC001057_o;PAC001057_f;PAC001108_g;PAC001531_s | 17 | 0.0765 |
| KE159571_g PAC001705_s | Bacteria;Firmicutes;Clostridia;Clostridiales;Lachnospiraceae;KE159571_g;PAC001705_s | 17 | 0.0765 |
| PAC001778_g PAC001778_s | Bacteria;Firmicutes;Clostridia;Clostridiales;Ruminococcaceae;PAC001778_g;PAC001778_s | 17 | 0.0765 |
| Bacteroides uniformis | Bacteria;Bacteroidetes;Bacteroidia;Bacteroidales;Bacteroidaceae;Bacteroides;Bacteroides uniformis | 16 | 0.072 |
| Eubacterium_g6 PAC002402_s | Bacteria;Firmicutes;Clostridia;Clostridiales;Lachnospiraceae;Eubacterium_g6;PAC002402_s | 16 | 0.072 |
| PAC001112_g PAC002441_s | Bacteria;Bacteroidetes;Bacteroidia;Bacteroidales;Muribaculaceae;PAC001112_g;PAC002441_s | 16 | 0.072 |
| Arthromitus AP012202_s group | Bacteria;Firmicutes;Clostridia;Clostridiales;Clostridiaceae;Arthromitus;AP012202_s group | 15 | 0.0675 |
| PAC000661_g GQ358437_s | Bacteria;Firmicutes;Clostridia;Clostridiales;Ruminococcaceae;PAC000661_g;GQ358437_s | 15 | 0.0675 |
| KE159605_g KE159605_s | Bacteria;Firmicutes;Clostridia;Clostridiales;Lachnospiraceae;KE159605_g;KE159605_s | 15 | 0.0675 |
| Paludicola PAC000694_s | Bacteria;Firmicutes;Clostridia;Clostridiales;Ruminococcaceae;Paludicola;PAC000694_s | 15 | 0.0675 |
| PAC000186_g PAC001064_s | Bacteria;Bacteroidetes;Bacteroidia;Bacteroidales;Muribaculaceae;PAC000186_g;PAC001064_s | 15 | 0.0675 |
| Paludicola PAC001508_s | Bacteria;Firmicutes;Clostridia;Clostridiales;Ruminococcaceae;Paludicola;PAC001508_s | 15 | 0.0675 |
| PAC001402_g PAC001561_s | Bacteria;Firmicutes;Clostridia;Clostridiales;Ruminococcaceae;PAC001402_g;PAC001561_s | 15 | 0.0675 |
| Oscillibacter PAC002110_s | Bacteria;Firmicutes;Clostridia;Clostridiales;Ruminococcaceae;Oscillibacter;PAC002110_s | 15 | 0.0675 |
| Clostridium_g24 AF371567_s | Bacteria;Firmicutes;Clostridia;Clostridiales;Lachnospiraceae;Clostridium_g24;AF371567_s | 14 | 0.063 |
| KE159605_g KB822463_s | Bacteria;Firmicutes;Clostridia;Clostridiales;Lachnospiraceae;KE159605_g;KB822463_s | 14 | 0.063 |
| PAC000661_g PAC001154_s | Bacteria;Firmicutes;Clostridia;Clostridiales;Ruminococcaceae;PAC000661_g;PAC001154_s | 14 | 0.063 |
| PAC001068_g PAC001736_s | Bacteria;Bacteroidetes;Bacteroidia;Bacteroidales;Muribaculaceae;PAC001068_g;PAC001736_s | 14 | 0.063 |
| PAC000692_g PAC001745_s | Bacteria;Firmicutes;Clostridia;Clostridiales;Lachnospiraceae;PAC000692_g;PAC001745_s | 14 | 0.063 |
| Clostridium_g21 PAC001772_s | Bacteria;Firmicutes;Clostridia;Clostridiales;Lachnospiraceae;Clostridium_g21;PAC001772_s | 13 | 0.0585 |
| KE159571_g PAC002009_s group | Bacteria;Firmicutes;Clostridia;Clostridiales;Lachnospiraceae;KE159571_g;PAC002009_s group | 13 | 0.0585 |
| PAC001068_g PAC001071_s | Bacteria;Bacteroidetes;Bacteroidia;Bacteroidales;Muribaculaceae;PAC001068_g;PAC001071_s | 12 | 0.054 |
| Massilioclostridium PAC001521_s | Bacteria;Firmicutes;Clostridia;Clostridiales;Ruminococcaceae;Massilioclostridium;PAC001521_s | 11 | 0.0495 |
| Harryflintia PAC001713_s | Bacteria;Firmicutes;Clostridia;Clostridiales;Ruminococcaceae;Harryflintia;PAC001713_s | 11 | 0.0495 |
| PAC001165_g PAC001796_s | Bacteria;Firmicutes;Clostridia;Clostridiales;Lachnospiraceae;PAC001165_g;PAC001796_s | 11 | 0.0495 |
| PAC000197_g PAC002517_s | Bacteria;Tenericutes;Mollicutes;PAC001057_o;PAC000197_f;PAC000197_g;PAC002517_s | 11 | 0.0495 |
| KE159538_g_uc | Bacteria;Firmicutes;Clostridia;Clostridiales;Lachnospiraceae;KE159538_g;KE159538_g_uc | 11 | 0.0495 |
| PAC000197_g_uc | Bacteria;Tenericutes;Mollicutes;PAC001057_o;PAC000197_f;PAC000197_g;PAC000197_g_uc | 11 | 0.0495 |
| Anaerotruncus colihominis | Bacteria;Firmicutes;Clostridia;Clostridiales;Ruminococcaceae;Anaerotruncus;Anaerotruncus colihominis | 10 | 0.045 |
| Enterorhabdus caecimuris | Bacteria;Actinobacteria;Coriobacteriia;Coriobacteriales;Coriobacteriaceae;Enterorhabdus;Enterorhabdus caecimuris | 10 | 0.045 |
| Pseudoflavonifractor FJ879979_s | Bacteria;Firmicutes;Clostridia;Clostridiales;Ruminococcaceae;Pseudoflavonifractor;FJ879979_s | 10 | 0.045 |
| PAC001308_g PAC001308_s | Bacteria;Firmicutes;Clostridia;Clostridiales;Lachnospiraceae;PAC001308_g;PAC001308_s | 10 | 0.045 |
| KE159538_g PAC001708_s | Bacteria;Firmicutes;Clostridia;Clostridiales;Lachnospiraceae;KE159538_g;PAC001708_s | 10 | 0.045 |
| KE159571_g PAC002035_s | Bacteria;Firmicutes;Clostridia;Clostridiales;Lachnospiraceae;KE159571_g;PAC002035_s | 10 | 0.045 |
| PAC001219_g PAC002168_s | Bacteria;Firmicutes;Clostridia;Clostridiales;Christensenellaceae;PAC001219_g;PAC002168_s | 10 | 0.045 |
| Acutalibacter muris | Bacteria;Firmicutes;Clostridia;Clostridiales;Ruminococcaceae;Acutalibacter;Acutalibacter muris | 9 | 0.0405 |
| Escherichia coli group | Bacteria;Proteobacteria;Gammaproteobacteria;Enterobacterales;Enterobacteriaceae;Escherichia;Escherichia coli group | 9 | 0.0405 |
| KE159797_g KE159797_s | Bacteria;Firmicutes;Clostridia;Clostridiales;Lachnospiraceae;KE159797_g;KE159797_s | 9 | 0.0405 |
| Acholeplasma_g2 PAC001080_s | Bacteria;Tenericutes;Mollicutes;Acholeplasmatales;Acholeplasmataceae;Acholeplasma_g2;PAC001080_s | 9 | 0.0405 |
| PAC001109_g PAC001203_s | Bacteria;Tenericutes;Mollicutes;PAC001057_o;PAC001057_f;PAC001109_g;PAC001203_s | 9 | 0.0405 |
| PAC000664_g PAC001488_s group | Bacteria;Firmicutes;Clostridia;Clostridiales;Lachnospiraceae;PAC000664_g;PAC001488_s group | 9 | 0.0405 |
| Oscillibacter PAC001502_s | Bacteria;Firmicutes;Clostridia;Clostridiales;Ruminococcaceae;Oscillibacter;PAC001502_s | 9 | 0.0405 |
| Paludicola PAC001507_s | Bacteria;Firmicutes;Clostridia;Clostridiales;Ruminococcaceae;Paludicola;PAC001507_s | 9 | 0.0405 |
| PAC001144_g PAC001593_s | Bacteria;Firmicutes;Clostridia;Clostridiales;Ruminococcaceae;PAC001144_g;PAC001593_s | 9 | 0.0405 |
| Parabacteroides PAC001783_s | Bacteria;Bacteroidetes;Bacteroidia;Bacteroidales;Porphyromonadaceae;Parabacteroides;PAC001783_s | 9 | 0.0405 |
| Harryflintia PAC002149_s | Bacteria;Firmicutes;Clostridia;Clostridiales;Ruminococcaceae;Harryflintia;PAC002149_s | 9 | 0.0405 |
| Acetatifactor FJ881083_s | Bacteria;Firmicutes;Clostridia;Clostridiales;Lachnospiraceae;Acetatifactor;FJ881083_s | 8 | 0.036 |
| Acetatifactor HM845729_s | Bacteria;Firmicutes;Clostridia;Clostridiales;Lachnospiraceae;Acetatifactor;HM845729_s | 8 | 0.036 |
| KE159797_g PAC001222_s | Bacteria;Firmicutes;Clostridia;Clostridiales;Lachnospiraceae;KE159797_g;PAC001222_s | 8 | 0.036 |
| Frisingicoccus PAC001399_s | Bacteria;Firmicutes;Clostridia;Clostridiales;Lachnospiraceae;Frisingicoccus;PAC001399_s | 8 | 0.036 |
| Oscillibacter PAC002372_s | Bacteria;Firmicutes;Clostridia;Clostridiales;Ruminococcaceae;Oscillibacter;PAC002372_s | 8 | 0.036 |
| Clostridium_g21 PAC002512_s group | Bacteria;Firmicutes;Clostridia;Clostridiales;Lachnospiraceae;Clostridium_g21;PAC002512_s group | 8 | 0.036 |
| Bacteroides faecichinchillae | Bacteria;Bacteroidetes;Bacteroidia;Bacteroidales;Bacteroidaceae;Bacteroides;Bacteroides faecichinchillae | 7 | 0.0315 |
| PAC001457_g AB606250_s | Bacteria;Firmicutes;Clostridia;Clostridiales;Lachnospiraceae;PAC001457_g;AB606250_s | 7 | 0.0315 |
| PAC001219_g EF603174_s | Bacteria;Firmicutes;Clostridia;Clostridiales;Christensenellaceae;PAC001219_g;EF603174_s | 7 | 0.0315 |
| Clostridium_g24 PAC000183_s | Bacteria;Firmicutes;Clostridia;Clostridiales;Lachnospiraceae;Clostridium_g24;PAC000183_s | 7 | 0.0315 |
| Frisingicoccus PAC000688_s | Bacteria;Firmicutes;Clostridia;Clostridiales;Lachnospiraceae;Frisingicoccus;PAC000688_s | 7 | 0.0315 |
| PAC001165_g PAC001165_s | Bacteria;Firmicutes;Clostridia;Clostridiales;Lachnospiraceae;PAC001165_g;PAC001165_s | 7 | 0.0315 |
| PAC000692_g PAC001387_s | Bacteria;Firmicutes;Clostridia;Clostridiales;Lachnospiraceae;PAC000692_g;PAC001387_s | 7 | 0.0315 |
| PAC001402_g PAC001546_s | Bacteria;Firmicutes;Clostridia;Clostridiales;Ruminococcaceae;PAC001402_g;PAC001546_s | 7 | 0.0315 |
| PAC001043_g PAC001557_s | Bacteria;Firmicutes;Clostridia;Clostridiales;Lachnospiraceae;PAC001043_g;PAC001557_s | 7 | 0.0315 |
| Coprococcus_g2 PAC001722_s | Bacteria;Firmicutes;Clostridia;Clostridiales;Lachnospiraceae;Coprococcus_g2;PAC001722_s | 7 | 0.0315 |
| Eubacterium_g6 PAC001759_s | Bacteria;Firmicutes;Clostridia;Clostridiales;Lachnospiraceae;Eubacterium_g6;PAC001759_s | 7 | 0.0315 |
| PAC002314_g PAC002314_s | Bacteria;Firmicutes;Erysipelotrichi;Erysipelotrichales;Erysipelotrichaceae;PAC002314_g;PAC002314_s | 7 | 0.0315 |
| Clostridium_g21 PAC002492_s | Bacteria;Firmicutes;Clostridia;Clostridiales;Lachnospiraceae;Clostridium_g21;PAC002492_s | 7 | 0.0315 |
| LARJ_g_uc | Bacteria;Proteobacteria;Alphaproteobacteria;Rhodospirillales;Rhodospirillaceae;LARJ_g;LARJ_g_uc | 7 | 0.0315 |
| PAC001485_g_uc | Bacteria;Bacteroidetes;Bacteroidia;Bacteroidales;Muribaculaceae;PAC001485_g;PAC001485_g_uc | 7 | 0.0315 |
| Ruminococcaceae_uc | Bacteria;Firmicutes;Clostridia;Clostridiales;Ruminococcaceae;Ruminococcaceae_uc | 6 | 0.027 |
| Caproiciproducens DQ777923_s | Bacteria;Firmicutes;Clostridia;Clostridiales;Ruminococcaceae;Caproiciproducens;DQ777923_s | 6 | 0.027 |
| Clostridium_g24 EU504811_s | Bacteria;Firmicutes;Clostridia;Clostridiales;Lachnospiraceae;Clostridium_g24;EU504811_s | 6 | 0.027 |
| Eubacterium_g6 PAC001586_s | Bacteria;Firmicutes;Clostridia;Clostridiales;Lachnospiraceae;Eubacterium_g6;PAC001586_s | 6 | 0.027 |
| Pseudoflavonifractor PAC001786_s | Bacteria;Firmicutes;Clostridia;Clostridiales;Ruminococcaceae;Pseudoflavonifractor;PAC001786_s | 6 | 0.027 |
| PAC001270_g PAC001899_s group | Bacteria;Firmicutes;Clostridia;Clostridiales;Lachnospiraceae;PAC001270_g;PAC001899_s group | 6 | 0.027 |
| Clostridium_g21_uc | Bacteria;Firmicutes;Clostridia;Clostridiales;Lachnospiraceae;Clostridium_g21;Clostridium_g21_uc | 6 | 0.027 |
| PAC001092_g_uc | Bacteria;Firmicutes;Clostridia;Clostridiales;Lachnospiraceae;PAC001092_g;PAC001092_g_uc | 6 | 0.027 |
| PAC001103_g_uc | Bacteria;Firmicutes;Clostridia;Clostridiales;Lachnospiraceae;PAC001103_g;PAC001103_g_uc | 6 | 0.027 |
| Bacteroides intestinalis | Bacteria;Bacteroidetes;Bacteroidia;Bacteroidales;Bacteroidaceae;Bacteroides;Bacteroides intestinalis | 5 | 0.0225 |
| PAC000661_g AB185622_s | Bacteria;Firmicutes;Clostridia;Clostridiales;Ruminococcaceae;PAC000661_g;AB185622_s | 5 | 0.0225 |
| KE159538_g EF097676_s | Bacteria;Firmicutes;Clostridia;Clostridiales;Lachnospiraceae;KE159538_g;EF097676_s | 5 | 0.0225 |
| Oscillibacter EU454374_s | Bacteria;Firmicutes;Clostridia;Clostridiales;Ruminococcaceae;Oscillibacter;EU454374_s | 5 | 0.0225 |
| PAC001063_g PAC001063_s | Bacteria;Bacteroidetes;Bacteroidia;Bacteroidales;Muribaculaceae;PAC001063_g;PAC001063_s | 5 | 0.0225 |
| PAC000186_g PAC001065_s | Bacteria;Bacteroidetes;Bacteroidia;Bacteroidales;Muribaculaceae;PAC000186_g;PAC001065_s | 5 | 0.0225 |
| PAC000197_g PAC001123_s | Bacteria;Tenericutes;Mollicutes;PAC001057_o;PAC000197_f;PAC000197_g;PAC001123_s | 5 | 0.0225 |
| PAC001201_g PAC001201_s group | Bacteria;Firmicutes;Clostridia;Clostridiales;Lachnospiraceae;PAC001201_g;PAC001201_s group | 5 | 0.0225 |
| PAC000664_g PAC001226_s | Bacteria;Firmicutes;Clostridia;Clostridiales;Lachnospiraceae;PAC000664_g;PAC001226_s | 5 | 0.0225 |
| PAC001362_g PAC001362_s | Bacteria;Firmicutes;Erysipelotrichi;Erysipelotrichales;Erysipelotrichaceae;PAC001362_g;PAC001362_s | 5 | 0.0225 |
| Acetatifactor PAC001373_s | Bacteria;Firmicutes;Clostridia;Clostridiales;Lachnospiraceae;Acetatifactor;PAC001373_s | 5 | 0.0225 |
| PAC000748_g PAC001496_s | Bacteria;Firmicutes;Clostridia;Clostridiales;Ruminococcaceae;PAC000748_g;PAC001496_s | 5 | 0.0225 |
| PAC001092_g PAC001517_s | Bacteria;Firmicutes;Clostridia;Clostridiales;Lachnospiraceae;PAC001092_g;PAC001517_s | 5 | 0.0225 |
| PAC001528_g PAC001528_s | Bacteria;Firmicutes;Erysipelotrichi;Erysipelotrichales;Erysipelotrichaceae;PAC001528_g;PAC001528_s | 5 | 0.0225 |
| Anaerotruncus PAC001590_s | Bacteria;Firmicutes;Clostridia;Clostridiales;Ruminococcaceae;Anaerotruncus;PAC001590_s | 5 | 0.0225 |
| PAC001609_g PAC001650_s | Bacteria;Firmicutes;Clostridia;Clostridiales;Mogibacterium_f;PAC001609_g;PAC001650_s | 5 | 0.0225 |
| PAC001573_g PAC001808_s | Bacteria;Firmicutes;Clostridia;Clostridiales;Mogibacterium_f;PAC001573_g;PAC001808_s | 5 | 0.0225 |
| Alistipes finegoldii | Bacteria;Bacteroidetes;Bacteroidia;Bacteroidales;Rikenellaceae;Alistipes;Alistipes finegoldii | 4 | 0.018 |
| PAC000692_g EU505164_s | Bacteria;Firmicutes;Clostridia;Clostridiales;Lachnospiraceae;PAC000692_g;EU505164_s | 4 | 0.018 |
| Christensenella EU508399_s | Bacteria;Firmicutes;Clostridia;Clostridiales;Christensenellaceae;Christensenella;EU508399_s | 4 | 0.018 |
| PAC001308_g EU511827_s | Bacteria;Firmicutes;Clostridia;Clostridiales;Lachnospiraceae;PAC001308_g;EU511827_s | 4 | 0.018 |
| Caproiciproducens EU622658_s | Bacteria;Firmicutes;Clostridia;Clostridiales;Ruminococcaceae;Caproiciproducens;EU622658_s | 4 | 0.018 |
| KE159571_g KE159641_s | Bacteria;Firmicutes;Clostridia;Clostridiales;Lachnospiraceae;KE159571_g;KE159641_s | 4 | 0.018 |
| Frisingicoccus PAC000700_s | Bacteria;Firmicutes;Clostridia;Clostridiales;Lachnospiraceae;Frisingicoccus;PAC000700_s | 4 | 0.018 |
| PAC000186_g PAC001128_s | Bacteria;Bacteroidetes;Bacteroidia;Bacteroidales;Muribaculaceae;PAC000186_g;PAC001128_s | 4 | 0.018 |
| Emergencia PAC001235_s | Bacteria;Firmicutes;Clostridia;Clostridiales;Mogibacterium_f;Emergencia;PAC001235_s | 4 | 0.018 |
| PAC001360_g PAC001371_s | Bacteria;Firmicutes;Clostridia;Clostridiales;Christensenellaceae;PAC001360_g;PAC001371_s | 4 | 0.018 |
| PAC000671_g PAC001473_s | Bacteria;Firmicutes;Clostridia;Clostridiales;Lachnospiraceae;PAC000671_g;PAC001473_s | 4 | 0.018 |
| Frisingicoccus PAC001481_s | Bacteria;Firmicutes;Clostridia;Clostridiales;Lachnospiraceae;Frisingicoccus;PAC001481_s | 4 | 0.018 |
| PAC001588_g PAC001588_s | Bacteria;Firmicutes;Clostridia;Clostridiales;Lachnospiraceae;PAC001588_g;PAC001588_s | 4 | 0.018 |
| PAC001612_g PAC001612_s | Bacteria;Firmicutes;Clostridia;Clostridiales;Mogibacterium_f;PAC001612_g;PAC001612_s | 4 | 0.018 |
| Sporobacter PAC001659_s | Bacteria;Firmicutes;Clostridia;Clostridiales;Ruminococcaceae;Sporobacter;PAC001659_s | 4 | 0.018 |
| PAC001103_g PAC001724_s group | Bacteria;Firmicutes;Clostridia;Clostridiales;Lachnospiraceae;PAC001103_g;PAC001724_s group | 4 | 0.018 |
| PAC001068_g PAC001751_s | Bacteria;Bacteroidetes;Bacteroidia;Bacteroidales;Muribaculaceae;PAC001068_g;PAC001751_s | 4 | 0.018 |
| PAC000664_g PAC001769_s | Bacteria;Firmicutes;Clostridia;Clostridiales;Lachnospiraceae;PAC000664_g;PAC001769_s | 4 | 0.018 |
| PAC000661_g PAC002012_s | Bacteria;Firmicutes;Clostridia;Clostridiales;Ruminococcaceae;PAC000661_g;PAC002012_s | 4 | 0.018 |
| PAC001199_g PAC002391_s | Bacteria;Firmicutes;Clostridia;Clostridiales;Lachnospiraceae;PAC001199_g;PAC002391_s | 4 | 0.018 |
| PAC002460_g PAC002460_s | Bacteria;Firmicutes;Clostridia;Clostridiales;Lachnospiraceae;PAC002460_g;PAC002460_s | 4 | 0.018 |
| PAC000186_g_uc | Bacteria;Bacteroidetes;Bacteroidia;Bacteroidales;Muribaculaceae;PAC000186_g;PAC000186_g_uc | 4 | 0.018 |
| PAC000664_g_uc | Bacteria;Firmicutes;Clostridia;Clostridiales;Lachnospiraceae;PAC000664_g;PAC000664_g_uc | 4 | 0.018 |
| PAC001544_g_uc | Bacteria;Firmicutes;Clostridia;Clostridiales;Lachnospiraceae;PAC001544_g;PAC001544_g_uc | 4 | 0.018 |
| PAC002482_g_uc | Bacteria;Bacteroidetes;Bacteroidia;Bacteroidales;AC160630_f;PAC002482_g;PAC002482_g_uc | 4 | 0.018 |
| Bacteroides caecimuris | Bacteria;Bacteroidetes;Bacteroidia;Bacteroidales;Bacteroidaceae;Bacteroides;Bacteroides caecimuris | 3 | 0.0135 |
| Blautia hansenii group | Bacteria;Firmicutes;Clostridia;Clostridiales;Lachnospiraceae;Blautia;Blautia hansenii group | 3 | 0.0135 |
| Clostridium symbiosum | Bacteria;Firmicutes;Clostridia;Clostridiales;Lachnospiraceae;Clostridium_g35;Clostridium symbiosum | 3 | 0.0135 |
| Faecalibacterium prausnitzii group | Bacteria;Firmicutes;Clostridia;Clostridiales;Ruminococcaceae;Faecalibacterium;Faecalibacterium prausnitzii group | 3 | 0.0135 |
| Streptococcus danieliae | Bacteria;Firmicutes;Bacilli;Lactobacillales;Streptococcaceae;Streptococcus;Streptococcus danieliae | 3 | 0.0135 |
| Pseudoflavonifractor EU454100_s | Bacteria;Firmicutes;Clostridia;Clostridiales;Ruminococcaceae;Pseudoflavonifractor;EU454100_s | 3 | 0.0135 |
| EU461719_g EU461719_s | Bacteria;Firmicutes;Clostridia;Clostridiales;Lachnospiraceae;EU461719_g;EU461719_s | 3 | 0.0135 |
| KE159600_g EU511112_s | Bacteria;Firmicutes;Clostridia;Clostridiales;Lachnospiraceae;KE159600_g;EU511112_s | 3 | 0.0135 |
| PAC000664_g PAC001083_s | Bacteria;Firmicutes;Clostridia;Clostridiales;Lachnospiraceae;PAC000664_g;PAC001083_s | 3 | 0.0135 |
| PAC001116_g PAC001116_s | Bacteria;Firmicutes;Clostridia;Clostridiales;Lachnospiraceae;PAC001116_g;PAC001116_s | 3 | 0.0135 |
| PAC001313_g PAC001313_s | Bacteria;Firmicutes;Clostridia;Clostridiales;Ruminococcaceae;PAC001313_g;PAC001313_s | 3 | 0.0135 |
| Oscillibacter PAC001368_s | Bacteria;Firmicutes;Clostridia;Clostridiales;Ruminococcaceae;Oscillibacter;PAC001368_s | 3 | 0.0135 |
| PAC001386_g PAC001386_s | Bacteria;Firmicutes;Clostridia;Clostridiales;Lachnospiraceae;PAC001386_g;PAC001386_s | 3 | 0.0135 |
| PAC001149_g PAC001511_s | Bacteria;Firmicutes;Clostridia;Clostridiales;Lachnospiraceae;PAC001149_g;PAC001511_s | 3 | 0.0135 |
| PAC000692_g PAC001666_s | Bacteria;Firmicutes;Clostridia;Clostridiales;Lachnospiraceae;PAC000692_g;PAC001666_s | 3 | 0.0135 |
| Agathobaculum PAC001688_s | Bacteria;Firmicutes;Clostridia;Clostridiales;Ruminococcaceae;Agathobaculum;PAC001688_s | 3 | 0.0135 |
| PAC000664_g PAC002004_s group | Bacteria;Firmicutes;Clostridia;Clostridiales;Lachnospiraceae;PAC000664_g;PAC002004_s group | 3 | 0.0135 |
| PAC002196_g PAC002196_s | Bacteria;Firmicutes;Clostridia;Clostridiales;Lachnospiraceae;PAC002196_g;PAC002196_s | 3 | 0.0135 |
| PAC002471_g PAC002471_s | Bacteria;Firmicutes;Clostridia;Clostridiales;Lachnospiraceae;PAC002471_g;PAC002471_s | 3 | 0.0135 |
| PAC000692_g_uc | Bacteria;Firmicutes;Clostridia;Clostridiales;Lachnospiraceae;PAC000692_g;PAC000692_g_uc | 3 | 0.0135 |
| PAC000197_f_uc | Bacteria;Tenericutes;Mollicutes;PAC001057_o;PAC000197_f;PAC000197_f_uc | 2 | 0.009 |
| Agathobacter rectalis | Bacteria;Firmicutes;Clostridia;Clostridiales;Lachnospiraceae;Agathobacter;Agathobacter rectalis | 2 | 0.009 |
| Alistipes shahii | Bacteria;Bacteroidetes;Bacteroidia;Bacteroidales;Rikenellaceae;Alistipes;Alistipes shahii | 2 | 0.009 |
| Bacteroides oleiciplenus | Bacteria;Bacteroidetes;Bacteroidia;Bacteroidales;Bacteroidaceae;Bacteroides;Bacteroides oleiciplenus | 2 | 0.009 |
| Bacteroides thetaiotaomicron | Bacteria;Bacteroidetes;Bacteroidia;Bacteroidales;Bacteroidaceae;Bacteroides;Bacteroides thetaiotaomicron | 2 | 0.009 |
| Clostridium innocuum group | Bacteria;Firmicutes;Erysipelotrichi;Erysipelotrichales;Erysipelotrichaceae;Longicatena;Clostridium innocuum group | 2 | 0.009 |
| Agathobaculum AB606336_s | Bacteria;Firmicutes;Clostridia;Clostridiales;Ruminococcaceae;Agathobaculum;AB606336_s | 2 | 0.009 |
| Pseudoflavonifractor ADDX_s | Bacteria;Firmicutes;Clostridia;Clostridiales;Ruminococcaceae;Pseudoflavonifractor;ADDX_s | 2 | 0.009 |
| PAC000661_g EF445156_s | Bacteria;Firmicutes;Clostridia;Clostridiales;Ruminococcaceae;PAC000661_g;EF445156_s | 2 | 0.009 |
| Acetatifactor EU507515_s | Bacteria;Firmicutes;Clostridia;Clostridiales;Lachnospiraceae;Acetatifactor;EU507515_s | 2 | 0.009 |
| Acetatifactor EU508111_s | Bacteria;Firmicutes;Clostridia;Clostridiales;Lachnospiraceae;Acetatifactor;EU508111_s | 2 | 0.009 |
| Pseudoflavonifractor FJ879818_s | Bacteria;Firmicutes;Clostridia;Clostridiales;Ruminococcaceae;Pseudoflavonifractor;FJ879818_s | 2 | 0.009 |
| Eubacterium_g17 PAC001119_s | Bacteria;Firmicutes;Clostridia;Clostridiales;Lachnospiraceae;Eubacterium_g17;PAC001119_s | 2 | 0.009 |
| PAC000198_g PAC001139_s | Bacteria;Bacteroidetes;Bacteroidia;Bacteroidales;Muribaculaceae;PAC000198_g;PAC001139_s | 2 | 0.009 |
| Christensenella PAC001140_s | Bacteria;Firmicutes;Clostridia;Clostridiales;Christensenellaceae;Christensenella;PAC001140_s | 2 | 0.009 |
| PAC001225_g PAC001225_s | Bacteria;Firmicutes;Clostridia;Clostridiales;Lachnospiraceae;PAC001225_g;PAC001225_s | 2 | 0.009 |
| Eubacterium_g6 PAC001227_s | Bacteria;Firmicutes;Clostridia;Clostridiales;Lachnospiraceae;Eubacterium_g6;PAC001227_s | 2 | 0.009 |
| Monoglobus PAC001245_s | Bacteria;Firmicutes;Clostridia;Clostridiales;Ruminococcaceae;Monoglobus;PAC001245_s | 2 | 0.009 |
| PAC001323_g PAC001323_s | Bacteria;Tenericutes;Mollicutes;PAC001057_o;PAC000197_f;PAC001323_g;PAC001323_s | 2 | 0.009 |
| PAC000748_g PAC001489_s | Bacteria;Firmicutes;Clostridia;Clostridiales;Ruminococcaceae;PAC000748_g;PAC001489_s | 2 | 0.009 |
| PAC000664_g PAC001515_s | Bacteria;Firmicutes;Clostridia;Clostridiales;Lachnospiraceae;PAC000664_g;PAC001515_s | 2 | 0.009 |
| PAC000661_g PAC001536_s | Bacteria;Firmicutes;Clostridia;Clostridiales;Ruminococcaceae;PAC000661_g;PAC001536_s | 2 | 0.009 |
| PAC001092_g PAC001587_s | Bacteria;Firmicutes;Clostridia;Clostridiales;Lachnospiraceae;PAC001092_g;PAC001587_s | 2 | 0.009 |
| Pseudoflavonifractor PAC001608_s | Bacteria;Firmicutes;Clostridia;Clostridiales;Ruminococcaceae;Pseudoflavonifractor;PAC001608_s | 2 | 0.009 |
| Oscillibacter PAC001706_s | Bacteria;Firmicutes;Clostridia;Clostridiales;Ruminococcaceae;Oscillibacter;PAC001706_s | 2 | 0.009 |
| PAC001360_g PAC001743_s | Bacteria;Firmicutes;Clostridia;Clostridiales;Christensenellaceae;PAC001360_g;PAC001743_s | 2 | 0.009 |
| PAC001068_g PAC001766_s | Bacteria;Bacteroidetes;Bacteroidia;Bacteroidales;Muribaculaceae;PAC001068_g;PAC001766_s | 2 | 0.009 |
| PAC001138_g PAC001775_s | Bacteria;Firmicutes;Clostridia;Clostridiales;Lachnospiraceae;PAC001138_g;PAC001775_s | 2 | 0.009 |
| PAC001199_g PAC001781_s | Bacteria;Firmicutes;Clostridia;Clostridiales;Lachnospiraceae;PAC001199_g;PAC001781_s | 2 | 0.009 |
| PAC001225_g PAC002005_s | Bacteria;Firmicutes;Clostridia;Clostridiales;Lachnospiraceae;PAC001225_g;PAC002005_s | 2 | 0.009 |
| PAC001092_g PAC002030_s | Bacteria;Firmicutes;Clostridia;Clostridiales;Lachnospiraceae;PAC001092_g;PAC002030_s | 2 | 0.009 |
| PAC002042_g PAC002042_s | Bacteria;Firmicutes;Clostridia;Clostridiales;Lachnospiraceae;PAC002042_g;PAC002042_s | 2 | 0.009 |
| Christensenella PAC002165_s | Bacteria;Firmicutes;Clostridia;Clostridiales;Christensenellaceae;Christensenella;PAC002165_s | 2 | 0.009 |
| Pseudoflavonifractor PAC002302_s | Bacteria;Firmicutes;Clostridia;Clostridiales;Ruminococcaceae;Pseudoflavonifractor;PAC002302_s | 2 | 0.009 |
| Acetatifactor PAC002333_s | Bacteria;Firmicutes;Clostridia;Clostridiales;Lachnospiraceae;Acetatifactor;PAC002333_s | 2 | 0.009 |
| Acutalibacter PAC002354_s | Bacteria;Firmicutes;Clostridia;Clostridiales;Ruminococcaceae;Acutalibacter;PAC002354_s | 2 | 0.009 |
| Frisingicoccus PAC002470_s | Bacteria;Firmicutes;Clostridia;Clostridiales;Lachnospiraceae;Frisingicoccus;PAC002470_s | 2 | 0.009 |
| Parasutterella PAC002476_s | Bacteria;Proteobacteria;Betaproteobacteria;Burkholderiales;Sutterellaceae;Parasutterella;PAC002476_s | 2 | 0.009 |
| Bilophila_uc | Bacteria;Proteobacteria;Deltaproteobacteria;Desulfovibrionales;Desulfovibrionaceae;Bilophila;Bilophila_uc | 2 | 0.009 |
| Lactococcus_uc | Bacteria;Firmicutes;Bacilli;Lactobacillales;Streptococcaceae;Lactococcus;Lactococcus_uc | 2 | 0.009 |
| Parabacteroides_uc | Bacteria;Bacteroidetes;Bacteroidia;Bacteroidales;Porphyromonadaceae;Parabacteroides;Parabacteroides_uc | 2 | 0.009 |
| Clostridium_g35_uc | Bacteria;Firmicutes;Clostridia;Clostridiales;Lachnospiraceae;Clostridium_g35;Clostridium_g35_uc | 2 | 0.009 |
| PAC000196_g_uc | Bacteria;Firmicutes;Clostridia;Clostridiales;Lachnospiraceae;PAC000196_g;PAC000196_g_uc | 2 | 0.009 |
| PAC001219_g_uc | Bacteria;Firmicutes;Clostridia;Clostridiales;Christensenellaceae;PAC001219_g;PAC001219_g_uc | 2 | 0.009 |
| PAC001296_g_uc | Bacteria;Firmicutes;Clostridia;Clostridiales;Lachnospiraceae;PAC001296_g;PAC001296_g_uc | 2 | 0.009 |
| PAC001651_g_uc | Bacteria;Firmicutes;Clostridia;Clostridiales;Ruminococcaceae;PAC001651_g;PAC001651_g_uc | 2 | 0.009 |
| Bacillus cereus group | Bacteria;Firmicutes;Bacilli;Bacillales;Bacillaceae;Bacillus;Bacillus cereus group | 1 | 0.0045 |
| Bacillus subtilis group | Bacteria;Firmicutes;Bacilli;Bacillales;Bacillaceae;Bacillus;Bacillus subtilis group | 1 | 0.0045 |
| Bacteroides ovatus group | Bacteria;Bacteroidetes;Bacteroidia;Bacteroidales;Bacteroidaceae;Bacteroides;Bacteroides ovatus group | 1 | 0.0045 |
| Cloacibacterium normanense | Bacteria;Bacteroidetes;Flavobacteria;Flavobacteriales;Flavobacteriaceae;Cloacibacterium;Cloacibacterium normanense | 1 | 0.0045 |
| Clostridium scindens | Bacteria;Firmicutes;Clostridia;Clostridiales;Lachnospiraceae;Clostridium_g21;Clostridium scindens | 1 | 0.0045 |
| Clostridium tertium group | Bacteria;Firmicutes;Clostridia;Clostridiales;Clostridiaceae;Clostridium;Clostridium tertium group | 1 | 0.0045 |
| Enterococcus faecium group | Bacteria;Firmicutes;Bacilli;Lactobacillales;Enterococcaceae;Enterococcus;Enterococcus faecium group | 1 | 0.0045 |
| Flavobacterium lutivivi | Bacteria;Bacteroidetes;Flavobacteria;Flavobacteriales;Flavobacteriaceae;Flavobacterium;Flavobacterium lutivivi | 1 | 0.0045 |
| Haemophilus parainfluenzae group | Bacteria;Proteobacteria;Gammaproteobacteria;Pasteurellales;Pasteurellaceae;Haemophilus;Haemophilus parainfluenzae group | 1 | 0.0045 |
| Leifsonia poae group | Bacteria;Actinobacteria;Actinobacteria_c;Micrococcales;Microbacteriaceae;Leifsonia;Leifsonia poae group | 1 | 0.0045 |
| Lysinibacillus xylanilyticus group | Bacteria;Firmicutes;Bacilli;Bacillales;Planococcaceae;Lysinibacillus;Lysinibacillus xylanilyticus group | 1 | 0.0045 |
| Mycoplasma sualvi | Bacteria;Tenericutes;Mollicutes;Mycoplasmatales;Mycoplasmataceae;Mycoplasma_g10;Mycoplasma sualvi | 1 | 0.0045 |
| Nakamurella lactea | Bacteria;Actinobacteria;Actinobacteria_c;Frankiales;Nakamurellaceae;Nakamurella;Nakamurella lactea | 1 | 0.0045 |
| Neisseria perflava | Bacteria;Proteobacteria;Betaproteobacteria;Neisseriales;Neisseriaceae;Neisseria;Neisseria perflava | 1 | 0.0045 |
| Paenibacillus residui | Bacteria;Firmicutes;Bacilli;Bacillales;Paenibacillaceae;Paenibacillus;Paenibacillus residui | 1 | 0.0045 |
| Parabacteroides merdae | Bacteria;Bacteroidetes;Bacteroidia;Bacteroidales;Porphyromonadaceae;Parabacteroides;Parabacteroides merdae | 1 | 0.0045 |
| Parafilimonas terrae | Bacteria;Bacteroidetes;Sphingobacteriia;Sphingobacteriales;Chitinophagaceae;Parafilimonas;Parafilimonas terrae | 1 | 0.0045 |
| Rhodococcus erythropolis group | Bacteria;Actinobacteria;Actinobacteria_c;Corynebacteriales;Nocardiaceae;Rhodococcus;Rhodococcus erythropolis group | 1 | 0.0045 |
| Romboutsia timonensis | Bacteria;Firmicutes;Clostridia;Clostridiales;Peptostreptococcaceae;Romboutsia;Romboutsia timonensis | 1 | 0.0045 |
| Sphingomonas lutea group | Bacteria;Proteobacteria;Alphaproteobacteria;Sphingomonadales;Sphingomonadaceae;Sphingomonas;Sphingomonas lutea group | 1 | 0.0045 |
| Sphingomonas palustris | Bacteria;Proteobacteria;Alphaproteobacteria;Sphingomonadales;Sphingomonadaceae;Sphingomonas;Sphingomonas palustris | 1 | 0.0045 |
| Streptococcus acidominimus group | Bacteria;Firmicutes;Bacilli;Lactobacillales;Streptococcaceae;Streptococcus;Streptococcus acidominimus group | 1 | 0.0045 |
| Streptococcus salivarius group | Bacteria;Firmicutes;Bacilli;Lactobacillales;Streptococcaceae;Streptococcus;Streptococcus salivarius group | 1 | 0.0045 |
| PAC000661_g AM277340_s | Bacteria;Firmicutes;Clostridia;Clostridiales;Ruminococcaceae;PAC000661_g;AM277340_s | 1 | 0.0045 |
| AP011765_g CU466780_s | Bacteria;Proteobacteria;Deltaproteobacteria;Myxococcales;Polyangiaceae;AP011765_g;CU466780_s | 1 | 0.0045 |
| Muribaculum DQ815542_s | Bacteria;Bacteroidetes;Bacteroidia;Bacteroidales;Muribaculaceae;Muribaculum;DQ815542_s | 1 | 0.0045 |
| KE159600_g EF098562_s | Bacteria;Firmicutes;Clostridia;Clostridiales;Lachnospiraceae;KE159600_g;EF098562_s | 1 | 0.0045 |
| Clostridium_g21 EU455375_s | Bacteria;Firmicutes;Clostridia;Clostridiales;Lachnospiraceae;Clostridium_g21;EU455375_s | 1 | 0.0045 |
| PAC000196_g EU466069_s | Bacteria;Firmicutes;Clostridia;Clostridiales;Lachnospiraceae;PAC000196_g;EU466069_s | 1 | 0.0045 |
| GQ451199_g EU472543_s | Bacteria;Firmicutes;Clostridia;Clostridiales;Ruminococcaceae;GQ451199_g;EU472543_s | 1 | 0.0045 |
| PAC001225_g EU504346_s | Bacteria;Firmicutes;Clostridia;Clostridiales;Lachnospiraceae;PAC001225_g;EU504346_s | 1 | 0.0045 |
| PAC001573_g EU509323_s | Bacteria;Firmicutes;Clostridia;Clostridiales;Mogibacterium_f;PAC001573_g;EU509323_s | 1 | 0.0045 |
| PAC001063_g EU510197_s | Bacteria;Bacteroidetes;Bacteroidia;Bacteroidales;Muribaculaceae;PAC001063_g;EU510197_s | 1 | 0.0045 |
| Pseudoflavonifractor FJ879530_s | Bacteria;Firmicutes;Clostridia;Clostridiales;Ruminococcaceae;Pseudoflavonifractor;FJ879530_s | 1 | 0.0045 |
| HQ158649_g GU454872_s | Bacteria;Bacteroidetes;Sphingobacteriia;Saprospirales;Saprospiraceae;HQ158649_g;GU454872_s | 1 | 0.0045 |
| Terrimonas GU454926_s | Bacteria;Bacteroidetes;Sphingobacteriia;Sphingobacteriales;Chitinophagaceae;Terrimonas;GU454926_s | 1 | 0.0045 |
| HM036033_g HM036033_s | Bacteria;Firmicutes;Bacilli;Bacillales;Thermoactinomycetaceae;HM036033_g;HM036033_s | 1 | 0.0045 |
| Pseudoflavonifractor HQ716472_s | Bacteria;Firmicutes;Clostridia;Clostridiales;Ruminococcaceae;Pseudoflavonifractor;HQ716472_s | 1 | 0.0045 |
| PAC000244_g JF215608_s | Bacteria;Proteobacteria;Deltaproteobacteria;Myxococcales;Polyangiaceae;PAC000244_g;JF215608_s | 1 | 0.0045 |
| DQ129389_g JQ624352_s | Bacteria;Chloroflexi;Thermomicrobia;DQ129389_o;DQ129389_f;DQ129389_g;JQ624352_s | 1 | 0.0045 |
| LARJ_g PAC000185_s | Bacteria;Proteobacteria;Alphaproteobacteria;Rhodospirillales;Rhodospirillaceae;LARJ_g;PAC000185_s | 1 | 0.0045 |
| PAC001062_g PAC001062_s | Bacteria;Bacteroidetes;Bacteroidia;Bacteroidales;Muribaculaceae;PAC001062_g;PAC001062_s | 1 | 0.0045 |
| PAC001068_g PAC001069_s | Bacteria;Bacteroidetes;Bacteroidia;Bacteroidales;Muribaculaceae;PAC001068_g;PAC001069_s | 1 | 0.0045 |
| Pseudoflavonifractor PAC001088_s | Bacteria;Firmicutes;Clostridia;Clostridiales;Ruminococcaceae;Pseudoflavonifractor;PAC001088_s | 1 | 0.0045 |
| Paludicola PAC001106_s | Bacteria;Firmicutes;Clostridia;Clostridiales;Ruminococcaceae;Paludicola;PAC001106_s | 1 | 0.0045 |
| Pseudoflavonifractor PAC001131_s | Bacteria;Firmicutes;Clostridia;Clostridiales;Ruminococcaceae;Pseudoflavonifractor;PAC001131_s | 1 | 0.0045 |
| PAC001108_g PAC001193_s | Bacteria;Tenericutes;Mollicutes;PAC001057_o;PAC001057_f;PAC001108_g;PAC001193_s | 1 | 0.0045 |
| Prevotella PAC001304_s | Bacteria;Bacteroidetes;Bacteroidia;Bacteroidales;Prevotellaceae;Prevotella;PAC001304_s | 1 | 0.0045 |
| Pseudoflavonifractor PAC001318_s | Bacteria;Firmicutes;Clostridia;Clostridiales;Ruminococcaceae;Pseudoflavonifractor;PAC001318_s | 1 | 0.0045 |
| Frisingicoccus PAC001364_s | Bacteria;Firmicutes;Clostridia;Clostridiales;Lachnospiraceae;Frisingicoccus;PAC001364_s | 1 | 0.0045 |
| PAC001375_g PAC001375_s | Bacteria;Firmicutes;Clostridia;Clostridiales;Lachnospiraceae;PAC001375_g;PAC001375_s | 1 | 0.0045 |
| PAC001092_g PAC001388_s | Bacteria;Firmicutes;Clostridia;Clostridiales;Lachnospiraceae;PAC001092_g;PAC001388_s | 1 | 0.0045 |
| PAC001456_g PAC001456_s | Bacteria;Firmicutes;Clostridia;Clostridiales;Lachnospiraceae;PAC001456_g;PAC001456_s | 1 | 0.0045 |
| Anaerotignum PAC001464_s | Bacteria;Firmicutes;Clostridia;Clostridiales;Lachnospiraceae;Anaerotignum;PAC001464_s | 1 | 0.0045 |
| PAC001103_g PAC001487_s | Bacteria;Firmicutes;Clostridia;Clostridiales;Lachnospiraceae;PAC001103_g;PAC001487_s | 1 | 0.0045 |
| Eubacterium_g23 PAC001490_s | Bacteria;Firmicutes;Clostridia;Clostridiales;Ruminococcaceae;Eubacterium_g23;PAC001490_s | 1 | 0.0045 |
| PAC001512_g PAC001512_s | Bacteria;Bacteroidetes;Bacteroidia;Bacteroidales;Muribaculaceae;PAC001512_g;PAC001512_s | 1 | 0.0045 |
| Acutalibacter PAC001519_s | Bacteria;Firmicutes;Clostridia;Clostridiales;Ruminococcaceae;Acutalibacter;PAC001519_s | 1 | 0.0045 |
| PAC001525_g PAC001525_s | Bacteria;Firmicutes;Clostridia;Clostridiales;Lachnospiraceae;PAC001525_g;PAC001525_s | 1 | 0.0045 |
| Pseudoflavonifractor PAC001542_s | Bacteria;Firmicutes;Clostridia;Clostridiales;Ruminococcaceae;Pseudoflavonifractor;PAC001542_s | 1 | 0.0045 |
| Oscillibacter PAC001551_s | Bacteria;Firmicutes;Clostridia;Clostridiales;Ruminococcaceae;Oscillibacter;PAC001551_s | 1 | 0.0045 |
| KE159571_g PAC001559_s | Bacteria;Firmicutes;Clostridia;Clostridiales;Lachnospiraceae;KE159571_g;PAC001559_s | 1 | 0.0045 |
| PAC000692_g PAC001577_s | Bacteria;Firmicutes;Clostridia;Clostridiales;Lachnospiraceae;PAC000692_g;PAC001577_s | 1 | 0.0045 |
| PAC001599_g PAC001599_s | Bacteria;Firmicutes;Clostridia;Clostridiales;Ruminococcaceae;PAC001599_g;PAC001599_s | 1 | 0.0045 |
| PAC001609_g PAC001610_s | Bacteria;Firmicutes;Clostridia;Clostridiales;Mogibacterium_f;PAC001609_g;PAC001610_s | 1 | 0.0045 |
| PAC001681_g PAC001681_s | Bacteria;Firmicutes;Clostridia;Clostridiales;Lachnospiraceae;PAC001681_g;PAC001681_s | 1 | 0.0045 |
| PAC001092_g PAC001691_s | Bacteria;Firmicutes;Clostridia;Clostridiales;Lachnospiraceae;PAC001092_g;PAC001691_s | 1 | 0.0045 |
| LLKB_g PAC001694_s | Bacteria;Firmicutes;Clostridia;Clostridiales;Lachnospiraceae;LLKB_g;PAC001694_s | 1 | 0.0045 |
| KE159538_g PAC001696_s | Bacteria;Firmicutes;Clostridia;Clostridiales;Lachnospiraceae;KE159538_g;PAC001696_s | 1 | 0.0045 |
| PAC001043_g PAC001752_s | Bacteria;Firmicutes;Clostridia;Clostridiales;Lachnospiraceae;PAC001043_g;PAC001752_s | 1 | 0.0045 |
| Marvinbryantia PAC001753_s | Bacteria;Firmicutes;Clostridia;Clostridiales;Lachnospiraceae;Marvinbryantia;PAC001753_s | 1 | 0.0045 |
| Clostridium_g21 PAC001757_s group | Bacteria;Firmicutes;Clostridia;Clostridiales;Lachnospiraceae;Clostridium_g21;PAC001757_s group | 1 | 0.0045 |
| Pseudoflavonifractor PAC001761_s | Bacteria;Firmicutes;Clostridia;Clostridiales;Ruminococcaceae;Pseudoflavonifractor;PAC001761_s | 1 | 0.0045 |
| PAC001765_g PAC001765_s | Bacteria;Bacteroidetes;Bacteroidia;Bacteroidales;Muribaculaceae;PAC001765_g;PAC001765_s | 1 | 0.0045 |
| PAC001236_g PAC001767_s | Bacteria;Firmicutes;Clostridia;Clostridiales;Mogibacterium_f;PAC001236_g;PAC001767_s | 1 | 0.0045 |
| PAC001212_g PAC001809_s | Bacteria;Firmicutes;Clostridia;Clostridiales;Lachnospiraceae;PAC001212_g;PAC001809_s | 1 | 0.0045 |
| PAC001778_g PAC001909_s | Bacteria;Firmicutes;Clostridia;Clostridiales;Ruminococcaceae;PAC001778_g;PAC001909_s | 1 | 0.0045 |
| Harryflintia PAC002011_s | Bacteria;Firmicutes;Clostridia;Clostridiales;Ruminococcaceae;Harryflintia;PAC002011_s | 1 | 0.0045 |
| PAC002390_g PAC002390_s | Bacteria;Firmicutes;Clostridia;Clostridiales;Dehalobacterium_f;PAC002390_g;PAC002390_s | 1 | 0.0045 |
| PAC001127_g PAC002399_s | Bacteria;Bacteroidetes;Bacteroidia;Bacteroidales;Muribaculaceae;PAC001127_g;PAC002399_s | 1 | 0.0045 |
| KE159605_g PAC002401_s group | Bacteria;Firmicutes;Clostridia;Clostridiales;Lachnospiraceae;KE159605_g;PAC002401_s group | 1 | 0.0045 |
| Enterorhabdus PAC002410_s | Bacteria;Actinobacteria;Coriobacteriia;Coriobacteriales;Coriobacteriaceae;Enterorhabdus;PAC002410_s | 1 | 0.0045 |
| PAC002331_g PAC002430_s | Bacteria;Bacteroidetes;Sphingobacteriia;Sphingobacteriales;Chitinophagaceae;PAC002331_g;PAC002430_s | 1 | 0.0045 |
| PAC001091_g PAC002458_s | Bacteria;Firmicutes;Clostridia;Clostridiales;Lachnospiraceae;PAC001091_g;PAC002458_s | 1 | 0.0045 |
| Pseudoflavonifractor PAC002475_s | Bacteria;Firmicutes;Clostridia;Clostridiales;Ruminococcaceae;Pseudoflavonifractor;PAC002475_s | 1 | 0.0045 |
| PAC000664_g PAC002486_s | Bacteria;Firmicutes;Clostridia;Clostridiales;Lachnospiraceae;PAC000664_g;PAC002486_s | 1 | 0.0045 |
| Pseudoflavonifractor PAC002490_s | Bacteria;Firmicutes;Clostridia;Clostridiales;Ruminococcaceae;Pseudoflavonifractor;PAC002490_s | 1 | 0.0045 |
| PAC000661_g PAC002496_s | Bacteria;Firmicutes;Clostridia;Clostridiales;Ruminococcaceae;PAC000661_g;PAC002496_s | 1 | 0.0045 |
| Oscillibacter_uc | Bacteria;Firmicutes;Clostridia;Clostridiales;Ruminococcaceae;Oscillibacter;Oscillibacter_uc | 1 | 0.0045 |
| Clostridium_g24_uc | Bacteria;Firmicutes;Clostridia;Clostridiales;Lachnospiraceae;Clostridium_g24;Clostridium_g24_uc | 1 | 0.0045 |
| PAC000683_g_uc | Bacteria;Firmicutes;Clostridia;Clostridiales;Ruminococcaceae;PAC000683_g;PAC000683_g_uc | 1 | 0.0045 |

**Set 1_0.5%_S: Day 28_heme-SCP 0.5%_Species**

| Taxon name | Taxonomy | Count | Proportion(%) |
|---|---|---|---|
| Bacteroides HM124113_s | Bacteria;Bacteroidetes;Bacteroidia;Bacteroidales;Bacteroidaceae;Bacteroides;HM124113_s | 2558 | 11.8651 |
| Akkermansia muciniphila | Bacteria;Verrucomicrobia;Verrucomicrobiae;Verrucomicrobiales;Akkermansiaceae;Akkermansia;Akkermansia muciniphila | 2441 | 11.3224 |
| Lactobacillus gasseri group | Bacteria;Firmicutes;Bacilli;Lactobacillales;Lactobacillaceae;Lactobacillus;Lactobacillus gasseri group | 1874 | 8.6924 |
| Lactobacillus reuteri group | Bacteria;Firmicutes;Bacilli;Lactobacillales;Lactobacillaceae;Lactobacillus;Lactobacillus reuteri group | 1106 | 5.1301 |
| Lactobacillus murinus group | Bacteria;Firmicutes;Bacilli;Lactobacillales;Lactobacillaceae;Lactobacillus;Lactobacillus murinus group | 966 | 4.4807 |
| Bacteroides acidifaciens | Bacteria;Bacteroidetes;Bacteroidia;Bacteroidales;Bacteroidaceae;Bacteroides;Bacteroides acidifaciens | 578 | 2.681 |
| KE159600_g KE159600_s | Bacteria;Firmicutes;Clostridia;Clostridiales;Lachnospiraceae;KE159600_g;KE159600_s | 515 | 2.3888 |
| PAC000198_g PAC001075_s | Bacteria;Bacteroidetes;Bacteroidia;Bacteroidales;Muribaculaceae;PAC000198_g;PAC001075_s | 505 | 2.3424 |
| PAC001141_g PAC001141_s | Bacteria;Firmicutes;Clostridia;Clostridiales;Christensenellaceae;PAC001141_g;PAC001141_s | 493 | 2.2867 |
| PAC001118_g PAC002344_s | Bacteria;Firmicutes;Clostridia;Clostridiales;Lachnospiraceae;PAC001118_g;PAC002344_s | 482 | 2.2357 |
| Bacteroides vulgatus | Bacteria;Bacteroidetes;Bacteroidia;Bacteroidales;Bacteroidaceae;Bacteroides;Bacteroides vulgatus | 421 | 1.9528 |
| PAC001074_g PAC001074_s | Bacteria;Bacteroidetes;Bacteroidia;Bacteroidales;Muribaculaceae;PAC001074_g;PAC001074_s | 339 | 1.5724 |
| Corynebacterium glutamicum | Bacteria;Actinobacteria;Actinobacteria_c;Corynebacteriales;Corynebacteriaceae;Corynebacterium;Corynebacterium glutamicum | 307 | 1.424 |
| Alistipes PAC001061_s | Bacteria;Bacteroidetes;Bacteroidia;Bacteroidales;Rikenellaceae;Alistipes;PAC001061_s | 291 | 1.3498 |
| Lactococcus lactis group | Bacteria;Firmicutes;Bacilli;Lactobacillales;Streptococcaceae;Lactococcus;Lactococcus lactis group | 272 | 1.2617 |
| Oscillibacter PAC001186_s group | Bacteria;Firmicutes;Clostridia;Clostridiales;Ruminococcaceae;Oscillibacter;PAC001186_s group | 241 | 1.1179 |
| PAC002482_g EF603735_s | Bacteria;Bacteroidetes;Bacteroidia;Bacteroidales;AC160630_f;PAC002482_g;EF603735_s | 239 | 1.1086 |
| Bilophila wadsworthia | Bacteria;Proteobacteria;Deltaproteobacteria;Desulfovibrionales;Desulfovibrionaceae;Bilophila;Bilophila wadsworthia | 218 | 1.0112 |
| Anaerotignum PAC001740_s | Bacteria;Firmicutes;Clostridia;Clostridiales;Lachnospiraceae;Anaerotignum;PAC001740_s | 217 | 1.0065 |
| Odoribacter DQ815763_s | Bacteria;Bacteroidetes;Bacteroidia;Bacteroidales;Odoribacteraceae;Odoribacter;DQ815763_s | 209 | 0.9694 |
| KE159571_g PAC002009_s group | Bacteria;Firmicutes;Clostridia;Clostridiales;Lachnospiraceae;KE159571_g;PAC002009_s group | 204 | 0.9462 |
| Bacteroides_uc | Bacteria;Bacteroidetes;Bacteroidia;Bacteroidales;Bacteroidaceae;Bacteroides;Bacteroides_uc | 176 | 0.8164 |
| PAC001112_g PAC001267_s | Bacteria;Bacteroidetes;Bacteroidia;Bacteroidales;Muribaculaceae;PAC001112_g;PAC001267_s | 170 | 0.7885 |
| PAC001400_g PAC001400_s | Bacteria;Firmicutes;Clostridia;Clostridiales;Peptococcaceae;PAC001400_g;PAC001400_s | 169 | 0.7839 |
| PAC001738_g PAC001738_s | Bacteria;Firmicutes;Clostridia;Clostridiales;Lachnospiraceae;PAC001738_g;PAC001738_s | 161 | 0.7468 |
| Bacteroides stercorirosoris | Bacteria;Bacteroidetes;Bacteroidia;Bacteroidales;Bacteroidaceae;Bacteroides;Bacteroides stercorirosoris | 160 | 0.7421 |
| Clostridium cocleatum | Bacteria;Firmicutes;Erysipelotrichi;Erysipelotrichales;Erysipelotrichaceae;Clostridium_g6;Clostridium cocleatum | 160 | 0.7421 |
| PAC001516_g PAC002384_s | Bacteria;Firmicutes;Clostridia;Clostridiales;Lachnospiraceae;PAC001516_g;PAC002384_s | 150 | 0.6958 |
| Bacteroides ovatus group | Bacteria;Bacteroidetes;Bacteroidia;Bacteroidales;Bacteroidaceae;Bacteroides;Bacteroides ovatus group | 145 | 0.6726 |
| PAC001452_g PAC001981_s group | Bacteria;Firmicutes;Clostridia;Clostridiales;Lachnospiraceae;PAC001452_g;PAC001981_s group | 138 | 0.6401 |
| Frisingicoccus PAC001744_s group | Bacteria;Firmicutes;Clostridia;Clostridiales;Lachnospiraceae;Frisingicoccus;PAC001744_s group | 132 | 0.6123 |
| PAC000683_g_uc | Bacteria;Firmicutes;Clostridia;Clostridiales;Ruminococcaceae;PAC000683_g;PAC000683_g_uc | 132 | 0.6123 |
| Anaerotruncus KE159677_s | Bacteria;Firmicutes;Clostridia;Clostridiales;Ruminococcaceae;Anaerotruncus;KE159677_s | 124 | 0.5752 |
| Flintibacter butyricus group | Bacteria;Firmicutes;Clostridia;Clostridiales;Ruminococcaceae;Pseudoflavonifractor;Flintibacter butyricus group | 104 | 0.4824 |
| Clostridium_g21 PAC001294_s | Bacteria;Firmicutes;Clostridia;Clostridiales;Lachnospiraceae;Clostridium_g21;PAC001294_s | 101 | 0.4685 |
| PAC000661_g PAC001716_s | Bacteria;Firmicutes;Clostridia;Clostridiales;Ruminococcaceae;PAC000661_g;PAC001716_s | 100 | 0.4638 |
| Oscillibacter PAC001638_s | Bacteria;Firmicutes;Clostridia;Clostridiales;Ruminococcaceae;Oscillibacter;PAC001638_s | 99 | 0.4592 |
| Bacteroides uniformis | Bacteria;Bacteroidetes;Bacteroidia;Bacteroidales;Bacteroidaceae;Bacteroides;Bacteroides uniformis | 92 | 0.4267 |
| Pseudoflavonifractor PAC002397_s | Bacteria;Firmicutes;Clostridia;Clostridiales;Ruminococcaceae;Pseudoflavonifractor;PAC002397_s | 89 | 0.4128 |
| Oscillibacter PAC001501_s | Bacteria;Firmicutes;Clostridia;Clostridiales;Ruminococcaceae;Oscillibacter;PAC001501_s | 87 | 0.4035 |
| KE159538_g PAC001093_s | Bacteria;Firmicutes;Clostridia;Clostridiales;Lachnospiraceae;KE159538_g;PAC001093_s | 86 | 0.3989 |
| Helicobacter japonicus | Bacteria;Proteobacteria;Epsilonproteobacteria;Campylobacterales;Helicobacteraceae;Helicobacter;Helicobacter japonicus | 84 | 0.3896 |
| Alistipes PAC001471_s | Bacteria;Bacteroidetes;Bacteroidia;Bacteroidales;Rikenellaceae;Alistipes;PAC001471_s | 72 | 0.334 |
| Faecalibacterium prausnitzii group | Bacteria;Firmicutes;Clostridia;Clostridiales;Ruminococcaceae;Faecalibacterium;Faecalibacterium prausnitzii group | 69 | 0.3201 |
| PAC001228_g PAC002027_s | Bacteria;Firmicutes;Clostridia;Clostridiales;Lachnospiraceae;PAC001228_g;PAC002027_s | 67 | 0.3108 |
| Sporobacter PAC001096_s | Bacteria;Firmicutes;Clostridia;Clostridiales;Ruminococcaceae;Sporobacter;PAC001096_s | 66 | 0.3061 |
| KE159600_g PAC001232_s | Bacteria;Firmicutes;Clostridia;Clostridiales;Lachnospiraceae;KE159600_g;PAC001232_s | 64 | 0.2969 |
| Clostridium_g21 EU506235_s | Bacteria;Firmicutes;Clostridia;Clostridiales;Lachnospiraceae;Clostridium_g21;EU506235_s | 63 | 0.2922 |
| Paludicola PAC001508_s | Bacteria;Firmicutes;Clostridia;Clostridiales;Ruminococcaceae;Paludicola;PAC001508_s | 63 | 0.2922 |
| PAC001457_g AB606250_s | Bacteria;Firmicutes;Clostridia;Clostridiales;Lachnospiraceae;PAC001457_g;AB606250_s | 58 | 0.269 |
| PAC001402_g PAC001402_s | Bacteria;Firmicutes;Clostridia;Clostridiales;Ruminococcaceae;PAC001402_g;PAC001402_s | 57 | 0.2644 |
| PAC001092_g PAC001710_s | Bacteria;Firmicutes;Clostridia;Clostridiales;Lachnospiraceae;PAC001092_g;PAC001710_s | 57 | 0.2644 |
| PAC001500_g PAC001500_s | Bacteria;Firmicutes;Clostridia;Clostridiales;Peptococcaceae;PAC001500_g;PAC001500_s | 54 | 0.2505 |
| PAC001068_g PAC001068_s | Bacteria;Bacteroidetes;Bacteroidia;Bacteroidales;Muribaculaceae;PAC001068_g;PAC001068_s | 53 | 0.2458 |
| KE159571_g PAC001776_s group | Bacteria;Firmicutes;Clostridia;Clostridiales;Lachnospiraceae;KE159571_g;PAC001776_s group | 52 | 0.2412 |
| Escherichia coli group | Bacteria;Proteobacteria;Gammaproteobacteria;Enterobacterales;Enterobacteriaceae;Escherichia;Escherichia coli group | 51 | 0.2366 |
| PAC000664_g PAC001104_s | Bacteria;Firmicutes;Clostridia;Clostridiales;Lachnospiraceae;PAC000664_g;PAC001104_s | 48 | 0.2226 |
| PAC001402_g PAC001764_s | Bacteria;Firmicutes;Clostridia;Clostridiales;Ruminococcaceae;PAC001402_g;PAC001764_s | 48 | 0.2226 |
| PAC001063_g PAC001063_s | Bacteria;Bacteroidetes;Bacteroidia;Bacteroidales;Muribaculaceae;PAC001063_g;PAC001063_s | 47 | 0.218 |
| Prevotella NOVE_s | Bacteria;Bacteroidetes;Bacteroidia;Bacteroidales;Prevotellaceae;Prevotella;NOVE_s | 46 | 0.2134 |
| Pseudoflavonifractor PAC001543_s | Bacteria;Firmicutes;Clostridia;Clostridiales;Ruminococcaceae;Pseudoflavonifractor;PAC001543_s | 46 | 0.2134 |
| LARJ_g PAC001927_s | Bacteria;Proteobacteria;Alphaproteobacteria;Rhodospirillales;Rhodospirillaceae;LARJ_g;PAC001927_s | 46 | 0.2134 |
| Pseudoflavonifractor PAC001549_s | Bacteria;Firmicutes;Clostridia;Clostridiales;Ruminococcaceae;Pseudoflavonifractor;PAC001549_s | 44 | 0.2041 |
| PAC001360_g PAC001371_s | Bacteria;Firmicutes;Clostridia;Clostridiales;Christensenellaceae;PAC001360_g;PAC001371_s | 42 | 0.1948 |
| Acetatifactor PAC001801_s | Bacteria;Firmicutes;Clostridia;Clostridiales;Lachnospiraceae;Acetatifactor;PAC001801_s | 42 | 0.1948 |
| Bacteroides plebeius | Bacteria;Bacteroidetes;Bacteroidia;Bacteroidales;Bacteroidaceae;Bacteroides;Bacteroides plebeius | 41 | 0.1902 |
| Desulfovibrio PAC001079_s | Bacteria;Proteobacteria;Deltaproteobacteria;Desulfovibrionales;Desulfovibrionaceae;Desulfovibrio;PAC001079_s | 41 | 0.1902 |
| PAC000661_g PAC002496_s | Bacteria;Firmicutes;Clostridia;Clostridiales;Ruminococcaceae;PAC000661_g;PAC002496_s | 41 | 0.1902 |
| PAC000197_g_uc | Bacteria;Tenericutes;Mollicutes;PAC001057_c;PAC000197_f;PAC000197_g;PAC000197_g_uc | 41 | 0.1902 |
| PAC000661_g PAC001095_s | Bacteria;Firmicutes;Clostridia;Clostridiales;Ruminococcaceae;PAC000661_g;PAC001095_s | 40 | 0.1855 |
| Pseudoflavonifractor PAC001574_s group | Bacteria;Firmicutes;Clostridia;Clostridiales;Ruminococcaceae;Pseudoflavonifractor;PAC001574_s group | 40 | 0.1855 |
| Pseudoflavonifractor_uc | Bacteria;Firmicutes;Clostridia;Clostridiales;Ruminococcaceae;Pseudoflavonifractor;Pseudoflavonifractor_uc | 40 | 0.1855 |
| Bacteroides EQ973383_s | Bacteria;Bacteroidetes;Bacteroidia;Bacteroidales;Bacteroidaceae;Bacteroides;EQ973383_s | 39 | 0.1809 |
| Pseudoflavonifractor PAC001379_s | Bacteria;Firmicutes;Clostridia;Clostridiales;Ruminococcaceae;Pseudoflavonifractor;PAC001379_s | 39 | 0.1809 |
| Acetatifactor PAC001773_s | Bacteria;Firmicutes;Clostridia;Clostridiales;Lachnospiraceae;Acetatifactor;PAC001773_s | 39 | 0.1809 |
| Pseudoflavonifractor PAC001087_s | Bacteria;Firmicutes;Clostridia;Clostridiales;Ruminococcaceae;Pseudoflavonifractor;PAC001087_s | 38 | 0.1763 |
| PAC001219_g HM124151_s | Bacteria;Firmicutes;Clostridia;Clostridiales;Christensenellaceae;PAC001219_g;HM124151_s | 37 | 0.1716 |
| PAC001165_g PAC001384_s | Bacteria;Firmicutes;Clostridia;Clostridiales;Lachnospiraceae;PAC001165_g;PAC001384_s | 37 | 0.1716 |
| PAC001221_g PAC001221_s | Bacteria;Firmicutes;Clostridia;Clostridiales;Dehalobacterium_f;PAC001221_g;PAC001221_s | 36 | 0.167 |
| PAC001544_g PAC001544_s | Bacteria;Firmicutes;Clostridia;Clostridiales;Lachnospiraceae;PAC001544_g;PAC001544_s | 35 | 0.1623 |
| PAC001092_g PAC001668_s | Bacteria;Firmicutes;Clostridia;Clostridiales;Lachnospiraceae;PAC001092_g;PAC001668_s | 35 | 0.1623 |
| PAC001402_g PAC001748_s | Bacteria;Firmicutes;Clostridia;Clostridiales;Ruminococcaceae;PAC001402_g;PAC001748_s | 35 | 0.1623 |
| Oscillibacter PAC001188_s | Bacteria;Firmicutes;Clostridia;Clostridiales;Ruminococcaceae;Oscillibacter;PAC001188_s | 34 | 0.1577 |
| Clostridium_g21 PAC002492_s | Bacteria;Firmicutes;Clostridia;Clostridiales;Lachnospiraceae;Clostridium_g21;PAC002492_s | 34 | 0.1577 |
| PAC000661_g_uc | Bacteria;Firmicutes;Clostridia;Clostridiales;Ruminococcaceae;PAC000661_g;PAC000661_g_uc | 34 | 0.1577 |
| Enterobacteriaceae group | Bacteria;Proteobacteria;Gammaproteobacteria;Enterobacterales;Enterobacteriaceae;Enterobacteriaceae_g;Enterobacteriaceae group | 33 | 0.1531 |
| Pseudoflavonifractor KI535319_s | Bacteria;Firmicutes;Clostridia;Clostridiales;Ruminococcaceae;Pseudoflavonifractor;KI535319_s | 33 | 0.1531 |
| PAC001219_g PAC001280_s | Bacteria;Firmicutes;Clostridia;Clostridiales;Christensenellaceae;PAC001219_g;PAC001280_s | 33 | 0.1531 |
| Parabacteroides goldsteinii | Bacteria;Bacteroidetes;Bacteroidia;Bacteroidales;Porphyromonadaceae;Parabacteroides;Parabacteroides goldsteinii | 32 | 0.1484 |
| KE159571_g PAC001497_s | Bacteria;Firmicutes;Clostridia;Clostridiales;Lachnospiraceae;KE159571_g;PAC001497_s | 31 | 0.1438 |
| PAC000186_g PAC001806_s | Bacteria;Bacteroidetes;Bacteroidia;Bacteroidales;Muribaculaceae;PAC000186_g;PAC001806_s | 29 | 0.1345 |
| PAC000661_g PAC001154_s | Bacteria;Firmicutes;Clostridia;Clostridiales;Ruminococcaceae;PAC000661_g;PAC001154_s | 28 | 0.1299 |
| LT706945_g PAC001747_s | Bacteria;Proteobacteria;Deltaproteobacteria;Desulfovibrionales;Desulfovibrionaceae;LT706945_g;PAC001747_s | 28 | 0.1299 |
| PAC000186_g PAC001064_s | Bacteria;Bacteroidetes;Bacteroidia;Bacteroidales;Muribaculaceae;PAC000186_g;PAC001064_s | 27 | 0.1252 |
| Pseudoflavonifractor PAC001550_s | Bacteria;Firmicutes;Clostridia;Clostridiales;Ruminococcaceae;Pseudoflavonifractor;PAC001550_s | 27 | 0.1252 |
| KE159600_g EU511097_s | Bacteria;Firmicutes;Clostridia;Clostridiales;Lachnospiraceae;KE159600_g;EU511097_s | 26 | 0.1206 |
| PAC002314_g PAC002314_s | Bacteria;Firmicutes;Erysipelotrichi;Erysipelotrichales;Erysipelotrichaceae;PAC002314_g;PAC002314_s | 26 | 0.1206 |
| Mucispirillum schaedleri | Bacteria;Deferribacteres;Deferribacteres_c;Deferribacterales;Deferribacteraceae;Mucispirillum;Mucispirillum schaedleri | 25 | 0.116 |
| PAC000195_g PAC000195_s | Bacteria;Firmicutes;Clostridia;Clostridiales;Lachnospiraceae;PAC000195_g;PAC000195_s | 25 | 0.116 |
| Pseudoflavonifractor FJ879979_s | Bacteria;Firmicutes;Clostridia;Clostridiales;Ruminococcaceae;Pseudoflavonifractor;FJ879979_s | 24 | 0.1113 |
| Clostridium_g21_uc | Bacteria;Firmicutes;Clostridia;Clostridiales;Lachnospiraceae;Clostridium_g21;Clostridium_g21_uc | 24 | 0.1113 |
| Faecalibaculum rodentium | Bacteria;Firmicutes;Erysipelotrichi;Erysipelotrichales;Erysipelotrichaceae;Faecalibaculum;Faecalibaculum rodentium | 23 | 0.1067 |
| Acholeplasma_g2 PAC001080_s | Bacteria;Tenericutes;Mollicutes;Acholeplasmatales;Acholeplasmataceae;Acholeplasma_g2;PAC001080_s | 23 | 0.1067 |
| PAC000748_g PAC001099_s | Bacteria;Firmicutes;Clostridia;Clostridiales;Ruminococcaceae;PAC000748_g;PAC001099_s | 23 | 0.1067 |
| Bacteroides faecichinchillae | Bacteria;Bacteroidetes;Bacteroidia;Bacteroidales;Bacteroidaceae;Bacteroides;Bacteroides faecichinchillae | 22 | 0.102 |
| Caproiciproducens PAC001505_s | Bacteria;Firmicutes;Clostridia;Clostridiales;Ruminococcaceae;Caproiciproducens;PAC001505_s | 22 | 0.102 |
| PAC001385_g PAC001727_s group | Bacteria;Firmicutes;Clostridia;Clostridiales;Lachnospiraceae;PAC001385_g;PAC001727_s group | 22 | 0.102 |
| PAC001778_g PAC001778_s | Bacteria;Firmicutes;Clostridia;Clostridiales;Ruminococcaceae;PAC001778_g;PAC001778_s | 21 | 0.0974 |
| Citrobacter_uc | Bacteria;Proteobacteria;Gammaproteobacteria;Enterobacterales;Enterobacteriaceae;Citrobacter;Citrobacter_uc | 21 | 0.0974 |
| PAC001066_g PAC001066_s | Bacteria;Bacteroidetes;Bacteroidia;Bacteroidales;Muribaculaceae;PAC001066_g;PAC001066_s | 20 | 0.0928 |
| Acetatifactor PAC001463_s | Bacteria;Firmicutes;Clostridia;Clostridiales;Lachnospiraceae;Acetatifactor;PAC001463_s | 20 | 0.0928 |
| Citrobacter portucalensis | Bacteria;Proteobacteria;Gammaproteobacteria;Enterobacterales;Enterobacteriaceae;Citrobacter;Citrobacter portucalensis | 18 | 0.0835 |
| PAC000664_g EU504374_s | Bacteria;Firmicutes;Clostridia;Clostridiales;Lachnospiraceae;PAC000664_g;EU504374_s | 18 | 0.0835 |
| Oscillibacter KE159714_s group | Bacteria;Firmicutes;Clostridia;Clostridiales;Ruminococcaceae;Oscillibacter;KE159714_s group | 18 | 0.0835 |
| Oscillibacter PAC001526_s | Bacteria;Firmicutes;Clostridia;Clostridiales;Ruminococcaceae;Oscillibacter;PAC001526_s | 18 | 0.0835 |
| Parabacteroides PAC001783_s | Bacteria;Bacteroidetes;Bacteroidia;Bacteroidales;Porphyromonadaceae;Parabacteroides;PAC001783_s | 18 | 0.0835 |
| PAC000661_g EU843173_s | Bacteria;Firmicutes;Clostridia;Clostridiales;Ruminococcaceae;PAC000661_g;EU843173_s | 17 | 0.0789 |
| Oscillibacter PAC001156_s | Bacteria;Firmicutes;Clostridia;Clostridiales;Ruminococcaceae;Oscillibacter;PAC001156_s | 17 | 0.0789 |
| PAC000661_g PAC001536_s | Bacteria;Firmicutes;Clostridia;Clostridiales;Ruminococcaceae;PAC000661_g;PAC001536_s | 17 | 0.0789 |
| PAC001112_g PAC002441_s | Bacteria;Bacteroidetes;Bacteroidia;Bacteroidales;Muribaculaceae;PAC001112_g;PAC002441_s | 17 | 0.0789 |
| PAC000661_g GQ358437_s | Bacteria;Firmicutes;Clostridia;Clostridiales;Ruminococcaceae;PAC000661_g;GQ358437_s | 16 | 0.0742 |
| PAC001457_g PAC001495_s group | Bacteria;Firmicutes;Clostridia;Clostridiales;Lachnospiraceae;PAC001457_g;PAC001495_s group | 16 | 0.0742 |
| Oscillibacter PAC001502_s | Bacteria;Firmicutes;Clostridia;Clostridiales;Ruminococcaceae;Oscillibacter;PAC001502_s | 16 | 0.0742 |
| Sporobacter PAC001659_s | Bacteria;Firmicutes;Clostridia;Clostridiales;Ruminococcaceae;Sporobacter;PAC001659_s | 16 | 0.0742 |
| KE159797_g_uc | Bacteria;Firmicutes;Clostridia;Clostridiales;Lachnospiraceae;KE159797_g;KE159797_g_uc | 16 | 0.0742 |
| GQ451199_g EU472543_s | Bacteria;Firmicutes;Clostridia;Clostridiales;Ruminococcaceae;GQ451199_g;EU472543_s | 15 | 0.0696 |
| PAC001219_g PAC002168_s | Bacteria;Firmicutes;Clostridia;Clostridiales;Christensenellaceae;PAC001219_g;PAC002168_s | 15 | 0.0696 |
| Acetatifactor PAC002332_s | Bacteria;Firmicutes;Clostridia;Clostridiales;Lachnospiraceae;Acetatifactor;PAC002332_s | 15 | 0.0696 |
| Megamonas rupellensis group | Bacteria;Firmicutes;Negativicutes;Selenomonadales;Selenomonadaceae;Megamonas;Megamonas rupellensis group | 14 | 0.0649 |
| Alistipes PAC001060_s | Bacteria;Bacteroidetes;Bacteroidia;Bacteroidales;Rikenellaceae;Alistipes;PAC001060_s | 14 | 0.0649 |
| Massilioclostridium PAC001521_s | Bacteria;Firmicutes;Clostridia;Clostridiales;Ruminococcaceae;Massilioclostridium;PAC001521_s | 14 | 0.0649 |
| PAC001524_g PAC001524_s | Bacteria;Firmicutes;Clostridia;Clostridiales;Lachnospiraceae;PAC001524_g;PAC001524_s | 14 | 0.0649 |
| KE159600_g PAC001901_s | Bacteria;Firmicutes;Clostridia;Clostridiales;Lachnospiraceae;KE159600_g;PAC001901_s | 14 | 0.0649 |
| Blautia wexlerae | Bacteria;Firmicutes;Clostridia;Clostridiales;Lachnospiraceae;Blautia;Blautia wexlerae | 13 | 0.0603 |
| PAC000661_g AB185622_s | Bacteria;Firmicutes;Clostridia;Clostridiales;Ruminococcaceae;PAC000661_g;AB185622_s | 13 | 0.0603 |
| Clostridium_g24 PAC000183_s | Bacteria;Firmicutes;Clostridia;Clostridiales;Lachnospiraceae;Clostridium_g24;PAC000183_s | 13 | 0.0603 |
| Paludicola PAC000694_s | Bacteria;Firmicutes;Clostridia;Clostridiales;Ruminococcaceae;Paludicola;PAC000694_s | 13 | 0.0603 |
| PAC001068_g PAC001071_s | Bacteria;Bacteroidetes;Bacteroidia;Bacteroidales;Muribaculaceae;PAC001068_g;PAC001071_s | 13 | 0.0603 |
| Oscillibacter PAC001584_s group | Bacteria;Firmicutes;Clostridia;Clostridiales;Ruminococcaceae;Oscillibacter;PAC001584_s group | 13 | 0.0603 |
| PAC001219_g EF603174_s | Bacteria;Firmicutes;Clostridia;Clostridiales;Christensenellaceae;PAC001219_g;EF603174_s | 12 | 0.0557 |
| Eubacterium_g6 EU510495_s | Bacteria;Firmicutes;Clostridia;Clostridiales;Lachnospiraceae;Eubacterium_g6;EU510495_s | 12 | 0.0557 |
| PAC000748_g PAC001496_s | Bacteria;Firmicutes;Clostridia;Clostridiales;Ruminococcaceae;PAC000748_g;PAC001496_s | 12 | 0.0557 |
| PAC001138_g PAC001518_s | Bacteria;Firmicutes;Clostridia;Clostridiales;Lachnospiraceae;PAC001138_g;PAC001518_s | 12 | 0.0557 |
| PAC001516_g PAC001725_s | Bacteria;Firmicutes;Clostridia;Clostridiales;Lachnospiraceae;PAC001516_g;PAC001725_s | 12 | 0.0557 |
| PAC001043_g PAC002511_s group | Bacteria;Firmicutes;Clostridia;Clostridiales;Lachnospiraceae;PAC001043_g;PAC002511_s group | 12 | 0.0557 |
| PAC001516_g_uc | Bacteria;Firmicutes;Clostridia;Clostridiales;Lachnospiraceae;PAC001516_g;PAC001516_g_uc | 12 | 0.0557 |
| Alistipes shahii | Bacteria;Bacteroidetes;Bacteroidia;Bacteroidales;Rikenellaceae;Alistipes;Alistipes shahii | 11 | 0.051 |
| Blautia hansenii group | Bacteria;Firmicutes;Clostridia;Clostridiales;Lachnospiraceae;Blautia;Blautia hansenii group | 11 | 0.051 |
| Citrobacter werkmanii | Bacteria;Proteobacteria;Gammaproteobacteria;Enterobacterales;Enterobacteriaceae;Citrobacter;Citrobacter werkmanii | 11 | 0.051 |
| Enterorhabdus caecimuris | Bacteria;Actinobacteria;Coriobacteriia;Coriobacteriales;Coriobacteriaceae;Enterorhabdus;Enterorhabdus caecimuris | 11 | 0.051 |
| Clostridium_g21 KE159781_s | Bacteria;Firmicutes;Clostridia;Clostridiales;Lachnospiraceae;Clostridium_g21;KE159781_s | 11 | 0.051 |
| Clostridium_g21 PAC001085_s | Bacteria;Firmicutes;Clostridia;Clostridiales;Lachnospiraceae;Clostridium_g21;PAC001085_s | 11 | 0.051 |
| Subdoligranulum PAC001173_s | Bacteria;Firmicutes;Clostridia;Clostridiales;Ruminococcaceae;Subdoligranulum;PAC001173_s | 11 | 0.051 |
| PAC001201_g PAC001201_s group | Bacteria;Firmicutes;Clostridia;Clostridiales;Lachnospiraceae;PAC001201_g;PAC001201_s group | 11 | 0.051 |
| FTRU_g PAC001367_s | Bacteria;Firmicutes;Clostridia;Clostridiales;Ruminococcaceae;FTRU_g;PAC001367_s | 11 | 0.051 |
| Acetatifactor PAC001373_s | Bacteria;Firmicutes;Clostridia;Clostridiales;Lachnospiraceae;Acetatifactor;PAC001373_s | 11 | 0.051 |
| PAC001516_g PAC001516_s | Bacteria;Firmicutes;Clostridia;Clostridiales;Lachnospiraceae;PAC001516_g;PAC001516_s | 11 | 0.051 |
| Oscillibacter PAC001522_s | Bacteria;Firmicutes;Clostridia;Clostridiales;Ruminococcaceae;Oscillibacter;PAC001522_s | 11 | 0.051 |
| KE159797_g PAC001222_s | Bacteria;Firmicutes;Clostridia;Clostridiales;Lachnospiraceae;KE159797_g;PAC001222_s | 10 | 0.0464 |
| Emergencia PAC001235_s | Bacteria;Firmicutes;Clostridia;Clostridiales;Mogibacterium_f;Emergencia;PAC001235_s | 10 | 0.0464 |
| Paludicola PAC001507_s | Bacteria;Firmicutes;Clostridia;Clostridiales;Ruminococcaceae;Paludicola;PAC001507_s | 10 | 0.0464 |
| PAC001092_g PAC001587_s | Bacteria;Firmicutes;Clostridia;Clostridiales;Lachnospiraceae;PAC001092_g;PAC001587_s | 10 | 0.0464 |
| PAC001144_g PAC001593_s | Bacteria;Firmicutes;Clostridia;Clostridiales;Ruminococcaceae;PAC001144_g;PAC001593_s | 10 | 0.0464 |
| PAC001573_g PAC001808_s | Bacteria;Firmicutes;Clostridia;Clostridiales;Mogibacterium_f;PAC001573_g;PAC001808_s | 10 | 0.0464 |
| Acutalibacter muris | Bacteria;Firmicutes;Clostridia;Clostridiales;Ruminococcaceae;Acutalibacter;Acutalibacter muris | 9 | 0.0417 |
| Anaerostipes hadrus group | Bacteria;Firmicutes;Clostridia;Clostridiales;Lachnospiraceae;Anaerostipes;Anaerostipes hadrus group | 9 | 0.0417 |
| Phascolarctobacterium faecium | Bacteria;Firmicutes;Negativicutes;Acidaminococcales;Acidaminococcaceae;Phascolarctobacterium;Phascolarctobacterium faecium | 9 | 0.0417 |
| Streptococcus danieliae | Bacteria;Firmicutes;Bacilli;Lactobacillales;Streptococcaceae;Streptococcus;Streptococcus danieliae | 9 | 0.0417 |
| Blautia LT635539_s group | Bacteria;Firmicutes;Clostridia;Clostridiales;Lachnospiraceae;Blautia;LT635539_s group | 9 | 0.0417 |
| PAC001043_g PAC001557_s | Bacteria;Firmicutes;Clostridia;Clostridiales;Lachnospiraceae;PAC001043_g;PAC001557_s | 9 | 0.0417 |
| PAC001270_g PAC001899_s group | Bacteria;Firmicutes;Clostridia;Clostridiales;Lachnospiraceae;PAC001270_g;PAC001899_s group | 9 | 0.0417 |
| Parabacteroides merdae | Bacteria;Bacteroidetes;Bacteroidia;Bacteroidales;Porphyromonadaceae;Parabacteroides;Parabacteroides merdae | 8 | 0.0371 |
| Acetatifactor HM124297_s | Bacteria;Firmicutes;Clostridia;Clostridiales;Lachnospiraceae;Acetatifactor;HM124297_s | 8 | 0.0371 |
| Monoglobus PAC001245_s | Bacteria;Firmicutes;Clostridia;Clostridiales;Ruminococcaceae;Monoglobus;PAC001245_s | 8 | 0.0371 |
| PAC001301_g PAC001301_s | Bacteria;Firmicutes;Clostridia;Clostridiales;Christensenellaceae;PAC001301_g;PAC001301_s | 8 | 0.0371 |
| Frisingicoccus PAC001399_s | Bacteria;Firmicutes;Clostridia;Clostridiales;Lachnospiraceae;Frisingicoccus;PAC001399_s | 8 | 0.0371 |
| Pseudoflavonifractor PAC001548_s | Bacteria;Firmicutes;Clostridia;Clostridiales;Ruminococcaceae;Pseudoflavonifractor;PAC001548_s | 8 | 0.0371 |
| PAC001144_g PAC001626_s | Bacteria;Firmicutes;Clostridia;Clostridiales;Ruminococcaceae;PAC001144_g;PAC001626_s | 8 | 0.0371 |
| Pseudoflavonifractor PAC001786_s | Bacteria;Firmicutes;Clostridia;Clostridiales;Ruminococcaceae;Pseudoflavonifractor;PAC001786_s | 8 | 0.0371 |
| PAC000197_g PAC001787_s | Bacteria;Tenericutes;Mollicutes;PAC001057_c;PAC000197_f;PAC000197_g;PAC001787_s | 8 | 0.0371 |
| Lactobacillus_uc | Bacteria;Firmicutes;Bacilli;Lactobacillales;Lactobacillaceae;Lactobacillus;Lactobacillus_uc | 8 | 0.0371 |
| Parabacteroides_uc | Bacteria;Bacteroidetes;Bacteroidia;Bacteroidales;Porphyromonadaceae;Parabacteroides;Parabacteroides_uc | 8 | 0.0371 |
| GQ458233_g_uc | Bacteria;Firmicutes;Clostridia;Clostridiales;Ruminococcaceae;GQ458233_g;GQ458233_g_uc | 8 | 0.0371 |
| PAC001360_g_uc | Bacteria;Firmicutes;Clostridia;Clostridiales;Christensenellaceae;PAC001360_g;PAC001360_g_uc | 8 | 0.0371 |
| Bacillus cereus group | Bacteria;Firmicutes;Bacilli;Bacillales;Bacillaceae;Bacillus;Bacillus cereus group | 7 | 0.0325 |
| Citrobacter braakii | Bacteria;Proteobacteria;Gammaproteobacteria;Enterobacterales;Enterobacteriaceae;Citrobacter;Citrobacter braakii | 7 | 0.0325 |
| Fusicatenibacter saccharivorans | Bacteria;Firmicutes;Clostridia;Clostridiales;Lachnospiraceae;Fusicatenibacter;Fusicatenibacter saccharivorans | 7 | 0.0325 |
| Veillonella dispar | Bacteria;Firmicutes;Negativicutes;Veillonellales;Veillonellaceae;Veillonella;Veillonella dispar | 7 | 0.0325 |
| Eubacterium_g6 JQ083736_s | Bacteria;Firmicutes;Clostridia;Clostridiales;Lachnospiraceae;Eubacterium_g6;JQ083736_s | 7 | 0.0325 |
| Dialister PAC001040_s | Bacteria;Firmicutes;Negativicutes;Veillonellales;Veillonellaceae;Dialister;PAC001040_s | 7 | 0.0325 |
| Eubacterium_g23 PAC001051_s | Bacteria;Firmicutes;Clostridia;Clostridiales;Ruminococcaceae;Eubacterium_g23;PAC001051_s | 7 | 0.0325 |
| PAC001062_g PAC001062_s | Bacteria;Bacteroidetes;Bacteroidia;Bacteroidales;Muribaculaceae;PAC001062_g;PAC001062_s | 7 | 0.0325 |
| PAC000664_g PAC001083_s | Bacteria;Firmicutes;Clostridia;Clostridiales;Lachnospiraceae;PAC000664_g;PAC001083_s | 7 | 0.0325 |
| Clostridium_g24 PAC001164_s | Bacteria;Firmicutes;Clostridia;Clostridiales;Lachnospiraceae;Clostridium_g24;PAC001164_s | 7 | 0.0325 |
| PAC001165_g PAC001165_s | Bacteria;Firmicutes;Clostridia;Clostridiales;Lachnospiraceae;PAC001165_g;PAC001165_s | 7 | 0.0325 |
| PAC001103_g PAC001361_s | Bacteria;Firmicutes;Clostridia;Clostridiales;Lachnospiraceae;PAC001103_g;PAC001361_s | 7 | 0.0325 |
| PAC001485_g PAC001485_s | Bacteria;Bacteroidetes;Bacteroidia;Bacteroidales;Muribaculaceae;PAC001485_g;PAC001485_s | 7 | 0.0325 |
| PAC001402_g PAC001546_s | Bacteria;Firmicutes;Clostridia;Clostridiales;Ruminococcaceae;PAC001402_g;PAC001546_s | 7 | 0.0325 |
| PAC001402_g PAC001561_s | Bacteria;Firmicutes;Clostridia;Clostridiales;Ruminococcaceae;PAC001402_g;PAC001561_s | 7 | 0.0325 |
| PAC000683_g PAC001581_s | Bacteria;Firmicutes;Clostridia;Clostridiales;Ruminococcaceae;PAC000683_g;PAC001581_s | 7 | 0.0325 |
| PAC001270_g PAC002420_s | Bacteria;Firmicutes;Clostridia;Clostridiales;Lachnospiraceae;PAC001270_g;PAC002420_s | 7 | 0.0325 |
| Clostridium_g21 PAC002512_s group | Bacteria;Firmicutes;Clostridia;Clostridiales;Lachnospiraceae;Clostridium_g21;PAC002512_s group | 7 | 0.0325 |
| Catenibacterium PAC002523_s | Bacteria;Firmicutes;Erysipelotrichi;Erysipelotrichales;Erysipelotrichaceae;Catenibacterium;PAC002523_s | 7 | 0.0325 |
| Alistipes finegoldii | Bacteria;Bacteroidetes;Bacteroidia;Bacteroidales;Rikenellaceae;Alistipes;Alistipes finegoldii | 6 | 0.0278 |
| Bacteroides caecimuris | Bacteria;Bacteroidetes;Bacteroidia;Bacteroidales;Bacteroidaceae;Bacteroides;Bacteroides caecimuris | 6 | 0.0278 |
| Bacteroides fragilis | Bacteria;Bacteroidetes;Bacteroidia;Bacteroidales;Bacteroidaceae;Bacteroides;Bacteroides fragilis | 6 | 0.0278 |
| Bacteroides thetaiotaomicron | Bacteria;Bacteroidetes;Bacteroidia;Bacteroidales;Bacteroidaceae;Bacteroides;Bacteroides thetaiotaomicron | 6 | 0.0278 |
| Bacteroides xylanisolvens group | Bacteria;Bacteroidetes;Bacteroidia;Bacteroidales;Bacteroidaceae;Bacteroides;Bacteroides xylanisolvens group | 6 | 0.0278 |
| Blautia obeum | Bacteria;Firmicutes;Clostridia;Clostridiales;Lachnospiraceae;Blautia;Blautia obeum | 6 | 0.0278 |
| Clostridium celatum group | Bacteria;Firmicutes;Clostridia;Clostridiales;Clostridiaceae;Clostridium;Clostridium celatum group | 6 | 0.0278 |
| Clostridium clostridioforme group | Bacteria;Firmicutes;Clostridia;Clostridiales;Lachnospiraceae;Clostridium_g24;Clostridium clostridioforme group | 6 | 0.0278 |
| Collinsella aerofaciens group | Bacteria;Actinobacteria;Coriobacteriia;Coriobacteriales;Coriobacteriaceae;Collinsella;Collinsella aerofaciens group | 6 | 0.0278 |
| Eubacterium hallii | Bacteria;Firmicutes;Clostridia;Clostridiales;Lachnospiraceae;Eubacterium_g5;Eubacterium hallii | 6 | 0.0278 |
| Lactobacillus rogosae group | Bacteria;Firmicutes;Clostridia;Clostridiales;Lachnospiraceae;Lachnospira;Lactobacillus rogosae group | 6 | 0.0278 |
| Ruminococcus callidus | Bacteria;Firmicutes;Clostridia;Clostridiales;Ruminococcaceae;Ruminococcus;Ruminococcus callidus | 6 | 0.0278 |
| Ruminococcus gnavus | Bacteria;Firmicutes;Clostridia;Clostridiales;Lachnospiraceae;Ruminococcus_g5;Ruminococcus gnavus | 6 | 0.0278 |
| Caproiciproducens DQ777923_s | Bacteria;Firmicutes;Clostridia;Clostridiales;Ruminococcaceae;Caproiciproducens;DQ777923_s | 6 | 0.0278 |
| Oscillibacter EU137434_s | Bacteria;Firmicutes;Clostridia;Clostridiales;Ruminococcaceae;Oscillibacter;EU137434_s | 6 | 0.0278 |
| Bacteroides EU728720_s | Bacteria;Bacteroidetes;Bacteroidia;Bacteroidales;Bacteroidaceae;Bacteroides;EU728720_s | 6 | 0.0278 |
| Frisingicoccus PAC000700_s | Bacteria;Firmicutes;Clostridia;Clostridiales;Lachnospiraceae;Frisingicoccus;PAC000700_s | 6 | 0.0278 |
| PAC000661_g PAC001182_s | Bacteria;Firmicutes;Clostridia;Clostridiales;Ruminococcaceae;PAC000661_g;PAC001182_s | 6 | 0.0278 |
| PAC001092_g PAC001230_s | Bacteria;Firmicutes;Clostridia;Clostridiales;Lachnospiraceae;PAC001092_g;PAC001230_s | 6 | 0.0278 |
| PAC001528_g PAC001528_s | Bacteria;Firmicutes;Erysipelotrichi;Erysipelotrichales;Erysipelotrichaceae;PAC001528_g;PAC001528_s | 6 | 0.0278 |
| Pseudoflavonifractor PAC001547_s | Bacteria;Firmicutes;Clostridia;Clostridiales;Ruminococcaceae;Pseudoflavonifractor;PAC001547_s | 6 | 0.0278 |
| Harryflintia PAC001713_s | Bacteria;Firmicutes;Clostridia;Clostridiales;Ruminococcaceae;Harryflintia;PAC001713_s | 6 | 0.0278 |
| Alistipes putredinis | Bacteria;Bacteroidetes;Bacteroidia;Bacteroidales;Rikenellaceae;Alistipes;Alistipes putredinis | 5 | 0.0232 |
| Bifidobacterium catenulatum group | Bacteria;Actinobacteria;Actinobacteria_c;Bifidobacteriales;Bifidobacteriaceae;Bifidobacterium;Bifidobacterium catenulatum group | 5 | 0.0232 |
| Blautia luti | Bacteria;Firmicutes;Clostridia;Clostridiales;Lachnospiraceae;Blautia;Blautia luti | 5 | 0.0232 |
| Citrobacter murliniae | Bacteria;Proteobacteria;Gammaproteobacteria;Enterobacterales;Enterobacteriaceae;Citrobacter;Citrobacter murliniae | 5 | 0.0232 |
| Lachnospira pectinoschiza | Bacteria;Firmicutes;Clostridia;Clostridiales;Lachnospiraceae;Lachnospira;Lachnospira pectinoschiza | 5 | 0.0232 |
| Proteus mirabilis | Bacteria;Proteobacteria;Gammaproteobacteria;Enterobacterales;Morganellaceae;Proteus;Proteus mirabilis | 5 | 0.0232 |
| Roseburia intestinalis group | Bacteria;Firmicutes;Clostridia;Clostridiales;Lachnospiraceae;Roseburia;Roseburia intestinalis group | 5 | 0.0232 |
| Streptococcus acidominimus group | Bacteria;Firmicutes;Bacilli;Lactobacillales;Streptococcaceae;Streptococcus;Streptococcus acidominimus group | 5 | 0.0232 |
| Ruminococcus HF545616_s | Bacteria;Firmicutes;Clostridia;Clostridiales;Ruminococcaceae;Ruminococcus;HF545616_s | 5 | 0.0232 |
| Blautia LN913006_s group | Bacteria;Firmicutes;Clostridia;Clostridiales;Lachnospiraceae;Blautia;LN913006_s group | 5 | 0.0232 |
| Eubacterium_g5 LT907848_s | Bacteria;Firmicutes;Clostridia;Clostridiales;Lachnospiraceae;Eubacterium_g5;LT907848_s | 5 | 0.0232 |
| Frisingicoccus PAC000688_s | Bacteria;Firmicutes;Clostridia;Clostridiales;Lachnospiraceae;Frisingicoccus;PAC000688_s | 5 | 0.0232 |
| PAC001103_g PAC001103_s | Bacteria;Firmicutes;Clostridia;Clostridiales;Lachnospiraceae;PAC001103_g;PAC001103_s | 5 | 0.0232 |
| Eubacterium_g17 PAC001119_s | Bacteria;Firmicutes;Clostridia;Clostridiales;Lachnospiraceae;Eubacterium_g17;PAC001119_s | 5 | 0.0232 |
| PAC001177_g PAC001177_s | Bacteria;Firmicutes;Clostridia;Clostridiales;Lachnospiraceae;PAC001177_g;PAC001177_s | 5 | 0.0232 |
| PAC001323_g PAC001323_s | Bacteria;Tenericutes;Mollicutes;PAC001057_c;PAC000197_f;PAC001323_g;PAC001323_s | 5 | 0.0232 |
| Pseudoflavonifractor PAC001370_s | Bacteria;Firmicutes;Clostridia;Clostridiales;Ruminococcaceae;Pseudoflavonifractor;PAC001370_s | 5 | 0.0232 |
| PAC000671_g PAC001473_s | Bacteria;Firmicutes;Clostridia;Clostridiales;Lachnospiraceae;PAC000671_g;PAC001473_s | 5 | 0.0232 |
| KE159571_g PAC001559_s | Bacteria;Firmicutes;Clostridia;Clostridiales;Lachnospiraceae;KE159571_g;PAC001559_s | 5 | 0.0232 |
| PAC001068_g PAC001736_s | Bacteria;Bacteroidetes;Bacteroidia;Bacteroidales;Muribaculaceae;PAC001068_g;PAC001736_s | 5 | 0.0232 |
| Clostridium_g21 PAC001772_s | Bacteria;Firmicutes;Clostridia;Clostridiales;Lachnospiraceae;Clostridium_g21;PAC001772_s | 5 | 0.0232 |
| Eubacterium_g6 PAC002402_s | Bacteria;Firmicutes;Clostridia;Clostridiales;Lachnospiraceae;Eubacterium_g6;PAC002402_s | 5 | 0.0232 |
| LARJ_g_uc | Bacteria;Proteobacteria;Alphaproteobacteria;Rhodospirillales;Rhodospirillaceae;LARJ_g;LARJ_g_uc | 5 | 0.0232 |
| PAC001219_g_uc | Bacteria;Firmicutes;Clostridia;Clostridiales;Christensenellaceae;PAC001219_g;PAC001219_g_uc | 5 | 0.0232 |
| Bacteroidaceae_uc | Bacteria;Bacteroidetes;Bacteroidia;Bacteroidales;Bacteroidaceae;Bacteroidaceae_uc | 4 | 0.0186 |
| Enterobacteriaceae_uc | Bacteria;Proteobacteria;Gammaproteobacteria;Enterobacterales;Enterobacteriaceae;Enterobacteriaceae_uc | 4 | 0.0186 |
| Acidaminococcus fermentans | Bacteria;Firmicutes;Negativicutes;Acidaminococcales;Acidaminococcaceae;Acidaminococcus;Acidaminococcus fermentans | 4 | 0.0186 |
| Bacillus subtilis group | Bacteria;Firmicutes;Bacilli;Bacillales;Bacillaceae;Bacillus;Bacillus subtilis group | 4 | 0.0186 |
| Bacteroides intestinalis | Bacteria;Bacteroidetes;Bacteroidia;Bacteroidales;Bacteroidaceae;Bacteroides;Bacteroides intestinalis | 4 | 0.0186 |
| Clostridium tertium group | Bacteria;Firmicutes;Clostridia;Clostridiales;Clostridiaceae;Clostridium;Clostridium tertium group | 4 | 0.0186 |
| Mitsuokella multacida | Bacteria;Firmicutes;Negativicutes;Selenomonadales;Selenomonadaceae;Mitsuokella;Mitsuokella multacida | 4 | 0.0186 |
| Prevotella stercorea | Bacteria;Bacteroidetes;Bacteroidia;Bacteroidales;Prevotellaceae;Prevotella;Prevotella stercorea | 4 | 0.0186 |
| Streptococcus salivarius group | Bacteria;Firmicutes;Bacilli;Lactobacillales;Streptococcaceae;Streptococcus;Streptococcus salivarius group | 4 | 0.0186 |
| Streptococcus sanguinis group | Bacteria;Firmicutes;Bacilli;Lactobacillales;Streptococcaceae;Streptococcus;Streptococcus sanguinis group | 4 | 0.0186 |
| PAC001109_g AB626901_s | Bacteria;Tenericutes;Mollicutes;PAC001057_c;PAC001057_f;PAC001109_g;AB626901_s | 4 | 0.0186 |
| Christensenella EU508399_s | Bacteria;Firmicutes;Clostridia;Clostridiales;Christensenellaceae;Christensenella;EU508399_s | 4 | 0.0186 |
| Caproiciproducens EU622658_s | Bacteria;Firmicutes;Clostridia;Clostridiales;Ruminococcaceae;Caproiciproducens;EU622658_s | 4 | 0.0186 |
| KE159571_g KE159641_s | Bacteria;Firmicutes;Clostridia;Clostridiales;Lachnospiraceae;KE159571_g;KE159641_s | 4 | 0.0186 |
| Enterobacter KQ235774_s | Bacteria;Proteobacteria;Gammaproteobacteria;Enterobacterales;Enterobacteriaceae;Enterobacter;KQ235774_s | 4 | 0.0186 |
| PAC001032_g PAC001032_s | Bacteria;Firmicutes;Clostridia;Clostridiales;Lachnospiraceae;PAC001032_g;PAC001032_s | 4 | 0.0186 |
| Eubacterium_g23 PAC001034_s | Bacteria;Firmicutes;Clostridia;Clostridiales;Ruminococcaceae;Eubacterium_g23;PAC001034_s | 4 | 0.0186 |
| PAC001068_g PAC001069_s | Bacteria;Bacteroidetes;Bacteroidia;Bacteroidales;Muribaculaceae;PAC001068_g;PAC001069_s | 4 | 0.0186 |
| Pseudoflavonifractor PAC001088_s | Bacteria;Firmicutes;Clostridia;Clostridiales;Ruminococcaceae;Pseudoflavonifractor;PAC001088_s | 4 | 0.0186 |
| PAC001313_g PAC001313_s | Bacteria;Firmicutes;Clostridia;Clostridiales;Ruminococcaceae;PAC001313_g;PAC001313_s | 4 | 0.0186 |
| PAC001149_g PAC001511_s | Bacteria;Firmicutes;Clostridia;Clostridiales;Lachnospiraceae;PAC001149_g;PAC001511_s | 4 | 0.0186 |
| Anaerotruncus PAC001590_s | Bacteria;Firmicutes;Clostridia;Clostridiales;Ruminococcaceae;Anaerotruncus;PAC001590_s | 4 | 0.0186 |
| PAC001599_g PAC001599_s | Bacteria;Firmicutes;Clostridia;Clostridiales;Ruminococcaceae;PAC001599_g;PAC001599_s | 4 | 0.0186 |
| Agathobaculum PAC001688_s | Bacteria;Firmicutes;Clostridia;Clostridiales;Ruminococcaceae;Agathobaculum;PAC001688_s | 4 | 0.0186 |
| Acetatifactor PAC001749_s | Bacteria;Firmicutes;Clostridia;Clostridiales;Lachnospiraceae;Acetatifactor;PAC001749_s | 4 | 0.0186 |
| Clostridium_g21 PAC001757_s group | Bacteria;Firmicutes;Clostridia;Clostridiales;Lachnospiraceae;Clostridium_g21;PAC001757_s group | 4 | 0.0186 |
| PAC001092_g PAC001774_s | Bacteria;Firmicutes;Clostridia;Clostridiales;Lachnospiraceae;PAC001092_g;PAC001774_s | 4 | 0.0186 |
| Paludicola PAC001784_s | Bacteria;Firmicutes;Clostridia;Clostridiales;Ruminococcaceae;Paludicola;PAC001784_s | 4 | 0.0186 |
| PAC001165_g PAC001796_s | Bacteria;Firmicutes;Clostridia;Clostridiales;Lachnospiraceae;PAC001165_g;PAC001796_s | 4 | 0.0186 |
| KE159571_g PAC002035_s | Bacteria;Firmicutes;Clostridia;Clostridiales;Lachnospiraceae;KE159571_g;PAC002035_s | 4 | 0.0186 |
| PAC001108_g PAC002140_s | Bacteria;Tenericutes;Mollicutes;PAC001057_c;PAC001057_f;PAC001108_g;PAC002140_s | 4 | 0.0186 |
| Pseudoflavonifractor PAC002302_s | Bacteria;Firmicutes;Clostridia;Clostridiales;Ruminococcaceae;Pseudoflavonifractor;PAC002302_s | 4 | 0.0186 |
| PAC002390_g PAC002390_s | Bacteria;Firmicutes;Clostridia;Clostridiales;Dehalobacterium_f;PAC002390_g;PAC002390_s | 4 | 0.0186 |
| Acetatifactor_uc | Bacteria;Firmicutes;Clostridia;Clostridiales;Lachnospiraceae;Acetatifactor;Acetatifactor_uc | 4 | 0.0186 |
| Oscillibacter_uc | Bacteria;Firmicutes;Clostridia;Clostridiales;Ruminococcaceae;Oscillibacter;Oscillibacter_uc | 4 | 0.0186 |
| Prevotella_uc | Bacteria;Bacteroidetes;Bacteroidia;Bacteroidales;Prevotellaceae;Prevotella;Prevotella_uc | 4 | 0.0186 |
| PAC000186_g_uc | Bacteria;Bacteroidetes;Bacteroidia;Bacteroidales;Muribaculaceae;PAC000186_g;PAC000186_g_uc | 4 | 0.0186 |
| Lachnospiraceae_uc | Bacteria;Firmicutes;Clostridia;Clostridiales;Lachnospiraceae;Lachnospiraceae_uc | 3 | 0.0139 |
| Agathobacter rectalis | Bacteria;Firmicutes;Clostridia;Clostridiales;Lachnospiraceae;Agathobacter;Agathobacter rectalis | 3 | 0.0139 |
| Alistipes ihumii | Bacteria;Bacteroidetes;Bacteroidia;Bacteroidales;Rikenellaceae;Alistipes;Alistipes ihumii | 3 | 0.0139 |
| Bacteroides cellulosilyticus | Bacteria;Bacteroidetes;Bacteroidia;Bacteroidales;Bacteroidaceae;Bacteroides;Bacteroides cellulosilyticus | 3 | 0.0139 |
| Bacteroides dorei | Bacteria;Bacteroidetes;Bacteroidia;Bacteroidales;Bacteroidaceae;Bacteroides;Bacteroides dorei | 3 | 0.0139 |
| Bifidobacterium adolescentis group | Bacteria;Actinobacteria;Actinobacteria_c;Bifidobacteriales;Bifidobacteriaceae;Bifidobacterium;Bifidobacterium adolescentis group | 3 | 0.0139 |
| Bifidobacterium dentium | Bacteria;Actinobacteria;Actinobacteria_c;Bifidobacteriales;Bifidobacteriaceae;Bifidobacterium;Bifidobacterium dentium | 3 | 0.0139 |
| Bifidobacterium longum group | Bacteria;Actinobacteria;Actinobacteria_c;Bifidobacteriales;Bifidobacteriaceae;Bifidobacterium;Bifidobacterium longum group | 3 | 0.0139 |
| Clostridium chartatabidum | Bacteria;Firmicutes;Clostridia;Clostridiales;Clostridiaceae;Clostridium;Clostridium chartatabidum | 3 | 0.0139 |
| Enterorhabdus mucosicola | Bacteria;Actinobacteria;Coriobacteriia;Coriobacteriales;Coriobacteriaceae;Enterorhabdus;Enterorhabdus mucosicola | 3 | 0.0139 |
| Eubacterium eligens group | Bacteria;Firmicutes;Clostridia;Clostridiales;Lachnospiraceae;Lachnospira;Eubacterium eligens group | 3 | 0.0139 |
| Gemmiger formicilis group | Bacteria;Firmicutes;Clostridia;Clostridiales;Ruminococcaceae;Subdoligranulum;Gemmiger formicilis group | 3 | 0.0139 |
| Raoultella planticola group | Bacteria;Proteobacteria;Gammaproteobacteria;Enterobacterales;Enterobacteriaceae;Raoultella;Raoultella planticola group | 3 | 0.0139 |
| Roseburia cecicola group | Bacteria;Firmicutes;Clostridia;Clostridiales;Lachnospiraceae;Roseburia;Roseburia cecicola group | 3 | 0.0139 |
| Terrisporobacter petrolearius | Bacteria;Firmicutes;Clostridia;Clostridiales;Peptostreptococcaceae;Terrisporobacter;Terrisporobacter petrolearius | 3 | 0.0139 |
| EU461719_g EU461719_s | Bacteria;Firmicutes;Clostridia;Clostridiales;Lachnospiraceae;EU461719_g;EU461719_s | 3 | 0.0139 |
| KE159605_g KE159605_s | Bacteria;Firmicutes;Clostridia;Clostridiales;Lachnospiraceae;KE159605_g;KE159605_s | 3 | 0.0139 |
| Anaerotignum PAC001031_s | Bacteria;Firmicutes;Clostridia;Clostridiales;Lachnospiraceae;Anaerotignum;PAC001031_s | 3 | 0.0139 |
| PAC000672_g PAC001048_s group | Bacteria;Firmicutes;Clostridia;Clostridiales;Ruminococcaceae;PAC000672_g;PAC001048_s group | 3 | 0.0139 |
| Parasutterella PAC001214_s | Bacteria;Proteobacteria;Betaproteobacteria;Burkholderiales;Sutterellaceae;Parasutterella;PAC001214_s | 3 | 0.0139 |
| PAC001225_g PAC001225_s | Bacteria;Firmicutes;Clostridia;Clostridiales;Lachnospiraceae;PAC001225_g;PAC001225_s | 3 | 0.0139 |
| Prevotella PAC001304_s | Bacteria;Bacteroidetes;Bacteroidia;Bacteroidales;Prevotellaceae;Prevotella;PAC001304_s | 3 | 0.0139 |
| Frisingicoccus PAC001481_s | Bacteria;Firmicutes;Clostridia;Clostridiales;Lachnospiraceae;Frisingicoccus;PAC001481_s | 3 | 0.0139 |
| Eubacterium_g6 PAC001586_s | Bacteria;Firmicutes;Clostridia;Clostridiales;Lachnospiraceae;Eubacterium_g6;PAC001586_s | 3 | 0.0139 |
| PAC001612_g PAC001612_s | Bacteria;Firmicutes;Clostridia;Clostridiales;Mogibacterium_f;PAC001612_g;PAC001612_s | 3 | 0.0139 |
| KE159571_g PAC001705_s | Bacteria;Firmicutes;Clostridia;Clostridiales;Lachnospiraceae;KE159571_g;PAC001705_s | 3 | 0.0139 |
| PAC001138_g PAC002349_s | Bacteria;Firmicutes;Clostridia;Clostridiales;Lachnospiraceae;PAC001138_g;PAC002349_s | 3 | 0.0139 |
| Oscillibacter PAC002372_s | Bacteria;Firmicutes;Clostridia;Clostridiales;Ruminococcaceae;Oscillibacter;PAC002372_s | 3 | 0.0139 |
| PAC000197_g PAC002408_s | Bacteria;Tenericutes;Mollicutes;PAC001057_c;PAC000197_f;PAC000197_g;PAC002408_s | 3 | 0.0139 |
| Allisonella histaminiformans | Bacteria;Firmicutes;Negativicutes;Veillonellales;Veillonellaceae;Dialister;Allisonella histaminiformans | 2 | 0.0093 |
| Bacteroides nordii | Bacteria;Bacteroidetes;Bacteroidia;Bacteroidales;Bacteroidaceae;Bacteroides;Bacteroides nordii | 2 | 0.0093 |
| Clostridium innocuum group | Bacteria;Firmicutes;Erysipelotrichi;Erysipelotrichales;Erysipelotrichaceae;Longicatena;Clostridium innocuum group | 2 | 0.0093 |
| Enterococcus faecium group | Bacteria;Firmicutes;Bacilli;Lactobacillales;Enterococcaceae;Enterococcus;Enterococcus faecium group | 2 | 0.0093 |
| Eubacterium ventriosum | Bacteria;Firmicutes;Clostridia;Clostridiales;Lachnospiraceae;Eubacterium_g4;Eubacterium ventriosum | 2 | 0.0093 |
| Gemella palaticanis | Bacteria;Firmicutes;Bacilli;Bacillales;Gemella_f;Gemella;Gemella palaticanis | 2 | 0.0093 |
| Lactobacillus ruminis | Bacteria;Firmicutes;Bacilli;Lactobacillales;Lactobacillaceae;Lactobacillus;Lactobacillus ruminis | 2 | 0.0093 |
| Megasphaera indica | Bacteria;Firmicutes;Negativicutes;Veillonellales;Veillonellaceae;Megasphaera;Megasphaera indica | 2 | 0.0093 |
| Paenibacillus lautus | Bacteria;Firmicutes;Bacilli;Bacillales;Paenibacillaceae;Paenibacillus;Paenibacillus lautus | 2 | 0.0093 |
| Phascolarctobacterium succinatutens group | Bacteria;Firmicutes;Negativicutes;Acidaminococcales;Acidaminococcaceae;Phascolarctobacterium;Phascolarctobacterium succinatutens group | 2 | 0.0093 |
| Prevotellamassilia timonensis | Bacteria;Bacteroidetes;Bacteroidia;Bacteroidales;Prevotellaceae;Alloprevotella;Prevotellamassilia timonensis | 2 | 0.0093 |
| Romboutsia timonensis | Bacteria;Firmicutes;Clostridia;Clostridiales;Peptostreptococcaceae;Romboutsia;Romboutsia timonensis | 2 | 0.0093 |
| Roseburia inulinivorans | Bacteria;Firmicutes;Clostridia;Clostridiales;Lachnospiraceae;Roseburia;Roseburia inulinivorans | 2 | 0.0093 |
| Streptococcus agalactiae | Bacteria;Firmicutes;Bacilli;Lactobacillales;Streptococcaceae;Streptococcus;Streptococcus agalactiae | 2 | 0.0093 |
| Sutterella wadsworthensis | Bacteria;Proteobacteria;Betaproteobacteria;Burkholderiales;Sutterellaceae;Sutterella;Sutterella wadsworthensis | 2 | 0.0093 |
| Agathobaculum AB606336_s | Bacteria;Firmicutes;Clostridia;Clostridiales;Ruminococcaceae;Agathobaculum;AB606336_s | 2 | 0.0093 |
| Arthromitus AP012202_s group | Bacteria;Firmicutes;Clostridia;Clostridiales;Clostridiaceae;Arthromitus;AP012202_s group | 2 | 0.0093 |
| Muribaculum DQ815542_s | Bacteria;Bacteroidetes;Bacteroidia;Bacteroidales;Muribaculaceae;Muribaculum;DQ815542_s | 2 | 0.0093 |
| Frisingicoccus EF409953_s | Bacteria;Firmicutes;Clostridia;Clostridiales;Lachnospiraceae;Frisingicoccus;EF409953_s | 2 | 0.0093 |
| Clostridium_g24 EU504811_s | Bacteria;Firmicutes;Clostridia;Clostridiales;Lachnospiraceae;Clostridium_g24;EU504811_s | 2 | 0.0093 |
| PAC000692_g EU505164_s | Bacteria;Firmicutes;Clostridia;Clostridiales;Lachnospiraceae;PAC000692_g;EU505164_s | 2 | 0.0093 |
| Acetatifactor EU508111_s | Bacteria;Firmicutes;Clostridia;Clostridiales;Lachnospiraceae;Acetatifactor;EU508111_s | 2 | 0.0093 |
| Acetatifactor EU509979_s | Bacteria;Firmicutes;Clostridia;Clostridiales;Lachnospiraceae;Acetatifactor;EU509979_s | 2 | 0.0093 |
| PAC001308_g EU511827_s | Bacteria;Firmicutes;Clostridia;Clostridiales;Lachnospiraceae;PAC001308_g;EU511827_s | 2 | 0.0093 |
| Pseudoflavonifractor FJ879530_s | Bacteria;Firmicutes;Clostridia;Clostridiales;Ruminococcaceae;Pseudoflavonifractor;FJ879530_s | 2 | 0.0093 |
| Caproiciproducens HQ806126_s | Bacteria;Firmicutes;Clostridia;Clostridiales;Ruminococcaceae;Caproiciproducens;HQ806126_s | 2 | 0.0093 |
| KE159605_g KB822463_s | Bacteria;Firmicutes;Clostridia;Clostridiales;Lachnospiraceae;KE159605_g;KB822463_s | 2 | 0.0093 |
| KE159797_g KE159797_s | Bacteria;Firmicutes;Clostridia;Clostridiales;Lachnospiraceae;KE159797_g;KE159797_s | 2 | 0.0093 |
| KE993550_g KE993550_s | Bacteria;Firmicutes;Clostridia;Clostridiales;Ruminococcaceae;KE993550_g;KE993550_s | 2 | 0.0093 |
| LARJ_g PAC000185_s | Bacteria;Proteobacteria;Alphaproteobacteria;Rhodospirillales;Rhodospirillaceae;LARJ_g;PAC000185_s | 2 | 0.0093 |
| PAC001100_g PAC001100_s | Bacteria;Firmicutes;Clostridia;Clostridiales;Ruminococcaceae;PAC001100_g;PAC001100_s | 2 | 0.0093 |
| Paludicola PAC001106_s | Bacteria;Firmicutes;Clostridia;Clostridiales;Ruminococcaceae;Paludicola;PAC001106_s | 2 | 0.0093 |
| PAC001109_g PAC001109_s | Bacteria;Tenericutes;Mollicutes;PAC001057_c;PAC001057_f;PAC001109_g;PAC001109_s | 2 | 0.0093 |
| PAC001116_g PAC001116_s | Bacteria;Firmicutes;Clostridia;Clostridiales;Lachnospiraceae;PAC001116_g;PAC001116_s | 2 | 0.0093 |
| KE159538_g PAC001120_s | Bacteria;Firmicutes;Clostridia;Clostridiales;Lachnospiraceae;KE159538_g;PAC001120_s | 2 | 0.0093 |
| Eubacterium_g23 PAC001121_s group | Bacteria;Firmicutes;Clostridia;Clostridiales;Ruminococcaceae;Eubacterium_g23;PAC001121_s group | 2 | 0.0093 |
| PAC000677_g PAC001192_s group | Bacteria;Saccharibacteria_TM7;Saccharimonas_c;Saccharimonas_o;Saccharimonas_f;PAC000677_g;PAC001192_s group | 2 | 0.0093 |
| PAC001109_g PAC001203_s | Bacteria;Tenericutes;Mollicutes;PAC001057_c;PAC001057_f;PAC001109_g;PAC001203_s | 2 | 0.0093 |
| Clostridium_g24 PAC001295_s group | Bacteria;Firmicutes;Clostridia;Clostridiales;Lachnospiraceae;Clostridium_g24;PAC001295_s group | 2 | 0.0093 |
| Sporobacter PAC001306_s | Bacteria;Firmicutes;Clostridia;Clostridiales;Ruminococcaceae;Sporobacter;PAC001306_s | 2 | 0.0093 |
| PAC001308_g PAC001308_s | Bacteria;Firmicutes;Clostridia;Clostridiales;Lachnospiraceae;PAC001308_g;PAC001308_s | 2 | 0.0093 |
| PAC000196_g PAC001335_s group | Bacteria;Firmicutes;Clostridia;Clostridiales;Lachnospiraceae;PAC000196_g;PAC001335_s group | 2 | 0.0093 |
| Oscillibacter PAC001369_s | Bacteria;Firmicutes;Clostridia;Clostridiales;Ruminococcaceae;Oscillibacter;PAC001369_s | 2 | 0.0093 |
| PAC000748_g PAC001412_s | Bacteria;Firmicutes;Clostridia;Clostridiales;Ruminococcaceae;PAC000748_g;PAC001412_s | 2 | 0.0093 |
| PAC001512_g PAC001512_s | Bacteria;Bacteroidetes;Bacteroidia;Bacteroidales;Muribaculaceae;PAC001512_g;PAC001512_s | 2 | 0.0093 |
| Oscillibacter PAC001551_s | Bacteria;Firmicutes;Clostridia;Clostridiales;Ruminococcaceae;Oscillibacter;PAC001551_s | 2 | 0.0093 |
| KE159571_g PAC001555_s | Bacteria;Firmicutes;Clostridia;Clostridiales;Lachnospiraceae;KE159571_g;PAC001555_s | 2 | 0.0093 |
| PAC000692_g PAC001577_s | Bacteria;Firmicutes;Clostridia;Clostridiales;Lachnospiraceae;PAC000692_g;PAC001577_s | 2 | 0.0093 |
| PAC001609_g PAC001650_s | Bacteria;Firmicutes;Clostridia;Clostridiales;Mogibacterium_f;PAC001609_g;PAC001650_s | 2 | 0.0093 |
| PAC001043_g PAC001752_s | Bacteria;Firmicutes;Clostridia;Clostridiales;Lachnospiraceae;PAC001043_g;PAC001752_s | 2 | 0.0093 |
| Eubacterium_g6 PAC001759_s | Bacteria;Firmicutes;Clostridia;Clostridiales;Lachnospiraceae;Eubacterium_g6;PAC001759_s | 2 | 0.0093 |
| PAC001212_g PAC001809_s | Bacteria;Firmicutes;Clostridia;Clostridiales;Lachnospiraceae;PAC001212_g;PAC001809_s | 2 | 0.0093 |
| PAC002039_g PAC002039_s | Bacteria;Firmicutes;Clostridia;Clostridiales;Lachnospiraceae;PAC002039_g;PAC002039_s | 2 | 0.0093 |
| Harryflintia PAC002149_s | Bacteria;Firmicutes;Clostridia;Clostridiales;Ruminococcaceae;Harryflintia;PAC002149_s | 2 | 0.0093 |
| Christensenella PAC002165_s | Bacteria;Firmicutes;Clostridia;Clostridiales;Christensenellaceae;Christensenella;PAC002165_s | 2 | 0.0093 |
| PAC001199_g PAC002391_s | Bacteria;Firmicutes;Clostridia;Clostridiales;Lachnospiraceae;PAC001199_g;PAC002391_s | 2 | 0.0093 |
| PAC002409_g PAC002409_s | Bacteria;Firmicutes;Clostridia;Clostridiales;Ruminococcaceae;PAC002409_g;PAC002409_s | 2 | 0.0093 |
| PAC002454_g PAC002454_s | Bacteria;Firmicutes;Clostridia;Clostridiales;Lachnospiraceae;PAC002454_g;PAC002454_s | 2 | 0.0093 |
| PAC000664_g PAC002486_s | Bacteria;Firmicutes;Clostridia;Clostridiales;Lachnospiraceae;PAC000664_g;PAC002486_s | 2 | 0.0093 |
| Coprobacter_uc | Bacteria;Bacteroidetes;Bacteroidia;Bacteroidales;Barnesiellaceae;Coprobacter;Coprobacter_uc | 2 | 0.0093 |
| PAC001066_g_uc | Bacteria;Bacteroidetes;Bacteroidia;Bacteroidales;Muribaculaceae;PAC001066_g;PAC001066_g_uc | 2 | 0.0093 |
| PAC001485_g_uc | Bacteria;Bacteroidetes;Bacteroidia;Bacteroidales;Muribaculaceae;PAC001485_g;PAC001485_g_uc | 2 | 0.0093 |
| Acrocarpospora macrocephala group | Bacteria;Actinobacteria;Actinobacteria_c;Streptosporangiales;Streptosporangiaceae;Acrocarpospora;Acrocarpospora macrocephala group | 1 | 0.0046 |
| Agathobaculum butyriciproducens group | Bacteria;Firmicutes;Clostridia;Clostridiales;Ruminococcaceae;Agathobaculum;Agathobaculum butyriciproducens group | 1 | 0.0046 |
| Aggregatibacter segnis | Bacteria;Proteobacteria;Gammaproteobacteria;Pasteurellales;Pasteurellaceae;Aggregatibacter;Aggregatibacter segnis | 1 | 0.0046 |
| Anaerostipes caccae group | Bacteria;Firmicutes;Clostridia;Clostridiales;Lachnospiraceae;Anaerostipes;Anaerostipes caccae group | 1 | 0.0046 |
| Anaerotignum lactatifermentans | Bacteria;Firmicutes;Clostridia;Clostridiales;Lachnospiraceae;Anaerotignum;Anaerotignum lactatifermentans | 1 | 0.0046 |
| Bacteroides coprophilus | Bacteria;Bacteroidetes;Bacteroidia;Bacteroidales;Bacteroidaceae;Bacteroides;Bacteroides coprophilus | 1 | 0.0046 |
| Bacteroides finegoldii | Bacteria;Bacteroidetes;Bacteroidia;Bacteroidales;Bacteroidaceae;Bacteroides;Bacteroides finegoldii | 1 | 0.0046 |
| Bacteroides oleiciplenus | Bacteria;Bacteroidetes;Bacteroidia;Bacteroidales;Bacteroidaceae;Bacteroides;Bacteroides oleiciplenus | 1 | 0.0046 |
| Bacteroides stercoris | Bacteria;Bacteroidetes;Bacteroidia;Bacteroidales;Bacteroidaceae;Bacteroides;Bacteroides stercoris | 1 | 0.0046 |
| Blautia faecis | Bacteria;Firmicutes;Clostridia;Clostridiales;Lachnospiraceae;Blautia;Blautia faecis | 1 | 0.0046 |
| Brucellaceae group | Bacteria;Proteobacteria;Alphaproteobacteria;Rhizobiales;Brucellaceae;Brucellaceae_g;Brucellaceae group | 1 | 0.0046 |
| Caenibacillus caldisaponilyticus | Bacteria;Firmicutes;Bacilli;Bacillales;Sporolactobacillaceae;Caenibacillus;Caenibacillus caldisaponilyticus | 1 | 0.0046 |
| Caldibacillus debilis | Bacteria;Firmicutes;Bacilli;Bacillales;Bacillaceae;Caldibacillus;Caldibacillus debilis | 1 | 0.0046 |
| Campylobacter concisus group | Bacteria;Proteobacteria;Epsilonproteobacteria;Campylobacterales;Campylobacteraceae;Campylobacter;Campylobacter concisus group | 1 | 0.0046 |
| Clostridioides difficile group | Bacteria;Firmicutes;Clostridia;Clostridiales;Peptostreptococcaceae;Clostridioides;Clostridioides difficile group | 1 | 0.0046 |
| Clostridium symbiosum | Bacteria;Firmicutes;Clostridia;Clostridiales;Lachnospiraceae;Clostridium_g35;Clostridium symbiosum | 1 | 0.0046 |
| Coprococcus comes group | Bacteria;Firmicutes;Clostridia;Clostridiales;Lachnospiraceae;Coprococcus_g2;Coprococcus comes group | 1 | 0.0046 |
| Dorea formicigenerans | Bacteria;Firmicutes;Clostridia;Clostridiales;Lachnospiraceae;Dorea;Dorea formicigenerans | 1 | 0.0046 |
| Dorea longicatena | Bacteria;Firmicutes;Clostridia;Clostridiales;Lachnospiraceae;Dorea;Dorea longicatena | 1 | 0.0046 |
| Enterococcus faecalis | Bacteria;Firmicutes;Bacilli;Lactobacillales;Enterococcaceae;Enterococcus;Enterococcus faecalis | 1 | 0.0046 |
| Flavobacterium lutivivi | Bacteria;Bacteroidetes;Flavobacteriia;Flavobacteriales;Flavobacteriaceae;Flavobacterium;Flavobacterium lutivivi | 1 | 0.0046 |
| Flavonifractor plautii | Bacteria;Firmicutes;Clostridia;Clostridiales;Ruminococcaceae;Pseudoflavonifractor;Flavonifractor plautii | 1 | 0.0046 |
| Gordonia terrae group | Bacteria;Actinobacteria;Actinobacteria_c;Corynebacteriales;Nocardiaceae;Gordonia;Gordonia terrae group | 1 | 0.0046 |
| Haemophilus parainfluenzae group | Bacteria;Proteobacteria;Gammaproteobacteria;Pasteurellales;Pasteurellaceae;Haemophilus;Haemophilus parainfluenzae group | 1 | 0.0046 |
| Herbiconiux ginsengi group | Bacteria;Actinobacteria;Actinobacteria_c;Micrococcales;Microbacteriaceae;Herbiconiux;Herbiconiux ginsengi group | 1 | 0.0046 |
| Holdemanella biformis | Bacteria;Firmicutes;Erysipelotrichi;Erysipelotrichales;Erysipelotrichaceae;Holdemanella;Holdemanella biformis | 1 | 0.0046 |
| Intestinibacter bartlettii | Bacteria;Firmicutes;Clostridia;Clostridiales;Peptostreptococcaceae;Intestinibacter;Intestinibacter bartlettii | 1 | 0.0046 |
| Lysinibacillus xylanilyticus group | Bacteria;Firmicutes;Bacilli;Bacillales;Planococcaceae;Lysinibacillus;Lysinibacillus xylanilyticus group | 1 | 0.0046 |
| Mitsuokella jalaludinii | Bacteria;Firmicutes;Negativicutes;Selenomonadales;Selenomonadaceae;Mitsuokella;Mitsuokella jalaludinii | 1 | 0.0046 |
| Mogibacterium pumilum group | Bacteria;Firmicutes;Clostridia;Clostridiales;Mogibacterium_f;Mogibacterium;Mogibacterium pumilum group | 1 | 0.0046 |
| Morganella morganii group | Bacteria;Proteobacteria;Gammaproteobacteria;Enterobacterales;Morganellaceae;Morganella;Morganella morganii group | 1 | 0.0046 |
| Mucilaginibacter ximonensis | Bacteria;Bacteroidetes;Sphingobacteriia;Sphingobacteriales;Sphingobacteriaceae;Mucilaginibacter;Mucilaginibacter ximonensis | 1 | 0.0046 |
| Paraprevotella clara | Bacteria;Bacteroidetes;Bacteroidia;Bacteroidales;Prevotellaceae;Paraprevotella;Paraprevotella clara | 1 | 0.0046 |
| Phocea massiliensis | Bacteria;Firmicutes;Clostridia;Clostridiales;Ruminococcaceae;Phocea;Phocea massiliensis | 1 | 0.0046 |
| Prevotella copri | Bacteria;Bacteroidetes;Bacteroidia;Bacteroidales;Prevotellaceae;Prevotella;Prevotella copri | 1 | 0.0046 |
| Pseudactinotalea terrae group | Bacteria;Actinobacteria;Actinobacteria_c;Micrococcales;Cellulomonadaceae;Pseudactinotalea;Pseudactinotalea terrae group | 1 | 0.0046 |
| Rhizobium leguminosarum group | Bacteria;Proteobacteria;Alphaproteobacteria;Rhizobiales;Rhizobiaceae;Rhizobium;Rhizobium leguminosarum group | 1 | 0.0046 |
| Staphylococcus sciuri group | Bacteria;Firmicutes;Bacilli;Bacillales;Staphylococcaceae;Staphylococcus;Staphylococcus sciuri group | 1 | 0.0046 |
| Streptococcus pneumoniae group | Bacteria;Firmicutes;Bacilli;Lactobacillales;Streptococcaceae;Streptococcus;Streptococcus pneumoniae group | 1 | 0.0046 |
| Veillonella atypica | Bacteria;Firmicutes;Negativicutes;Veillonellales;Veillonellaceae;Veillonella;Veillonella atypica | 1 | 0.0046 |
| Weissella kandleri group | Bacteria;Firmicutes;Bacilli;Lactobacillales;Leuconostocaceae;Weissella;Weissella kandleri group | 1 | 0.0046 |
| LLKB_g AB626939_s | Bacteria;Firmicutes;Clostridia;Clostridiales;Lachnospiraceae;LLKB_g;AB626939_s | 1 | 0.0046 |

| | | | |
|---|---|---|---|
| CCMM_g CCMM_s | Bacteria;Firmicutes;Erysipelotrichi;Erysipelotrichales;Erysipelotrichaceae;CCMM_g;CCMM_s | 1 | 0.0046 |
| Bacteroides DQ799557_s | Bacteria;Bacteroidetes;Bacteroidia;Bacteroidales;Bacteroidaceae;Bacteroides;DQ799557_s | 1 | 0.0046 |
| AB021325_g EF125420_s | Bacteria;Actinobacteria;Actinobacteria_c;Frankiales;AB021325_f;AB021325_g;EF125420_s | 1 | 0.0046 |
| PAC000661_g EF445156_s | Bacteria;Firmicutes;Clostridia;Clostridiales;Ruminococcaceae;PAC000661_g;EF445156_s | 1 | 0.0046 |
| Clostridium_g21 EU509115_s | Bacteria;Firmicutes;Clostridia;Clostridiales;Lachnospiraceae;Clostridium_g21;EU509115_s | 1 | 0.0046 |
| KE159600_g EU511112_s | Bacteria;Firmicutes;Clostridia;Clostridiales;Lachnospiraceae;KE159600_g;EU511112_s | 1 | 0.0046 |
| Clostridium_g24 FCEY_s | Bacteria;Firmicutes;Clostridia;Clostridiales;Lachnospiraceae;Clostridium_g24;FCEY_s | 1 | 0.0046 |
| Bacteroides FJ684447_s | Bacteria;Bacteroidetes;Bacteroidia;Bacteroidales;Bacteroidaceae;Bacteroides;FJ684447_s | 1 | 0.0046 |
| Acetatifactor FJ879577_s | Bacteria;Firmicutes;Clostridia;Clostridiales;Lachnospiraceae;Acetatifactor;FJ879577_s | 1 | 0.0046 |
| Oscillibacter FJ881219_s | Bacteria;Firmicutes;Clostridia;Clostridiales;Ruminococcaceae;Oscillibacter;FJ881219_s | 1 | 0.0046 |
| PAC000683_g GQ448528_s | Bacteria;Firmicutes;Clostridia;Clostridiales;Ruminococcaceae;PAC000683_g;GQ448528_s | 1 | 0.0046 |
| HQ158649_g GU454872_s | Bacteria;Bacteroidetes;Sphingobacteriia;Saprospirales;Saprospiraceae;HQ158649_g;GU454872_s | 1 | 0.0046 |
| Oscillibacter HM124063_s | Bacteria;Firmicutes;Clostridia;Clostridiales;Ruminococcaceae;Oscillibacter;HM124063_s | 1 | 0.0046 |
| Paludibacter HM445934_s | Bacteria;Bacteroidetes;Bacteroidia;Bacteroidales;Porphyromonadaceae;Paludibacter;HM445934_s | 1 | 0.0046 |
| GQ500701_g HQ827993_s | Bacteria;Chloroflexi;Anaerolineae;Anaerolinaeles;Anaerolinaceae;GQ500701_g;HQ827993_s | 1 | 0.0046 |
| Flavobacterium JF220437_s | Bacteria;Bacteroidetes;Flavobacteria;Flavobacteriales;Flavobacteriaceae;Flavobacterium;JF220437_s | 1 | 0.0046 |
| Oscillibacter JPJG_s | Bacteria;Firmicutes;Clostridia;Clostridiales;Ruminococcaceae;Oscillibacter;JPJG_s | 1 | 0.0046 |
| DQ129389_g JQ624352_s | Bacteria;Chloroflexi;Thermomicrobia;DQ129389_c;DQ129389_f;DQ129389_g;JQ624352_s | 1 | 0.0046 |
| Rothia KV831974_s group | Bacteria;Actinobacteria;Actinobacteria_c;Micrococcales;Micrococcaceae;Rothia;KV831974_s group | 1 | 0.0046 |
| Clostridium_g24 PAC000191_s | Bacteria;Firmicutes;Clostridia;Clostridiales;Lachnospiraceae;Clostridium_g24;PAC000191_s | 1 | 0.0046 |
| PAC000197_g PAC000197_s | Bacteria;Tenericutes;Mollicutes;PAC001057_c;PAC000197_f;PAC000197_g;PAC000197_s | 1 | 0.0046 |
| Anaerotignum PAC000685_s | Bacteria;Firmicutes;Clostridia;Clostridiales;Lachnospiraceae;Anaerotignum;PAC000685_s | 1 | 0.0046 |
| PAC000740_g PAC000740_s | Bacteria;Firmicutes;Clostridia;Clostridiales;Lachnospiraceae;PAC000740_g;PAC000740_s | 1 | 0.0046 |
| Eubacterium_g23 PAC001035_s | Bacteria;Firmicutes;Clostridia;Clostridiales;Ruminococcaceae;Eubacterium_g23;PAC001035_s | 1 | 0.0046 |
| Prevotella PAC001042_s | Bacteria;Bacteroidetes;Bacteroidia;Bacteroidales;Prevotellaceae;Prevotella;PAC001042_s | 1 | 0.0046 |
| PAC000186_g PAC001065_s | Bacteria;Bacteroidetes;Bacteroidia;Bacteroidales;Muribaculaceae;PAC000186_g;PAC001065_s | 1 | 0.0046 |
| PAC000197_g PAC001123_s | Bacteria;Tenericutes;Mollicutes;PAC001057_c;PAC000197_f;PAC000197_g;PAC001123_s | 1 | 0.0046 |
| PAC000186_g PAC001128_s | Bacteria;Bacteroidetes;Bacteroidia;Bacteroidales;Muribaculaceae;PAC000186_g;PAC001128_s | 1 | 0.0046 |
| Christensenella PAC001140_s | Bacteria;Firmicutes;Clostridia;Clostridiales;Christensenellaceae;Christensenella;PAC001140_s | 1 | 0.0046 |
| PAC001149_g PAC001149_s | Bacteria;Firmicutes;Clostridia;Clostridiales;Lachnospiraceae;PAC001149_g;PAC001149_s | 1 | 0.0046 |
| Oscillibacter PAC001157_s | Bacteria;Firmicutes;Clostridia;Clostridiales;Ruminococcaceae;Oscillibacter;PAC001157_s | 1 | 0.0046 |
| Eubacterium_g4 PAC001174_s | Bacteria;Firmicutes;Clostridia;Clostridiales;Lachnospiraceae;Eubacterium_g4;PAC001174_s | 1 | 0.0046 |
| Bacteroides PAC001204_s | Bacteria;Bacteroidetes;Bacteroidia;Bacteroidales;Bacteroidaceae;Bacteroides;PAC001204_s | 1 | 0.0046 |
| Eubacterium_g6 PAC001227_s | Bacteria;Firmicutes;Clostridia;Clostridiales;Lachnospiraceae;Eubacterium_g6;PAC001227_s | 1 | 0.0046 |
| Ruminococcus_g2 PAC001256_s | Bacteria;Firmicutes;Clostridia;Clostridiales;Ruminococcaceae;Ruminococcus_g2;PAC001256_s | 1 | 0.0046 |
| PAC001276_g PAC001276_s | Bacteria;Bacteroidetes;Bacteroidia;Bacteroidales;Muribaculaceae;PAC001276_g;PAC001276_s | 1 | 0.0046 |
| PAC001138_g PAC001305_s | Bacteria;Firmicutes;Clostridia;Clostridiales;Lachnospiraceae;PAC001138_g;PAC001305_s | 1 | 0.0046 |
| Pseudoflavonifractor PAC001318_s | Bacteria;Firmicutes;Clostridia;Clostridiales;Ruminococcaceae;Pseudoflavonifractor;PAC001318_s | 1 | 0.0046 |
| PAC001372_g PAC001372_s | Bacteria;Firmicutes;Clostridia;Clostridiales;Lachnospiraceae;PAC001372_g;PAC001372_s | 1 | 0.0046 |
| PAC001386_g PAC001386_s | Bacteria;Firmicutes;Clostridia;Clostridiales;Lachnospiraceae;PAC001386_g;PAC001386_s | 1 | 0.0046 |
| PAC000692_g PAC001387_s | Bacteria;Firmicutes;Clostridia;Clostridiales;Lachnospiraceae;PAC000692_g;PAC001387_s | 1 | 0.0046 |
| PAC001092_g PAC001388_s | Bacteria;Firmicutes;Clostridia;Clostridiales;Lachnospiraceae;PAC001092_g;PAC001388_s | 1 | 0.0046 |
| Christensenella PAC001413_s | Bacteria;Firmicutes;Clostridia;Clostridiales;Christensenellaceae;Christensenella;PAC001413_s | 1 | 0.0046 |
| Anaerotignum PAC001464_s | Bacteria;Firmicutes;Clostridia;Clostridiales;Lachnospiraceae;Anaerotignum;PAC001464_s | 1 | 0.0046 |
| KE159538_g PAC001504_s | Bacteria;Firmicutes;Clostridia;Clostridiales;Lachnospiraceae;KE159538_g;PAC001504_s | 1 | 0.0046 |
| Paludicola PAC001506_s | Bacteria;Firmicutes;Clostridia;Clostridiales;Ruminococcaceae;Paludicola;PAC001506_s | 1 | 0.0046 |
| PAC000664_g PAC001515_s | Bacteria;Firmicutes;Clostridia;Clostridiales;Lachnospiraceae;PAC000664_g;PAC001515_s | 1 | 0.0046 |
| Acutalibacter PAC001519_s | Bacteria;Firmicutes;Clostridia;Clostridiales;Ruminococcaceae;Acutalibacter;PAC001519_s | 1 | 0.0046 |
| PAC000661_g PAC001583_s | Bacteria;Firmicutes;Clostridia;Clostridiales;Ruminococcaceae;PAC000661_g;PAC001583_s | 1 | 0.0046 |
| PAC001588_g PAC001588_s | Bacteria;Firmicutes;Clostridia;Clostridiales;Lachnospiraceae;PAC001588_g;PAC001588_s | 1 | 0.0046 |
| PAC001116_g PAC001589_s | Bacteria;Firmicutes;Clostridia;Clostridiales;Lachnospiraceae;PAC001116_g;PAC001589_s | 1 | 0.0046 |
| FTRU_g PAC001607_s | Bacteria;Firmicutes;Clostridia;Clostridiales;Ruminococcaceae;FTRU_g;PAC001607_s | 1 | 0.0046 |
| PAC001637_g PAC001653_s | Bacteria;Firmicutes;Clostridia;Clostridiales;Ruminococcaceae;PAC001637_g;PAC001653_s | 1 | 0.0046 |
| PAC001063_g PAC001664_s | Bacteria;Bacteroidetes;Bacteroidia;Bacteroidales;Muribaculaceae;PAC001063_g;PAC001664_s | 1 | 0.0046 |
| PAC000692_g PAC001666_s | Bacteria;Firmicutes;Clostridia;Clostridiales;Lachnospiraceae;PAC000692_g;PAC001666_s | 1 | 0.0046 |
| PAC001092_g PAC001691_s | Bacteria;Firmicutes;Clostridia;Clostridiales;Lachnospiraceae;PAC001092_g;PAC001691_s | 1 | 0.0046 |
| Frisingicoccus PAC001702_s | Bacteria;Firmicutes;Clostridia;Clostridiales;Lachnospiraceae;Frisingicoccus;PAC001702_s | 1 | 0.0046 |
| Ruminococcus_g4 PAC001711_s | Bacteria;Firmicutes;Clostridia;Clostridiales;Lachnospiraceae;Ruminococcus_g4;PAC001711_s | 1 | 0.0046 |
| PAC001723_g PAC001723_s | Bacteria;Firmicutes;Clostridia;Clostridiales;Lachnospiraceae;PAC001723_g;PAC001723_s | 1 | 0.0046 |
| PAC001103_g PAC001724_s group | Bacteria;Firmicutes;Clostridia;Clostridiales;Lachnospiraceae;PAC001103_g;PAC001724_s group | 1 | 0.0046 |
| Frisingicoccus PAC001728_s group | Bacteria;Firmicutes;Clostridia;Clostridiales;Lachnospiraceae;Frisingicoccus;PAC001728_s group | 1 | 0.0046 |
| PAC000692_g PAC001745_s | Bacteria;Firmicutes;Clostridia;Clostridiales;Lachnospiraceae;PAC000692_g;PAC001745_s | 1 | 0.0046 |
| PAC001765_g PAC001765_s | Bacteria;Bacteroidetes;Bacteroidia;Bacteroidales;Muribaculaceae;PAC001765_g;PAC001765_s | 1 | 0.0046 |
| PAC001068_g PAC001766_s | Bacteria;Bacteroidetes;Bacteroidia;Bacteroidales;Muribaculaceae;PAC001068_g;PAC001766_s | 1 | 0.0046 |
| PAC001138_g PAC001775_s | Bacteria;Firmicutes;Clostridia;Clostridiales;Lachnospiraceae;PAC001138_g;PAC001775_s | 1 | 0.0046 |
| PAC000661_g PAC001777_s group | Bacteria;Firmicutes;Clostridia;Clostridiales;Ruminococcaceae;PAC000661_g;PAC001777_s group | 1 | 0.0046 |
| Stenotrophobacter PAC001842_s | Bacteria;Acidobacteria;Blastocatellia;Blastocatellales;Blastocatellaceae;Stenotrophobacter;PAC001842_s | 1 | 0.0046 |
| Harryflintia PAC002011_s | Bacteria;Firmicutes;Clostridia;Clostridiales;Ruminococcaceae;Harryflintia;PAC002011_s | 1 | 0.0046 |
| Sporobacter PAC002139_s | Bacteria;Firmicutes;Clostridia;Clostridiales;Ruminococcaceae;Sporobacter;PAC002139_s | 1 | 0.0046 |
| PAC002152_g PAC002152_s | Bacteria;Firmicutes;Clostridia;Clostridiales;Lachnospiraceae;PAC002152_g;PAC002152_s | 1 | 0.0046 |
| Eubacterium_g6 PAC002177_s | Bacteria;Firmicutes;Clostridia;Clostridiales;Lachnospiraceae;Eubacterium_g6;PAC002177_s | 1 | 0.0046 |
| KE159571_g PAC002348_s | Bacteria;Firmicutes;Clostridia;Clostridiales;Lachnospiraceae;KE159571_g;PAC002348_s | 1 | 0.0046 |
| Lachnospira PAC002369_s | Bacteria;Firmicutes;Clostridia;Clostridiales;Lachnospiraceae;Lachnospira;PAC002369_s | 1 | 0.0046 |
| PAC001127_g PAC002399_s | Bacteria;Bacteroidetes;Bacteroidia;Bacteroidales;Muribaculaceae;PAC001127_g;PAC002399_s | 1 | 0.0046 |
| Frisingicoccus PAC002470_s | Bacteria;Firmicutes;Clostridia;Clostridiales;Lachnospiraceae;Frisingicoccus;PAC002470_s | 1 | 0.0046 |
| Pseudoflavonifractor PAC002475_s | Bacteria;Firmicutes;Clostridia;Clostridiales;Ruminococcaceae;Pseudoflavonifractor;PAC002475_s | 1 | 0.0046 |
| PAC000720_g PAC002507_s | Bacteria;Firmicutes;Clostridia;Clostridiales;Lachnospiraceae;PAC000720_g;PAC002507_s | 1 | 0.0046 |
| Akkermansia_uc | Bacteria;Verrucomicrobia;Verrucomicrobiae;Verrucomicrobiales;Akkermansiaceae;Akkermansia;Akkermansia_uc | 1 | 0.0046 |
| Cellulomonas_uc | Bacteria;Actinobacteria;Actinobacteria_c;Micrococcales;Cellulomonadaceae;Cellulomonas;Cellulomonas_uc | 1 | 0.0046 |
| Enterobacter_uc | Bacteria;Proteobacteria;Gammaproteobacteria;Enterobacterales;Enterobacteriaceae;Enterobacter;Enterobacter_uc | 1 | 0.0046 |
| Gordonia_uc | Bacteria;Actinobacteria;Actinobacteria_c;Corynebacteriales;Nocardiaceae;Gordonia;Gordonia_uc | 1 | 0.0046 |
| HM124015_g_uc | Bacteria;Firmicutes;Clostridia;Clostridiales;Ruminococcaceae;HM124015_g;HM124015_g_uc | 1 | 0.0046 |
| KE159600_g_uc | Bacteria;Firmicutes;Clostridia;Clostridiales;Lachnospiraceae;KE159600_g;KE159600_g_uc | 1 | 0.0046 |
| PAC000664_g_uc | Bacteria;Firmicutes;Clostridia;Clostridiales;Lachnospiraceae;PAC000664_g;PAC000664_g_uc | 1 | 0.0046 |
| PAC001092_g_uc | Bacteria;Firmicutes;Clostridia;Clostridiales;Lachnospiraceae;PAC001092_g;PAC001092_g_uc | 1 | 0.0046 |
